# Supplementary material for: Nutritional Assessment of Baby Food Available in Italy
Source: Nutrients. 2022 Sep 9;14(18):3722. doi: 10.3390/nu14183722 (PMC9501114; doi:10.3390/nu14183722)
Supplement: Supplementary file 1 [file nutrients-14-03722-s001.zip › nutrients-1869107-supplementary.pdf]

CODEX ALIMENTARIUS
INTERNATIONAL FOOD STANDARDS
Food and Agriculture Organization of the United Nations
World Health Organization
E-mail: codex@fao.org - www.codexalimentarius.org

STANDARD FOR PROCESSED CEREAL-BASED FOODS FOR INFANTS AND YOUNG CHILDREN

CXS 74-1981

Adopted in 1981. Revised in 2006. Amended in 2017, 2019.

1. SCOPE

This Standard covers processed cereal-based foods intended for feeding infants as a complementary food generally from the age of 6 months onwards, taking into account infants' individual nutritional requirements, and for feeding young children as part of a progressively diversified diet, in accordance with the Global Strategy for Infant and Young Child Feeding and World Health Assembly Resolution WHA54.2 (2001).

2. DESCRIPTION

Processed cereal-based foods are prepared primarily from one or more milled cereals, which should constitute at least 25% of the final mixture on a dry weight basis.

2.1 Product definitions

Four categories are distinguished:

2.1.1 Products consisting of cereals which are or have to be prepared for consumption with milk or other appropriate nutritious liquids;

2.1.2 Cereals with an added high protein food which are or have to be prepared for consumption with water or other appropriate protein-free liquid;

2.1.3 Pasta which are to be used after cooking in boiling water or other appropriate liquids;

2.1.4 Rusks and biscuits which are to be used either directly or, after pulverization, with the addition of water, milk or other suitable liquids.

2.2 Other definitions

2.2.1 The term infant means a person not more than 12 months of age.

2.2.2 The term young children means persons from the age of more than 12 months up to the age of three years (36 months).

3. ESSENTIAL COMPOSITION AND QUALITY FACTORS

3.1 Essential composition

3.1.1 The four categories listed in 2.1.1 to 2.1.4 are prepared primarily from one or more milled cereal products, such as wheat, rice, barley, oats, rye, maize, millet, sorghum and buckwheat. They may also contain legumes (pulses), starchy roots (such as arrow root, yam or cassava) or starchy stems or oil seeds in smaller proportions.

3.1.2 The requirements concerning energy and nutrients refer to the product ready for use as marketed or prepared according to the instructions of the manufacturer, unless otherwise specified.

3.2 Energy density

The energy density of cereal-based foods should not be less than 3.3 kJ/g (0.8 kcal/g).

3.3 Protein

3.3.1 The chemical index of the added protein shall be equal to at least 80% of that of the reference protein casein or the Protein Efficiency Ratio (PER) of the protein in the mixture shall be equal to at least 70% of that of the reference protein casein. In all cases, the addition of amino acids is permitted solely for the purpose of improving the nutritional value of the protein mixture, and only in the proportions necessary for that purpose. Only natural forms of L-amino acids should be used.

3.3.2 For products mentioned in points 2.1.2 and 2.1.4, the protein content shall not exceed 1.3 g/100 kJ (5.5 g/100 kcal).

3.3.3 For products mentioned in point 2.1.2 the added protein content shall not be less than 0.48 g/100 kJ (2 g/100 kcal).

3.3.4 For biscuits mentioned in point 2.1.4 made with the addition of a high protein food, and presented as such, the added protein shall not be less than 0.36 g/100 kJ (1.5 g/ 100 kcal).

3.4 Carbohydrates

3.4.1 If sucrose, fructose, glucose, glucose syrup or honey are added to products mentioned in points 2.1.1 and 2.1.4:

- the amount of added carbohydrates from these sources shall not exceed 1.8 g/100 kJ (7.5 g/100 kcal);
- the amount of added fructose shall not exceed 0.9 g/100 kJ (3.75 g/100 kcal).

3.4.2 If sucrose, fructose, glucose, glucose syrup or honey are added to products mentioned in point 2.1.2:

- the amount of added carbohydrates from these sources shall not exceed 1.2 g/100 kJ (5 g/100 kcal);
- the amount of added fructose shall not exceed 0.6 g/100 kJ (2.5 g/100 kcal).

3.5 Lipids

3.5.1 For products mentioned in point 2.1.2 the lipid content shall not exceed 1.1g/100 kJ (4.5 g/100 kcal). If the lipid content exceeds 0.8g/100kJ (3.3g/100kcal):

- the amount of linoleic acid (in the form of triglycerides=linoleates) shall not be less than 70 mg/100 kJ (300 mg/100 kcal) and shall not exceed 285 mg/100 kJ (1200 mg/100 kcal);
- the amount of lauric acid shall not exceed 15% of the total lipid content;
- the amount of myristic acid shall not exceed 15% of the total lipid content.

3.5.2 Product categories 2.1.1 and 2.1.4 shall not exceed a maximum lipid content of 0.8 g /100 kJ (3.3 g/100 kcal).

3.6 Minerals

3.6.1 The sodium content of the products described in Sections 2.1.1 to 2.1.4 of this Standard shall not exceed 24 mg/100 kJ (100 mg/100 kcal) of the ready-to-eat product.

3.6.2 The calcium content shall not be less than 20 mg/100 kJ (80 mg/100 kcal) for products mentioned in points 2.1.2.

3.6.3 The calcium content shall not be less than 12 mg/100 kJ (50 mg/100 kcal) for products mentioned in point 2.1.4 manufactured with the addition of milk and presented as such.

3.7 Vitamins

3.7.1 The amount of vitamin B1 (thiamin) shall not be less than 12.5µg/100 kJ (50µg/100 kcal).

3.7.2 For products mentioned in 2.1.2, the amount of vitamin A and vitamin D shall be within the following limits:

	**µg/100kJ**	**µg/100kcal**
**vitamin A**<br>**(µg retinol equivalents)**	14-43	60 – 180
**vitamin D**	0.25-0.75	1 – 3

These limits are also applicable to other processed cereal-based foods when vitamin A or D are added.

3.7.3 Reductions of the maximum amounts for vitamin A and Vitamin D referred to in 3.7.2 and the addition of vitamins and minerals for which specifications are not set above shall be in conformity with the legislation of the country in which the product is sold.

3.7.4 Vitamins and/or minerals added should be selected from the *Advisory Lists of Nutrient Compounds for Use in Foods for Special Dietary Uses intended for Infants and Children* (CXG 10-1979).

3.8 Optional ingredients

3.8.1 In addition to the ingredients listed under 3.1, other ingredients suitable for infants who are more than six months of age and for young children can be used.

3.8.2 Products containing honey or maple syrup should be processed in such a way as to destroy spores of *Clostridium botulinum*, if present.

3.8.3 Only L(+) lactic acid producing cultures may be used.

3.9 Flavourings

The following flavourings may be used:

- Natural fruit extracts and vanilla extract: GMP
- Ethyl vanillin and vanillin: 7 mg/100 g RTU

3.10 Quality factors

3.10.1 All ingredients, including optional ingredients, shall be clean, safe, suitable and of good quality.

3.10.2 All processing and drying should be carried out in a manner that minimizes loss of nutritive value, particularly protein quality.

3.10.3 The moisture content of the products shall be governed by good manufacturing practice for the individual product categories and shall be at such a level that there is a minimum loss of nutritive value and at which microorganisms cannot multiply.

3.11 Consistency and particle size

3.11.1 When prepared according to the label directions for use, processed cereal-based foods should have a texture appropriate for the spoon feeding of infants or young children of the age for which the product is intended.

3.11.2 Rusks and biscuits may be used in the dry form so as to permit and encourage chewing or they may be used in a liquid form, by mixing with water or other suitable liquid that would be similar in consistency to dry cereals.

3.12 Specific prohibition

The product and its components shall not have been treated by ionizing radiation.

The use of partially hydrogenated fats for these products is prohibited.

4. FOOD ADDITIVES

Only the food additives listed in this Section or in the *Advisory Lists of Nutrient Compounds for Use in Foods for Special Dietary Uses intended for Infants and Children* (CXG 10-1979) may be present in the foods described in Section 2.1 of this Standard, as a result of carry-over from a raw material or other ingredient (including food additive) used to produce the food, subject to the following conditions:

a) The amount of the food additive in the raw materials or other ingredients (including food additives) does not exceed the maximum level specified; and

b) The food into which the food additive is carried over does not contain the food additive in greater quantity than would be introduced by the use of the raw materials or ingredients under good manufacturing practice, consistent with the provisions on carry-over in the Preamble of the *General Standard for Food Additives* (CXS 192-1995).

The following additives are permitted in the preparation of processed cereal-based foods for infants and young children, as described in Section 2.1 of this Standard (in 100 g of product, ready for consumption prepared following manufacturer's instructions unless otherwise indicated).

**INS no.**		**Maximum level**
**Emulsifiers**		
322	Lecithins	1500 mg
471	Mono- and diglycerides	500 mg<br>Singly or in combination
472a	Acetic and fatty acid esters of glycerol	
472b	Lactic and fatty acid esters of glycerol	
472c	Citric and fatty acid esters of glycerol	
**Acidity Regulators**		
500 ii	Sodium hydrogen carbonate	GMP
501 ii	Potassium hydrogen carbonate	GMP
170 i	Calcium carbonate	GMP
270	L(+) Lactic acid	GMP
330	Citric acid	GMP
260	Acetic acid	GMP
261	Potassium acetates	
262 i	Sodium acetate	
263	Calcium acetate	
296	Malic acid (DL) – L(+)-form only	

325	Sodium lactate (solution) – L(+)-form only	
326	Potassium lactate (solution) – L(+)-form only	
327	Calcium lactate – L(+)-form only	
331 i	Monosodium citrate	
331 ii	Trisodium citrate	
332 i	Monopotassium citrate	
332 ii	Tripotassium citrate	
333	Calcium citrate	
507	Hydrochloric acid	
524	Sodium hydroxide	
525	Potassium hydroxide	
526	Calcium hydroxide	
575	Glucono delta-lactone	GMP
334	L(+)-Tartaric acid – L(+)form only	500 mg<br>Singly or in combination<br><br>Tartrates as residue in biscuits and rusks
335 ii	Disodium tartrate	
337	Potassium sodium L(+)tartrate<br>L(+)form only	
338	Orthophosphoric acid	Only for pH adjustment<br>440 mg<br>Singly or in combination as phosphorous
339 i	Monosodium orthophosphate	
339 ii	Disodium orthophosphate	
339 iii	Trisodium orthophosphate	
340 i	Monopotassium orthophosphate	
340 ii	Dipotassium orthophosphate	
340 iii	Tripotassium orthophosphate	
341 i	Monocalcium orthophosphate	
341 ii	Dicalcium orthophosphate	
341 iii	Tricalcium orthophosphate	
**Antioxidants**		
306	Mixed tocopherols concentrate	300 mg/kg fat or oil basis, Singly or in combination
307	Alpha-tocopherol	
304	L-Ascorbyl palmitate	200 mg/kg fat
300	L-Ascorbic acid	50 mg, expressed as ascorbic acid
301	Sodium ascorbate	
303	Potassium ascorbate	
302	Calcium ascorbate	20 mg, expressed as ascorbic acid
**Raising Agents**		
503 i	Ammonium carbonate	Limited by GMP

503 ii	Ammonium hydrogen carbonate	
500 i	Sodium carbonate	
500 ii	Sodium hydrogen carbonate	
**Thickeners**		
410	Carob bean gum	1000 mg singly or in combination<br><br>2000 mg in gluten-free cereal-based foods
412	Guar gum	
414	Gum arabic	
415	Xanthan gum	
440	Pectins (Amidated and Non-Amidated)	
1404	Oxidized starch	5000 mg<br>Singly or in combination
1410	Monostarch phosphate	
1412	Distarch phosphate	
1413	Phosphated distarch phosphate	
1414	Acetylated distarch phosphate	
1422	Acetylated distarch adipate	
1420	Starch acetate esterified with acetic anhydride	
1450	Starch sodium octenyl succinate	
1451	Acetylated oxidized starch	
**Anticaking Agents**		
551	Silicon dioxide (amorphous)	200 mg for dry cereals only
**Packaging Gases**		
290	Carbon dioxide	GMP
941	Nitrogen	GMP

5. CONTAMINANTS

5.1 Pesticide residues

The product shall be prepared with special care under good manufacturing practices, so that residues of those pesticides which may be required in the production, storage or processing of the raw materials or the finished food ingredient do not remain, or, if technically unavoidable, are reduced to the maximum extent possible.

These measures shall take into account the specific nature of the products concerned and the specific population group for which they are intended.

5.2 Other contaminants

The product shall be free from residues of hormones, antibiotics as determined by means of agreed methods of analysis and practically free from other contaminants, especially pharmacologically active substances.

6. HYGIENE

It is recommended that the products covered by the provisions of this Standard be prepared and handled in accordance with the appropriate sections of the *General Principles of Food Hygiene* (CXC 1-1969), and other relevant Codex texts such as Codes of Hygienic Practice and Codes of Practice.

The product should comply with any microbiological criteria established in accordance with the *Principles and Guidelines for the Establishment and Application of Microbiological Criteria Related to Foods* (CXG 21-1997).

7. PACKAGING

7.1 The product shall be packed in containers which will safeguard the hygienic and other qualities of the food.

7.2 The containers, including packaging material, shall be made only of substances which are safe and suitable for their intended use. Where the Codex Alimentarius Commission has established a standard for any such substance used as packaging material, that standard shall apply.

8. LABELLING

8.1.1 The requirements of the *General Standard for the Labelling of Prepackaged Foods* (CXS 1-1985), the *Guidelines on Nutrition Labelling* (CXG 2-1985) and the *Guidelines for Use of Nutrition and Health Claims* (CXG 23-1997) apply to this Standard. With specific reference to Section 7 of the *General Standard for the Labelling of Prepackaged Foods* national jurisdictions may further restrict the use of pictorial devices.

8.1.2 Taking into account paragraph 1.4 of the *Guidelines for Use of Nutrition and Health Claims*, nutrition claims may be permitted under national legislation for the foods that are the subject of the standard provided that they have been demonstrated in rigorous studies with adequate scientific standards.

8.1.3 Any indication required in the labelling should be made in the appropriate language(s) of the country in which the product is sold.

8.2 The name of the food

The name of the food shall be "Dry Cereal for Infants (and/or Young Children)", "Rusks for Infants (and/or Young Children)" or "Biscuits (or "Milk Biscuits") for Infants (and/or Young Children)" or "Pasta for Infants (and/or Young Children)", or any appropriate designation indicating the true nature of the food, in accordance with national legislation.

8.3 List of ingredients

8.3.1 A complete list of ingredients shall be declared on the label in descending order of proportion except that in the case of added vitamins and minerals, these may be arranged as separate groups for vitamins and minerals, respectively, and within these groups the vitamins and minerals need not be listed in descending order of proportion.

8.3.2 The specific name shall be declared for ingredients and food additives. In addition, appropriate class names for these ingredients and additives may be included on the label.

8.4 Declaration of nutritive value

8.4.1 The declaration of nutrition information shall contain the following information which should be in the following order:

a) The energy value, expressed in kilocalories (kcal) and kilojoules (kJ), and the amount of protein, carbohydrate and fat expressed in grammes (g) per 100 g or 100 ml of the food as sold, and where appropriate, as per specified quantity of the food as suggested for consumption;

b) The average amount of each vitamin and mineral for which specific levels are defined in section 3.6 and 3.7 expressed in numerical form per 100g or 100 ml of the food as sold and, where appropriate, as per specified quantity of the food as suggested for consumption;

c) Any other nutritional information required by national legislation.

8.4.2 The labelling may bear the average amount of the vitamins and minerals when their declaration is not covered by the provisions of section 8.4.1 (b) expressed in numerical form per 100g or 100 ml of the product as sold and, where appropriate, per specified quantity of the food as suggested for consumption.

8.5 Date marking and storage instructions

8.5.1 The date of minimum durability (preceded by the words "best before") shall be declared by the day, month and year in uncoded numerical sequence except that for products with a shelf-life of more than three months, the month and year will suffice. The month may be indicated by letters in those countries where such use will not confuse the consumer. In the case of products requiring a declaration of month and year only, and the shelf-life of the product is valid to the end of a given year, the expression "end (stated year)" may be used as an alternative.

8.5.2 In addition to the date, any special conditions for the storage of the food shall be indicated if the validity of the date depends thereon.

8.5.3 Where practicable, storage instructions shall be in close proximity to the date marking.

8.6 Information for utilization

8.6.1 Directions as to the preparation and use of the food, and its storage and keeping before and after the container has been opened, shall appear on the label and may also appear on the accompanying leaflet.

8.6.2 For products covered by 2.1.1, directions on the label shall state "Milk or formula but no water shall be used for dilution or mixing" or an equivalent statement.

8.6.3 When the product is composed of gluten-free ingredients and food additives, the label may show the statement "gluten-free"[1].

8.6.4 The label shall indicate clearly from which age the product is recommended for use. This age shall not be less than six months for any product. In addition, the label shall include a statement indicating that the decision when precisely to begin complementary feeding, including any exception to six months of age, should be made in consultation with a health worker, based on the individual infant's specific growth and development needs. Additional requirements in this respect may be made in accordance with the legislation of the country in which the product is sold.

8.7 Additional requirements

The products covered by this standard are not breast-milk substitutes and shall not be presented as such.

9. METHODS OF ANALYSIS AND SAMPLING

See Section on methods in the Standard for Infant Formula.

In addition:

<u>Detection of Irradiated Foods</u>

Codex General Methods.

[1] *Standard for Foods for Special Dietary Use for Persons Intolerant to Gluten* (CXS 118-1979).

World Health Organization
REGIONAL OFFICE FOR Europe

Ending inappropriate promotion of commercially available complementary foods for infants and young children between 6 and 36 months in Europe

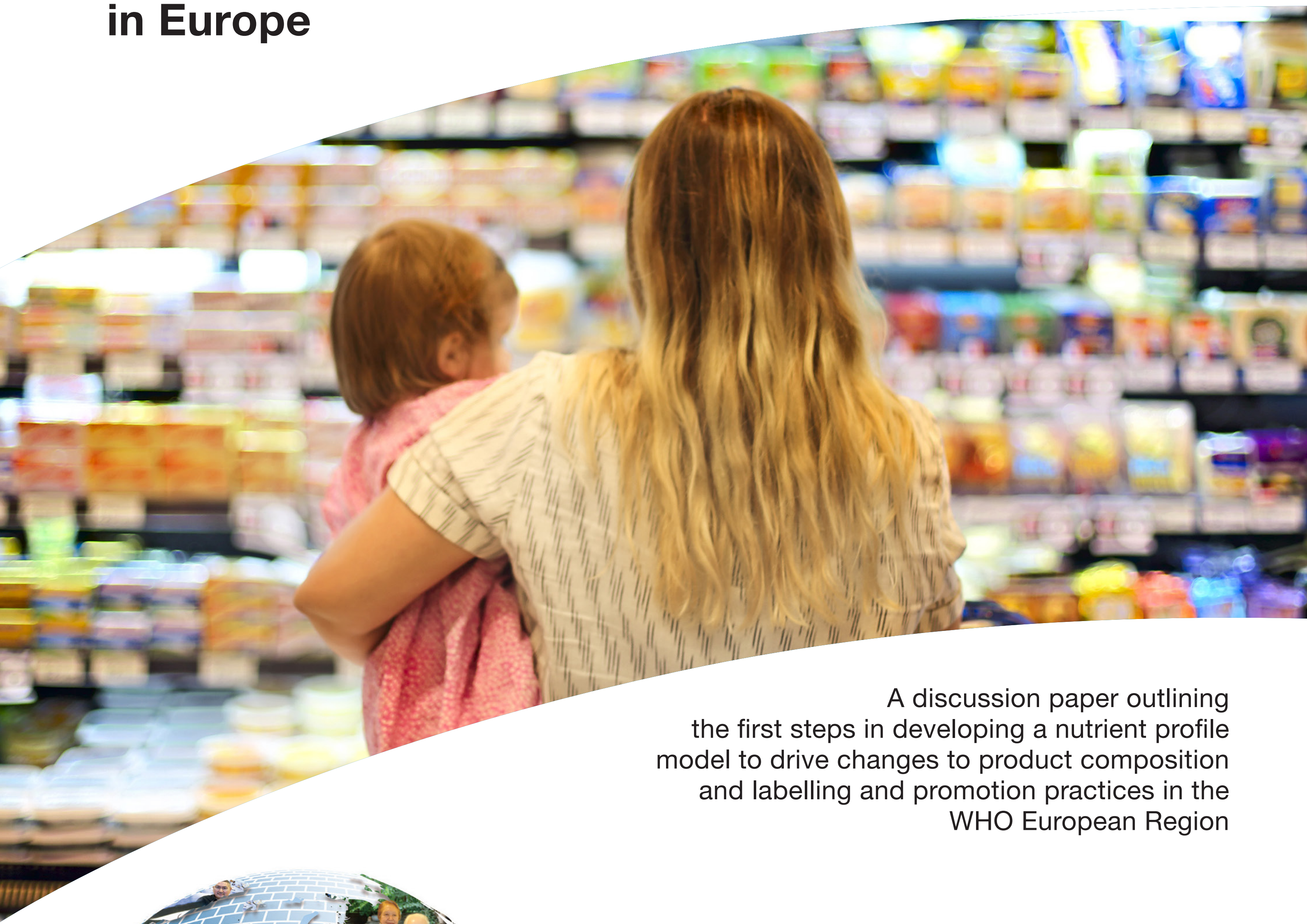

A discussion paper outlining the first steps in developing a nutrient profile model to drive changes to product composition and labelling and promotion practices in the WHO European Region

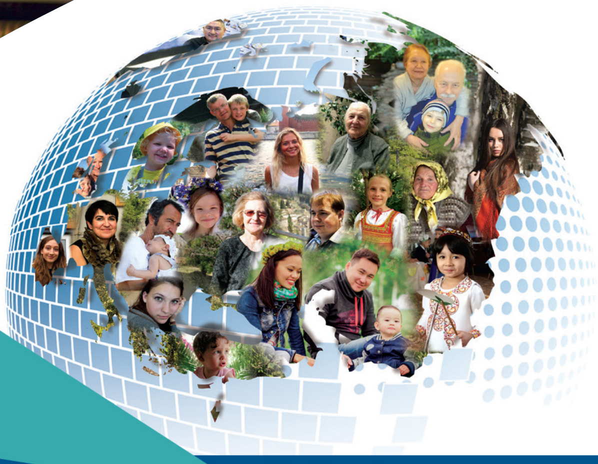

World Health Organization

REGIONAL OFFICE FOR Europe

Ending inappropriate promotion of commercially available complementary foods for infants and young children between 6 and 36 months in Europe

A discussion paper outlining the first steps in developing a nutrient profile model to drive changes to product composition and labelling and promotion practices in the WHO European Region

ABSTRACT

In 2016, the World Health Assembly approved WHO guidance on ending the inappropriate promotion of foods for infants and young children through resolution WHA69.9. The aims of the guidance are to protect breastfeeding, prevent obesity and chronic diseases, promote a healthy diet, and ensure caregivers receive clear and accurate information on infant and young child feeding. Resolution WHA69.9 requested WHO to provide technical support to Member States in implementing the guidance recommendations, including the development of nutrient profiling tools. This discussion paper outlines the first steps in developing a nutrient profile model for commercially-available complementary foods marketed as suitable for infants and young children (6–36 months).

Address requests about publications of the WHO Regional Office for Europe to:

Publications
WHO Regional Office for Europe
UN City, Marmorvej 51
DK-2100 Copenhagen Ø, Denmark

Alternatively, complete an online request form for documentation, health information, or for permission to quote or translate, on the Regional Office website (http://www.euro.who.int/pubrequest).

© World Health Organization 2019
All rights reserved. The Regional Office for Europe of the World Health Organization welcomes requests for permission to reproduce or translate its publications, in part or in full.

The designations employed and the presentation of the material in this publication do not imply the expression of any opinion whatsoever on the part of the World Health Organization concerning the legal status of any country, territory, city or area or of its authorities, or concerning the delimitation of its frontiers or boundaries. Dotted lines on maps represent approximate border lines for which there may not yet be full agreement.

The mention of specific companies or of certain manufacturers' products does not imply that they are endorsed or recommended by the World Health Organization in preference to others of a similar nature that are not mentioned. Errors and omissions excepted, the names of proprietary products are distinguished by initial capital letters.

All reasonable precautions have been taken by the World Health Organization to verify the information contained in this publication. However, the published material is being distributed without warranty of any kind, either express or implied. The responsibility for the interpretation and use of the material lies with the reader. In no event shall the World Health Organization be liable for damages arising from its use. The views expressed by authors, editors, or expert groups do not necessarily represent the decisions or the stated policy of the World Health Organization.

CONTENTS

Page

Acknowledgements ... vi

Acronyms ... vii

Executive summary ... viii

Aims and tasks ... viii

Document purpose and scope ... ix

Summary of the proposed provisions for foods and nutritional composition ... ix

Summary of proposed requirements on packaging, labelling and promotions of CACFs marketed for infants and young children up to 36 months ... x

Scope of application ... xi

Key findings of the supplementary materials: literature review, and validation and pilot testing of the NPM for infants and young children ... xi

Nutrient composition of products on the market in relation to existing regulations ... xii

Meeting proposed compositional thresholds ... xiii

PART 1 ... 1

Draft nutrient profile model and proposed requirements for labelling, marketing and promotion 1

1.1. Background ... 2

Aims and tasks ... 4

1.2. Summary of the NPM and proposed requirements on labelling, marketing and promotion of CACFs ... 5

Intended audience and purpose ... 5

Definitions and scope of application ... 5

Summary of the proposed provisions for foods and nutritional composition ... 8

Summary of proposed requirements on packaging, labelling and promotions of CACFs marketed for infants and young children up to 36 months ... 8

Recommendations to food producers ... 10

How to use the NPM and proposed requirements on labelling, marketing and promotion of CACFs ... 10

1.3. Summary of key steps for developing the draft NPM and proposed requirements on labelling, marketing and promotion of CACFs ... 24

Aligning the development process to the WHO advice for building an NPM ... 25

References ... 36

PART 2 39

Supplementary material 39

2.1. Literature review of complementary feeding practices and marketing of commercial products 40

Age of solid-food introduction 40

Development of taste preferences 42

Sweet and savoury flavours in commercial foods 44

The role of food texture 45

Puréed commercial foods and pouches 47

Nutritional quality of CACFs 48

Health implications of CACFs 50

Marketing: claims and aspirational statements 53

2.2. Currently unresolved issues and further considerations for strengthening the proposed NPM and labelling requirements 57

2.3. Validating the NPM for infants and young children using CACFs data from Denmark, Spain and the United Kingdom 60

Nutrient composition of CACFs: assessment of individual nutrients in relation to current European Commission regulations and proposed thresholds 60

Meeting European Commission regulations overall 96

Assessing the overall strictness of the compositional criteria of the NPM 96

2.4. Pilot testing the NPM with a further seven European countries 100

2.5. Supplementary tables 103

References 130

PART 3 134

Annexes 134

Annex 1 135

Annex 2 137

Annex 3 139

Annex 4 140

Annex 5 142

Annex 6 148

Annex 7 149

Annex 8 153

Annex 9 155

Annex 10 156

Acknowledgements

This document was prepared for WHO Regional Office for Europe by Dr Jayne Hutchinson, Dr Diane Threapleton and Professor Janet Cade (University of Leeds Nutrition Epidemiology Group, United Kingdom) and Mr Jo Jewell (WHO Regional Office for Europe). Significant contributions and input, notably in the conceptualization of the work, were provided by Dr João Breda (WHO Regional Office for Europe) and Ms Holly Rippin (University of Leeds, United Kingdom) during the pilot-testing phase. Further thanks are owed to Dr Helen Crawley (First Steps Nutrition Trust, United Kingdom), Dr Margherita Caroli (European Childhood Obesity Group, Italy), Dr Charlene Elliot (University of Calgary, Canada), Dr Ada Garcia (University of Glasgow, United Kingdom), Ms Nathali Hirsch (independent consultant, Denmark), Professor Mike Rayner (University of Oxford, United Kingdom) and Dr Antonina Starodubova (Federal Research Centre of Nutrition, Biotechnology and Food Safety, Russian Federation) for their inputs and kind review of an earlier draft. Thanks also to colleagues at Helen Keller International and to researchers who collected or collated the Danish, Spanish and United Kingdom data, and to teams in the countries that collected data for pilot testing (Estonia, Hungary, Italy, Malta, Norway, Portugal and Slovenia). These activities were partially funded through a grant of the Russian Government in the context of the WHO European Office for the Prevention and Control of Noncommunicable Diseases.

Acronyms

BLW	baby-led weaning
BMI	body mass index
CACF	commercially available complementary food
EFSA	European Food Safety Authority
ESPGHAN	European Society for Paediatric Gastroenterology Hepatology and Nutrition
EU	European Union
FFL	flavour–flavour learning
FNL	flavour–nutrient learning
IQR	interquartile range
NPM	nutrient profile model
SACN	(United Kingdom) Scientific Advisory Committee on Nutrition
SD	standard deviation

Executive summary

There is increasing recognition that some commercially available complementary foods (CACF) do not support public health dietary recommendations and do not adhere to WHO's recommended age of food introduction. Many CACFs are sweet, mask vegetable and other flavours with fruit and are puréed, which may negatively influence children's learning about food taste and texture. There are also concerns over the high sodium and added and total-sugar content of some CACFs, as well as their fatty acids profile, which may not be consistent with international and national dietary and nutrient guidelines. Regulations on product composition, packaging and promotions could be considered insufficient, as they permit some unsuitable CACFs to be marketed for infants and young children up to 36 months. Consequently, calls have been made by Member States at the World Health Assembly to end the inappropriate promotion of foods for infants and young children.

In 2016, the World Health Assembly approved WHO guidance on ending the inappropriate promotion of foods for infants and young children through resolution WHA69.9. The aims of the guidance are to protect breastfeeding, prevent obesity and chronic diseases, promote a healthy diet, and ensure caregivers receive clear and accurate information on feeding. These calls have been echoed at regional level in the WHO *European food and nutrition action plan 2015–2020*, through which Member States of the WHO European Region reaffirmed the need to promote appropriate complementary feeding practices, notably by establishing standards for the marketing of complementary foods.

Resolution WHA69.9 requested WHO to provide technical support to Member States in implementing the guidance recommendations (paragraph 7(1)), which call on Member States to consider the development of nutrient profiling tools to aid implementation. Governments require guidance on steering food manufacturers to improve the quality of their products and provide unambiguous information on packets that will neither mislead consumers nor undermine public health recommendations. As such, further technical support to Member States and tools from WHO are necessary to facilitate policy action by Member States.

Aims and tasks

Nutrient profiling is used to classify foods according to nutrient levels related to promoting health. This work aimed to take the first steps in developing a nutrient profile model (NPM) for CACFs marketed as suitable for infants and young children (6–36 months) as a basis for discussion and consultation with Member States in the WHO European Region. The draft model was developed by following recommended WHO steps, and was informed by data from several sources. It refers to existing European Commission directives and Codex standards, and reflects the approach used in the WHO Regional Office for Europe NPM for children over 36 months.

The supplementary material provides detailed analysis of CACFs on the market from 10 countries, including an estimate of the percentage of products that would meet the proposed NPM requirements before and after modelling steps for product reformulation. The supplementary material also presents a rapid literature review of evidence on factors identified as current issues of concern for complementary feeding and marketing of CACFs that informed the process of developing the NPM. The work also considers changes that may be required to labelling and promotion practices.

Document purpose and scope

Identifying products that can and cannot be promoted for infants and young children up to 36 months is a crucial first step towards implementing the WHO guidance in countries and will support the development of effective legal and policy measures to avoid inappropriate promotion. This document aims to support countries through the development of an NPM that is appropriate for foods marketed for infants and young children.

The draft model is primarily for consultation. Earlier versions of the model have been validated against data from Denmark, Spain and the United Kingdom and pilot-tested in a further seven countries. Details of the validation and pilot exercises are available in the supplementary material. The NPM was amended following validation and pilot testing. The NPM contained in the main body of the report reflects these changes. Ultimately, it is intended that governments will be able to adapt and use the model for the purposes of restricting the inappropriate promotion of foods for infants and young children in their own countries.

The draft NPM aims to categorize CACFs, identify products that may be considered suitable to be marketed for infants and young children up to 36 months, and ensure that permitted products are promoted appropriately. To achieve these aims, the model establishes compositional thresholds and provides guidance on product-labelling and promotions. The NPM pays particular attention to the marketing of CACFs high in saturated fats, trans fatty acids, free sugars and salt, as called for in the WHO guidance.

The key points from the NPM are summarized below, with clarification of the proposed requirements for labelling, marketing and promotion that will prevent inappropriate promotion of foods for infants and young children. Together, the nutrient thresholds, categorization of unsuitable foods, and requirements for labelling, marketing and promotion ensure that nutritional composition will improve, but also guarantee that fundamental overarching provisions relating to appropriate promotion apply to all CACFs.

Summary of the proposed provisions for foods and nutritional composition

- Confectionery and sweet snacks should not be marketed as suitable for infants and young children up to 36 months.
- Fruit drinks and juices and sweetened cows' milk/milk alternatives should not be marketed as suitable for infants and young children up to 36 months.
- Savoury snacks and finger foods with greater than 15% energy from total sugars should not be marketed as suitable for infants and young children up to 36 months.
- Added sugars and other sweetening agents (including all syrups, honey, fruit juice, fruit juice concentrates or non-sugar sweeteners such as saccharin, acesulfame, sucralose, aspartame and stevia) should not be used in CACFs for infants and young children up to 36 months. Fruit juice and fruit juice concentrate are considered added sugars and should not be used in CACFs (except for a small permitted amount of lemon or lime juice as a preservative).
- A threshold allowing only a limited amount (≤ 5% by weight) of processed or concentrated 100% fruit (whole fruit that is puréed or dried) to be used as ingredients (for instance, powder of dried apple and purée of dried strawberries) is proposed in certain categories such as meals.
- Thresholds for total sugar are also proposed, leading to a requirement to display the percentage of total energy from sugar on the front of pack. This is intended to assert

downward pressure on the use of processed or concentrated 100% fruit to impart sweet taste to the product.

- A minimum energy density threshold of 60 kcal/100 g is required for some soft–wet spoonable foods to ensure that complementary foods provide adequate nutrition for infants and young children between 6 and 12 months and are not made largely of water or very low-energy foods.
- The maximum permitted sodium content should be reduced for CACFs for infants and young children up to 36 months, limiting sodium content to 50 mg/100 kcal and 50 mg/100 g for all foods except cheese purées and meals (where cheese is listed in the front-of-pack product name and the protein content from dairy is 2.2 g/100 kcal), where the limit suggested is 100 mg/100 kcal and 100 mg/100 g of product.
- For CACFs that name a protein source in the product name on the front of pack, the minimum required proportion of fish, poultry, meat or other traditional source of protein should be 8% (of total product weight) and 3 g/100 kcal. For products where any source of protein is the first listed food in the product name, the minimum proportion is 10% and 4 g/100 kcal.
- Industrially produced trans fatty acids should not be included in CACFs. It is recommended that they be eliminated from the food supply. According to WHO guidance and national dietary recommendations from several European countries, CACFs should contain no industrially produced trans fatty acids and intake should be as low as possible among this age group.
- Total fat should not exceed 4.5 g/100 kcal except in certain types of products with higher protein content.

Summary of proposed requirements on packaging, labelling and promotions of CACFs marketed for infants and young children up to 36 months

- No CACFs should be marketed as suitable for infants less than 6 months of age.
- All products must state the suitable age of introduction.
- No products should include any image, text or other representation that might suggest use for infants under the age of 6 months (including references to milestones and stages).
- All CACF products must include a statement on the importance of continued breastfeeding for up to two years or beyond and the importance of not introducing complementary feeding before 6 months of age.
- No products should include any image (for example, idealizing images of babies or young animals), text or other representation that is likely to undermine or discourage breastfeeding, that makes a comparison to breast milk, or that suggests that the product is nearly equivalent or superior to breast milk.
- The packaging design, labelling and materials used for the promotion of complementary foods must be different from those used for breast-milk substitutes to avoid any cross-promotion. Products that function as breast-milk substitutes should not be promoted in any way, in line with the International Code of Marketing of Breast-milk Substitutes and subsequent World Health Assembly resolutions.
- Other products on the market targeted at older children over 36 months that are unsuitable for infants and young children up to 36 months (those that are not intended for infants and young children, do not comply with the NPM and/or do not meet other regulatory requirements for CACFs) should clearly state a minimum age of 36 months/3 years on packs (including sweet breakfast cereals, energy drinks and children’s snack foods).

- All CACFs containing fruit (fresh or processed in any way) should state the percentage of this product in the ingredient list.
- All CACFs should state the percentage of added water in the ingredients list.
- Food packaging with a spout should state clearly, “Infants and young children must not be allowed to suck directly from the pouch/pack/container”.
- If the total-sugar content exceeds specified limits, the front of pack must show the percentage of energy from total sugar. Limits for different foods are set at 30% energy for dry cereals and fruit/vegetable purées, 40% for dairy-based foods, 20% for vegetable purées with cereals or milk, and 15% for savoury and meal-type foods.
- Compositional (that is, nutritional) and health claims should not be permitted for CACFs, in line with CAC/GL 23-1997 Codex guidelines for the use of nutrition and health claims.
- No product should convey an endorsement or anything that may be construed as an endorsement by a professional or other body, unless this has been specifically approved by relevant national, regional or international regulatory authorities.
- Mandatory guidelines should be drafted to ensure product promotions and labelling claims are not misleading or confusing, provide appropriate instructions on preparation and use (that is, do not encourage sucking direct from the pouch), do not encourage (either implicitly or explicitly) early introduction, do not imply that commercial foods are nutritionally superior to home-prepared foods or otherwise undermine important public health recommendations for this vulnerable demographic.
- Front-of-pack product names should better reflect the ingredients in descending order of content to ensure they do not mislead parents and caregivers. They must state the name of the largest ingredient, when appropriate, as the first listed food in the front-of-pack product name and possibly with the amount expressed as a percentage of total weight or in grams or other measures (according to country customs) in the ingredient list. Note that fruit or vegetables are considered the largest ingredient if the sum of all fruits or vegetables is the largest ingredient; the front-of-pack name should therefore indicate that fruits or vegetables constitute the majority of the product.

Scope of application

Promotion practices are constantly evolving and changing in terms of how, where and by whom designated products are promoted, and to what extent families and other caregivers of infants and young children are exposed to such practices. The final goal is to end all forms of inappropriate promotion of identified products. Common types of promotion include: advertising activities and materials, including online promotions; non-advertising promotions in communities and health-care facilities; labelling and messaging on packing, such as health claims; and cross-promotion of products.

Key findings of the supplementary materials: literature review, and validation and pilot testing of the NPM for infants and young children

The literature review

The literature review identified a conflict between WHO recommendations to introduce complementary foods at 6 months and many existing commercial products marketed as suitable from 4+ months. Since CACFs for early introduction of solid foods are predominantly smooth and sweet food blends/purées and rarely include single food flavours or bitter vegetables, many CACFs may not be suited to meeting infants’ need for exposure to a variety of textures, single flavours, bitter flavours and other non-sweet foods. Processed CACFs sold in pouches with

spouts are also increasingly popular but have limited textures, high water content (meaning low nutrient/energy density) and high free-sugar content. Not all CACFs are nutritionally inadequate, but frequent selection of low-quality foods (those with low energy density or high sugar content) may not provide the appropriate supplementary nutrition required for healthy growth and development.

The review also found that guidelines and regulations applying to CACFs are inconsistent and do not necessarily reflect the modern market, where the range and form of products has proliferated greatly. Categorization of new types of products can be difficult and many CACFs have misleading names or include promotional statements that imply superiority of commercial products over home-prepared foods. Updated guidelines, regulations and legislation may be needed to ensure product promotions and labelling do not undermine important public health recommendations.

Nutrient composition of products on the market in relation to existing regulations

Analysis of packet-label information of CACFs for infants and young children up to 36 months on the market in 2016/2017 in the United Kingdom (n = 768), Denmark (n = 319) and Spain (n = 241) revealed that the vast majority of products comply with current regulatory requirements: only 4–8% of products examined in each country did not meet the existing 2006/125 EC regulations relating to salt, protein, fat and carbohydrate content, as far as could be determined from the available data.

Many CACFs nevertheless were high in total sugar. According to nutritional information available on packages, the mean percentage energy from total sugar in fruit purées was over 70% in each country. Many savoury-type meals sold in the United Kingdom and Denmark derived over 15% energy from total sugars, with fruit purée providing much of the sugar content even in ostensibly savoury products. Similar to fruit juices, these sugars can be considered free sugars, due to the high maceration and consequent release of sugars from the cell wall. Frequent intake of CACFs with a high free-sugar content may negatively affect oral health. It is reasonable to suggest that if eaten frequently or over prolonged periods, they may pose a threat to the very young as their first teeth erupt. Frequent exposure to sweet foods is also likely to influence taste preferences as children develop.

When examining the list of ingredients to determine presence of sugars added by the manufacturer, the types of product that contained added sugar and the type of sugars used varied across the three countries. About a quarter of the products on the United Kingdom (28%) and Danish (21%) markets listed one or more added sugars in their ingredients, compared to nearly half of Spanish products (44%). Where national guidelines exist for this age group, most recommend that free-sugar intake for infants should be as low as possible, indicating that any NPM developed should address the use of added sugars and sweetening agents as well as the use of fruit purée as an ingredient.

Purées of mainly fruits and vegetables often had relatively low energy density (lower than 50 kcal/100 g), but some Spanish vegetable purées had higher energy density due to the addition of olive oil. Some of the savoury-type purées also had relatively low energy density, with levels similar to the density of simple fruit or vegetable purées (around 50–70 kcal/100 g). Being less energy-dense than breast milk, these products may not provide sufficient energy for infants under 12 months if they are the main source of solid food. For this reason, the addition of water to many of these products may also be a concern.

Few CACFs exceeded the current European Commission thresholds for fat (roughly equivalent to 40% energy from total fat), but some dairy-based foods contained a high proportion of total energy from saturated fat. Current sodium product regulations were exceeded in only a very small number of products, but some foods across all categories from each country contained over 100 mg sodium/100 kcal, which indicates that sodium content could be reduced further through reformulation.

Meeting proposed compositional thresholds

An earlier version of the NPM was validated using data sets from three countries, before further pilot testing of the model was conducted using data sets from seven other countries.

Only about a third of products on the market in the United Kingdom, Denmark and Spain in 2016/2017 that could be analysed met all of the proposed compositional thresholds of the earlier version of the NPM. These related to increasing protein and energy density of purées and reducing sodium, total fat, total sugar and added sugar content. This low pass rate overall occurred despite large proportions of products meeting the individual requirements (for sodium or total fat, for example).

Modelling reformulation changes of 10% towards the proposed requirements did not substantially alter the percentage of products meeting all compositional thresholds for the six areas (only a further 6% in the United Kingdom, 9% in Denmark and 1% in Spain) when no restrictions to added sugars were modelled. Modelling restrictions of “no added sugar” alone (a theoretical removal of all added sugars), however, increased products meeting the six NPM requirements by 11% of products in the United Kingdom, 13% in Denmark and 37% in Spain. These results indicate that initial reformulation efforts should focus on “no added sugars” to provide the largest effect in meeting the proposed NPM thresholds. Modelling a 10% reformulation scenario plus the restriction of “no added sugars” meant that 49% of products in the United Kingdom, 58% in Denmark and 69% in Spain met all draft NPM thresholds.

A further pilot test was conducted in seven additional countries – Estonia, Hungary, Italy, Malta, Norway, Portugal and Slovenia – that provided nutrition and market data on approximately 100–400 products from their domestic markets. The data were cleaned and analysed, and the proportion of products passing each criterion of the proposed NPM, and the NPM overall, was calculated.

The percentage of products passing the earlier version of the NPM in its entirety ranged from 15% (Hungary) to 42% (Estonia). The criterion with the lowest pass rate in most countries was that concerning the protein content of meals. The protein minimum threshold for meals in this earlier version of the NPM was raised from the European Commission regulations, and the pass rate was low across all countries for this nutrient, particularly Malta (4%) and Hungary (6%). The percentage energy from total sugar in finger foods criterion also had a relatively low pass rate, particularly in Hungary and Italy (14%). Salt was generally less of an issue. Hungary also had a lower pass rate (42%) for “no added sugars/sweeteners” than the other countries. It is clear that added sugars are an issue in CACFs in all of the countries: if all products did not contain added sugars, the overall NPM pass rate in the 10 countries would range from 42% in the United Kingdom to 66% in Portugal.

The proposed NPM was updated following the pilot test and feedback from the countries involved, involving amendments to some of the thresholds (notably, protein was lowered to the

European Commission threshold), simplification of categories and improvements in practical application. The version of the NPM included in this discussion paper is the revised version.

PART 1

Draft nutrient profile model and proposed requirements for labelling, marketing and promotion

1.1. Background

When, how and what to feed infants, from their first foods to participating in family meals, are complex and emotive questions. Commercial baby foods have been available for over 100 years *(1)* and their use is pervasive in many developed countries *(2)*. Concerns have been raised over the content and marketing of commercially available complementary foods (CACFs), centring on the sweet-taste profile of many foods, the lack of diverse ingredients (such as bitter vegetables), the nutritional quality (CACFs are often lower in iron or higher in free sugars than homemade foods) and limited food textures. CACFs are also criticized for lacking the authentic taste, texture and appearance of simple homemade foods *(1)*, which may negatively influence the acceptance of regular foods later in childhood.

Evidence supporting the link between early life nutrition and adult health now forms a cornerstone of health promotion and public health programmes globally *(3)*. The variation in quality or quantity of nutrients consumed in early life is thought to be responsible for so-called programming for important risk factors for noncommunicable diseases in adulthood *(3)*. Dietary habits are formed at young ages and persist into later years. WHO recommends that infants should be breastfed exclusively for the first 6 months of life to achieve optimal growth, development and health. Thereafter, they should receive nutritionally adequate and safe complementary foods while breastfeeding continues up to 2 years of age or beyond.

The health consequences of different complementary feeding practices have been much debated in recent years, but evidence indicates that food and drinks high in fats, free sugars and salt are being marketed for consumption by young children, and greater protection against such marketing practices is important *(4)*. To date, however, there has been some uncertainty around the effects of certain foods or feeding practices, largely because of insufficient evidence.

In the WHO European Region, Codex guidelines and European Union (EU) directives ensure that CACFs are safe and adhere to specified minimum or maximum nutrient thresholds *(5,6)* (Annexes 1–4). The market has diversified and proliferated extensively since these guidelines and regulations were developed. Some studies argue that the so-called health halo surrounding CACFs is unwarranted *(7)* and the issue of CACF quality has been the subject of many recent studies, with concern that regulations are insufficient and patchy in the current market environment *(7–17)*. The high added sugar, free sugar or total-sugar content of CACFs, the texture and flavours used, the development of new snacking products, misleading product names, widespread use of claims and the early recommended age of introduction on the label (typically under 6 months) are some of the concerns that are not fully addressed in current guidelines.

An example of the level of concern about the composition and promotion of these products is the European Parliament's rejection of the European Commission's proposal to retain existing provisions for the sugar content of baby foods in their proposal for a revised EU regulation (EU No. 609/2013) (Annexes 5 and 6) *(18–20)*. Echoing these concerns, the World Health Assembly approved through resolution WHA69.9 (paragraph 7(1)) the WHO guidance on ending the inappropriate promotion of foods for infants and young children in 2016 (Annex 7) *(21)*, and WHO was requested to provide technical support to Member States in implementing the guidance recommendations.

The aims of the guidance are to protect breastfeeding, prevent obesity and chronic diseases, promote a healthy diet, and ensure that caregivers receive clear and accurate information on feeding. This call has been repeated at regional level in the WHO *European food and nutrition action plan 2015–2020 (22)*, through which Member States of the WHO European Region

reaffirmed the need to promote appropriate complementary feeding practices, notably by establishing standards for the marketing of complementary foods.

The WHO Secretariat established a scientific and technical advisory group on inappropriate promotion of foods for infants and young children in 2013 to inform development of the guidance. The group reviewed definitions of terms, current guidance on complementary feeding (globally and for individual countries), current marketing practices, current laws and regulations on marketing of complementary foods (globally and for individual countries), evidence on the health effects of CACFs, and evidence on the effects of marketing commercial complementary foods on infant and young child feeding. It produced two reports to the Secretariat, the first (in 2013) providing a definition of the term "inappropriate promotion", and the second (2015) containing draft guidance to help achieve the goal of ending inappropriate promotion of foods for infants and young children (Box 1).

Box 1. What is considered inappropriate promotion of foods for infants and young children?

Promotion of foods for infants and young children is considered inappropriate if it interferes with breastfeeding, contributes to obesity and noncommunicable diseases, creates a dependency on commercial products, or otherwise is misleading.

Recommended breastfeeding practices can be undermined by inappropriate promotion in various ways. This includes promotion of such products as suitable for infants under 6 months, as equivalent or superior to breast milk, or as a replacement for breast milk, or by using brands/labels/logos that are the same/similar to those used for breast-milk substitutes.

Promotion of products that contain high levels of sugar, salts or fats may contribute to childhood obesity and noncommunicable diseases; such promotion should therefore be considered inappropriate.

Promotion of foods not recommended in national food-based dietary guidelines is likewise inappropriate. Promotion is also inappropriate if the product fails to adhere to all applicable standards for safety and nutrient composition or discourages a diverse diet based on a wide variety of foods, including minimally processed fruits, vegetables and animal-source foods, or if it undermines the use of suitable home-prepared and/or local foods.

Promotion is inappropriate if it is misleading, confusing or could lead to inappropriate use through, for instance, health and nutrition claims. Promotional claims idealize the product, imply that it is better than family foods and mask the risks. Promotional claims put unprocessed family foods at a disadvantage. Nutrition and health claims shall not be permitted for foods for infants and young children.

There are hundreds of CACFs on the market labelled as suitable for infants and young children (many labels indicate products are suitable from 4 months of age), demonstrating that such products are widely purchased/consumed in many countries. Dietary surveys among this age group suggest that use of CACFs is common: in the 2011 United Kingdom diet and nutrition survey of infants and young children between 4 and 18 months, for example, 58% of those who had taken food other than milk had consumed CACFs *(23)*.

Given that many infants and young children are exposed to CACFs, it is important to understand and monitor the appropriateness of ingredients, nutrient contents and product types aimed at this young age group. Identifying products that can and cannot be promoted for infants and young

children up to 36 months is a crucial first step for countries to take in implementing the WHO guidance.

The final WHO guidance on ending the inappropriate promotion of foods for infants and young children recommended that nutrient profile models (NPMs) be developed and utilized to guide decisions about which foods are inappropriate for promotion, with a particular focus on avoiding free sugars and salt *(21)*. Nutrient profiling is the science of classifying or ranking foods according to their nutritional composition for reasons related to preventing disease and promoting health, and can be used, for instance, to generate criteria to classify foods according to nutrient levels. In addition to developing an NPM, this discussion paper also proposes requirements for labelling, marketing and promotion that are needed alongside the NPM to ensure that consumers are not misled and that appropriate infant feeding is protected.

The individual nutritional needs of infants and young children, which change rapidly as they develop, represent one of the main challenges in applying nutrient profiling to the nutritional content of CACFs. Needs vary depending on age and growth, and will be met in different ways depending on the amount and frequency of breast milk, formula or other foods and drinks consumed. An NPM aimed at CACFs can nevertheless provide guidance on foods likely to be of particular nutritional concern. An approach that incorporates guidance around clearer labelling, commercial messaging, packaging and/or age of suitability can supplement the traditional format of an NPM to promote selection of healthy and nutritious foods, address challenges faced by consumers in choosing the right combination of CACFs to complement breast milk or formula consumed (that is, nutrient-rich, diverse, increasingly textured, but not salt- or sugar-rich), and ensure marketing does not undermine breastfeeding or go against other nutritional recommendations or perceptions of home-prepared foods.

Aims and tasks

The WHO Regional Office for Europe has developed this discussion paper to support countries to create effective policy and legal measures to avoid inappropriate promotion. The document provides a tool in the form of a draft NPM along with a set of proposed requirements for labelling, marketing and promotion. The supplementary material describes the findings of the analyses of different types of products currently being marketed in 10 countries of the WHO European Region, their composition and promotional practices. It also explores the most relevant literature relating to current issues with complementary feeding practices to develop understanding of where changes might be most needed in the European Region.

The draft NPM aims to categorize CACFs, identify products that are suitable to be marketed for infants and young children up to 36 months, and ensure permitted products are promoted appropriately.

The draft model is primarily for consultation. Ultimately, governments will be able to adapt and use the model for the purposes of restricting the inappropriate promotion of foods for infants and young children in their country.

1.2. Summary of the NPM and proposed requirements on labelling, marketing and promotion of CACFs

Intended audience and purpose

The model aims to:

- define CACFs that are suitable to be marketed for infants and young children up to 36 months; and
- ensure that permitted products are labelled and promoted appropriately.

To achieve these aims, the model establishes compositional thresholds and provides additional guidance on product labelling and promotions. The NPM pays particular attention to the marketing of CACFs high in saturated fats, trans fatty acids, free sugars and salt.

The model and proposed requirements are designed for use by governments to restrict the inappropriate promotion of foods for infants and young children, consistent with the WHO guidance approved by the World Health Assembly through resolution WHA69.9 *(21)*. When determining whether a food product may or may not be marketed to children, a government (or food company) should take a series of steps, described below. It is understood that if CACFs do not comply with the relevant provisions, it is expected that they would not be promoted as being suitable for infants and young children up to 36 months.

Definitions and scope of application

CACFs are defined as all foods and beverage products that are specifically marketed as suitable for feeding infants and young children up to 36 months of age and that are complementary to breast milk or breast-milk substitutes. CACFs are considered to be marketed as being suitable for this age group if they:

- are labelled with the words “baby”, “infant,” “toddler” or “young child”;
- are recommended for introduction at an age of less than 3 years;
- have a label with an image of a child who appears to be younger than 3 years of age or who is feeding with a bottle; or
- are in any other way presented as being suitable for children under the age of 3 years.

This definition is consistent with World Health Assembly resolution WHA69.9 and the guidance on inappropriate promotion of foods for infants and young children, which it approved.

Marketing is defined as product promotion, distribution, selling, advertising, product public relations and information services.

Promotion is broadly interpreted to include the communication of messages that are designed to persuade or encourage the purchase or consumption of a product or raise awareness of a brand. Promotional messages may be communicated in a variety of settings, including in-store promotions and via traditional (such as television) and digital mass media. Marketing promotion also includes the packaging, branding and labelling of a product. In addition to promotional techniques aimed directly at consumers, measures to promote products to health workers or to consumers through other intermediaries are included. There does not have to be a reference to a brand name of a product for the activity to be considered as advertising or promotion. Cross-promotion (also called brand-crossover promotion or brand stretching) is an additional form of marketing promotion included in this definition of marketing in which customers of one product

or service are targeted with promotion of a related product. Promotional messages and cross-promotion that this NPM applies to are further defined in the WHO guidance on ending the inappropriate promotion of foods for infants and young children and its accompanying implementation manual *(21,24,25)*.

Some common types of promotion include those shown in Box 2.

Box 2. Common kinds of promotion

Advertising activities and materials, including online promotions (such as via Facebook, Twitter or other social media)
Examples include:
- media advertisements (such as TV, radio, online and print materials); and
- any audiovisual material meant to promote relevant products (such as TV/radio commercials, billboards, posters, newsletters, pamphlets, and promotion in books, magazines, journals, newspapers).

Non-advertising promotion activities
Examples include:
- promotion or sales inducement at the location/place where designated products are sold (such as special displays/offers/sales, discount coupons and rebates, loss-leaders and tie-in sales);
- promotion in communities and public places (such as banners, free product distribution/ company gifts and discount coupons); and
- promotion in health-care facilities and by health workers (such as donation or acceptance of company equipment/services/gifts/other incentives, use of health facilities for commercial events/contests/campaigns, and distribution of any gifts or coupons to parents/ caregivers/families)

Labelling, messaging and packaging
Examples include:
- health and structural claims, recommending or promoting bottle-feeding; and
- pictures, images, and wording suggesting appropriate use of the product for infants less than 6 months.

Cross-promotion of products
Examples include:
- cross-promotion through misleading labelling (e.g. using similar logos, colours and labelling formats on breast-milk substitutes and food products); and
- direct contact of company representatives with mothers and other caregivers via social media.

Breast-milk substitutes are not included in the NMP. WHO considers that breast-milk substitutes include any milks (or products that could be used to replace milk, such as fortified soy milk alternatives) in either liquid or powdered form that are specifically marketed for feeding of infants and young children up to the age of 3 years (including follow-up formula and growing-up milks). Other foods and beverages promoted as suitable for feeding a baby during the first 6 months of life when exclusive breastfeeding is recommended (including baby teas, juices and waters) are also considered to be breast-milk substitutes. WHO maintains that breast milk remains the most appropriate liquid part of a progressively diversified diet for the vast majority of children between 6 months and 2 years of age, once complementary feeding has begun. Acceptable milk sources exist for those children who, for various reasons, are not breastfed, or for whom breastfeeding will stop before the recommended duration of two years or beyond. The World Health Assembly stated in 1986 that “the practice being introduced in some

countries of providing infants with specially formulated milks (so-called follow-up milks) is not necessary". World Health Assembly resolution WHA 69.9 restated that provisions relating to the inappropriate promotion of breast-milk substitutes are contained in the International Code of Marketing of Breast-milk Substitutes *(26)* and subsequent relevant World Health Assembly Resolutions *(21,24,25).* The Code *(26)*:

> applies to the marketing, and practices related thereto, of the following products: breast-milk substitutes, including infant formula; other milk products, foods and beverages, including bottlefed complementary foods, when marketed or otherwise represented to be suitable, with or without modification, for use as a partial or total replacement of breast milk ...

Products that function as breast-milk substitutes should not be promoted. If follow-up formula or milks marketed for children over 1 year of age are marketed or otherwise represented to be suitable, with or without modification, for use as a partial or total replacement for breast milk, they are covered by the Code. Where follow-up formula is otherwise represented in a manner which results in the product being perceived or used as a partial or total replacement for breast milk, such a product falls within the scope of the Code *(26)*. Specialised milk products (sometimes called infant foods for special medical purposes) are also covered by the Code. For this reason, breast-milk substitutes fall outside the scope of this NPM.

The NPM does, however, cover cross-promotions for CACFs that may have the direct or indirect effect of promoting breast-milk substitutes and also includes provisions for statements about the benefits of breastfeeding on labels. A decision was taken to include a category for fruit drinks and juices and sweetened cow's milk/milk alternatives in the NPM, as they may fall out of the scope of policies and regulations applied to breast-milk substitutes.

Non-breast-milk substitute products for special dietary or medical purposes are not included in the NPM. These include vitamin and mineral food supplements and home-fortification products such as micronutrient powders and small-quantity lipid-based nutrient supplements *(21,25)*. Some foods for infants and young children are fortified with vitamins and minerals: these are included within the definition of CACFs and are therefore included in the NPM.

The NPM developed for this report is presented in two parts: first, food categories are defined (Table 1); and second, proposed nutrient composition thresholds are presented according to each food category, alongside labelling requirements and promotional restrictions (Table 2). Examples of compositional, health and marketing claims on product packaging marketing and promotional activities are shown in Table 3.

The key points from the NPM are summarized below, with clarification of the proposed requirements for labelling, marketing and promotion that will prevent inappropriate promotion of foods for infants and young children. Together, the nutrient thresholds, categorization of unsuitable foods, and requirements for labelling, marketing and promotion ensure that nutritional composition will improve, but also guarantee that fundamental overarching provisions relating to appropriate promotion apply to all CACFs.

Summary of the proposed provisions for foods and nutritional composition

- Confectionery and sweet snacks should not be marketed as suitable for infants and young children up to 36 months.
- Fruit drinks and juices and sweetened cows' milk/milk alternatives should not be marketed as suitable for infants and young children up to 36 months.
- Savoury snacks and finger foods with greater than 15% energy from total sugars should not be marketed as suitable for infants and young children up to 36 months.
- Added sugars and other sweetening agents (including all syrups, honey, fruit juice, fruit juice concentrates or non-sugar sweeteners such as saccharin, acesulfame, sucralose, aspartame and stevia) should not be used in CACFs for infants and young children up to 36 months. Fruit juice and fruit juice concentrate are considered added sugars and should not be used in CACFs (except for a small permitted amount of lemon or lime juice as a preservative).
- A threshold allowing only a limited amount (≤ 5% by weight) of processed or concentrated 100% fruit (whole fruit that is puréed or dried) to be used as ingredients (for instance, powder of dried apple and purée of dried strawberries) is proposed in certain categories such as meals.
- Thresholds for total sugar are also proposed, leading to a requirement to display the percentage of total energy from sugar on the front of pack. This is intended to assert downward pressure on the use of processed or concentrated 100% fruit to impart sweet taste to the product.
- A minimum energy density threshold of 60 kcal/100 g is required for some soft–wet spoonable foods to ensure that complementary foods provide adequate nutrition for infants and young children between 6 and 12 months and are not made largely of water or very low-energy foods.
- The maximum permitted sodium content should be reduced for CACFs for infants and young children up to 36 months, limiting sodium content to 50 mg/100 kcal and 50 mg/100 g for all foods except cheese purées and meals (where cheese is listed in the front-of-pack product name and the protein content from dairy is 2.2 g/100 kcal), where the limit suggested is 100 mg/100 kcal and 100 mg/100 g of product.
- For CACFs that name a protein source in the product name on the front of pack, the minimum required proportion of fish, poultry, meat or other traditional source of protein should be 8% (of total product weight) and 3 g/100 kcal. For products where any source of protein is the first listed food in the product name, the minimum proportion is 10% and 4 g/100 kcal.
- Industrially produced trans fatty acids should not be included in CACFs. It is recommended that they be eliminated from the food supply. According to WHO guidance and national dietary recommendations from several European countries, CACFs should contain no industrially produced trans fatty acids and intake should be as low as possible among this age group.
- Total fat should not exceed 4.5 g/100 kcal except in certain types of products with higher protein content.

Summary of proposed requirements on packaging, labelling and promotions of CACFs marketed for infants and young children up to 36 months

- No CACFs should be marketed as suitable for infants less than 6 months of age.

- All products must state the suitable age of introduction.
- No products should include any image, text or other representation that might suggest use for infants under the age of 6 months (including references to milestones and stages).
- All CACF products must include a statement on the importance of continued breastfeeding for up to two years or beyond and the importance of not introducing complementary feeding before 6 months of age.
- No products should include any image (for example, idealizing images of babies or young animals), text or other representation that is likely to undermine or discourage breastfeeding, that makes a comparison to breast milk, or that suggests that the product is nearly equivalent or superior to breast milk.
- The packaging design, labelling and materials used for the promotion of complementary foods must be different from those used for breast milk substitutes to avoid any cross-promotion. Products that function as breast milk substitutes should not be promoted in any way, in line with the International Code of Marketing of Breast-milk Substitutes and subsequent World Health Assembly resolutions.
- Other products on the market targeted at older children over 36 months that are unsuitable for infants and young children up to 36 months (those that are not intended for infants and young children, do not comply with the NPM and/or do not meet other regulatory requirements for CACFs) should clearly state a minimum age of 36 months/3 years on packs (including sweet breakfast cereals, energy drinks and children’s snack foods).
- All CACFs containing fruit (fresh or processed in any way) should state the percentage of this product in the ingredient list.
- All CACFs should state the percentage of added water in the ingredients list.
- Food packaging with a spout should state clearly, “Infants and young children must not be allowed to suck directly from the pouch/pack/container”.
- If the total-sugar content exceeds specified limits, the front of pack must show the percentage of energy from total sugar. Limits for different foods are set at 30% energy for dry cereals and fruit/vegetable purées, 40% for dairy-based foods, 20% for vegetable purées with cereals or milk, and 15% for savoury and meal-type foods.
- Compositional (that is, nutritional) and health claims should not be permitted for CACFs, in line with CAC/GL 23-1997 Codex guidelines for the use of nutrition and health claims.
- No product should convey an endorsement or anything that may be construed as an endorsement by a professional or other body, unless this has been specifically approved by relevant national, regional or international regulatory authorities.
- Mandatory guidelines should be drafted to ensure product promotions and labelling claims are not misleading or confusing, provide appropriate instructions on preparation and use (that is, do not encourage sucking direct from the pouch), do not encourage (either implicitly or explicitly) early introduction, do not imply that commercial foods are nutritionally superior to home-prepared foods or otherwise undermine important public health recommendations for this vulnerable demographic. Examples are listed in Table 3.
- Front-of-pack product names should better reflect the ingredients in descending order of content to ensure they do not mislead parents and caregivers. They must state the name of the largest ingredient, when appropriate, as the first listed food in the front-of-pack product name and possibly with the amount expressed as a percentage of total weight or in grams or other measures (according to country customs) in the ingredient list. Note that fruit or vegetables are considered the largest ingredient if the sum of all fruits or vegetables is the largest ingredient; the front-of-pack name should therefore indicate that

fruits or vegetables constitute the majority of the product. Further naming details and examples are listed in Box 3 and Table 4.

Recommendations to food producers

Food producers should:

- produce more vegetable and savoury foods than fruit-based and sweet foods;
- refrain from masking the flavour of (bitter) vegetables with sweet purées or fruit flavours;
- produce more single-flavour foods for infants and young children between 6 and 12 months;
- produce fewer highly blended foods and more foods with texture for infants and young children between 6 and 12 months;
- reduce the total- and free-sugar contents of foods;
- use no added sugars or sweetening agents;
- phase out pouches with spouts and instead sell foods in clear rip-top packets or jars so that foods are visible and must be served using a spoon;
- avoid the unnecessary addition of water to, for instance, make foods that can be served through a spout;
- sell soft–wet spoonable foods of higher energy density and in smaller portion sizes for infants below 12 months old; and
- avoid producing treats or desserts, and make it clear on packaging that these should not be provided daily or as part of the usual diet.

How to use the NPM and proposed requirements on labelling, marketing and promotion of CACFs

Users should take the following steps.

1. Identify which food category the product falls under. In some cases, this will be clear according to the food category name (for example, fruit purées, meat-based meals) in Table 2. In other cases, it may be necessary to reference the definition and examples and/or the nutritional declaration and ingredients list (Table 1).
2. Once the appropriate food category has been identified, the ingredients list and the nutritional content of the food product must be crosschecked against the thresholds in the "Proposed nutrient composition standards to be met" columns of Table 2.
3. A food product must meet each of the compositional requirements for that product category if it is to be marketed as suitable for children under 36 months of age. No CACFs should be marketed as suitable for children under 6 months.
4. Using the nutrition declaration and ingredients list, the product labelling requirements and promotional restrictions for that food product category should be considered next. For example, once the food category has been identified and the compositional requirements have been met, users should assess whether additional front-of-pack labelling is required, whether the age restrictions as stated on the package have been met and whether the product name is appropriate or needs to change to comply with the NPM. The NPM states that no nutrition and health claims are permitted on CACFs.

Table 1. Proposed food categories for all foods marketed as suitable for infants and young children 6–36 months of age[a]

	Food category	Definition and examples
**1**	**Dry, powdered and instant cereal/starchy food**	
1.1	Dry or instant cereals/starch	Dry rice, cereal, pulverized rusks or starchy root (at least 25% cereal and/or starch root content) with or without naturally sweet foods (such as dry fruit and powdered fruit juice), milk powder or whey powder<br>Products to be made up with liquid<br>Includes dry instant-type porridges and dry breakfast cereals (such as puffed rice or cereal hoops), if marketed as suitable for infants and young children<br>Excludes wet ready-to-eat cereals
**2**	**Soft–wet spoonable, ready-to-eat foods, typically smooth or semi-pureed packaged in jars or pouches and can be spoon-fed**	
2.1	Dairy-based desserts and cereal products	Foods with dairy as the largest main ingredient by weight (i.e. greater than the sum of total fruit or total grain ingredients). This may include yogurt, fromage frais, custards, porridge or rice pudding, made with or without other naturally sweet foods such as fresh fruit, fruit juice or dried fruit. Does not contain meat or fish.
2.2	Fruit purée with or without addition of vegetables, cereals or milk	Largest ingredient single or mixed fruit. May contain vegetables, cereals and dairy<br>Includes any spoonable fruit or fruit-and-vegetable purée, high-fruit breakfast foods (such as fruit-based breakfast rice/ porridge) and desserts (such as apple crumble or fruit-based baby rice). May include some products labelled as "smoothies", without the addition of juice or water.
2.3	Vegetable only purée	≥ 95% single or mixed vegetables or legumes and water combined<br>Excludes products containing any fruit, or >5% cereals or other ingredients<br>May include some products labelled as "smoothies", without the addition of fruit or vegetable juice.
2.4	Puréed vegetables and cereals	Puréed vegetables/legumes, where largest ingredient by weight is vegetables, legumes, cereals or pseudocereals , with > 5% cooked weight in cereal (e.g. pasta, rice, barley), or a pseudocereal (such as quinoa, chia, buckwheat)<br>Includes savoury-type meals with cereals (such as pasta with tomato and courgette) or pseudocereal (such as butternut squash, carrot and quinoa). Does not contain meat or fish.<br>Includes vegetable-based foods containing cheese, where cheese is not mentioned in the product name
2.5	Puréed meal with cheese (but not meat or fish) mentioned in the name	A puréed meal containing cheese, vegetables, starchy carbohydrates, where cheese is mentioned in the name (such as "Cheesy pasta with tomato and vegetables" or "Cauliflower cheese" or "Macaroni cheese"). Does not contain meat or fish.
2.6	Puréed meal with meat or fish mentioned as first food in product name	A puréed meal containing meat or fish in addition to vegetables, and other starchy carbohydrates. May contain other ingredients<br>Meat or fish is mentioned as first food in product name (such as "Tasty fish pie" or "Salmon and pea risotto" or "Hearty beef hotpot" or "Chicken and potato pie")
2.7	Puréed meals with meat or fish (but not named as the first food in product name)	A puréed meal containing meat or fish, vegetables, and starchy carbohydrates, where the fish/meat protein source is not listed as first food in product name (such as "Hearty shepherd's pie", "Cottage pie" or "Carrot, potato and lamb hotpot"). May contain other ingredients
2.8	Purées with only meat, fish or cheese in name	Puréed meat, fish, or cheese where they are the only food listed in product name and constitutes the single largest

	Food category	Definition and examples
	of product	ingredient (except water). These are not intended to be complete meals and should be served with vegetables and starchy carbohydrates.
**3**	**Meals with chunky pieces, often sold in trays or pots for older infants and young children**	
3.1	Meat, fish or cheese-based meal with chunky pieces	A non-puréed soft meal containing chunky pieces of meat or fish in addition to vegetables, and starchy carbohydrates. May contain other ingredients such as cheese.<br>Fish or meat is mentioned as first food in product name (such as “Tasty fish pie” or “Salmon and pea risotto” or “Hearty beef hotpot” or “Chicken and potato pie”)
3.2	Vegetable-based meal with chunky pieces	A non-puréed soft meal containing chunky pieces of vegetables, and other starchy carbohydrates. May contain other ingredients such as beans and pulses as sources of protein and iron. May contain meat or fish or cheese not mentioned in the product name
**4**	**Dry finger foods and snacks**	
4.1	Confectionery, sweet spreads and fruit chews	Confectionery includes: chocolate and other products containing cocoa; white chocolate; jelly sweets and boiled sweets; chewing gum and bubble gum; caramels; liquorice sweets; marzipan; sweetened or “yogurt”-coated fruit etc.<br>Sweet spreads: spreadable chocolate and any other sweet sandwich/toast topping such as jam, marmalade or honey and sweet nut spreads etc.<br>Fruit chews include any dried and processed fruit products such as fruit gums, bars or fruit strips/leathers/roll-ups (i.e. a dense chewy food made from fruit juice or pulped and dehydrated/dried fruit), including fruit pieces coated in sugar or oils/fats (such as banana chips, sweetened cranberries or yogurt raisins)
4.2	Fruit (fresh or dry whole fruit or pieces)	Includes fresh whole or peeled fruit (such as apple) and dried fruit (such as dry slices of plain apple, freeze-dried strawberries, raisins, dry apricots, prunes)<br>Excludes fruit pieces coated in sugar or oils/fats (such as banana chips, sweetened cranberries or yogurt raisins) and dried and further processed fruit products (see category 4.1.)
4.3	Other snacks and finger foods	Includes foods such as savoury biscuits and pretzels, baked chips/crisps (such as potato, grain or other starchy food etc.), rice cakes coated in powdered fruit or vegetables, cereal bars<br>Also includes any rusks/teething biscuits, sweet baked, fried, dried or dehydrated food intended to be eaten between meals, sweet pastries; croissants; cookies/biscuits; sponge cakes; wafers; fruit pies; sweet buns; chocolate-covered biscuits; cake mixes and batters; cereal or energy bars (i.e. cereal/ granola or muesli bars); and crisps/puff products made from fruit, vegetables or starchy foods (which may be coated in fat/oil)
**5**	**Juices and other drinks,** products are typically packaged in bottles, cans or tetrapaks and can be poured or served to infants as a drink in cups with/without spouts.	
5.1	Single or mixed fruit juices, vegetable juices, or other non-formula drinks	Any drinkable product containing crushed, blended, pulped or puréed fruit or vegetable, fruit or vegetable juice and/or water, with or without added sugar or sweetening agents, including 100% juices, reconstituted juice from concentrate, smoothies with added juice or water. Also includes drinks ready made from cordials, energy drinks, ices, cola, lemonade, orangeade, other soft drinks, and mineral and/or flavoured waters (including aerated) with added sugars or sweetener.<br>Excludes smoothies/purées without the addition of juice or water (see category 2.1)<br>Excludes all products that function as breast-milk substitutes (see exclusions to the model)

	Food category	Definition and examples
		Excludes unsweetened cow's milk and unsweetened milk alternatives (such as soya, oat, almond) marketed for consumption by general population
5.2	Cow's milk and milk alternatives with added sugar or sweetening agent	Whole cow's milk and milk alternatives including soya, oat or almond milk with added sugar or sweetening agent<br>Excludes other products that function as breast-milk substitutes (see exclusions to the model)

[a] Exclusions to the model.

- Products not specifically marketed for children younger than 3 years of age.
- Vitamin and mineral food supplements, whether to be consumed as tablets/drops or added to foods at home (such as home fortification products such as micronutrient powders, lipid nutrient powders).
- Products that function as breast-milk substitutes; these should not be promoted at all. These include any milks (or products that could be used to replace milk, such as fortified soya milk alternatives), in either liquid or powdered form, that are specifically marketed for feeding infants and young children up to the age of 3 years. This includes milk or milk-like formulations commonly marketed for infants from 6 months of age and prepared in accordance with relevant international or national standards. The upper age indication on the product label varies country to country but is usually between 12 and 36 months. Any milk product that is marketed or represented as suitable as a partial or total replacement of the breast milk part of the young child's diet is a breast-milk substitute and therefore falls under the scope of the International Code. This product always replaces breast milk, as breastfeeding is recommended to continue for 2 years or beyond. Follow-up formula should therefore not be promoted. These provisions also apply to growing-up milk (also known as growing-up formula, toddler milk or formulated milk), which is targeted at infants and young children from 1 year old (sometimes younger) to 3 years old. Often, the product name is similar to a company's formula products, with a figure "3" added on. Where growing-up milks are marketed as suitable for feeding young children up to the age of 36 months, they fall under the International Code definition of "breast-milk substitute" read together with WHA resolution 58.32 from 2005, which recommends breastfeeding should continue for up to 2 years or beyond.
- Products whose labels state that they are intended only for pregnant women, mothers or children older than 3 years.

Table 2. Draft nutrient profile model for CACFs for infants and young children up to 36 months old

Food category		Proposed nutrient composition standards to be met	Proposed labelling requirements and promotional restrictions
		**Age under 6 months**<br>No complementary foods should be marketed as suitable for infants < 6 months	**Age under 6 months**<br>No labelling of products as suitable for ages < 6 months<br>No pictures of babies < 6 months
		**Age 6–36 months**<br>Marketing of products allowed if they conform to the below rules for composition.	**Age 6–36 months**<br>Permitted products must conform to the below general rules regarding labelling and promotions:<br>No cross-promotions are permitted between products that function as breast-milk substitute and CACFs marketed as suitable for infants and young children > 6 months<br>All products must include a statement on the importance of continued breastfeeding for up to two years or beyond and the importance of not introducing complementary feeding before 6 months of age<br>No products should include any image, text or other representation that is likely to undermine or discourage breastfeeding, that makes a comparison to breast-milk, or that suggests that the product is nearly equivalent or superior to breast-milk;<br>All products must state the suitable age of introduction<br>No products should include any image, text or other representation that might suggest use for infants under the age of 6 months (including references to milestones and stages);<br>No product should convey an endorsement or anything that may be construed as an endorsement by a professional or other body, unless this has been specifically approved by relevant national, regional or international regulatory authorities.<br>Further specific provisions by category follow.
**1**	**Dry, powdered and instant cereal/starchy food**		
1.1	Dry or instant cereals/starch	**No added sugar or sweetening agent**<br>≤ 10% by weight **dried or powdered fruit**<br>**Sodium** <50mg/100kcal<br>**No industrially produced trans fatty acids**<br>≤ 4.5 g/ 100 kcals **total fats**<br>< 5.5 g/100 kcal **total protein**	**Total sugar:** front-of-pack flag if ≥ 30% total energy<br>**Age restriction**: ≥ 6 months only<br>**No claims**<br>**Product name/ingredient clarity**<br>**No added sodium in liquid used to reconstitute product**<br>**Ingredients list should state the amount of dried and powdered fruit (%)**

Food category		Proposed nutrient composition standards to be met	Proposed labelling requirements and promotional restrictions
**2**	**Soft–wet spoonable, ready-to-eat foods**		
2.1	Dairy: ready to eat cereals or desserts	**No added sugar or sweetening agent**<br>≤ 5% by weight **fruit puree** with a maximum of 2% from pureed dried fruit)<br>**Energy density** ≥ 60 kcal/100 g<br>**Sodium** < 50 mg/100 kcal and <50mg/100g<br>**No industrially produced trans fatty acids**<br>≤ 4.5g/100 kcal **total fat**<br>≥ 2.2 g **dairy protein**/100kcal (if dairy product is first or only ingredient in name)	**Total sugar:** front-of-pack flag if ≥ 40% total energy<br>**Age restriction**: ≥ 6 m only<br>**No claims**<br>**Product name/ingredient clarity**<br>**Statement about no sucking from spouts if in pouch**<br>**Ingredient list should state the amount of added water (%), total fruit (%), total cereal (%) by weight of total product**
2.2	Fruit purée with or without addition of vegetables, cereals or milk	**No added sugar or sweetening agent**<br>**Energy density** ≥ 60 kcal/100 g<br>**Sodium** < 50mg/100 kcal and < 50 mg/100 g<br>**No industrially produced trans fatty acids**<br>≤ 4.5 g/100 kcal **total fat**	**Total sugar:** front-of-pack flag if ≥ 30% total energy<br>**Age restriction**: ≥ 6 m and < 12 m only<br>**No claims**<br>**Product name/ingredient clarity**<br>**Statement about no sucking from spouts if in pouch**<br>**Ingredient list should state the amount of added water (%), total fruit (%), total cereal (%) by weight of total product**
2.3	Vegetable only purée	**No added sugar or sweetening agent**<br>**No added fruit/ fruit purée**<br>**Added water** < 25% by weight<br>**Sodium** < 50 mg/100 kcal and < 50 mg/100 g<br>**No industrially produced trans fatty acids**<br>≤ 4.5g/100 kcal **total fat**	**Total sugar:** front-of-pack flag if ≥ 30% total energy<br>**Age restriction**: ≥ 6 m and < 12 m only<br>**No claims**<br>**Product name/ingredient clarity**<br>**Statement about no sucking from spouts if in pouch**<br>**Ingredient list should state the amount of added water (%)**
2.4	Puréed vegetables and cereals	**No added sugar or sweetening agent**<br>**No added fruit/fruit purée**<br>**Energy density** ≥ 60 kcal/100 g<br>**Sodium** < 50 mg/100 kcal and < 50 mg/100 g<br>≤ 4.5 g/100 kcal **total fat**<br>**No industrially produced trans fatty acids**	**Total sugar:** front-of-pack flag if ≥ 20% total energy<br>**Age restriction**: ≥ 6 m and < 12 m only<br>**No claims**<br>**Product name/ingredient clarity**<br>**Statement about no sucking from spouts if in pouch**<br>**Ingredient list should state the amount of added water (%), total fruit (%), total cereal (%) by weight of total product**
2.5	Puréed meal with cheese (but not meat or fish) mentioned in the name	**No added sugar or sweetening agent**<br>≤ 5% by weight **fruit puree** with a maximum of 2% from pureed dried fruit<br>**Energy density** ≥ 60 kcal/100 g<br>**Sodium** < 100 mg/100 kcal and 100mg/100g	**Total sugar:** front-of-pack flag if ≥ 15% total energy<br>**Age restriction**: ≥ 6 m and < 12 m only<br>**No claims**<br>**Product name/ingredient clarity**<br>**Statement about no sucking from spouts if in pouch**

Food category		Proposed nutrient composition standards to be met	Proposed labelling requirements and promotional restrictions
		**No industrially produced trans fatty acids**<br>≥ 3g/100 kcal **total protein**<br>≥ 2.2g/100 kcal **protein from dairy**<br>≤ 6g/100 kcal **total fat if cheese is mentioned first**	**Ingredient list should state the amount of added water (%), total fruit (%), total cereal (%) by weight of total product**
2.6	Puréed meal with meat or fish mentioned as first food in product name	**No added sugar or sweetening agent**<br>≤ 5% by weight **fruit puree** with a maximum of 2% from pureed dried fruit<br>**Energy density** ≥ 60kcal/100 g<br>**Total protein** ≥ 4 g/100 kcal from the named sources (of which ≥ 2.2g/100 kcal **protein from dairy if cheese mentioned in front-of-pack name)**<br>**Protein named as the first food(s)** in the product name must be ≥ 10% by weight of the total product<br>Each **named protein** not less than 25% by weight of total named protein<br>**Sodium** < 50 mg/100 kcal and <50mg/100g (or < 100 mg/100 kcal and <100mg/100g if **cheese is listed in front-of-pack name**)<br>**No industrially produced trans fatty acids**<br>≤ 6g/100 kcal **total fat**	**Total sugar:** front-of-pack flag if ≥ 15% total energy<br>**Age restriction**: ≥ 6 m and < 12 m only<br>**No claims**<br>**Product name/ingredient clarity**<br>**Statement about no sucking from spouts if in pouch**<br>**Ingredient list should state the amount of added water (%), total fruit (%), total cereal (%) by weight of total product**
2.7	Puréed meals with meat or fish (but not named as the first food in product name)	**No added sugar or sweetening agent**<br>≤ 5% by weight **fruit puree** with a maximum of 2% from pureed dried fruit<br>**Energy density** ≥ 60 kcal/100 g<br>**Total protein** ≥ 3g/100 kcal from all sources (of which ≥ 2.2 g/100 kcal **protein from dairy if cheese mentioned in front-of-pack name)**<br>**Protein** source mentioned in the product name must be ≥ 8% by weight of the total product<br>Each **named protein** not less than 25% by weight of total named protein<br>**Sodium** < 50mg/100 kcal and <50mg/100g (or < 100 mg/100 kcal and <100mg/100g if **cheese is mentioned in front-of-pack name)**<br>**No industrially produced trans fatty acids**<br>≤ 4.5 g/100 kcal **total fat**	**Total sugar:** front-of-pack flag if ≥ 15% total energy<br>**Age restriction**: ≥ 6 m and < 12 m only<br>**No claims**<br>**Product name/ingredient clarity**<br>**Statement about no sucking from spouts if in pouch**<br>**Ingredient list should state the amount of added water (%), total fruit (%), total cereal (%) by weight of total product**

Food category		Proposed nutrient composition standards to be met	Proposed labelling requirements and promotional restrictions
2.8	Purées with only meat, fish or cheese in name of product	**No added sugar or sweetening agent**<br>≤ 5% by weight **fruit puree** with a maximum of 2% from pureed dried fruit<br>**Sodium** < 50 mg/100 kcal and <50mg/100g (or < 100 mg/100 kcal and <100mg/100g if cheese is mentioned in front-of-pack name)<br>**No industrially produced trans fatty acids**<br>≥ 30% **named protein** by weight of total product<br>≤ 6 g/100 kcal **total fat**<br>≥ 7 g/100 kcal **total protein** from the named sources (of which ≥ 2.2g/100 kcal protein from dairy if **cheese mentioned in front-of-pack name**)	**Total sugar:** front-of-pack flag if ≥ 15% total energy<br>**Age restriction**: ≥ 6 m and < 12 m only<br>**No claims**<br>**Product name/ingredient clarity**<br>**Statement about no sucking from spouts if in pouch**<br>**Ingredient list should state the amount of added water (%), total fruit (%), total cereal (%) by weight of total product**<br>**Should state that these products are not intended as a complete meal and should be served with vegetables and starchy carbohydrates**
**3**	**Meals with chunky pieces**		
3.1	Meat, fish or cheese-based meal with chunky pieces	**No added sugar or sweetening agent**<br>≤ 5% by weight **fruit puree** with a maximum of 2% from pureed dried fruit<br>**Sodium** < 50 mg/100 kcal and <50mg/100g (or < 100 mg/100 kcal and <100mg/100g if **cheese is listed in front-of-pack name**)<br>**Total protein** ≥ 3g/10 kcal from all protein sources, or ≥ 4g/10 kcal if protein source is named as first food (of which ≥ 2.2g/100 kcal protein from dairy if **cheese mentioned in front-of-pack name**)<br>Each **named protein** not less than 25% by weight of total named protein<br>**Protein** source mentioned in the product name must be ≥ 8% by weight of the total product, or ≥ 10% If protein named as the first food(s) in front-of-pack name<br>**No industrially produced trans fatty acids**<br>≤ 4.5 g/100 kcal **total fat**, or ≤ 6 g/100 kcal if protein source is named as first food	**Total sugar:** front-of-pack flag if ≥ 15% total energy<br>**Age restriction**: ≥ 6 m only<br>**No claims**<br>**Product name/ingredient clarity**<br>**Ingredient list should state the amount of added water (%), total fruit (%), total cereal (%) by weight of total product**
3.2	Vegetable-based meal with chunky pieces	**No added sugar or sweetening agent**<br>≤ 5% by weight **fruit puree** with a maximum of 2% from pureed dried fruit<br>**Sodium** < 50 mg/100 kcal and <50mg/100g (or < 100 mg/100 kcal and <100mg/100g if **cheese is listed in front-**	**Total sugar:** front-of-pack flag if ≥ 15% total energy<br>**Age restriction**: ≥ 6 m only<br>**No claims**<br>**Product name/ingredient clarity**<br>**Ingredient list should state the amount of added water (%), total fruit (%),**

Food category		Proposed nutrient composition standards to be met	Proposed labelling requirements and promotional restrictions
		**of-pack name**)<br>≥ 3 g/100 kcal **total protein**<br>**No industrially produced trans fatty acids**<br>≤ 4.5 g/100 kcal **total fat**	**total cereal (%) by weight of total product**
**4**	**Dry finger foods and snacks**		
4.1	Sweet confectionery, sweet spreads and fruit chews	**Should not be marketed** as suitable for infants and young children ≤ 36 months	***'Not suitable for infants and young children under 36 months'*** should be added to foods aimed at older children
4.2	Fruit (fresh or dry whole fruit or pieces)	**No added sugar or sweetening agent**<br>**No added fats**<br>**Sodium** < 50 mg/100 kcal and <50mg/100g<br>**No industrially produced trans fatty acids**<br>≤ 4.5 g/100 kcal **total fat**<br>≤50 kcal per portion or serve	**Total sugar:** front-of-pack flag if ≥ 30% total energy<br>**Age restriction**: ≥ 6 m only<br>**No claims**<br>**Product name/ingredient clarity**
4.3	Other snacks and finger foods	**No added sugar or sweetening agent**<br>**Total sugar** < 15% of total energy (3.75 g/100 kcal) otherwise considered a sweet snack that should not be marketed (see 4.1)<br>**Sodium** < 50 mg/100 kcal and <50mg/100g<br>**No industrially produced trans fatty acids**<br>≤ 4.5 g/100 kcal **total fat**<br>≤50 kcal per portion or serve	**Age restriction**: ≥ 8m only<br>**No claims**<br>**Product name/ingredient clarity**<br>If ≥ 15% of total energy (3.75 g/100 kcals) from **total sugars, *"Not suitable for infants and young children under 36 months"*** should be added to foods, including those aimed at older children or adults<br>**Ingredient list should state the amount of total fruit (%) by weight of total product**
**5**	**Juices and other drinks**		
5.1	Single or mixed fruit juices, vegetable juices, or other non-formula drinks	**Should not be marketed** as suitable for infants and young children < 36 months	**"Not suitable for infants and young children under 36 months"** should be added to foods aimed at older children or adults
5.2	Cow's milk and milk alternatives, with added sugar or sweetening agent	**Should not be marketed** as suitable for infants and young children < 36 months	**"Not suitable for infants and young children under 36 months"** should be added to foods aimed at older children or adults

Added sugars and sweetening agents which should not be included in complementary foods for infants are: (i) all mono- and disaccharides (including sugars derived from fruits, sugarcane, palms or root vegetables); (ii) all syrups, nectars and honey (including molasses/agave/maple/blossom nectar/malted barley syrup/brown rice syrup etc.); (iii) fruit juices or concentrated/powdered fruit juice, excluding lemon or lime juice; (iv) non-sugar sweeteners (such as saccharin, acesulfame, aspartame, sucralose or stevia).
Note that unsweetened whole fruits, are permitted. Blended, pulped, puréed or powdered 100% fruits (including dried fruit which has been puréed) are only permitted to be added as an ingredient in certain categories in limited amounts, as they are high in free sugars.
Total sugar: front-of-pack flag/label showing the percentage energy from total sugar is required if total-sugar content is ≥ the threshold for that product category in the NPM (15% total energy = 3.75 g/100 kcal; 20% total energy = 5 g/100 kcal; 30% total energy = 7.5 g/100 kcal; 40% total energy = 10 g/100 kcal).

Age restriction: all packaging and related marketing or promotional materials (promotional communications/ websites etc.) must clearly state that products are not suitable for babies < 6 months of age; puréed, ready-to-eat products must indicate that these are only suitable for infants > 6 and < 12 months of age because infants and young children should be encouraged to develop the ability to eat food with greater texture.
No claims (compositional, health or marketing) are permitted on packs or related marketing materials (promotional communications/websites etc.). This is in line with the Codex guidelines for the use of nutrition and health claims CAC/GL 23-1997. Refer to Table 3 for examples of claims.
Product name/ingredient clarity: the front-of-pack product name and legal product name must clearly represent the main ingredients and be listed in an appropriate order (refer to Box 1 and Table 4 for detail and examples) and the ingredient list must clearly indicate the proportion (%) of: (i) the largest single ingredient (even if this is water/stock); (ii) the amount of added water/stock (except where used for rehydration of legumes/grains etc.); (iii) the total or individual proportion of fresh or dried fruit; and (iv) the amount of fish, poultry, meat or other traditional source of protein.
Statement to discourage sucking from spouts: ready-to-eat puréed foods sold in packs with a spout must include a clear statement to discourage caregivers from allowing infants and young children to suck the food directly via the spout, such as: “Infants and young children must not be allowed to suck directly from the pouch/container”.
Retained cooking water or other added water should be minimal; this restriction does not apply to water necessary for rehydration of dried foods (such as legumes or grains).

Table 3. Examples of compositional, health and marketing claims on product packaging, marketing and promotional materials

	Examples of claims that would not be permitted	Permissible statements
**Composition and nutrition claims** include statements like the following examples	No added sugar, contains only naturally occurring sugars and salt, no added salt, contributes one of your five-a-day [fruit/vegetables], contains three types of vegetables, no added preservatives, no added colouring agents, no added seasoning, organic food, no added condiments, natural, fresh, contains vegetables, no allergens, no food additives, non-GMO food, no maltodextrin or modified starch, wholegrain, no added artificial flavour, contains the perfect balance of vitamins and minerals to help your body thrive, contains calcium, contains iron, contains vitamin C, contains a host of nutrients, contains dietary fibre, contains multiple vitamins, contains vitamin E, contains multiple minerals, contains vitamin A/β-carotene, contains vitamin B1, contains ω-3, low sodium, contains zinc, contains probiotics or prebiotics, contains protein or amino acids, contains vitamin B2, contains phospholipid, contains iodine, contains phosphorus, contains vitamin D, contains DHA, contains carbohydrate, contains magnesium, contains selenium, contains arachidonic acid, unique blend of nutrients	Statements relating to **common allergens** (such as: gluten-free or contains gluten; dairy/lactose-free or contains dairy/lactose; nut-free or contains nuts)<br>Statements relating to **religious or cultural food requirements** (such as: meat-free, or vegetarian, or contains meat; Kosher; Halal)<br>**Descriptive words** may be used within the ingredient list (such as organic carrots and wholegrain wheat flour)
**Health claims** include statements like the following examples	Nutritionally balanced, healthy, provides good nutrition to children, improves appetite, suitable for picky eaters, supports healthy growth, improves growth, good for digestion and absorption, supports learning to chew, supports learning to hold, combats constipation, good for bones and teeth, good for enteric flora, good for the brain, good for the eyes, supports vision and skin health, good for defecation, good for thyroxine synthesis, good for red blood cell synthesis and preventing iron-deficiency anaemia, good for metabolism, good for collagen synthesis, contributes to normal cognitive development, needed for the normal growth and development of bone, key minerals and vitamins that participate in the good functioning of baby's immune system, breakfast is one of the most important meals of the day, goodness of cereals, infant cereal is the ideal foundation to a healthy and balanced diet, perfectly balanced for growing babies, draws inspiration from the Mediterranean approach to health and well-being, extra goodness with wholegrain oats<br>The Department of Health and the World Health Organization recommend exclusive breastfeeding for the first six months. However, if you choose to wean earlier, our ingredients are suitable from 4 months	
**Marketing claims** include statements like the following examples	**Taste and quality:** delight for tiny taste buds; tasty; yummy; delicious; in my home the whole family loves them; picked at the peak of ripeness; truly tasty; bursting with goodness and flavour; my flavours are a new journey for tiny taste buds; exotic dishes are full of variety and flavour; our delicious new range of jars; individually steam cooked; we use over 27 different fruits and vegetables<br>**Texture:** smooth; easy-to-swallow texture and a simple flavour that is great for helping your little one as they start to explore solid foods; I'm textured; not lumpy and my yummy crispy bits will encourage your baby to begin to chew; ideally suited to promote exposure to textures; no bits/chunks; wider spout; perfectly smooth texture has been specially developed as an ideal first weaning food<br>**Convenience/lifestyle:** convenient; great for a busy and active life; ideal for breakfast or meals on the	

go; simply to top up between meals; great way to make fruit fun; closest thing to homemade with all of the goodness and none of the guilt; inspired by my favourite home-cooked recipes; encourages self-feeding

Conveying ideals on optimum feeding: making the right feeding choices for you and your baby; helps to build confidence and enjoyment with food; we've been pioneering research into infant and toddler nutrition for over 50 years to help you give your baby the best start in life; carefully prepared by our baby-food experts; we only use specially selected ingredients; grown by farmers we know and trust; we select the finest; nothing unnecessary; no junk; nothing nasty; setting standards; real fruit/vegetables; perfect for small hands; perfect; ideal; optimum; perfect way to start introducing your baby to solid food

Others: the government advises that you don't need to wean your little one until they are 6 months old. Every baby is different!; committed to giving 10% of profits to help fund food education charities; quality approved by Mumsnet Mums

Box 3. Further details relating to product-naming issues

Front-of-pack product names for soft–wet spoonable ready-made foods must better reflect the ingredients in descending order of content to not be misleading, and must state the name of the largest ingredient, when appropriate, as the first listed food. Exemptions for this rule apply when the largest ingredient is implied in the name, such as milk in porridge or potato in cottage pie. Note that fruit or vegetables are considered the largest ingredient if the sum of all fruits or vegetables is the largest ingredient; the front-of-pack name must therefore indicate that fruits or vegetables constitute the majority of the product.

Note that all ingredients do not need to be listed in name. Examples are as follows and in Table 4:

a) a product currently named “Spinach and sweet potato” that includes apple as the largest ingredient, and greater sweet potato than spinach, must be named “Apple, sweet potato and spinach”;
b) a product currently named “Chicken dinner” that includes 35% carrot, 30% parsnip, 20% potato and 15% chicken must be named to indicate that vegetables are the largest ingredient, such as “Root vegetable and chicken dinner” or “Carrot and potato mash with chicken”;
   - products currently named as porridge, baby rice or dairy (for example, yogurt, fromage frais and custard) but that are composed mainly of fruit/vegetables must indicate that fruit/vegetables (singly or together) are the largest ingredient by placing fruit/vegetables first in the name **and** using the terms “with” or “and” porridge/rice/yogurt/custard: products predominantly composed of fruit currently labelled as “Blueberry porridge” or “Baby rice with mangoes” should be named, for example, “Apple and mango with baby rice” or “Fruity mix with porridge”; products predominately composed of fruit currently named “Banana yogurt/custard” instead should be named, for example, “Bananas and yogurt/custard” or “Apple and strawberry with yogurt/custard”; a product with the following ingredients – yogurt 35%, apple 30%, pear 25%, blueberries 5% and raspberries 5% – must indicate that fruits form the basis of the food (for example, be named “Fruit and berries with yogurt”, and not “Berry yogurt”); and
c) a product with the following ingredients – cooked rice 30%, carrot 27%, pumpkin 20%, kale 15% and chicken 8% – that has less than 10% in weight from chicken must indicate that vegetables form the basis of the food (for example, “Vegetables and risotto with chicken” and not “Chicken risotto with kale”).

Table 4. United Kingdom commercial infant food product-naming issues and proposed improved names

Brand/producer	Target age	Front-of-pack product name	Ingredients (largest in bold)	Main name issue(s)	Proposed front-of-pack name
Cow & Gate	From 7 months	**Apple and blueberry yogurt**	**Apple (59%)**, yogurt (contains **milk**) (20%), blueberry (9%), banana (8%), rice flour, Aronia juice from concentrate,[a] vitamin C	Name implies yogurt is main ingredient but fruit content = 76%.	Apple with yogurt and blueberry<br>OR<br>Apple and blueberry with yogurt
Cow & Gate	From 7 months	**Butternut squash, chicken & pasta**	Vegetables (46%) (**tomato**, butternut squash (10%), onion, parsnip), pasta (contains gluten) (20%)<br>(durum **wheat** semolina, **egg** white), water, chicken (9.5%), wheat starch (gluten free), corn starch, basil, oregano, rapeseed oil	Largest vegetable not named<br>Chicken placed before pasta in name	Tomato pasta with butternut squash and chicken<br>OR<br>Vegetable pasta with chicken
Ella's Kitchen	4+ months	**Peppers, sweet potatoes + apples**<br>**[purée]**	**Organic apples (78%),** organic red peppers (11%), organic sweet potatoes (11%), organic lemon juice concentrate (a dash)	Misleading order of foods in name	Apple with red peppers and sweet potatoes<br>[purée]
Ella's Kitchen	4+ months	**Peaches, pears + baby rice**<br>**[baby rice]**	**Organic pears (59%)**, organic peaches (38%), organic brown rice (3%), organic lemon juice (a dash)	Misleading order of foods in name<br>Only 3% rice and named as baby rice	Pears, peaches + rice<br>[fruit purée with rice]
Ella's Kitchen	6+ months	**Berry yogurt**<br>**[yogurt]**	**Organic Greek-style yogurt (milk) (34%)**, organic apples (31%), organic bananas (22%), organic blackcurrants (7%), organic blueberries (5%), organic rice starch (1%), organic lemon juice concentrate (a dash)	Name and label implies yogurt is main ingredient but fruit content = 65%<br>Main ingredient (apple) is not named on front of pack	Apple with yogurt and berries<br>[fruit puree with yogurt]<br>OR<br>Mixed fruit with yogurt and berries<br>[fruit puree with yogurt]
Asda Little Angels	7+ months	**Organic chicken casserole**	Tomatoes from concentrate, carrots (20%), potatoes (18%), peas(10%), onions (9.0%), chicken (8.0%), sweet potatoes, water, rosemary, thyme,	Name implies chicken forms a large part of the dish, but vegetables are the main ingredient	Vegetable, potato and chicken casserole
Heinz	4+ months	**Strawberry, raspberry & banana purée**	Fruit (100%, **concentrated apple purée (79%**), strawberries (8%), banana (8%), raspberries (5%), concentrated lemon	Main ingredient (apple) is not named on front of pack	Concentrated apple purée with banana and raspberry

Brand/producer	Target age	Front-of-pack product name	Ingredients (largest in bold)	Main name issue(s)	Proposed front-of-pack name
			juice), vitamin C		
Piccolo	6+ months	**Cherry and yogurt with wholegrain oats**	**Apples (43%)**, whole milk yogurt (milk) (30%), cherries (24%), wholemeal oat flavour (gluten) (3%), vanilla powder	Main ingredient (apple) is not named on front of pack	Fruity yogurt with wholegrain oats<br>OR<br>Apple and yogurt with cherries and wholegrain oats

[a] Concentrated sweet fruit products are not recommended as sweetening agents within the new NPM for infants and young children < 36 months.

1.3. Summary of key steps for developing the draft NPM and proposed requirements on labelling, marketing and promotion of CACFs

The draft NPM was developed by following recommended WHO steps, using the advice reported from the 2010 WHO nutrient profiling technical meeting *(27)*. This advice prompted the documentation of each consideration and issue surrounding complementary feeding and forms part of the rationale for the draft NPM.

The NPM was informed by data from several sources and the collated information was discussed among the main authors. Through an iterative process, decisions were made about suggested product categories, nutrient levels and recommended marketing restrictions. The NPM development process involved the following stages:

1. collecting examples of how CACFs for infants and young children aged up to 36 months are categorized in research literature, surveys, regulations and recommendations, and retail websites;
2. making reference to the International Code of Marketing of Breast-milk Substitutes, existing European Commission directives and Codex standards for CACFs and relevant WHO guidance (Annexes 1–4, 7 and 8);
3. making reference to the existing WHO Regional Office for Europe NPM for children over 36 months *(28)* (Annex 9);
4. undertaking a literature search of the issues related to complementary feeding and marketing of CACFs, and preparing a review to cover the following areas of concern:
    - age of solid food introduction and so-called target age of CACFs;
    - development of taste preferences and sweet and savoury flavours in CACFs;
    - the role of food texture, puréed CACFs and pouches;
    - nutritional quality of CACFs;
    - health implications of CACFs in relation to breastfeeding and milk intake, overweight and obesity, and tooth decay;
    - CACF marketing and packaging claims; and
5. detailed analysis of back-of-packet information from food labels, manufacturers’ and retailers’ websites from CACFs marketed for infants and young children up to 36 months in Denmark, Spain and the United Kingdom (see supplementary material for more information).

Descriptive analysis of current products informed the categorization of CACFs and the content for the draft NPM in terms of nutritional and marketing requirements. After establishing these NPM categories and requirements, they were applied to the product data sets to validate the NPM and identify what proportion of current foods meet (or do not meet) the proposed nutrient content thresholds, where possible, including estimates of the percentage meeting these thresholds after modelling product reformulation.

Once the first draft of the NPM had been prepared, it was shared with Member States of the WHO European Action Network on Reducing Marketing Pressures on Children for consultation and pilot testing.[1] Data from a further seven countries were analysed to assess the suitability of categories and the strictness of the model (that is, proportion of products that meet all

[1] The WHO Regional Office for Europe circulated the draft NPM to members of the WHO European Action Network on Reducing Food Marketing Pressures on Children following a meeting in Vienna, Austria, in May 2018. WHO received data sets, validated against the NPM, from Estonia, Hungary, Italy, Norway, Malta, Portugal and Slovenia.

requirements and "pass" the model), and to inform final amends. The draft model that appears in this document is the revised version.

Aligning the development process to the WHO advice for building an NPM

The WHO main areas of advice for building an NPM are shown below in the subheadings and highlighted in bold in the text, with considerations and suggestions for each point.

Define the purpose of the model and guidance

Identifying products that can and cannot be promoted for infants and young children up to 36 months is a crucial first step towards implementing the WHO guidance in countries and will support the development of effective legal and policy measures to avoid inappropriate promotion.

This involves defining suitable food categories and nutrient levels for CACFs for infants and young children aged up to 36 months, with guidance to avoid the inappropriate promotion of products for infants and young children. WHO guidance on ending inappropriate promotion of foods for infants and young children, approved by the World Health Assembly in 2016 *(25)*, states that particular attention should be given to marketing of foods high in saturated fats, trans fatty acids, free sugars and salt.

The WHO advice for building an NPM states that defining the purpose includes agreeing on whether the model should classify foods by a single nutrient or several nutrients. It is appropriate to classify food categories by several nutrients, as there is precedence in European Commission directives and Codex standards that apply nutrient-level provisions and recommendations for various macronutrients in CACFs that differ by product type. These regulations and standards specify levels for carbohydrates, total fats, lipids, protein, sodium, calcium, total sugars, added sucrose, fructose, glucose, glucose syrup or honey *(5,6)*. Chapter 1.2, describing the draft NPM, details CACF categories used and provides examples and exclusions.

The European Commission regulations also stipulate maximum limits for added vitamins, minerals and trace elements. No proposed changes to these have been made in the NPM (except for sodium) and existing limits for other micronutrients have not been included in the NPM. The usefulness of including content thresholds in the NPM for these nutrients should be further considered; setting iron and zinc minimum and maximum content thresholds could, for instance, be included in the NPM for CACFs that have a protein source named in the front-of-pack product name (poultry, fish, meat or other traditional source of protein). Requiring the back-of-pack label of all products to state iron, zinc and some other micronutrient content may help to increase the likelihood that complementary foods contribute to meeting recommendations that cannot fully be obtained from breast milk after 6 months *(29)*. As the Pan American Health Organization/WHO guiding principles for complementary feeding of the breastfed child *(30)* state:

> At 9–11 months of age, for example, it has been reported that the proportion of the Recommended Nutrient Intake that needs to be supplied by complementary foods is 97% for iron, 86% for zinc, 81% for phosphorus, 76% for magnesium, 73% for sodium and 72% for calcium.

Setting a minimum iron content threshold could, however, result in increased micronutrient fortification of CACFs when manufacturers do not wish to increase the content of the protein

source for cost or other reasons, or where a specific food cannot supply the required micronutrient level in a certain recipe. Also, the bioavailability of micronutrients in foods, micronutrient content and bioavailability in breast milk, volume of breast milk or formula consumed, and individual requirements will differ, meaning the exact micronutrient requirements for CACFs would be difficult to determine.

Define the target population group

The target population group for the NPM is infants and young children up to 36 months. This age group has been selected because existing WHO and Codex guidelines on formulated complementary foods for older infants and young children define young children as those up to 3 years. In addition, WHO recommends breastfeeding for two years or beyond: protection of continued breastfeeding beyond 2 years of age against inappropriate promotion is therefore essential (Annex 7).

The current WHO Regional Office for Europe NPM used for restricting marketing of products to children relates to those over 36 months (Annex 9), leaving a gap for infants and younger children *(28)*. Complementary feeding is needed when breast milk alone is no longer sufficient to meet the needs of growing infants; the target age range is generally taken to be 6–24 months, even though it is recommended that breastfeeding continues to age 2 and beyond *(30)*. Many products are currently marketed as suitable from 4 months of age, despite WHO guidelines that foods should not be marketed for infants less than 6 months *(21)*.

The age at which solid foods are introduced varies across countries (this is discussed in the supplementary material), in part driven by the persistent gap between the recommendation for exclusive breastfeeding for six months and the reality that this is often not achieved, with solid foods being introduced closer to 4 or 5 months of age in many countries *(23,31,32)*.

Nevertheless, in line with the WHO recommendations for exclusive breastfeeding to 6 months, the draft NPM for infants and young children states that no complementary foods can be recommended for infants below the age of 6 months. The introduction of potentially allergenic foods before 6 months is discussed further in the supplementary material. There has been debate about early (before 6 months) introduction of food and so-called sensitive periods in terms of exposure to different food tastes and textures that may influence later acceptance of foods, but evidence to support this is weak.

The draft NPM for infants and young children does not differentiate CACFs by age group, as the European Commission directive and Codex standards apply to all ages between 6 and 36 months. Instead, recommendations are based on nutrient intake per 100 g or 100 kcal of product, which avoids the need to specify intake per day for different age groups *(5,6)*.

Many CACFs specify a minimum age range for more textured meals or snack foods (9+ or 12+ months, for example). There is a case for also specifying a maximum age range for soft–wet puréed foods that are higher in free sugars than whole foods and which can provide rapid energy intake. Once teeth emerge and infants develop fine and gross motor skills, they no longer require puréed foods and should progress to consuming age-appropriate regular or family-style foods. The NPM therefore specifies that puréed and semi-puréed foods carry a maximum age label of 12 months.

The draft NPM for infants and young children does not distinguish between breastfed and non-breastfed infants, but there is a good case for considering this in greater detail in the future, as a high proportion of infants are not exclusively breastfed to 6 months. Among Italian women

between 2008 and 2011, for example, the prevalence of any/exclusive breastfeeding was reported as 92%/57% at discharge, 72%/49% at 3 months and 58%/6% at 6 months *(33)*. In the United Kingdom in 2010, exclusive breastfeeding at 4 months was 12%, and at 6 months just over one third of mothers were still breastfeeding; only 1% of infants were exclusively breastfed up to 6 months *(34)*. Breastfeeding rates in Germany tend to decline in the first two months and by 6 months, about 50% of infants still receive breast milk *(35)*.

Some complementary feeding recommendations, such as those on daily energy intake, are based on breastfed infants *(30)*, but they may not be appropriate for formula-fed infants. Preparing separate recommendations for breastfed and formula-fed infants is challenging, in that some babies receive both, with varying intakes. It is also unclear whether it is feasible to design an NPM that effectively distinguishes or discriminates between products based on whether they are intended for breastfed or non-breastfed infants.

Does the model need to define foods with a favourable profile, an unfavourable profile, or both?

The draft NPM stipulates both unfavourable and favourable CACF nutrient profiles. The Regional Office NPM for children over 36 months only defines unfavourable nutrient values, such as a maximum total fat g/100 g of the listed food *(28)*, but minimum requirements in the draft NPM for infants and young children are given for protein and energy density.

Some current CACFs contain low proportions of traditional sources of protein, such as meat and fish (around 8–10%), even when meat or fish features in the product name; CACFs sold in the United Kingdom, for instance, were found to be less protein-dense and have less iron and zinc than equivalent homemade foods *(1)*. The meat (and therefore protein) content of baby foods will differ across Europe according to cultural feeding norms or traditional recipes. In Italy, for example, the pilot testing revealed that many puréed meat products for infants contain 30% cooked meat by product weight; these products are not meant to be complete meals, however, and should be served with vegetables and other starchy carbohydrates. Protein intake is discussed further in the supplementary material.

Since meat, fish and other traditional sources of protein can add other important nutrients to the diet, any future recommendations based on concerns of excess intake of protein may be more suitably directed towards regulating protein in formula rather than in complementary foods *(36–38)*. Further research on the effects of protein in CACFs on the risk of future obesity may be needed.

The earlier version of the NPM proposed a modest increase from the current European Commission directive in the minimum content of any meat, poultry, fish, offal or other traditional source of protein listed in the product name; this was intended to increase the proportion of energy from protein and other beneficial nutrients such as iron. Ultimately, following pilot testing, it was decided to retain the original European Commission directive thresholds but highlight protein and iron content as an important consideration for future work.

CACFs should aim to add more nutrients and energy to the diet than breast milk *(1)*. A minimum energy density requirement is therefore likely needed for puréed foods, which are commonly given as first foods and often have low energy densities. As observed in the supplementary materials, a substantial proportion of purées in the United Kingdom and Denmark currently have energy densities below 60 kcal/100 g. The *Baby foods in the UK* review reports that the water content of many commercial baby foods in the United Kingdom is greater than that likely to be present in homemade foods *(1)*. This means that the energy density of the CACF is likely to be

lower than homemade foods *(39)* and is often lower than the energy density previously recommended for complementary feeding (at least 0.8 kcal/g) *(29)* and of breast milk (0.69 kcal/g) *(1)*.

To increase the energy density of soft–wet spoonable foods for infants, manufacturers should be encouraged to reduce and declare the water content of products. Setting a minimum energy density of 60 kcal/100 g for many purées will also encourage manufacturers to add less water for cooking and blending and will therefore ensure higher quality and better value products. The restrictions on adding sugars will ensure that this increase in energy density is not achieved through an increase in sugar content. A labelling requirement has also been added to the NPM to specify that CACFs must list the proportion of added water to products.

Minimum energy density requirements for simple vegetable purées may not be appropriate, as more than half of these products in the United Kingdom and Danish markets currently do not achieve this. It is unclear from packet information if the low energy density is due to intrinsic or added water, but many vegetables are naturally low in energy. To maintain variety in terms of simple vegetable tastes available on the market, a minimum energy requirement for vegetable purées has not been specified (category 2.3 in Table 1 and 2 of Chapter 1.2). Instead, a maximum added water content of 25% for simple vegetable purées (after allowing for any water required for cooking or rehydration of dried grains or legumes) is defined.

Conversely, maximum energy density or portion-size thresholds may be needed for some high-energy CACFs that can easily be overconsumed by older infants (savoury snacks or energy-dense tray meals). Also, despite the lower energy density of purées, a product that appears to be a single-serve portion can exceed estimated energy requirements at meals due to its large jar/package size *(1)*. Given that the NPM requires purées not to be marketed for infants and young children over 12 months, a review of portion sizes is required and recommendations for maximum portions for these products should be made.

Despite the need to provide adequate energy, CACFs should not provide excess nutrients of public health concern, such as free sugars. CACFs marketed for early introduction of solid foods (labelled as 4+ or 6+ months) are predominantly sweet and puréed, consisting mainly of fruit purées or fruit purée mixed with vegetables or cereals, but sweet flavours and ingredients are also common in foods for infants and young children up to 36 months more generally, even ostensibly savoury foods.

The addition of fruit purée, fruit juice or other added sugars/sweetening agents means that the total- and free-sugar values can be very high. WHO recommends limiting intake of free sugars to less than 10% (ideally 5%) of total energy intake. The American Heart Association recently stated that added sugars (defined as sugars used as ingredients) should be avoided under 2 years of age *(40)*, and a recent detailed review of issues surrounding CACFs in the United Kingdom also recommended that diet in the first year of life should be free of added sugars *(1)*.

WHO defines free sugars as “monosaccharides and disaccharides added to foods and beverages by the manufacturer, cook or consumer, and sugars naturally present in honey, syrups, fruit juices and fruit juice concentrates” *(41)*. The United Kingdom Scientific Advisory Committee on Nutrition (SACN) has expanded on the WHO definition of free sugars to stipulate exclusions for milk sugars and sugar within fruit and vegetable cell walls *(42)*:

> Under this definition lactose (milk sugar) when naturally present in milk and milk products and sugars contained within the cellular structure of foods (particularly fruits and vegetables) are excluded.

Many CACFs include packaging claims of “no added sugar” or “contains only naturally occurring sugar”, but even products that appear to be savoury can taste sweet if naturally sweet foods such as fruit purées are used (see supplementary materials). A paper describing Public Health England’s practical interpretation of the definition of free sugars has clarified that blended, pulped, puréed or extruded fruit is considered free sugars, which is a logical extension and interpretation of the WHO definition *(43)*.

As such, the presence of free sugars liberated from intrinsically sweet food ingredients, such as fruit, is an additional issue that needs to be addressed. Intense maceration and heat treatment, such as that used in the commercial production of infant purées, liberates sugars from the cell walls of fruit and vegetables *(1,44)*; intrinsic sugars contained in fruits may therefore be classed as free when foods are prepared in this way. Analysis of back-of-packet commercial product data revealed that the mean percentage energy derived from total sugar in puréed fruits was over 70% for products sold in Denmark, Spain and the United Kingdom, reaching almost 100% energy from sugar in some blended fruit products. In such instances, the total- and free-sugar content of purées is likely to be almost equivalent, and the use of blended, pulped or puréed fruit as an *ingredient* in products other than fruit purées should be limited. In contrast, lightly processed foods made at home will maintain more of the cell wall structure and therefore will have lower free (liberated from the cell) sugar contents *(1)*.

As total sugars are listed on nutrient declarations and added sugars can be identified from the ingredients list, the draft NPM addresses total and added sugars, and not free sugars. The intention of the model nevertheless is to ensure that CACFs do not contain added sugars and that their free-sugar content is as low as possible. Current infant food regulations in the European Region allow cereal-based products to contain up to 30% of energy from added sugars (7.5 g sugar/100 kcal is equivalent to 30 kcal from sugar in 100 kcal energy). The draft NPM proposes that no added sugars or sweetening agents are used in foods for infants and young children up to 36 months.

Purées containing fruit or dairy products are likely to contain 30–100% energy from total sugars (as shown in the analysis of foods in Denmark, Spain and the United Kingdom). A ban on all intrinsically sweet foods (including fruit) is neither feasible nor desirable. Consideration should nevertheless be given to how purées that contain exclusively or predominantly fruit are consumed in the diet (their frequency, not as a meal on their own) and how fruit purées are used as ingredients in other CACFs. Currently, the NPM states that fruit purées may be added to certain categories of products, but only in limited amounts. The NPM sets maximum limits by weight for fruit purées in certain categories, such as meals where consumers might rightly expect the product to be savoury and low in sugars. The aim of the maximum limits by weight is to ensure that products are less sweet and contribute less energy from free sugars. Currently, a puréed meal with meat might provide approximately 60–65 kcal per 100 g. If this contains more than 5% fruit purée, more than 30% of the total energy will come from sugars. From the databases of food products reviewed, the 5% threshold appears feasible. A salient front-of-pack indicator (with an icon, flag or traffic light stating the percentage energy from sugar, for example) on any product where total-sugar content exceeds specified thresholds has also been suggested. The thresholds differ by food category and are listed in the NPM (Table 2 in Chapter 1.2).

The thresholds for these front-of-pack indicators have been set close to the mean and median total-sugar content for the product categories, as calculated from the United Kingdom back-of-pack product data (see supplementary material). There must be a balance between what sugar levels are optimal (for health) and feasible (many fruits and vegetables are inherently high in sugar). For most categories, thresholds in the NPM were set to avoid over 40% of the products exceeding the threshold.

The draft NPM also proposes that CACFs should not include “no claims”, such as pack statements for “no added sugars”. Non-sugar sweeteners, including those marketed as “natural sweeteners”, should not be used in infant foods to avoid accustoming infants to very sweet tastes

The draft NPM for infants and young children has a separate category for vegetable purées without the addition of fruit/fruit purée, since a substantial proportion of CACFs (about 25%) in the United Kingdom contain fruit. The model recommends that vegetables are not mixed with fruit, so infants have the opportunity to develop a taste for vegetables. Overall, literature demonstrates consistently that there is no benefit from flavour–flavour learning (such as pairing fruit with vegetables) over repeated or variety exposure; repeated and variety exposure therefore appear to be the preferred approaches for shaping food preferences (see supplementary material) *(45)*. This finding highlights the significant role of single-flavour taste exposure during introduction of solid foods *(46)*.

Recognizing that some vegetables are intrinsically sweet, the threshold at which purées must include front-of-pack indicators is 30% total energy from sugar (7.5 g/100 kcals). Other foods with vegetables (plus cereals/grains) that one would expect to be savoury must also include a front-of-pack indicator if total sugar exceeds 20% (5 g/100 kcals) and savoury meal-type purées must include a front-of-pack indicator if total sugar exceeds 15%. This should discourage the production/consumption of savoury foods with high total-sugar content.

The thresholds for these indicators were set close to the mean and median total-sugar content for these product categories calculated from back-of-pack product data (see supplementary materials) and using a pragmatic approach to achieve a sensible category threshold. In most categories, the threshold resulted in fewer than 40% of existing products exceeding the limit, but for spoonable meat meals (where meat is the first food listed in the product name) in the United Kingdom, 59% exceeded the threshold (15% total energy from sugar) and would therefore require reformulation or packet labels.

To aid the identification of sweet and savoury products, previous reports have recommended that “all product names should reflect the ingredients present, so that care-givers are not misled into thinking that they are offering savoury vegetable tastes when this is not the case” *(1)*. Currently, “when fruits and vegetables are combined, the name often does not reflect the main ingredient, and many of these products have a very high proportion of puréed apple or pear, even when this is not highlighted in the name” *(1)*. As such, the NPM requires that product names reflect main ingredients and the presence of naturally sweet foods.

The NPM also addresses the issue of added sodium salts. For some product categories (fruit, desserts and puddings), the European Commission directive states, “Sodium salts may not be added except for technological purposes”. This does not apply to all food product categories, and the wording is ambiguous. The proposed NPM requires less than 50 mg/100 kcal and 50 mg/100 g sodium content across all products to prevent children becoming accustomed to a diet with high salt content. Higher thresholds are established for products where cheese appears in the

name, and the per 100 g threshold does not apply to dry or instant cereals. Based on the available compositional data examined, this appears feasible.

Only a small number of products in the United Kingdom had fat contents over the European Commission requirements of less than 4.5 g/100 kcal (~ 40% energy) from fat. Further consideration is required to determine the need, and requirements, for setting threshold levels for saturated fat. Currently, the NPM has not set new thresholds for fat content.

Should the model be designed to discriminate between foods within food categories, or across all foods?

The draft NPM for infants and young children was designed to discriminate between categories of foods because some of the current European Commission (Annex I and II of the European Commission directive 2006/125/EC (see Annexes 1 and 2 in this document)) and Codex (Codex/STAN 074-1981 revised 2017) regulations, which informed the new NPM, distinguish by food categories *(5,6)*.

Some marketing and labelling regulations apply to all food categories in the proposed NPM, but other nutrient requirements in the NPM apply to foods within specific categories (see Table 2 in Chapter 1.2). To clarify which CACFs are categorized into each category, a detailed table has been provided with examples of included and excluded products (Table 1 in Chapter 1.2).

After considering the products currently on the European market and existing product categories in published literature, broad food groups used in the draft NPM were initially based on CACF texture and form:

1. dry, powdered and instant cereals;
2. soft–wet spoonable, ready-to-eat foods (sold in jars, pots or pouches), which includes smooth purées and dairy-based products and slightly more textured foods (usually marketed for 4+ and 6+ months);
3. meals with chunky/chopped pieces (those often sold in trays/pots and usually marketed for 10+ months);
4. dry finger foods and snacks (usually marketed for 12+ months); and
5. juices and sweetened milk drinks.[2]

These main product categories initially were subdivided according to existing examples (from food data sets, literature and online retailer product categorization), then further divided or regrouped to tie in with the current European Commission directive relating to the protein, carbohydrate, fat, sodium and calcium content of product categories (see Annexes 1 and 2, which reproduce Annexes I and II of the European Commission directive 2006/125/EC on processed cereal-based foods and baby foods for infants and young children *(6)*). Annex I of the directive details the requirements for cereal-based foods (cereals composing not less than 25% of final mixture on a dry weight-for-weight basis).

Four different types of cereal-based foods are defined in Article 1 of the European Commission directive 2006/125/EC (and the Codex/STAN 074-1981 revised 2017 on cereal-based foods):

1. simple cereals which are or have to be reconstituted with milk or other appropriate nutritious liquids;

[2] Products that function as breast-milk substitutes should not be promoted and were not considered in the development of the NPM. These include any milks (or products that could be used to replace milk, such as fortified soy milk), in either liquid or powdered form, that are specifically marketed for feeding infants and young children up to the age of 3 years. However, a decision was taken to include a category for fruit drinks and juices and sweetened cow's milk/milk alternatives as they may fall out of the scope of policies and regulations applied to formula and products that function as breast-milk substitutes.

2. cereals with an added high-protein food which are or have to be reconstituted with water or other protein-free liquid;
3. pastas which are to be used after cooking in boiling water or other appropriate liquids; and
4. rusks and biscuits which are to be used either directly or, after pulverization, with the addition of water, milk or other suitable liquids.

Annex II of the directive specifies requirements relating to:

1. the amount of meat and fish and nutrients that must be present for a product to have the name of the meat/fish in the product label, depending upon whether it is mentioned first in the product name or not *(6)*;
2. nutrient content for products that mention cheese in the name of a savoury product;
3. nutrient content for "sweet dishes that mention dairy products as the first or only ingredient in the name"; and
4. nutrient content for other product types such as fruit-only dishes, fruit-based dishes, desserts and puddings.

Separate categories for these were therefore incorporated into the draft NPM to allow the existing detailed requirements to be specified for each category. Dry finger food and snacks are not mentioned explicitly in the European Commission directive 2006/125/EC, but carbohydrate, fat and sodium requirements from Annex II that appeared relevant were applied to these products in the NPM (see the "Existing regulations" column in the earlier version of the NPM in the supplementary materials).

Categories and definitions for which products are included or excluded from categories were further refined after the NPM was validated using Danish, Spanish and United Kingdom back-of-pack product data and following the pilot testing (see supplementary material).

The draft NPM for infants and young children does not permit sweet confectionery, juice or other drinks, and sets nutrient thresholds (notably for added and total sugar) in the other snacks category because of the significant detrimental effects of sugar on dental health and because accustoming infants to very sweet tastes may contribute to overweight in later childhood (see supplementary material) *(1)*.

The draft NPM did not include separate categories for fortified complementary foods (complementary foods are fortified with iron in some countries, for instance *(30)*). There may be a case for further considering content and regulation of fortified regular CACFs (that is, not products used for medicinal purposes), but micronutrient content is lacking from most CACF products.

After pilot testing with a wider group of countries, and after considering their feedback, some categories were merged and the descriptions improved to form a revised version of the NPM, which is presented in this document.

Decide whether to use an existing model (possibly with some limited adaptation) or develop a new model

Most of the food categories in the existing Regional Office NPM for marketing to children (over 36 months) (Annex 9) are not suitable for the NPM for infants and young children because they do not reflect the types of CACFs on the market that are suitable for this age group. Some

information from comparable food categories has nevertheless been used to inform the model for snack-food categories.

To avoid spillover from other products on the market targeted at older children above 36 months, which are unsuitable for infants and young children up to 36 months (they do not fit the NPM categories or thresholds), it is recommended that these products (such as sweet breakfast cereals and children's snack foods) state a minimum age of 36 months/3 years on packs. Efforts have also been made in the draft NPM, however, to establish food product categories that are currently small in terms of market share but may grow in importance as new products and future marketing strategies develop; these may be unsuitable for those up to 36 months and should not be marketed for this age group.

Decide on the scope of, and exemptions to, the model

NPMs need to be clear about which products are covered (and which are not), including, for example, whether the model should cover foods as sold or as eaten.

The draft NPM for infants and young children covers only complementary foods that are marketed and sold for infants and young children aged up to 36 months. The term "complementary foods" is used to refer to both foods and beverages. This definition applies to all commercially produced foods that are marketed as being suitable for infants and young children from the age of 6–36 months. The NPM explicitly defines products that are considered to be marketed as being suitable for this age group (see Chapter 1.2).

The NPM for infants and young children includes only foods as sold. Exclusions noted in the NPM are as follows:

- products not specifically marketed for children younger than 3 years of age as defined in WHO guidance on ending the inappropriate promotion of foods for infants and young children *(25)*;
- vitamin and mineral food supplements, whether to be consumed as tablets/drops or added to foods at home (including home-fortification products such as micronutrient powders and lipid nutrient powders);
- products that function as breast-milk substitutes (including formula milk, follow-on formula milk, so-called growing-up milks and fortified toddler milk), which fall under the scope of the International Code of Marketing of Breast-milk Substitutes and should not be promoted at all (the current document and NPM do not replace any provisions in the Code); and
- products whose labels state that they are intended only for pregnant women, mothers or children older than 3 years.

Decide the number of food categories that will be used in the model and weigh up the advantages and disadvantages of using many, few or even no categories

The number of food categories in the NPM was determined by consideration of:

- current regulations and proposed thresholds for different products (for instance, regarding protein content);
- texture and food form;
- nutrient content of products on the market (see supplementary materials); and
- information from the literature review.

Decide which nutrients and other food components should be involved, including the possible components and the advantages and disadvantages of many or few components

There are many reasons why nutrients might not end up in a model, including non-availability of composition data and co-linearity among nutrients, meaning there is no need to keep all of them in the same model. Although maximum recommended levels for iron are available by 100 kcal of food energy, the lower reference nutrient intake is given as intake per day *(47)*, which is not as useful for the NPM. Iron and zinc regulations or recommendations currently are not included in the NPM for infants and young children.

The NPM focuses currently on macronutrient and food contents of CACFs, such as the total-sugar content or proportion of a named protein source (fish, poultry, meat or other traditional source), rather than the exact protein requirement. Micronutrient contents have not been considered, apart from sodium.

Decide the reference amount for the model

Theoretically, there are many possible reference amounts that could be used in developing an NPM, but in practice, the three main ways in which the amount of a food component is expressed are:

- per 100 g of food
- per 100 kJ of food
- per serving of food.

Per 100 g or per 100 kcals of food have been used in the draft NPM, and sometimes combined within the model. Serving-size recommendations are less useful for this demographic, as portions vary depending on the infant’s age and growth, how much breast milk or formula and other foods are consumed during the day, and the complementary feeding practices in the country. Additionally, differing energy densities of apparently similar foods from different manufacturers may make serving-size recommendations in the NPM challenging to set or apply.

A maximum portion size for energy- or sugar-dense finger foods has nevertheless been proposed in the draft NPM, as this is particularly necessary for older infants and young children who are able to consume large portion sizes. Countries also may consider establishing a maximum portion size for soft–wet spoonable products.

Decide whether to use scoring or thresholds (or both) for the model

The draft NPM uses thresholds; this is likely to be clearer and easier to use than a scoring system and easier to modify appropriately for national use by different countries.

Decide which numbers should be used

Three main ways can be used to decide which numbers should be used to determine thresholds or scores:

- a pragmatic approach
- an approach that ensures consistency with, for example, legislation
- an approach derived from dietary recommendations and guidelines.

The draft NPM for infants and young children uses an approach that is informed by the European Commission directive and Codex standards, but provides further recommendations based on an

overview of the current baby food market and existing dietary recommendations from countries. Following validation and pilot testing – which assessed the application of categories, provided more information on common ingredients and composition, and evaluated the strictness of the model – a number of thresholds/provisions were adjusted to those found in the revised version presented in Chapter 1.2.

References[3]

1. Crawley H, Westland S. Baby foods in the UK. A review of commercially produced jars and pouches of baby foods marketed in the UK. London: First Steps Nutrition Trust; 2017 (http://www.firststepsnutrition.org/pdfs/Baby_Food_in_the_UK%20_2017.pdf).
2. Maslin K, Venter C. Nutritional aspects of commercially prepared infant foods in developed countries: a narrative review. Nutr Res Rev. 2017;30(1):138–48.
3. Langley-Evans SC. Nutrition in early life and the programming of adult disease: a review. J Hum Nutr Diet 2015;28(Suppl. 1):1–14.
4. Guidance on ending the inappropriate promotion of foods for infants and young children. Geneva: World Health Organization; 2017 (https://www.who.int/nutrition/topics/guidance-inapproriate-food-promotion-iyc-backgroundprocess.pdf?ua=1).
5. Codex standard for processed cereal-based foods for infants and young children. CODEX STAN 74–1981. Rome: Food and Agriculture Organization of the United Nations & World Health Organization; adopted 1981, revised 2006, amended 2017 (http://www.fao.org/fao-who-codexalimentarius/sh-proxy/en/?lnk=1&url=https%253A%252F%252Fworkspace.fao.org%252Fsites%252Fcodex%252FStandards%252FCODEX%2BSTAN%2B74-1981%252FCXS_074e.pdf).
6. The Commission of the European Communities. Commission Directive 2006/125/EC on processed cereal-based foods and baby foods for infants and young children. O. J. E. U. 2006, L 339:16–35 (http://eur-lex.europa.eu/legal-content/EN/TXT/PDF/?uri=CELEX:32006L0125&from=EN).
7. Elliott CD. Sweet and salty: nutritional content and analysis of baby and toddler foods. J Public Health (Oxf.) 2011;33(1):63–70.
8. Carstairs SA, Craig LC, Marais D, Bora QE, Kizebrink K. A comparison of preprepared commercial infant feeding meals with home-cooked recipes. Arch Dis Child. 2016;101(11):1037–42.
9. Mesch CM, Stimming M, Foterek K, Hilbig A, Alexy U, Kersting M et al. Food variety in commercial and homemade complementary meals for infants in Germany. Market survey and dietary practice. Appetite 2014;76:113–9.
10. Garcia AL, Raza S, Parrett A, Wright CM. Nutritional content of infant commercial weaning foods in the UK. Arch Dis Child. 2013;98(10):793–7.
11. Hilbig A, Foterek K, Kersting M, Alexy U. Home-made and commercial complementary meals in German infants: results of the DONALD study. J Hum Nutr Diet. 2015;28(6):613–22.
12. Maalouf J, Cogswell ME, Bates M, Yuan K, Scanlon KS, Pehrsson P et al. Sodium, sugar, and fat content of complementary infant and toddler foods sold in the United States, 2015. Am J Clin Nutr. 2017;105(6):1443–52.
13. Dunford E, Louie JC, Byrne R, Walker KZ, Flood VM. The nutritional profile of baby and toddler food products sold in Australian supermarkets. Matern Child Health J 2015;19(12):2598–604.
14. Elliott CD, Conlon MJ. Packaged baby and toddler foods: questions of sugar and sodium. Pediatr Obes. 2015;10(2):149–55.
15. Garcia AL, McLean K, Wright CM. Types of fruits and vegetables used in commercial baby foods and their contribution to sugar content. Matern Child Nutr. 2016;12(4):838–47.
16. Cogswell ME, Gunn JP, Yuan K, Park S, Merritt R. Sodium and sugar in complementary infant and toddler foods sold in the United States. Pediatrics 2015;135(31):416–23.
17. Elliott CD, Conlon MJ. Toddler foods, children's foods: assessing sodium in packaged supermarket foods targeted at children. Public Health Nutr. 2011;14(3):490–8.
18. Regulation (EU) No. 609/2013 of the European Parliament and of The Council of 12 June 2013 on food intended for infants and young children, food for special medical purposes, and total diet replacement for weight control and repealing Council Directive 92/52/EEC, Commission Directives 96/8/EC, 1999/21/EC, 2006/125/EC and 2006/141/EC, Directive 2009/39/EC of the European Parliament and of the Council and Commission Regulations (EC) No 41/2009 and (EC) No 953/2009. Strasbourg: European Parliament; 2013 (http://extwprlegs1.fao.org/docs/pdf/eur125448.pdf).
19. Annexes 1 to 5 to the Commission delegated regulation (EU) supplementing Regulation (EU) No. 609/2013 of the European Parliament and of the Council as regards the specific compositional and information requirements for processed cereal-based food and baby food. Brussels, 25.9.2015 C(2015)

[3] All weblinks accessed 12 March 2019.

6507 final. Brussels: European Commission; 2015 (http://www.europarl.europa.eu/RegData/docs_autres_institutions/commission_europeenne/actes_delegues/2015/06507/COM_ADL(2015)06507(ANN)_EN.pdf).
20. P8 TA(2016)0015. Objection to a delegated act: specific compositional and information requirements for processed cereal-based food and baby food. Strasbourg: European Parliament; 2016 (http://www.europarl.europa.eu/sides/getDoc.do?pubRef=-//EP//NONSGML+TA+P8-TA-2016-0015+0+DOC+PDF+V0//EN).
21. Resolution WHA69.9. Ending inappropriate promotion of foods for infants and young children. Sixty-ninth World Health Assembly agenda item 12.1. Geneva: World Health Organization; 2016 (http://apps.who.int/gb/ebwha/pdf_files/WHA69/A69_R9-en.pdf).
22. European food and nutrition action plan 2015–2020. Copenhagen: WHO Regional Office for Europe; 2014 (http://www.euro.who.int/en/publications/abstracts/european-food-and-nutrition-action-plan-20152020-2014).
23. Lennox A, Sommerville J, Ong K, Henderson H, Allen R, editors. Diet and nutrition survey of infants and young children, 2011. London: Department of Health and Social Care; 2013 (https://www.gov.uk/government/publications/diet-and-nutrition-survey-of-infants-and-young-children-2011).
24. Guidance on ending the inappropriate promotion of foods for infants and young children. Implementation manual. Geneva: World Health Organization; 2017 (http://apps.who.int/iris/bitstream/handle/10665/260137/9789241513470-eng.pdf;jsessionid=E2794EB5967BC17BAA1F78A8A66C2B04?sequence=1).
25. Maternal, infant and young child nutrition. Guidance on ending the inappropriate promotion of foods for infants and young children. Report by the Secretariat. In: Sixty-ninth World Health Assembly: documentation [website]. Geneva: World Health Organization; 2016 (Document A69/7 Add.1; http://apps.who.int/gb/e/e_wha69.html).
26. International Code of Marketing of Breast-milk Substitutes. Geneva: World Health Organization; 1981 (https://www.who.int/nutrition/publications/code_english.pdf).
27. Nutrient profiling. Report of a WHO/IASO technical meeting. London, United Kingdom 4–6 October 2010. Geneva: World Health Organization; 2011 (http://www.who.int/nutrition/publications/profiling/WHO_IASO_report2010/en/).
28. WHO Regional Office for Europe nutrient profile model. Copenhagen: WHO Regional Office for Europe; 2015 (http://www.euro.who.int/en/health-topics/disease-prevention/nutrition/publications/2015/who-regional-office-for-europe-nutrient-profile-model-2015).
29. Dewey KG. Nutrition, growth, and complementary feeding of the brestfed infant. Pediatr Clin North Am. 2001;48(1):87–104.
30. Pan American Health Organization, World Health Organization. Guiding principles for complementary feeding of the breastfed child. Washington: Pan American Health Organization; 2003 (https://www.ennonline.net/compfeedingprinciples).
31. Daniels L, Mallan KM, Fildes A, Wilson J. The timing of solid introduction in an "obesogenic" environment: a narrative review of the evidence and methodological issues. Aust N Z J Public Health 2015;39(4):366–73.
32. Hetherington MM, Cecil JE, Jackson DM, Schwartz C. Feeding infants and young children. From guidelines to practice. Appetite 2011;57(3):791–5.
33. Lauria L, Spinelli A, Grandolfo M. Prevalence of breastfeeding in Italy: a population based follow-up study. Ann Ist Super Sanita 2016;52(3):457–61.
34. McAndrew F, Thompson J, Fellows L, Large A, Speed M, Renfrew MJ. Infant Feeding Survey 2010. Leeds: Health and Social Care Information Centre, IFF Research; 2012 (http://digital.nhs.uk/catalogue/PUB08694).
35. Weissenborn A, Abou-Dakn M, Bergmann R, Both D, Gresens R, Hahn B et al. Stillhäufigkeit und Stilldauer in Deutschland – eine systematische Übersicht [Breastfeeding rates and duration in Germany – a systematic review]. Gesundheitswesen 2016;78(11):695–707 (in German).
36. Michaelsen KF, Greer FR. Protein needs early in life and long-term health. Am J Clin Nutr. 2014;99(3):718S–22S.
37. Tang M, Krebs NF. High protein intake from meat as complementary food increases growth but not adiposity in breastfed infants: a randomized trial. Am J Clin Nutr. 2014;100(5):1322–8.

38. Koletzko B, von Kries R, Closa R, Escribano J, Scaglioni S, Giovannini M et al. Lower protein in infant formula is associated with lower weight up to age 2 y: a randomized clinical trial. Am J Clin Nutr. 2009;89(6):1836–45.
39. Coulthard H, Harris G, Emmett P. Delayed introduction of lumpy foods to children during the complementary feeding period affects child's food acceptance and feeding at 7 years of age. Matern Child Nutr. 2009;5(1):75–85.
40. Vos MB, Kaar JL, Welsh JA, van Horn LV, Feig DI, Anderson CAM et al. Added sugars and cardiovascular disease risk in children: a scientific statement from the American Heart Association. Circulation 2017;135(19):e1017–34.
41. Guideline: sugars intake for adults and children. Geneva: World Health Organization; 2015 (http://www.who.int/nutrition/publications/guidelines/sugars_intake/en/).
42. Buttriss J. Why 5%? An explanation of the Scientific Advisory Committee on Nutrition’s recommendations about sugars and health, in the context of current intakes of free sugars, other dietary recommendations and the changes in dietary habits needed to reduce consumption of free sugars to 5% of dietary energy. London: Public Health England; 2015 (https://www.gov.uk/government/publications/sacns-sugars-and-health-recommendations-why-5).
43. Swan GE, Powell NA, Knowles BL, Bush MT, Levy LB. Short communication. A definition of free sugars for the UK. Public Health Nutr. 2018;21(9):1636–8. doi:10.1017/S136898001800085X.
44. Scientific Advisory Committee on Nutrition 48th meeting, 30th June 2016, Skipton House, London. Final minutes. London: SACN; 2016 (https://app.box.com/s/qv74594fo7mwxgsqbrvc5fdbrycchma4/1/8429053429/77229943237/1).
45. Anzman-Frasca S, Ventura AK, Ehrenberg S, Myers KP. Promoting healthy food preferences from the start: a narrative review of food preference learning from the prenatal period through early childhood. Obes Rev. 2018;19(4):576–604.
46. Mura Paroche M, Caton SJ, Vereijken C, Weenen H, Houston-Price C. How infants and young children learn about food: a systematic review. Front Psychol. 2017;8:1046.
47. World Health Organization, Food and Agricultural Organization of the United Nations. Vitamin and mineral requirements in human nutrition, 2nd edition. Geneva: World Health Organization; 2004 (http://www.who.int/nutrition/publications/micronutrients/9241546123/en/).

PART 2

Supplementary material

2.1. Literature review of complementary feeding practices and marketing of commercial products

The aim of this literature review was to inform the development of the NPM in terms of food categorization and nutrient or marketing requirements for each category. As the number and type of CACF available on the market has expanded, it has become apparent that the European Commission directive and Codex standards may need to be supplemented with additional nutrient recommendations (for total sugar, for instance), content recommendations (to promote non-sweet or bitter flavours, for example) or with more practical marketing restrictions (to prevent, for instance, misleading product-naming and health claims). The rapid review informed the early stages of work on the NPM.

The literature review therefore aimed to examine evidence in relation to key topical issues identified in the WHO guidance *(1)* in terms of:

- age of complementary food introduction
- how complementary food tastes and textures affect food preferences
- evidence relating to CACF food tastes, texture and nutrition
- potential health implications of consuming CACFs
- how product marketing and packaging influences infants' and young children's diet.

A rapid scoping review approach was taken to summarize evidence for important themes.

Age of solid-food introduction

Complementary feeding is needed when breast milk alone is no longer sufficient to meet the needs of growing infants. The target age range is generally taken to be 6–24 months, even though breastfeeding may continue beyond two years *(2)*. A WHO expert consultation on the optimum duration of breastfeeding concluded in 2002 that the potential health benefits of waiting until 6 months to introduce complementary foods outweigh any potential risks, and this recommendation still stands today *(3)*.

Breastfeeding represents the physiological ideal for infant feeding, so complementary feeding (its role, timing and type) should only be considered within the context of potential benefits versus the risk of displacing breast milk *(4)*. According to guidance from the National Health Service in the United Kingdom, it is rare for all of the signs that a baby is ready to accept their first food to appear before 6 months of age. Some parents, however, may mistake behaviour such as chewing fists, wanting extra milk or waking in the night to indicate the need to introduce complementary foods *(5)*. Although some concerns have been raised that exclusive breastfeeding up to 6 months may limit intake of some micronutrients for some infants *(2,6)*, it is generally accepted that the nutrient needs of full-term, normal-birth-weight infants can typically be met with human milk alone for the first six months, if the mother is well nourished *(6–8)*. Recent evidence also reinforces the position of WHO that exclusive breastfeeding provides sufficient energy and does not constrain infant growth for around the first six months of life *(4)*.

In contrast to WHO, the European Food Safety Authority (EFSA) opinion on complementary feeding of infants concluded that *(6)*:

> on the basis of present knowledge the introduction of complementary food into the diet of healthy term infants in the EU between the age of 4 and 6 months is safe and does not pose a risk for adverse health effects (both in the short-term, including infections and retarded or excessive weight gain, and possible long-term effects such as allergy and obesity).

The EFSA panel acknowledged that their conclusions (on introducing complementary foods before 6 months) were largely based on evidence from exclusively breastfed infants and, as noted above, that exclusive breastfeeding to 6 months generally provides adequate nutrition. Given that this conclusion contradicts long-standing WHO recommendations, it would be useful to pose the question as to whether introducing foods before 6 months has any potential *beneficial effects*, rather than to recommend this practice based on insufficient evidence of harms.

A recent position paper from the European Society for Paediatric Gastroenterology, Hepatology, and Nutrition (ESPGHAN) Committee on Nutrition highlighted that the limited scientific evidence surrounding complementary feeding practices explains the variation in recommendations. The Committee recommended that complementary foods and liquids should not be introduced before 4 months or delayed past 6 months *(7)* and stated that exclusive breastfeeding should be promoted up to 4 months and *exclusive or predominant* breastfeeding be promoted up to 6 months.

For infants between 4 and 6 months, a recent Cochrane review concluded that there is no evidence of benefit from introducing additional foods to infants who are exclusively breastfed, nor any risk related to morbidity or weight change (low- and moderate-quality evidence) *(9)*. Moderate-quality evidence was also found showing that the early (before 6 months) introduction of potentially allergenic foods, compared to exclusive breastfeeding, did not lower the risk of food allergies between 1 and 3 years. The authors conclude there is no evidence to disagree with current recommendations that healthy infants should exclusively be breastfed for the first six months. This conclusion is in line with an earlier Cochrane review that identified exclusive breastfeeding for six months, compared to exclusive breastfeeding for a shorter duration, confers health benefits and no apparent harms *(10)*.

The recent and comprehensive report from the United Kingdom SACN of evidence on feeding in the first year of life also concluded that exclusive breastfeeding for the first six months, as compared to four months with mixed feeding thereafter, is beneficial for, and does not adversely affect, infant health *(4,10)*. SACN also identified that introducing solids at 3–4 months is associated with higher risk of gastrointestinal, respiratory and ear infections than continuing exclusive breastfeeding *(4)*. Evidence to support the WHO recommendation for exclusive breastfeeding to 6 months *(3)* therefore continues to emerge.

Despite this long-standing WHO recommendation on the suitable age of complementary food introduction, it is clear that foods are introduced much earlier. Nearly two thirds of infants under 6 months are not exclusively breastfed, and fewer than one in five in high-income countries are breastfed for 12 months. The 2011 United Kingdom diet and nutrition survey of infants and young children between 4 and 18 months reported that complementary foods (either home or commercial) were introduced before the age of 3 months for 10% of children, and before 5 months for 75%. Foods were introduced at 6 months for 22% of children and at 7 months or more for 3% *(11)*. Almost half of CACFs in the United Kingdom (from the four largest manufacturers) are marketed for infants less than 6 months old. Allowing product labels to state they are suitable from children of 4 months seems likely to encourage the earlier introduction of complementary foods, contrary to WHO recommendations *(12)*.
Since many infants are exposed to foods before 6 months, it is important to examine the risk of later food sensitivity. A recent review conducted in the United Kingdom identified that the introduction of some foods (egg, peanut or fish) before 6 months is not associated with higher risk of some allergenic sensitivity. The review found high-quality evidence to support the conclusion that introducing gluten from 4–6 months is not associated with coeliac disease,

moderate-quality evidence to support that egg introduction between 4–6 months and peanut between 4–11 months is not associated with egg or peanut allergy, respectively, low-quality evidence that introducing fish between 6 and 12 months is associated with reduced allergic rhinoconjunctivitis, and very low-quality evidence that fish introduction before 6–9 months may reduce the risk of allergic sensitization *(13)*.

Despite the long-stated recommendation from WHO, the age at which to introduce solid foods continues to be controversial and confusing, with a lack of policy coherence among major organizations in Europe. This may in part be driven by the persistent gap between the recommendation for exclusive breastfeeding for six months and the reality that this is often not achieved, with the age for introduction to solid foods being closer to 4 or 5 months in many countries *(14,15)*. Conflicting recommendations on the age at which to introduce complementary foods from leading organizations, including EFSA, mean that many products are currently marketed as being suitable from 4 months of age, despite WHO guidelines that foods should not be marketed for infants less than 6 months *(1)*. Based on the evidence reviewed by WHO and summarized here, commercial products should not be marketed as suitable for infants under 6 months old as:

1. **breast milk provides adequate nutrition for most infants;**
2. **early complementary foods are not more nutrient- or energy-dense than usual milk (discussed below); and**
3. **there is insufficient evidence of clear benefit for introducing foods before 6 months of age.**

Development of taste preferences

Young children and babies have an innate preference for sweet tastes and dislike of bitter and sour flavours. Preferences are malleable, however, and it is the interplay of biological, social and environmental factors that shape food preferences *(16)*. Behaviours and preferences established in early childhood tend to track into later years *(17)*. Parents and caregivers therefore have pivotal roles in exposing infants to varied and healthy foods during the introduction of solid foods and in providing supportive environments for this to continue as children grow up. Child needs may not match the caregiver's perceptions in terms of how or which foods are offered, and the needs of individual child development must be taken into account *(4)*.

Many studies have explored how children learn about food, and a recent systematic review neatly summarizes evidence for different learning processes *(17)*. Despite identifying 48 studies, largely from the United States of America and Europe, evidence for how infants learn is limited, in that many studies focus only on fruit and vegetables rather than other wholesome foods such as fish and wholegrains. Robust evidence was found to support the role of familiarization via *repeated exposure*: this is an important approach for food acceptance during introduction of solid foods and early childhood. Repeated exposure to novel foods, even just a few times, has been shown to reduce neophobic tendencies, increase food intake and increase taste preferences *(4,16)*. For instance, the review identified many studies on vegetable tastes that found repeated exposure leads to greater immediate and longer-term liking for vegetables *(17)*.
Despite the growing popular belief that vegetables should be introduced before fruit, few studies have systematically examined whether the timing of food introduction influences acceptance. There is therefore limited evidence to support the introduction of one food type before others *(18)*, but a recent paper from ESPGHAN recommends that infants should be offered bitter-tasting green vegetables during introduction of solid foods *(7)*; exposure to a range of flavours may therefore be more important than the order of food introduction.

Exposure to a *variety of foods* at a young age has been identified as being effective in promoting acceptance and increasing intake of the food to which the child is exposed, as well as other foods *(4,17)*. The link between taste variation and better future general acceptance of all foods may explain why breastfed infants, who are exposed to varying flavours in breast milk as opposed to a single flavour in formula, tend to show better food acceptance later on *(19)*. During breastfeeding, infants are exposed to flavour compounds in breast milk *(16)*, and evidence suggests that exposure to food tastes before 6 months of age programmes acceptance for bitter and other specific foods *(20)*.

Associative learning may be characterized as flavour–flavour learning (FFL) (such as pairing disliked foods with liked foods) and flavour–nutrient learning (FNL) (new foods are paired with energy-dense nutrients). Overall, the literature is consistent in demonstrating no benefit of FFL or FNL over repeated exposure *(17)*. Given the small number of human studies on associative learning, more research is needed, with the literature currently supporting repeated exposure and variety exposure more strongly *(18)*. These findings highlight the significant role of single-flavour taste exposure during introduction of solid foods *(17)*. During the first year of life, infants cannot distinguish foods from non-foods; this knowledge comes with experience and development, but older infants learn about food through categorization and can recognize new foods as belonging to familiar categories. Toddlers therefore recognize food by shape and colour and tend to *prefer foods presented separately* rather than combined with other foods or hidden in sauce *(17)*.

Parents were identified as highly influential in shaping food preferences through observational learning (that is, social learning and modelling) from 17 studies of generally high quality. Observational learning appears to be more relevant for older infants than at the beginning of solid food introduction, but this conclusion was reached because of a dearth of studies in younger children. Evidence supports the position that demonstrating healthy eating predicts lower fussiness and is a stronger predictor for children's fruit and vegetable intake in the longer term than parenting style or socioeconomic status. The correlation between children's food intake and that of their surrounding family may also be attributed to the simple availability of foods rather than to observational learning *(17)*.

The concept of a so-called sensitive period or critical window for exposure to key flavours and foods is widely discussed in literature, whereby exposing infants to certain foods during an optimum window may increase later food acceptance *(19,21)*. The *early* sensitive period hypothesis (sensitive to introduction between 4 and 6 months) for influencing later food acceptance is not supported by good evidence, and introducing foods after 6 months is not associated with difficulty in accepting solid foods *(4)*. The hypothesis is also contradicted by WHO recommendations to introduce complementary foods from 6 months *(2,22)*.

Overall, it appears that food preferences are not fixed and that through *frequent* and *varied* exposure to different *tastes* from *early* (that is, after 6 months) in the process of introducing solid foods, caregivers can improve later food acceptance and later preferences. It is unclear when exposure to different tastes should begin, but it seems that exposure to vegetable and bitter flavours is important as part of a varied diet and may enhance later acceptance. *Single flavours* and *more recognizable foods* (not blended together) may also be advantageous in learning and in the development of taste and food preferences.

Sweet and savoury flavours in commercial foods

Since infants have an inborn preference for sweet tastes, early and repeated exposure to sweet complementary foods is particularly worrying as it may further reinforce sweet preferences *(16)*. Using a criterion to define foods with more than 20% energy from sugar as "poor", a Canadian study found 53% of commercial baby and toddler foods were of poor nutritional quality. Baby desserts, teething biscuits and fruit/yogurt snacks and cereals had the greatest energy provided by sugar *(23)*.

An infant's predisposition for sweet foods can easily be exploited by food companies *(24)* who produce and market highly palatable foods with high free-sugar contents and even manufacture savoury foods with sweet taste profiles. Such foods may condition children into hard-to-break habits, such as becoming accustomed to frequent sweet food intake *(24)*. Marketing sweet dessert-type foods and those flavoured with vanilla or chocolate for the period of early solid food introduction may also serve to reinforce social norms about the suitability of such foods for solid food introduction. A United Kingdom report recommends that for nutrient-rich foods to be provided as first foods around 6 months, when the volume of food intake will be low, dessert foods should not be introduced until 10 months, with a focus instead on providing breakfast and two savoury meals *(25)*.

Although fruits and vegetables are healthy foods and provide essential micronutrients, studies have identified that sweet and fruit-based products dominate the United Kingdom and Australian CACF markets *(12,26,27)*. While fruit-based products technically adhere to many dietary guidelines and may not contain added sugars, they are rich in intrinsic sugar, which is equally likely to contribute to taste preference development, excess energy intake or tooth decay *(26)*. Sweet fruits and vegetables serve to increase the total-sugar content of foods without the requirement for added sugars, so caregivers think they are providing healthy foods while reinforcing preferences for sweet foods *(27)*.

Sweet products dominate the market for early complementary foods (labelled as 4+ or 6+ months) *(12)* and many infants are therefore likely to be exposed to very sweet foods early in the process of solid food introduction. The observation from one prospective observational study that greater sugar intake in children is associated with higher commercial food intake in infancy supports the hypothesis that commercial foods in early infancy facilitate later life choices for sweeter foods *(28)*. Offering home-prepared foods or carefully selected commercial foods may be a useful strategy to reduce sugar intake in infancy and beyond *(28)*.

In terms of fruit and vegetable content, a United Kingdom study identified that CACFs more frequently include fruits rather than vegetables in the name. The six most common fruits and vegetables were all relatively sweet (apple, banana, tomato, mango, carrot and sweet potato) and the sweet foods contained more fruit (64%) than the savoury foods contained vegetables (46%). Fruit juice was also added to 18% of products *(27)* and analysis of United Kingdom products identified that 19% of puréed meals, 22% of meals with chunky/chopped pieces and 24% of vegetable purées also contained fruit. Sweet and fruit-based products were also more likely to be those targeted as suitable for 4+ months of age *(12,27)*. The total-sugar content of foods (including savoury spoonable foods) was found to correlate with the content of fruit or vegetables, suggesting that these ingredients often act as sweetening agents *(27)*.

In terms of food variety, CACFs in the United Kingdom most often included three different fruits or vegetables per food, combined vegetables with fruit tastes, and rarely included bitter vegetables *(27)*, all of which may not be ideal for learning about varied flavours (as discussed

above). A comparison of commercial foods and home recipes in the United Kingdom also identified that commercial vegetable foods are likely to contain three vegetables, whereas home foods contain two on average *(29)*.

In Germany, three-day weighed dietary records identified that vegetable variety was low for all infants aged 6 and 9 months, with carrot being the most common vegetable and the use of other vegetables far below. In the same study, vegetable variety at 12 months was greater in those fed commercial meals *(30)*. While a greater vegetable variety per meal may appear advantageous, such foods may not facilitate between-meal variety in terms of vegetable taste. The German study identified that commercial meals may often contain carrot and tomato to optimize colour and make products more marketable, and the study authors recommend that single-vegetable meals should also be emphasized in infant diets to improve familiarity with these flavours *(30)*.

Interestingly, a prospective longitudinal study in the United Kingdom found a positive association between home-cooked, but not ready prepared, fruit and vegetable intake at 6 months and subsequent intake at age 7. There was also evidence of an interaction between age of introduction and frequency of exposure, suggesting that later initial exposure to *home-prepared* fruit and vegetables (after 5.6 months in this study) must be accompanied by more frequent exposure to reach the equivalent intake at 7 years of infants who were exposed earlier (before 5.6 months). Contrastingly, early provision of *ready prepared* fruit and vegetables was not associated with intake at age 7, but the authors caution that their observations may be a result of preference development or of supply, in that families who eat fewer vegetables are more likely to buy ready prepared complementary foods *(22)*.

While there is increasing interest in the role of sugar and recommendations for total- and free-sugar intake are now lower, some researchers argue that the lack of drastic national action to tackle the detrimental impact of sugar on health results from scientific confusion and is partly induced by pressure from major industrial interests *(31)*. Coherent and comprehensive policies are needed to support the reduction in intake of sugars from CACFs from a very young age *(24)*.

In summary, the specific role of CACFs in the development of taste preferences is unclear at present because of limited high-quality data. Since CACFs targeted for early solid food introduction (labelled as 4+ or 6+ months) have predominant sweet tastes and rarely include single food flavours or bitter vegetables, home-prepared foods may be more ideally suited to meet infants' need for exposure to a variety of single flavours, bitter flavours and other non-sweet foods. Commercial sweet foods or desserts may not be appropriate for infants and young children, especially at the start of solid food introduction. To support public health recommendations, commercial food manufacturers should aim to reduce total-sugar content and offer fewer fruit-based foods, more vegetable-based foods with single or simple flavour combinations, and savoury foods without sweet fruits or sweet vegetables. Clear and consistent information should be provided to facilitate caregivers to introduce solid foods using home-prepared foods, where possible.

The role of food texture

The recent fashion to follow the baby-led weaning (BLW) approach has brought new attention to early practices for solid food introduction. BLW advocates more than simply providing soft solid foods as first foods and also encourages a family mealtime and healthy approach to feeding infants *(32)*. There is scant evidence to support any specific advantage to either BLW or spoon-feeding of first foods *(33)*, an opinion shared by the United Kingdom's recent SACN review, which identified insufficient evidence to conclude that the BLW approach leads to better

outcomes compared to traditional feeding practices *(4)*. Opinions on which approach is best are often polarized, however *(34)*.

Development of jaw movement, tongue manipulation and swallowing reflexes progresses with age. Before solid food introduction, infants initially swallow milk that is delivered to the back of the mouth and then, during introduction of solids, must push soft food from the front to the back of the mouth. Any solid or textured foods must be mashed with action from the tongue or jaw, which initially is only an up-and-down motion *(35)*. A detailed review article found that inconsistency in study methodologies means conclusions about the age at which mastication efficiency reaches maturity, or the effect of food consistency on development of mastication efficiency, cannot be drawn. The review concludes, however, that children are more likely to accept textures they can manipulate, and early exposure to textures facilitates later acceptance *(35)*.

There has been some discussion about sensitive periods in terms of exposure to different food textures, with the introduction to solids after 6 months being associated with greater fussiness and introduction to lumpy food after 9 months being associated with more feeding issues at age 7 years *(22,36)*. The recent review from SACN identified that there is insufficient evidence (a few observational studies) to support claims that delayed texture introduction (that is, of lumpy or chewable foods) adversely affects later dietary variety *(4)*.

The obvious advantage of efficient mastication (or of providing puréed food) is that nutrients are more readily available. Chewing actions also stimulate endocrinal pathways leading to satiation *(35)*. There is some suggestion that the texture of the diet will influence orofacial muscle growth, meaning early exposure to texture may boost development and make foods easier to process. *Efficient* chewing and food manipulation in the mouth is not physiologically possible at the beginning of solid food introduction, however. Pre-mashed foods enable efficient nutrient uptake for infants who are not yet able to manipulate foods in their mouth, such as at the start of food introduction. Indeed, many cultures without access to puréed foods provide infants with mashed and pre-chewed options as their first foods *(34)*: spoon feeding iron-enriched infant cereal or puréed meat, for example, can therefore provide important nutrients that would otherwise be very challenging to introduce during BLW *(34)*. This important point is especially relevant, since the BLW approach focuses initially on tasting rather than swallowing foods; some infants may therefore be at risk of nutritional deficiency because of the low volume of food consumed *(34)*.

One of the main criticisms of the BLW approach centres on the risk of choking *(32)*, but there is some limited evidence (from one study) that choking is no more likely with the BLW than standard approach *(37)*. In this study, demographic characteristics (age of complementary food introduction, parental education, socioeconomic status and family eating habits) differed between those following the BLW approach and those not; parental reports of choking or gagging frequency or of food preferences may therefore be biased and unreliable *(34)*. Regarding the introduction of solids as first foods, there is a need to appreciate that safe oral manipulation of first solids is complex and that more complex actions are required for some foods (such as toast), which are advocated in the BLW approach *(34)*. Although infants are encouraged to grab food and self-feed during BLW, foods must be provided that are appropriate to the infant's ability and constant supervision is always required to prevent choking.

The guiding principles for feeding breastfed and non-breastfed children from WHO and the Pan American Health Organization have long recommended that food consistency and variety can be increased as infants get older, in line with their individual requirements and abilities. The guidance *(2,38)* states that:

- infants from 6 months can eat puréed, mashed and semi-solid foods
- by 8 months, most infants can also manage to self-feed finger foods
- by 12 months, most children can eat the same food types as the rest of the family.

The SACN report similarly identified that texture should be progressed from smooth to lumpy and then to more complex textures during the first months of complementary feeding, but that the speed of progression to solid textures cannot be determined from current evidence *(4)*. A United Kingdom survey identified a progression in infant ability to manage more textured foods with increasing age and most children (55%) began to eat foods with lumps, unblended or unmashed foods by age 7–9 months, but a significant number (32%) began to eat these more textured foods at 4–6 months *(11)*. It is therefore likely that most infants can manage faster progression to more textured foods (mashed/chopped) than the labels on smooth commercial foods encourage *(25)*.

Overall, the decision to introduce complementary foods and soft solids must be based on the individual development of each child, their multiple signals of readiness for feeding *(34,35)* and caregiver confidence/ability. Providing very soft foods when infants can handle greater textures may stunt development in terms of recognizing family foods, chewing skills (jaw strength and tongue manipulation of foods) and fine motor skills that are developed when children pick up and self-feed foods. Currently, there is insufficient evidence to draw conclusions about whether spoon-feeding or a BLW approach is more appropriate, but caregivers should respond to individual hunger and satiety cues *(7)*. While nutrient-enriched and puréed or mashed foods provide readily accessible nutrients and allow infants gradually to develop oral food manipulation skills, it appears that progression to textured foods may also improve eating skills. Current commercially available foods targeted for early food introduction (labelled as 4+ or 6+ months) are often very smooth in texture and do not include an upper age limit. Caregivers must therefore be guided by stricter product labelling on the appropriate age, and they should use judgement as to when these puréed foods are no longer appropriate, selecting nutrient-rich first foods and introducing a range of soft textures, and harder textures once teeth erupt. To address the various concerns over nutrition, choking risk and texture exposure during solid food introduction, iron-rich and nutrient-rich puréed/mashed foods may be provided alongside progressive exposure to textured foods, as is appropriate for infants' ability and development.

Puréed commercial foods and pouches

The increasing popularity of puréed foods that are often sold in pouches with spouts presents several issues:

- exposure to very soft and smooth foods, even when infants develop chewing ability (discussed above);
- the lower nutrient density of foods (discussed in next section);
- exposure to high concentrations of free sugars that may quickly be absorbed; and
- issues with sucking foods directly from the pouches.

Puréeing foods means much of the intrinsic sugar (within cell walls of fruit and vegetables) is liberated and is readily available. This drives up the free-sugar content of foods, compared to less processed foods. Analysis of back-of-packet commercial product data revealed that the mean percentage energy derived from total sugar in puréed fruits was over 70% for products sold in Denmark, Spain and the United Kingdom, reaching almost 100% energy from sugar in some blended fruit products. Puréeing also changes the flavour and appearance of foods, making them

less recognizable, and may lead to overeating as foods can rapidly be swallowed without chewing *(39)*.

Although most packs with a spout indicate that the contents should be squeezed onto a spoon or into a bowl, many parents squeeze the contents directly into the mouth or allow infants to suck from pouches. Some European and United States manufacturers also directly encourage this: "Older babies may enjoy slurping straight from the pouch" (Ella's Kitchen, Mango Baby Brekkie 6+ months, Stage 1); "I'm perfect to pop snuggly in a lunch box, picnic or straight into little mitts!" (Ella's Kitchen, The Red One Squished Fruits, 6+ months); "To serve: squeeze onto a spoon or serve straight from the pouch!" (Happy Baby Organic Stage 2 Baby Food, Simple Combos, Spinach, Mango & Pear).

The key problems with pouches and allowing infants to suck from packs have been neatly summarized in a recent report on commercial baby foods *(25)*:

> Why buying food in pouches and allowing infants to suck from the pouch is not recommended:
> - Sucking from a pouch does not encourage the learning of, and use of, chewing skills.
> - Children cannot distinguish what it is they are eating, and cannot see or smell the food easily.
> - Children who are given smooth foods in pouches for longer periods may become fussier eaters.
> - Children develop fine motor skills when picking up food or playing with it.
> - Puréed fruit and vegetables in pouches are high in free sugars, and sucking these foods across the teeth may contribute to tooth decay.
> - There is no portion control if food is eaten directly from the pouch, and there may be considerable food waste.

In summary, sweet foods dominate the commercial baby food market and many foods derive much of their energy content directly from free sugars (often from added fruit juice or puréed fruit and sweet vegetables). Processing foods so they can fit through spouts means they have unnecessarily limited textures, high water content and are likely to have higher free-sugar content as a result of intense maceration. Infants should be provided with foods they can see and feel, contributing to their learning and development. Single-portion foods (expected to be consumed in one sitting) do not need to be sold in resealable packs with spouts and instead can be provided in rip-top packets or other containers that allow for greater variety in terms of texture and must be served using a spoon. Any packet with a spout should include a clear statement that infants should not be fed by sucking/squeezing through the spout. Foods high in free-sugar content or of low nutritional value may include a front-of-pack indicator (such as a flag or traffic light) to highlight products with undesirable characteristics.

Nutritional quality of CACFs

Studies comparing the nutritional quality of home-prepared foods and CACFs report heterogeneous findings. A systematic review *(40)* that identified four studies in high-income countries found that a high proportion of CACFs, particularly mixed dishes, snacks and desserts, are high in added sugars, salt and fats *(12,41–43)*. The analysis of back-of-pack data from Danish, Spanish and United Kingdom CACFs (see Chapter 2.3) revealed that many foods are high in total sugars, some United Kingdom and Danish foods exceed current European Commission thresholds for maximum fat contents (40% of total energy), and some Spanish dry cereals (intended to be eaten with liquid) exceed current European Commission sodium recommendations (100 mg/100 kcal).

The nutritional content of CACFs may be a cause for concern because current content regulations or packaging requirements may be insufficient *(44)*. For example, packets are not required to indicate the trans fat or added sugar content of foods, and there are no minimum thresholds for some important micronutrients *(45)*. Evaluation of United Kingdom products revealed that total dietary fat intake may exceed recommended guidelines if CACFs are provided in addition to dessert and snack foods *(44)*. Some authors have argued that when considered in the context of a whole menu, many CACFs in the United Kingdom may not contain adequate micronutrient contents, and the content of essential (except potassium) and trace elements would be inadequate to meet United Kingdom reference nutrient intake levels if only commercial foods were provided; they highlight that this knowledge presents an opportunity for CACF manufacturers to improve the quality of their products *(46,47)*.

In contrast, laboratory assessment of other United Kingdom products revealed that the estimated intake of vitamins A and E (in a diet consisting of complementary foods plus formula) would exceed the current United Kingdom reference nutrient intake levels, and warrants further study *(48)*. The recent SACN report concluded that infant diet in the United Kingdom provides ample vitamin A, but infants who consume high volumes of fortified milk plus supplements may exceed their tolerable upper limit *(4)*. The SACN review concluded that in healthy full-term infants, endogenous iron stores should meet needs for the first six months of life, but iron-containing complementary foods (from a diverse diet) should be introduced to meet infants' needs after 6 months *(4)*. It is recommended that first complementary foods should be provided around 6 months, should include a wide range of foods and flavours, and should include iron- and calcium-containing foods *(4)*.

A Canadian study that looked at whole-diet diversity using WHO recommended food groups identified that diversity was higher for infants fed homemade rather than commercial foods, but there were no differences in terms of nutrient or energy intake *(49)*. Using a nutrient-based diet quality index in the United Kingdom, a study found that consuming CACFs was not associated with lower nutritional adequacy compared to not consuming commercial foods *(50)*. In Germany, with a few exceptions, a comparison of commercial and homemade foods did not reveal any serious inadequacy in terms of nutritional content *(51)*.

CACFs in Germany and the United Kingdom were found to have similar energy density to breast milk or formula but were much less energy-dense than appropriate/suitable homemade foods *(12,51)*. Appropriate/suitable home-prepared denser foods are likely to include a greater amount of protein, zinc and iron than commercially prepared foods, which often contain relatively small amounts of meat in dishes *(25)*; for example, although the meat-containing savoury foods in the United Kingdom had the highest iron content of all foods, this was no higher than formula milk and not much higher than foods that did not contain meat *(12)*. Supplementing the diet with CACFs of low quality may therefore not serve the intended purpose of enhancing the nutrient density of infants' diet *(12)*.

Water is often added to allow foods to flow smoothly out of pouches. This reduces the nutrient density and means a greater volume of food must be consumed to take in equivalent nutrients. Some commercial products do not list the proportion of added water (even when it may be the largest ingredient); this may mislead caregivers about the nutritional quality of foods. It is estimated that the energy density of CACFs is often lower than 0.8–1 kcal/g, a value estimated to provide dishes with adequate energy and nutrient density to suit small stomachs *(25)*. The back-of-pack analysis of current United Kingdom CACFs revealed that many foods have low energy densities, particularly fruit- and vegetable-based foods and some puréed meal-type foods (see Chapter 2.3). For Danish and Spanish products, fewer meal-type purées appeared to have very

low energy density compared to United Kingdom products, but fruit-based purées also had low energy density.

In summary, because of the infinite diversity and range of food combinations available during introduction of solid foods, it is not possible to state definitively whether commercial or homemade foods can provide so-called optimum nutrition. It is clear, however, that many individual commercial products are far from ideal, in that some have low energy or nutrient density and others are high in sugar or sodium. Preparing age-appropriate suitable homemade foods allows for better control of the nutritional composition and variety of foods *(52)*. Because very few studies directly compare homemade and commercial infant foods, further high-quality research is needed *(53)*. While all CACFs do not appear to be nutritionally inadequate, frequent selection of low-quality foods (low energy density or high sugar content) and relying heavily on CACFs may not provide the appropriate supplementary nutrition that is required for healthy growth and development. The quality of CACFs should be improved through legal requirements (non-voluntary), and foods with undesirable qualities that are regulated (natural sugar from fruit) should be clearly labelled.

Health implications of CACFs

Breastfeeding and milk intake

There are concerns that complementary feeding may displace intake of breast milk, but a systematic review published in 2015 (non-peer-reviewed) did not find high-quality evidence to support this. Rather, complementary foods were associated with shorter duration of breastfeeding *(40)*. The reviewers noted that very few studies (eight) met their criteria, and the evidence base was judged as low-quality. They also noted a handful of other studies which suggest that breast-milk intake is sensitive to the energy density and feeding frequency of all complementary foods (not only commercial), which in turn suggests some potential for displacement of breast milk depending on complementary food characteristics and frequency of feeding *(40)*. A WHO-commissioned rapid evidence review of marketing strategies, however, identified that CACF marketing affects optimal feeding practices, and found some evidence of movement away from exclusive breastfeeding up to 6 months and evidence of a reduction in the duration of breastfeeding *(54)*.

Overweight and obesity

Early or inappropriate complementary feeding practices in low- and middle-income countries may lead to poor growth, but in high-income countries, where obesity is a greater public health concern, it is uncertain how complementary feeding styles relate to obesity and long-term health *(55)* because of the dearth of high-quality evidence. Extensive work has demonstrated the beneficial role of breastfeeding for later weight status *(56,57)*, so this is not repeated here. Other risk factors for the development of childhood obesity in relation to complementary diets include rapid weight gain, premature introduction of solids and high protein intake, in addition to potential interactions with the gut microbiome *(58)*.

Rapid weight gain in infancy also predicts taller stature and some studies have identified rapid weight gain as a predictor, rather than a consequence, of solid food introduction *(4)*. The impact on gut microbiota from solid food introduction has barely been researched, but there is consistent evidence that the introduction of solids or the cessation of breastfeeding or formula feeding significantly changes the microbial composition. There is nevertheless a knowledge gap about how different foods affect the gut microbiota and the potential consequences for child and long-

term health *(59)*. The issues of energy density, protein content and age of solid food introduction are very relevant to CACFs as the protein and sugar content of commercial foods and average age of introduction differ between home-prepared foods and CACFs.

Identifying key dietary risk factors for overweight presents an opportunity for public health interventions, but whether CACFs adversely contribute to obesity risk factors is a question yet to be fully answered. In terms of CACFs, a recent non-peer-reviewed systematic review identified low-quality and insufficient evidence (just four studies) examining how commercially available products, consumed between 6 and 23 months, increase the risk of childhood obesity or chronic disease risk factors, so no clear answer was found *(40)*.

The optimum age for introducing solids, in terms of obesity risk, is an area of interest and controversy. The recent SACN review concluded that there appears to be no consistent evidence about the age of food introduction and later overweight, and it is challenging to separate out any effects of solid food intake from curtailment of breastfeeding and account for other confounding factors, such as socioeconomic status or method of milk feeding *(4)*. Difficulties in evaluating the question of whether *early complementary feeding* (before 6 months) relates to later obesity is also complicated by the well established links between confounding factors such as maternal age, smoking and education *(14)*. Understanding of this relationship will remain limited in evidence from observational studies and because of the large and persistent gap between policy and the reality that many infants are not exclusively breastfed until 6 months *(14)*.

Despite the lack of strong evidence for risks (of overweight and obesity) for introducing foods before 6 months, there is consensus that risks are greater when foods are introduced before 4 months *(14)*, particularly in non-breastfed infants *(60,61)*. There is also no evidence of benefits from solid food introduction before 6 months.

The quantity and quality of protein intake in the first two years of life has important consequences for child growth and development *(62)*. Protein requirements (as a percentage of energy) fall, and yet protein intake increases for most infants during the first years of life *(62)*. In late infancy and among toddlers, the average protein intake in many countries is 3–5 times the physiologic requirement, but with a high degree of variation *(62,63)*.

Protein from cow's milk is the largest contributor to total protein intake in toddlers and seems to have a specific effect on growth, possibly by affecting insulin-like growth factors. Despite the high protein content of meat, the amount of meat required to ensure adequate iron intake will have a smaller impact on total protein than from milk *(62)*. High protein intake is understood to adversely affect glucose homeostasis, growth trajectories and adipocyte proliferation via endocrine and metabolic responses *(62,64)*. The food source (meat, cereals or dairy, for example) and the varying amino acid profiles of foods may both influence growth and satiety, and therefore have long-term consequences for health, but further studies are required to examine the relationship between the amino acid profile of foods and satiety or growth (*62,65)*.

Tooth decay

Childhood caries can begin early in life and pose a serious problem in both developing and industrialized countries. Early childhood caries can be virulent, start soon after tooth eruption and have serious detrimental consequences. Essentially, caries form when bacteria interact with fermentable carbohydrates (sugars), resulting in acid production and enamel demineralization. Although tooth decay may be conceptualized as a microbial disease, few would deny that diet plays a crucial role *(66)*, and some argue that sugar intake is the primary driving factor for all

tooth decay *(31)*. The principal factor in the aetiology of dental caries is known to be the length of time the teeth are exposed to sugar, as bacterial acids persist for 20–40 minutes after sugar intake. Food retentiveness (how long it stays in the mouth) is also considered a contributing factor for demineralization *(66)*.

The causes of tooth decay may be conceptualized as multifactorial (that is, socioeconomic, behavioural and psychosocial) and there are undeniable factors linked to early childhood tooth decay, such as poorer economic conditions, belonging to ethnic and racial minorities, being born to single mothers, and lower parental education level *(66)*. To focus on the caries process as multifactorial results in misdirected policy, however, since the detrimental role of free-sugar intake for tooth decay is a fact that has been established through extensive scientific evidence *(31)*. Free-sugar intake alone (both quantity and frequency) therefore may be considered as the single most important determinant of tooth decay *(31)*.

Sugar intake is high in many infant populations and sugars contribute significantly to total energy intake. In the United States, infants of 12–24 months consume 30% of their total energy (90.5 g/d) as sugars, mainly through naturally occurring (64.4 g/d) rather than added sugar *(67)*. Of the natural sugars, approximately half are from dairy sources, but added sugars (8.4% of energy intake) make a significant contribution to total energy and are derived from fruit drinks, baked goods and confectionery. A comparison of nationally representative data from 10 countries found that infants (less than 4 years) had the highest proportion of energy from total sugar. Infants relying exclusively on milk-based diets derived 38% of their energy from total sugar, decreasing to 20–30% energy when solid foods were introduced. From the age of 1 year, added sugar intake also began to rise *(68)*.

Prospective studies provide evidence supporting the link between sugar intake in infancy and subsequent tooth decay. Finnish studies have provided mechanistic evidence to show that sucrose intake at 3 years increases aciduric oral flora and is also associated with poorer oral health at 6 years *(69,70)*. A cohort study from Brazil with long follow up identified that the increase in dental caries from 6 to 18 years was consistently and positively associated with high sugar intake patterns, after adjustment for confounding factors. Caries risk increases even in low-sugar consumers *(71)*, indicating the absence of a threshold, and that sugar intake should be as low as possible.

The 2015 WHO guideline on sugars intake in adults and children makes a “strong recommendation” to reduce the intake of free sugars throughout the life-course and recommends that free-sugar intake should not exceed 10% of total energy intake *(72)*. The report defines free sugars as mono- and disaccharides added to foods or beverages during production or before consumption, and sugars that are naturally present in honey, syrups, fruit juice and fruit juice concentrates *(72)*. The guidelines were derived, in part, from a comprehensive systematic review which found consistent evidence and a large effect size supporting a relationship between sugar intake and dental caries *(73)*, leading to WHO recommending that the intake of free sugars should be as low as possible *(72)*. The review concluded with evidence that caries in childhood track into adulthood; the findings are therefore relevant to caries risk throughout the life-course *(73)*. Eight countries in the WHO European Region report dietary reference intakes for sugar, but variation currently is large. More recent recommendations (such as the Nordic dietary recommendations) specify that added sugars should be kept to a minimum and below 10% of total energy intake.

The nutritional and corrosive characteristics of the infant diet can affect tooth tissue, though the evidence relating to infant feeding and tooth decay is inconsistent *(4)*. As diet is diversified in the

first year of life, risk of dental caries changes according to the free-sugar content and frequency of consumption of complementary food and drinks *(4)*.

Feeding behaviours for avoiding caries in the very young include avoiding repetitive intake of any liquid containing fermentable carbohydrates from a bottle or no-spill cup, avoiding between-meal snacks and avoiding prolonged exposures to any foods or beverages containing fermentable carbohydrates *(66)*. Those consuming low-sugar but high-starch diets typically have low levels of caries *(31)*, so sugary commercial recipes could be improved by lowering free-sugar content and replacing the energy with starch. A proactive approach of supporting avoidance of so-called sugar-habit development in infancy may be more effective in preventing caries than restricting intake in older children with tooth decay *(74)*.

In summary, there is insufficient evidence to state conclusively whether CACFs have a detrimental effect on breastfeeding practices, but evidence does indicate that CACFs are associated with shorter duration of breastfeeding. Complementary feeding practices appear to also influence later risk of overweight and obesity, but the specific role of commercial foods in this relationship is currently difficult to unpick. It is evident that sugar intake has significant detrimental effects on dental health. Although there is no direct evidence linking intake of CACFs to tooth decay, frequent intake of foods with high sugar contents will negatively affect oral health. Exposure to free sugars can be minimized by avoiding sugary foods, avoiding repetitive intake of liquids that contain sugars and avoiding between-meal snacks. Infants may be exposed to high levels of free sugars via puréed fruit-based foods. Sweet snack foods and fruit juices that contain sugar (including naturally occurring sugar from fruit) may not be suitable for infants. Since many CACFs are fruit-based purées/smoothies and have a high free-sugar content, it is reasonable to conclude that they may pose a threat to the very young as their first teeth erupt if eaten frequently or over prolonged periods. Free-sugar intake for infants should be as low as possible and policies are required for CACFs to support parents and caregivers to achieve this.

Marketing: claims and aspirational statements

The WHO guidance on ending the inappropriate promotion of foods for infants and young children aims to "protect and support breastfeeding, prevent obesity and noncommunicable diseases, promote healthy diets, and ensure that caregivers receive clear and accurate information on feeding". The guidance applies to all commercially produced food and beverages marketed as suitable for infants and young children from 6 to 36 months of age and states that optimal complementary feeding should focus on suitable, nutrient-rich, home-prepared and locally available foods *(1)*.

WHO also recommends that irrespective of the format (online information, sponsorship or packaging labels), there should always be a statement about the importance of continued breastfeeding for up to 2 years or beyond. The guidance states that messages should not make any suggestions that they are suitable for infants under 6 months. The Sixty-ninth World Health Assembly called upon Member States, manufacturers and distributors, health-care professionals, the media and civil society to support the WHO guidelines. Manufacturers and distributors are requested to end all forms of inappropriate promotion (such as advertising that products are suitable for infants under 6 months) and state the importance of ongoing breastfeeding *(1)*.

European Commission labelling requirements for processed cereal-based foods and baby foods for infants and young children (Annex 10) were included in the 2006 European Commission

directive. These are very brief, are outdated in terms of new food packaging (such as pouches), recommend that foods may be listed as suitable from 4 months of age and make no attempt to limit highly persuasive marketing and health claims on product packaging. This is in contrast to CAC/GL 23-1997 Codex guidelines, which state that nutrition and health claims shall not be permitted for foods for infants and young children except where specifically provided for in relevant Codex standards or national legislation. As such, regulatory provisions relating to the labelling of CACFs need to be updated, and claims should be restricted.

A WHO-commissioned review of CACF marketing and the impact on carer attitudes and infant and young children feeding practices raises serious concerns over the effects of marketing on encouraging early food introduction and displacement of home-prepared foods. The review identified that marketing strategies are based on the understanding of, and playing to, mothers' concerns about the nutritional and health needs of their child. The review also found that marketing can mislead and confuse caregivers about nutritional and health-related qualities of CACFs and about the appropriate age of use. Industry reports of exploiting mothers' high motivations and lack of confidence and knowledge to promote commercial foods are of great concern; marketing may be unethical, as it may exploit the vulnerability of caregivers during a transitional stage of their lives. Marketing was found to influence knowledge, attitudes, preferences and behaviours of caregivers and resulted in increased use of products and negative impacts on breastfeeding exclusivity, duration of breastfeeding or early introduction of solid foods *(54)*.

A recent narrative review of mainly European studies summarized evidence on perceptions of CACFs. The review found that perceptions are influenced by educational level, parity, previous experience of solid food introduction and cultural factors. Second-time mothers and those from lower socioeconomic groups were more likely to have higher perceptions of commercial foods. Across studies in different countries, homemade foods were generally perceived well and CACFs more negatively among all socioeconomic groups. The review presents some insight into why CACFs are so widely used, which includes convenience and portability, as well as the perceived disadvantages of commercial foods, such as poorer taste and less nutritional content. Some study participants viewed CACFs as "safer", possibly composed of better or more varied ingredients, or identified homemade food as laborious and wasteful. This narrative review noted, however, that the qualitative studies it included were often small in size and were likely to be subject to participant selection bias and social desirability bias in reporting of preferences *(53)*. Irrespective of product perceptions and reasons for purchasing foods, it is clear that CACFs continue to be popular, so a large proportion of infants will be consuming such products.

Front-of-pack product names are often not representative of the main food ingredients and may mislead consumers about the relative amounts of different foods in products. This especially applies to soft and puréed foods, which often include apple or pear as the largest ingredient but do not state this on the front of pack. Other products are described as a yogurt or cereal/milk-based food, but instead consist mainly of fruit; the product name may therefore be misleading.

Online comments for some products reveal that consumers find product names misleading: for example, two online reviews for Ella's Kitchen Mango and Raspberry Rice Pudding (ingredients: organic whole milk 48%, organic apples 28%, organic rice flour 9%, organic raspberries 5%) comment that, "I wonder … why this mango and raspberry pudding is mainly milk and apples, is it because apples are a cheap filler?", and "I was expecting this to be rice pudding but it doesn't contain any rice just rice flour … it is completely smooth. Maybe consider renaming as it is very misleading" (comments taken from Shop.ellaskitchen.co.uk).

A further issue of concern that creates confusion or is misleading is that legal naming requirements apply to the legal product name, which may be significantly different from the front-of-pack or brand name of a product. If product naming requirements are to be implemented, it is vital that these apply to the front of pack and also the legal product name to ensure caregivers are not misled about food contents and therefore can ensure that varied and nutritious complementary foods are provided.

It can be argued that there is a so-called halo effect around products that are specifically marketed as suitable for infants and young children, whereby one can reasonably expect such foods to be nutritionally superior to adult foods *(23)*. This is often not the case, however, with some infant foods having equivalent or higher levels of sodium or sugar as products for older children or adults *(23,41)*. The unrestricted marketing of such foods may therefore be inappropriate, and also undermines public health recommendations *(25)*. Additionally, laboratory analysis of CACFs in the United States identified that the pack labels often grossly over- or underestimated sugar content. The actual sugar content of foods ranged from 88% less to 82% more than listed on the pack; infants may therefore be at risk of greater than anticipated sugar intake *(75)*.

The issue of excessive sodium levels in foods may be less pressing in some locations where manufacturers appear to be adhering to Codex and European Commission food regulations by producing CACFs with low sodium levels *(45,76)*. For example, analysis of products currently on the market in Europe only identified a small number (largely dry cereals intended to be eaten with liquid and snack foods) that exceeded maximum threshold levels. Sodium, however, is a nutrient of potential concern and should be included in an NPM to set a strict upper limit on sodium/salt added to foods.

Most commercial infant food manufacturers use various marketing strategies to promote their products, including emphasizing the taste, healthiness and convenience of their foods *(25)*. Foods that include claims related to taste, texture, health or other aspirational statements give the impression of being optimum foods for this demographic. While individual claims may be factually true, they can distract customers' attention from less desirable attributes, imply superiority over other foods, and make it challenging to identify healthy products from the many on the market *(25,42)*. For instance, many products highlight that they have "no added sugar", despite most of the calories being derived from sugar. Caregivers may not have time to study each food item in a supermarket and, arguably, should not have to examine all details of food labels.

As a minimum, food products high in total sugars might be required to bear a label/indicator on the front of pack indicating the percentage of energy from total sugar. This may simply be a flag that indicates the sugar content, or it could be in line with traffic-light systems or warning-label schemes, as used for adult foods *(77)*, to further highlight high-sugar products. The implications of this should be considered in the context of existing rules where foods for infants and young children do not carry front-of-pack labelling. Claims should be disallowed, especially on foods high in sugar, saturated fat and salt *(42)*. The presence of many snack-food products on the market that are labelled as suitable for infants and young children may mean the idea of snacking on commercial foods becomes normalized and may encourage undesirable habits throughout childhood. Many products targeted for early solid food introduction also highlight that they are smooth and contain no bits or no chunks, reinforcing the concept that very smooth foods are the ideal way to deliver first foods.

Adding statements related to the nutritional content or quality of the ingredients may also imply superiority over home-prepared foods. For example, some products state they are “nutritionist approved”, “perfectly nutritionally balanced” or “full of goodness”. The claim that products are “nutritionally balanced” has no real meaning; it simply indicates that the product falls within a series of content regulation thresholds. Including such a claim on packs may give the impression that commercially prepared foods are superior in nutritional content to home-prepared foods *(25)*.

Commercial foods should not position themselves as “perfect” or imply superiority over other foods; for example, some Ella’s Kitchen products include a pack statement stating, “Ella’s Kitchen Stage One Baby Food is both nutritious and delicious, a perfect way to introduce your baby to solid foods”. Several Ella’s Kitchen products marketed as suitable for 4+ months also include the following statement: “The government advises that you don’t need to wean your little one until they are 6 months. Every baby is different!” The second sentence positioned after the government guideline serves to undermine it and should, instead, emphasize that introduction of solid foods before 6 months should be done under consultation with an appropriate professional.

In Australia, a federal court ruled that a product which stated on the front of pack that it was composed of fruit (“berries, apples and veg”; “peach, apple and veg”; “strawberry and apple with chia seeds”) had misled consumers. The packaging of the product contained a stylized picture of a tree on the front, with an image of a boy climbing a ladder up the tree, with coloured photographs of an apple, strawberry, raspberry and blackcurrant, with some sweetcorn kernels and slices of pumpkin. The front of the packaging carried the words “99% fruit and veg”, “no preservatives” and “no artificial colours or flavours”. The front of the box also gave prominence to the age the product was aimed at: 1–3 years. On the back, in smaller font, were the words:

> Made with 99% fruit and vegetable juice and purees, these tasty treats are a fun and convenient snack for toddlers on the go. Our range of snacks and meals encourages your toddler to independently discover the delicious taste of nutritious food. With our dedicated nutritionists who are also mums, we aim to inspire a love of nutritious food that lasts a life time.

The court held that the packaging conveyed the representation that the product was a nutritious food and beneficial to the health of children aged 1–3 years, and therefore found that this was false and misleading. The product had a very high total sugar and, since it was puréed, would also be very high in free sugars. In reaching the conclusion, the judge considered the WHO sugar guidelines *(78)*.

In summary, previous WHO calls to end inappropriate promotion of foods for infants under 6 months of age have been ignored by most large food manufacturers. Many complementary food products also have misleading front-of-pack names or include pack statements that imply superiority of commercial foods over home-prepared foods. Mandatory guidelines should be drafted to ensure product marketing and labelling does not undermine important public health recommendations for this vulnerable demographic. In particular, when it is not feasible to include a maximum sugar content threshold (such as blended fruit), products with a high sugar content should include a front-of-pack warning, and “no added sugar” statements should not be allowed on products with high sugar content. Health, nutrition or other pack claims should not be allowed so that the promotion of commercial foods does not undermine public health messages or caregivers’ confidence in home-prepared foods and imply features of the product are desirable or advantageous (such as “fits into little fingers”, “no bits”, “full of goodness” and “organic”).

2.2. Currently unresolved issues and further considerations for strengthening the proposed NPM and labelling requirements

Differences in breastfeeding practices, individual growth trajectories, and the changing energy requirements during infancy and early childhood mean that there are many competing factors to consider when developing guidance for this age group. While the development of an NPM focusing on foods that are of nutritional concern is feasible, the authors consider that certain aspects merit further consideration and periodic review, including nutritional composition of foods, minimum/maximum ages of suitability, product portion size, energy density and consumption frequency. Some of these are summarised below.

- Reflection on current portion sizes may be necessary. The changing nutritional requirements of infants and young children as they get older means that an appropriate portion size for a 6-month-old is not the same as that for an 18-month-old. A review of portion sizes and recommendations for minimum and maximum portions for CACFs may be necessary, including discussions on whether changes should be achieved through improved labelling or package size changes.
- The draft NPM for infants and young children does not distinguish between breastfed and non-breastfed infants, but there is good case to consider this in greater detail, as a high proportion of infants are not exclusively breastfed to 6 months. Further research is needed to determine whether weaning practices in breastfed and non-breastfed infants differ, and whether this affects the types of foods consumed and later health outcomes, such as overweight, obesity and related conditions. For instance, further research is needed to establish whether high protein – and what types of protein – in CACFs consumed by exclusively breastfed infants are associated with an increased risk of being overweight in childhood *(79)*.
- Balancing considerations in relation to protein and iron may require further reflection. Although the protein requirements in the European Commission directive have been retained, WHO and the authors in an earlier version of the NPM found in the supplementary material tested increasing the minimum proportion of fish, poultry, meat or other traditional sources of protein in meals to increase iron intake. The protein content of these commercial products is typically lower than comparable homemade recipes and may not be contributing sufficient iron to infants' and young children's diet, particularly for breastfed babies *(80)*. The percentage of Danish, Spanish and United Kingdom meal-type products that would have met the protein thresholds under consideration in the earlier NPM version was low, even after modelling a 5–10% increase in protein content of products. Similarly, most meal-type products from the other countries piloted did not meet the suggested increased protein thresholds, although some CACFs in other countries were found to be high in total meat content; several Italian puréed CACFs contained approximately 30% meat, fish, poultry or other traditional sources of protein, with the remainder of the product being largely added water content. Rather than being complete meals, however, these products are meant to be served with vegetables and starchy foods, and the package label should indicate this.
- While an increase in meat protein might be desirable for some products to improve iron status, consideration of a maximum protein or meat content, portion size or consumption frequency might also be needed to avoid excess intake in others. Currently, the guidance in many European countries sets maximum recommended protein intake in infants at or near 15% of energy from protein (3.75 g/100 kcal), though requirements may vary with age. Studies provide evidence on whether high protein in complementary foods (rather than breast-milk substitutes) is associated with excess weight gain later in

childhood. Although there is evidence from randomized controlled trials and observational studies that high animal protein in formula is associated with increased body mass index (BMI) in childhood, Gunther et al. *(81)* found no significant association in German infants with high protein intake at 6–12 months of age and BMI or body composition in childhood. More evidence is needed to determine whether the animal protein found in complementary foods is associated with later BMI.

- The ratio of vegetables and added water in vegetable purées may need consideration in terms of energy density. It is suggested that retained cooking water or other added water should be no more than 25% of the total product, but this could be reduced to ensure that vegetable purées provide sufficient energy. Further research may be required to determine what can realistically be recommended, based on what can be consumed successfully by infants aged 6 months. Depending on the intrinsic water content of a vegetable, adding only small amounts of water may mean the product consistency is still too difficult for young infants to consume. Consideration could also be given to recommending maximum as well as minimum energy density thresholds for certain additional product categories, such as meals with chunky pieces intended for older infants who can quickly consume large portion sizes.
- The European Commission directive currently permits salt addition to foods for technological purposes. This is vague, and it is unclear for which foods it is technologically required, who determines this (producers or regulators) and whether it remains appropriate. Lower sodium content thresholds are proposed in the draft NPM, based on assessment of feasibility and existing composition across food product categories and countries, but greater clarity on what constitutes a technological purpose in relation to CACFs would help confirm the validity of the lower thresholds established.
- Further discussion may be required on whether concentrated and processed 100% fruit (that is, fruit that is dehydrated, puréed or powdered) should be permitted as an ingredient in composite products. Such items (for example, dried apple powder from whole apples, concentrated apple purée and purée of raisins added to meals) would therefore not be allowed at all as a sweetener in baby food. Currently, the draft NPM opts for setting limits on the use of fruit purée and dried fruit purée as ingredients because these ingredients can be defined as free sugars, but the NPM stops short of completely prohibiting their use as ingredients. The NPM does not currently set any limits on sweet vegetable purées as an added ingredient. A similar discussion was presented in a meeting of the United Kingdom SACN in 2016 *(82)* relating to whether processed fruit (fruit that is stewed, puréed or dried) should be classed as added free sugars. A definition published by Public Health England determined that they should, but it is not clear how this will be applied in practice with products on the market.
- Consideration should also be given to whether sugar thresholds in the draft NPM for infants and young children should relate to free sugar to better align with WHO guidelines. A key consideration here is whether reliable estimates of free/added sugars can be produced; for purées, the amount of total sugars less the amount of lactose sugars may be a good enough estimate of free sugars, as sugars will be released as free sugars from cell walls during the puréeing process. Free sugars currently are difficult to monitor in most European countries, as free/added and lactose sugars are not required on the nutrition label. They therefore could not be included in this analysis.
- Consideration should also be given to the consequences of setting different total sugar threshold requirements for front-of-pack labels. Currently, foods for infants and young children do not carry front-of-pack labels, so parents therefore have little at-a-glance information about the nutritional composition (such as total-sugar content). It may be desirable to set different thresholds for front-of-pack labels by category to indicate

products that have a higher sugar content than would normally be expected for that category. According to the draft NPM, a savoury meal with 17% energy from total sugar, for example, will be required to state this percentage on the front of pack (because it is considered high in sugar for this product category), but a dairy-based purée with 38% energy from total sugar will not be required to do so, because of the different (higher) thresholds that apply to this product category (it contains a high proportion of lactose sugars, which are not classed as free sugars). Some formative research may be required before implementation to understand how parents would make comparisons across food product categories and how they would respond to the labels. Alternatively, a more easily understood comparison would be to estimate the percentage energy from free sugars for inclusion on the front-of-pack labels of all purées. Further research into the nature, value and consumer acceptability and understanding of such a system is required.

- Limits on the marketing of all sweet snack foods (which are defined in the NPM as having above 15% energy from total sugar) should be weighed against the risk of caregivers providing foods of worse nutritional quality (such as snacks intended for older children or adult foods). Although the aims should be to limit sugar intake in foods given to infants and young children up to 36 months and avoid early introduction of unhealthy snacking behaviours that are not recommended in dietary guidelines, it may be more practical to ensure better nutritional quality of snack foods aimed at infants and young children by allowing the total-sugar content of snacks to reach a higher level (such as 20% of total energy or 5 g/100 kcal), which may still be lower than snacks currently marketed to older children. It is likely that parents give sweet snacks to their infant or young child because they currently are labelled as suitable for this age group, but formative research with parents would be useful to understand how they would respond to changes.
- The role of teething biscuits (distinct from sweet rusks for eating as a soft cereal with added liquid) should also be considered by countries in Europe. Currently, most rusks would not pass the NPM criteria, as most have added sugars and are likely to exceed total sugar thresholds (more than 15%). In many countries, biscuits with a reasonably firm but crumbly texture are often given during teething (although they generally are not recommended by health authorities); such biscuits, with no added sugars, may not be able to achieve the same texture (although some sugar-free teething biscuits are available). The role and necessity for such products across Europe should be considered and weighed against the benefits/risks of permitting items that contain added sugars within the NPM. If teething biscuits with a small amount of added sugar are permitted, it is likely that many infant biscuit snacks will also claim the purpose of being “for teething” to ensure a place in the market.
- Finally, further consideration is necessary to determine the need for saturated fat content thresholds and, if required, the thresholds for different food categories.

2.3. Validating the NPM for infants and young children using CACFs data from Denmark, Spain and the United Kingdom

The validation and pilot testing were performed on an earlier version of the NPM, which was conducted in two phases.

In Phase 1, which is described in this chapter, product details for CACFs marketed for infants and young children up to 36 months were obtained for three European countries: Denmark, Spain and the United Kingdom. First, the composition of CACFs was explored by proposed categories and nutrient thresholds. The data were then used to validate the NPM, to determine whether categorization was appropriate and to assess, where possible, the strictness of thresholds under consideration by nutrient and overall compositional criteria. This also involved modelling product content reformulations towards the NPM thresholds.

The NPM was further validated in Phase 2 (Chapter 2.4) through pilot testing with data from seven additional countries, obtained and categorized by them for WHO following the NPM category descriptions, and using information they compiled from the package or national company websites.

Following the outcomes of the validation and pilot testing, the model was amended slightly, with some food categories being combined for the NPM presented in Chapter 1.2. Additionally, the increased protein content thresholds proposed in the earlier version of the NPM were reduced to those stated in the current European Commission regulations.

Nutrient composition of CACFs: assessment of individual nutrients in relation to current European Commission regulations and proposed thresholds

The United Kingdom CACF product data were extracted from an online repository holding a commercial database of packet-label information. The Danish and Spanish data were collected primarily from manufacturers' or supermarkets' websites. The data provided a good indication of the range of products on the market, but were not sales-weighted.

Brands in the United Kingdom data set included, among others, Asda Little Angels, Annabel Karmel, Aptamil, Cow & Gate, Ella's Kitchen, Farley's, For Aisha, HiPP, Heinz, Little Dish, Kiddylicious, Nestlé, Organix, and Pip and Pear. Those in the Danish data set included Anglamark/Coop, Aria Baby and Me, Aurion, Ella's Kitchen, HiPP, Holle, Nestlé, Organix, Quinolababy and Semper. The Spanish data set included Hero Baby, Blevit Ordesa, HiPP, Nestlé, Nutriben and Almiron.

Composition data were analysed for 768 products on sale in the United Kingdom in 2016/2017, 319 items on sale in Denmark in 2016/2017 and 241 items on sale in Spain in 2017. The product name, ingredients, packaging and nutrient details of each product were used to group CACFs for analysis, aligning them as closely as possible to product categories in the NPM. Categorization in this section varies slightly by country, as described below.

Descriptive analysis was undertaken for each country and results are displayed in Fig. 1–24 and Table 5–19. These show the energy density, proportion of energy from total sugar, total fat, saturated fat and sodium levels. The proportion of energy from sugar, fat and protein was determined by multiplying the number of grams/100 g product of sugar, fat or protein by four, nine and four, respectively, and dividing by the kcals/100 g of product. The grams/100 g of

nutrient and kcals/100 g of product were sourced from back-of-packet product information taken from the websites and the repository. Where salt content, instead of sodium, was provided on the back of packet, it was assumed these were sodium salts, and the sodium contents were estimated.

In addition to the mean, standard deviation (SD), maximum, minimum and median, the tables also show the percentage of products in each food category that exceed a number of chosen thresholds, including the percentage that do not meet specified European Commission or new suggested thresholds for individual nutrients. This exercise helped WHO and the authors to assess the proportion of existing products that exceeded certain levels and to see if the thresholds under consideration were both appropriate and achievable, given the proportion of products on the market that currently are above the thresholds. The strictness of individual thresholds and the overall compositional criteria of the NPM was assessed using the data from Denmark, Spain and the United Kingdom, as summarized in the "Assessing the overall strictness of the compositional criteria of the NPM" section below.

The percentage of products containing "added sugars" by food category was also determined; results are displayed in Chapter 2.5, Table 24–26. The list of ingredients on the pack-label data of each product was used to determine whether it contained an added sugar. For these analyses, the following listed ingredients were classed as added sugars: sugar or sucrose, dextrose, fructose, glucose, maltose, galactose, trehalose, (any) syrup, honey, malt extract/malted barley, molasses, and also juice (other than lemon or lime juice, as they are not sweet-tasting).

Lactose was not classed as an "added sugar" for these analyses or for proposed future restriction; lactose is a component of milk, and some CACF products list lactose in the ingredients along with other components of milk, rather than listing milk singly. The percentage of products in each food category containing lactose and other ingredients that may be as sweet as or sweeter than lactose (these being oligosaccharides (such as fructo-oligosaccharides or galactosaccaharides), inulin, maltodextrin, dextrinomaltosa and glycerol, most of which are found in breast-milk substitutes (formula)) are shown in Chapter 2.5, Table 27–29.

The percentage of products meeting all regulations in the European Commission directive 2006/125/EC was determined as far as possible from the available pack-label composition data, taking into account salt, protein, fat and carbohydrate content. These are displayed in Chapter 2.5, Table 30–32. It was not possible, however, to determine the amount of added sugar ingredients such as sucrose, fructose, syrups and honey, or salt added for technological purposes, as stipulated in the regulations.

Category alignment

Categories used in the analyses to validate the NPM were closely aligned to those in the earlier version of the NPM, but some minor changes in the United Kingdom categories were undertaken between the initial exploration of categories and thresholds presented in this chapter and the validation of the overall NPM compositional criteria presented below. The categories for soft–wet spoonable meals shown as "meal fish" or "meal meat" in the figures and tables include only products that have fish or a meat as the first food listed in the front-of-pack name. Those CACFs in the "meal, spoonable" category contain meat or fish but not as the first food listed in the product name. Separate categories for these meals were used because current European regulations require products with meat or fish shown first in the name to have higher protein content (10% of product weight must be from the named protein source) than those where it is not listed first (8%). Through examining product names and meat content of foods, it appears manufacturers (at least in the United Kingdom) are avoiding the requirement to use 10% of the

named protein source by using an adjective first in the name of the product (such as “Scrummy chicken dinner”). Most of these puréed products were categorized for this analysis under meat meals or fish meals rather than “meal other”.

Vegetable purées that did not contain fruit and were judged to contain less than 5% of other ingredients (except water) were analysed in a separate category. All fruit purées with or without vegetables that appeared to contain less than 5% from other ingredients were analysed in one category for Spain and Denmark, but in the initial United Kingdom analysis (where more products were available for analysis), fruit purées with vegetables were grouped separately from those without vegetables. The aim here was to understand the nutritional differences (such as total-sugar content) between the two. In the later validation analyses for the United Kingdom, these have been combined into one category for fruit purées with and without vegetables.

Similarly, products that predominately were dairy, with or without added fruit, initially were split into separate categories in the United Kingdom analysis to examine differences in macronutrient content between those sweetened with fruit and those without. Similar categorization was employed in the United Kingdom analyses for dry and instant cereals, using separate categories for those with and without fruit, but these CACFs were not split further into products with or without high-protein foods, as most did not contain milk or whey protein. In the later validation analyses for the United Kingdom, these have been combined in one category. Confectionery, sweet finger foods and rusks and teething biscuits initially were grouped in the UK analyses, but a separate category was created for rusks and teething biscuits in the later validation analyses. Some food categories included only a small number of products; to avoid misleading results, the remaining sections discuss only results from categories that had at least five products

Energy density

Puréed meal-type foods and dairy-based smooth foods in the United Kingdom tended to have higher energy density than fruit or vegetable-based purées (Fig. 1 and Table 5). Purées of fruits and (especially) vegetable-only purée often had less than 50 kcal/100 g, with minimum energy densities found for vegetable purées being 26, 20 and 50 kcal/100 g of product in the United Kingdom, Denmark and Spain (n = 3) respectively. These were likely to contain a much higher proportion of water than the 5% suggested in the earlier NPM version, but it was not possible to determine the proportion of intrinsic and extrinsic water (such as that used for cooking) in the products.

The mean energy density of soft–wet spoonable foods ranged in the United Kingdom from 50 kcal/100 g for vegetable purées to 93 kcal/100 g for foods made with dairy and fruits. In Denmark, the simple fruit and vegetable purées had lower mean energy density than most other categories (60 and 41 kcal/100 g respectively), meal purées ranged from around 46 to 73 kcal/100 g, and purées with fruit plus dairy or cereals had on average 78 kcal/100 g (Fig. 2, Table 6).

The energy density of simple vegetable purées in Spain was not as low as the United Kingdom or Denmark (66 kcal/100 g), because products included olive oil. Both smooth dairy-based and fruit plus cereals or dairy foods had the highest energy density among purées in Spain (97 and 78 kcal/100 g respectively (Fig. 3, Table 7)). Meals with chunky/chopped pieces (aimed at older-age infants) in the United Kingdom had on average 86 kcal/100 g; similarly, in Denmark, the one product in this category had the highest energy density of non-dried foods (93 kcal/100 g).

Dry cereal foods and snacks were, as expected, much higher in energy at around 400 kcal/100g (Fig. 4–6, Table 5–7). The mean energy density for savoury snacks and finger foods sold in the United Kingdom, Denmark and Spain (n = 2) was 423, 419 and 388 kcal/100 g respectively, and the mean energy density of sweet snacks and confectionery was 388, 402 and 426 kcal/100 g respectively. Four out of the nine products in the "fruit pieces" category in the United Kingdom had very high energy density (~ 500 kcal/100 g); these were found to be dried slices of fruit covered in oil (crisps).

Energy density in relation to existing and proposed thresholds

The European Commission directive 2006/125/EC does not contain any provisions relating to energy density, so in effect all products meet this directive. Applying a more than 60 kcal/100 g threshold to the energy content of different food categories, however, shows that 57%, 58% and 24% of fruit purées in the United Kingdom (without vegetables), Denmark and Spain did not meet the minimum threshold. Of the meal-type purées with meat as the first-named food, half of products in the United Kingdom and Denmark did not reach the minimum 60 kcal/100 g threshold, but all 11 Spanish meal purées with meat achieved this.

Fig. 1. Box plots[a] of energy density (kcals/100 g) of CACFs targeted for 4–36-month-olds on the United Kingdom market in 2016/2017

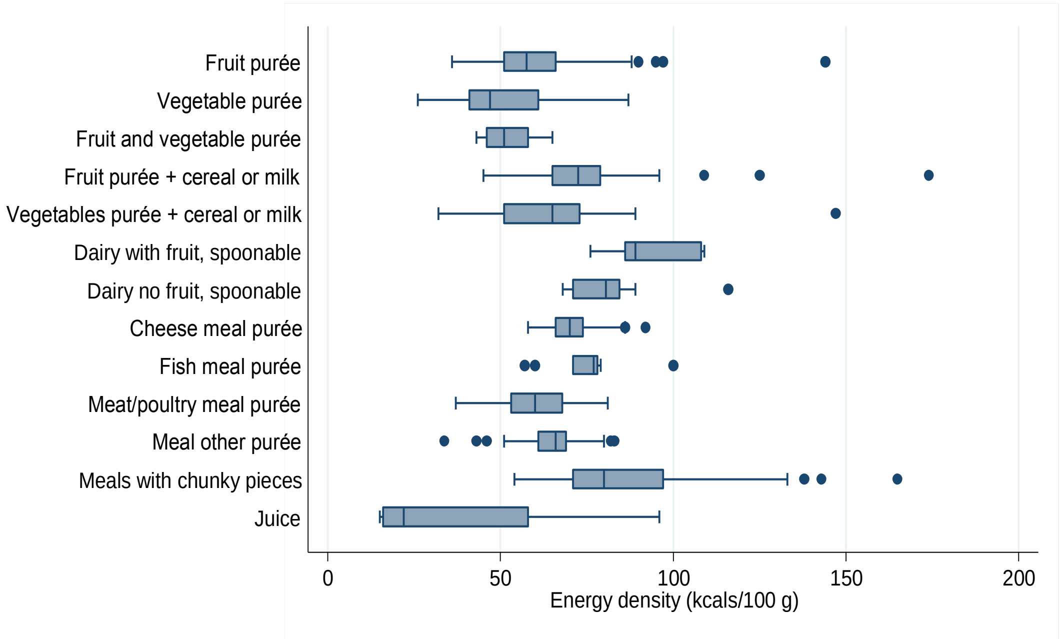

[a] Box plot explanation for Fig. 1–24: for each box, the line inside the box represents the median, the top and bottom of the box are first and third quartiles (Q1 and Q3) respectively, representing the interquartile range (IQR) and the middle 50% of values for each food category. The vertical lines (whiskers) and the dots (outliers) show the minimum and maximum values; data are presented as outliers if they are more than 1.5 x IQR below Q1 or above Q3.

Fig. 2. Box plots of energy density (kcals/100 g) of CACFs targeted for 4–36-month-olds on the Danish market in 2016/2017

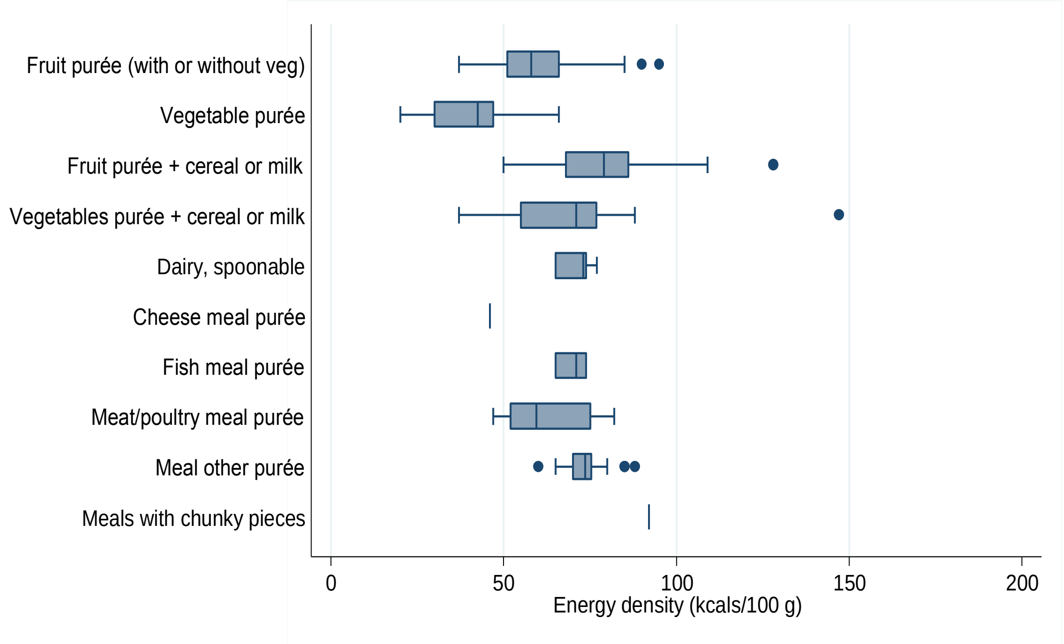

Fig. 3. Box plots of energy density (kcals/100 g) of CACFs targeted for 4–36-month-olds on the Spanish market in 2017

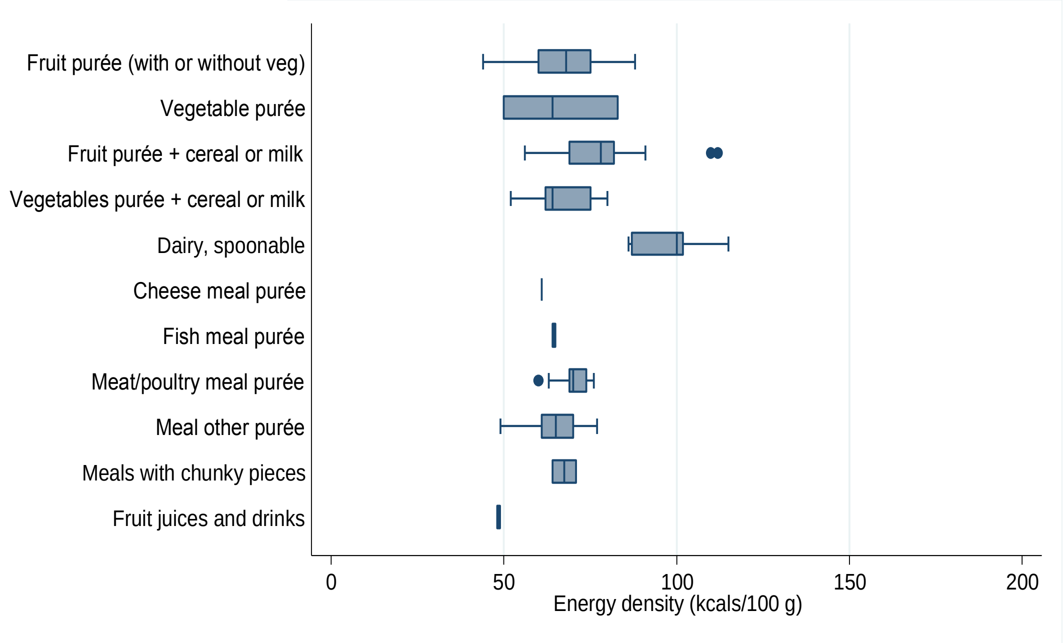

Table 5. Summary of energy density of infant complementary foods commercially available in the United Kingdom (N = 768) in 2016/2017, showing the percentage of products under or exceeding different energy density thresholds

	Marketed in United Kingdom for infants 4–36 months									
		Percentage (%) of products with energy densities under different kcal/100 g thresholds				Energy density kcal/100 g				
	**n**	**50 kcal**	**60 kcal**	**70 kcal**	**80 kcal**	**Mean**	**SD**	**Min**	**Max**	**Median**
**Soft–wet spoonable**										
Fruit purée	124	18[a]	57[a]	82	94	**60**	14	36	144	57.5
Vegetable purée	33	55	70	91	97	**50**	15	26	87	47
Fruit and vegetable blended purée	16	38[a]	81[a]	100	100	**52**	7	43	65	51
Fruit purée with cereal/milk	54	6[a]	13[a]	39	76	**74**	20	45	174	72.5
Vegetable purée with cereal/milk	43	21[a]	40[a]	60	79	**65**	20	32	147	65
Dairy with/without fruit	10	0	0	0	10	**93**	12	76	109	89
Dairy no fruit, spoonable	16	0	0	13	44	**81**	12	68	116	80.5
Cheese meal purée	18	0	6[a]	44	89	**71**	8	58	92	70
Fish meal purée	9	0	11[a]	22	89	**75**	12	57	100	77
Meat meal purée	37	22[a]	49[a]	81	95	**60**	11	37	81	60
Meal other purée	81	4[a]	19[a]	78	96	**65**	8	34	83	66
**Meal with chunky pieces**	89	0	7	20	49	**86**	22	54	165	80
**Fruit juice**[b]	13	69	77	85	85	**38**	30	15	96	22
		Percentage (%) of products with energy densities above different kcal/100 g thresholds				Energy density kcal/100 g				
	**n**	**350 kcal**	**400 kcal**	**425 kcal**	**450 kcal**	**Mean**	**SD**	**Min**	**Max**	**Median**
**Dry, powdered and instant**										
Cereal, dry/instant	32	97	55	36	0	**405**	27	344	440	410
Cereal with fruit, instant	46	98	50	13	0	**395**	25	340	448	400.5
**Dry finger foods and snacks**										
Savoury finger food	80	99	71	56	17	**423**	38	249	519	429
Sweet finger food and confectionery and rusks[b]	57	74	56	14	2	**388**	45	266	455	405
Fruit pieces/dried	9	44	44	44	44	**393**	99	296	497	343

[a] Blue text indicates the percentage of products that do not reach the minimum energy thresholds proposed for that food category.

[b] NPM recommends all juice, all confectionery and sweet snacks > 15% energy from sugar are not marketed for infants and young children < 36 months of age.

Table 6. Summary of energy density of infant complementary foods commercially available in Denmark (N = 319) in 2016/2017, showing the percentage of products under or exceeding different energy density thresholds

	Marketed in Denmark for infants 4–36 months									
		Percentage (%) of products with energy densities under different kcal/100 g thresholds				Energy density kcal/100 g				
	n	50 kcal	60 kcal	70 kcal	80 kcal	Mean	SD	Min	Max	Median
**Soft–wet spoonable**										
Fruit purée (with/without vegetables)	97	14[a]	58[a]	80	**96**	60	11	37	95	58
Vegetable purée	10	80	90	100	**100**	41	14	20	66	43
Fruit purée with cereal/milk	47	0	11[a]	32	**51**	78	15	50	128	79
Vegetable purée with cereal/milk	21	24[a]	33[a]	43	**76**	69	24	37	147	71
Dairy with/without fruit	5	0	0	40	**100**	71	5	65	77	73
Cheese meal purée	1	100[a]	100[a]	100	**100**	46	–	46	46	46
Fish meal purée	3	0	0	33	**100**	70	5	65	74	71
Meat meal purée	12	17[a]	50[a]	58	**92**	63	13	47	82	60
Meal other purée	44	0	0	23	**91**	73	5	60	88	74
**Meals with chunky pieces**	1	0	0	0	**0**	92	–	92	92	92
**Fruit juice**[b]	0	–	–	–	**–**	–	–	–	–	–
		Percentage (%) of products with energy densities above different kcal/100 g thresholds				Energy density kcal/100 g				
	n	350 kcal	400 kcal	425 kcal	450 kcal	Mean	SD	Min	Max	Median
**Dry, powdered and instant**										
Cereal, dry/instant	3	67	0	0	0	**351**	27	320	368	364
Cereal with high added protein	45	98	84	69	44	**436**	34	342	478	445
**Dry finger foods and snacks**										
Savoury finger food	15	100	73	47	0	**419**	28	366	450	422
Sweet finger food and confectionery[b]	15	100	47	13	0	**402**	23	378	448	392
Fruit pieces/dried	0	–	–	–	–	**–**	–	–	–	–

[a] Blue text indicates the percentage of products that do not reach the minimum energy thresholds proposed for that food category.
[b] NPM recommends all juice, all confectionery and sweet snacks > 15% energy from sugar are not marketed for infants and young children < 36 months of age.

Table 7. Summary of energy density of infant complementary foods commercially available in Spain (N = 241) in 2017, showing the percentage of products under or exceeding different energy density thresholds

	Marketed in Spain for infants 4–36 months									
		Percentage (%) of products with energy densities under different kcal/100 g thresholds				Energy density kcal/100 g				
	**n**	**50 kcal**	**60 kcal**	**70 kcal**	**80 kcal**	**Mean**	**SD**	**Min**	**Max**	**Median**
**Soft-wet spoonable**										
Fruit purée (with/without vegetables)	41	2[a]	24[a]	56	93	**67**	10	**44**	88	68
Vegetable purée	3	0	33	67	67	**66**	17	**50**	83	64
Fruit purée with cereal/milk	25	0	8[a]	28	56	**78**	14	**56**	112	78
Vegetable purée with cereal/milk	5	0	20[a]	60	80	**67**	11	**52**	80	64
Dairy with/without fruit	15	0	0	0	0	**97**	9	**86**	115	100
Cheese meal purée	1	0	0	100	100	**61**	–	**61**	61	61
Fish meal purée	2	0	0	100	100	**65**	1	**64**	65	65
Meat meal purée	11	0	0	36	100	**70**	5	**60**	76	70
Meal other purée	43	5[a]	23[a]	72	100	**65**	7	**49**	77	65
**Meals with chunky pieces**	2	0	0	50	100	**68**	5	**64**	71	68
**Fruit juice**[b]	2	100	100	100	100	**49**	1	**48**	49	49
		Percentage (%) of products with energy densities above different kcal/100 g thresholds				Energy density kcal/100 g				
	**n**	**350 kcal**	**400 kcal**	**425 kcal**	**450 kcal**	**Mean**	**SD**	**Min**	**Max**	**Median**
**Dry, powdered and instant**										
Cereal, dry/instant	74	100	5	0	0	383	11	352	423	382
Cereal with high added protein	8	100	50	0	0	390	14	375	404	392
**Dry finger foods and snacks**										
Savoury finger food	2	100	0	0	0	388	2	386	389	388
Sweet finger food and confectionery[b]	7	100	85	57	0	426	16	397	444	426
Fruit pieces/dried	0	–	–	–	–	–	–	–	–	–

[a] Blue text indicates the percentage of products that do not reach the minimum energy thresholds proposed for that food category.
[b] NPM recommends all juice, all confectionery and sweet snacks > 15% energy from sugar are not marketed for infants and young children < 36 months of age.

Fig. 4. Box plots of energy density (kcals/100 g) of CACFs targeted for 4–36-month-olds on the United Kingdom market in 2016/2017

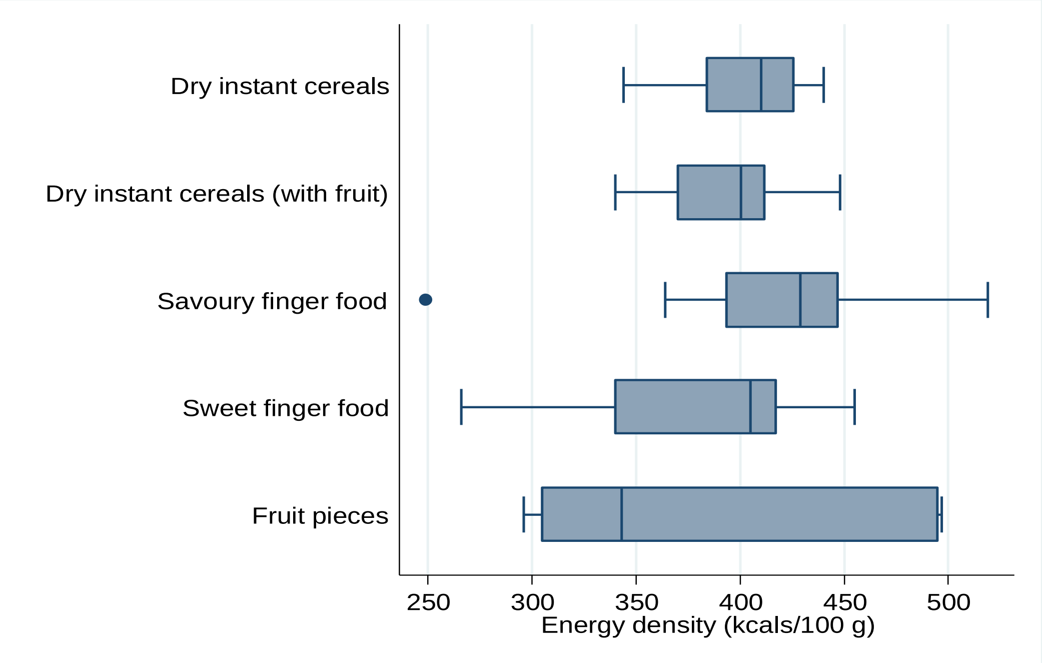

Fig. 5. Box plots of energy density (kcals/100 g) of CACFs targeted for 4–36-month-olds on the Danish market in 2016/2017

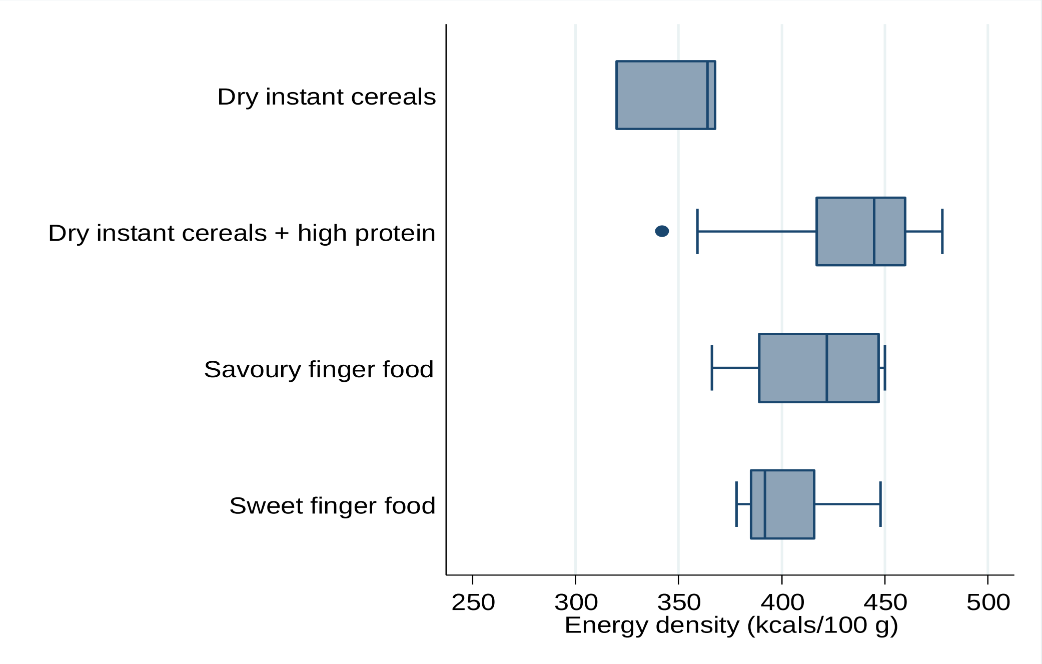

Fig. 6. Box plots of energy density (kcals/100 g) of CACFs targeted for 4–36-month-olds on the Spanish market in 2017

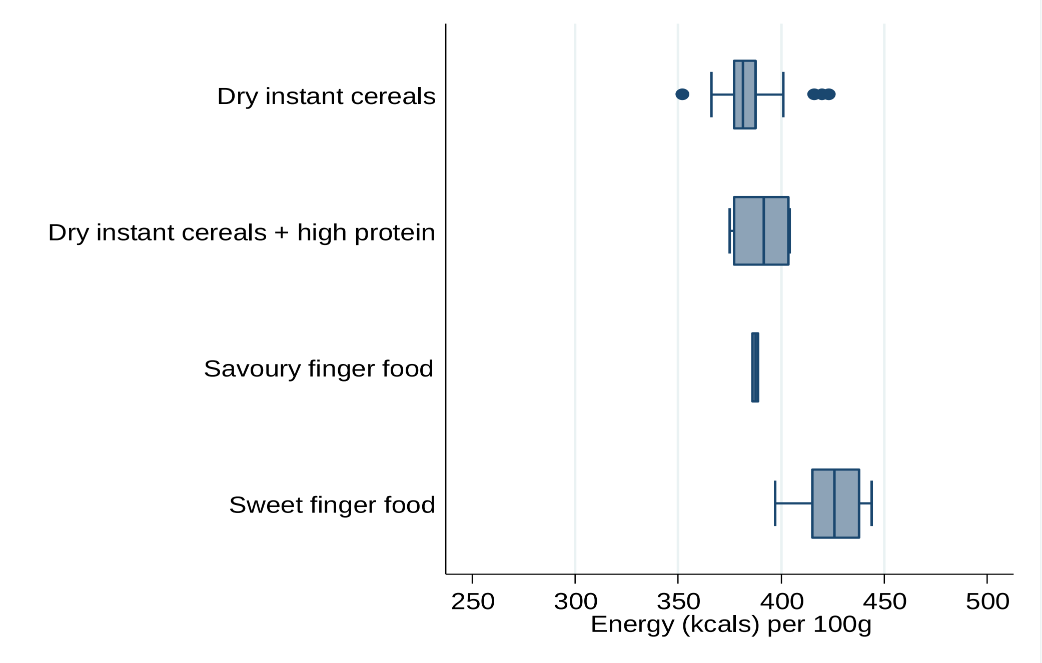

All meals with chunky/chopped pieces (typically labelled as suitable from 10 or 12 months) met the 60 kcal/100 g threshold. No threshold was set for vegetable-only purées (95% vegetables) so that manufacturers are not dissuaded from preparing purées of naturally low-energy vegetables and are not encouraged to add sweet/starchy vegetables, fruit or fats to achieve the minimum energy threshold.

With respect to dry products, the WHO Codex CAC/GL 8-1991 guidelines for formulated complementary foods suggest energy densities should be at least 4 kcal/g (400 kcal/100 g) on a dry-weight basis (which includes, but is not limited to, porridges containing cereals and ready-to-use products). Most products in the dry-cereal category in the three countries were over 400 kcal/100 g. Additionally, about 50% of sweet snacks and finger foods and 70% of savoury finger foods sold in the United Kingdom and Denmark contained more than 400 kcal/100 g; a high proportion of sweet finger foods contained over 425 kcal/100 g. Although it seems sensible to suggest maximum energy density thresholds for finger/snack foods, a suitable threshold or maximum portion size had not been included in the earlier NPM version because this required further thought in relation to individual child size/age, growth and frequency of consumption.

Total sugar

Analysis of the energy contribution from total sugars (Fig. 7–9, Table 8–10) reveals that on average, fruit-only purées (and fruit/vegetable blends examined in the United Kingdom sample) are higher in total sugars than simple vegetable purées. The mean percentage energy from total sugar in simple fruit purées in the United Kingdom, Denmark and Spain was similar, being 76%, 73% and 74% respectively (ranging up to a maximum of 98%, 100% and 91% respectively). The percentage energy from total sugar in simple vegetable purées in the

Fig. 7. Box plots of percentage energy from total sugars in CACFs targeted for 4–36-month-olds on the United Kingdom market in 2016/2017

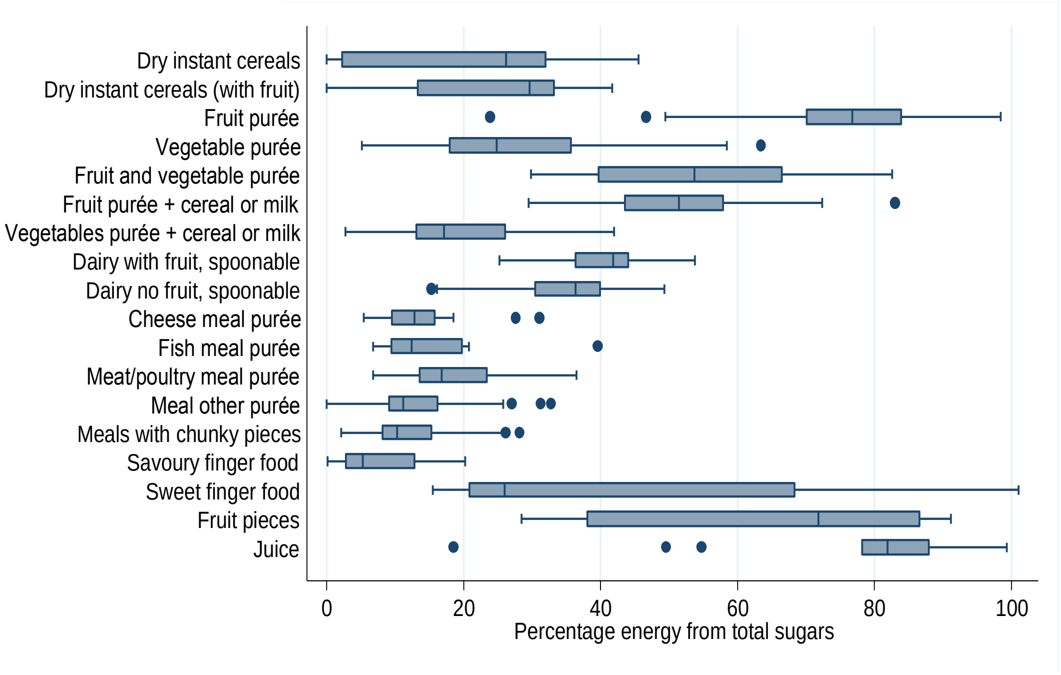

Fig. 8. Box plots of percentage energy from total sugars in CACFs targeted for 4–36-month-olds on the Danish market in 2016/2017

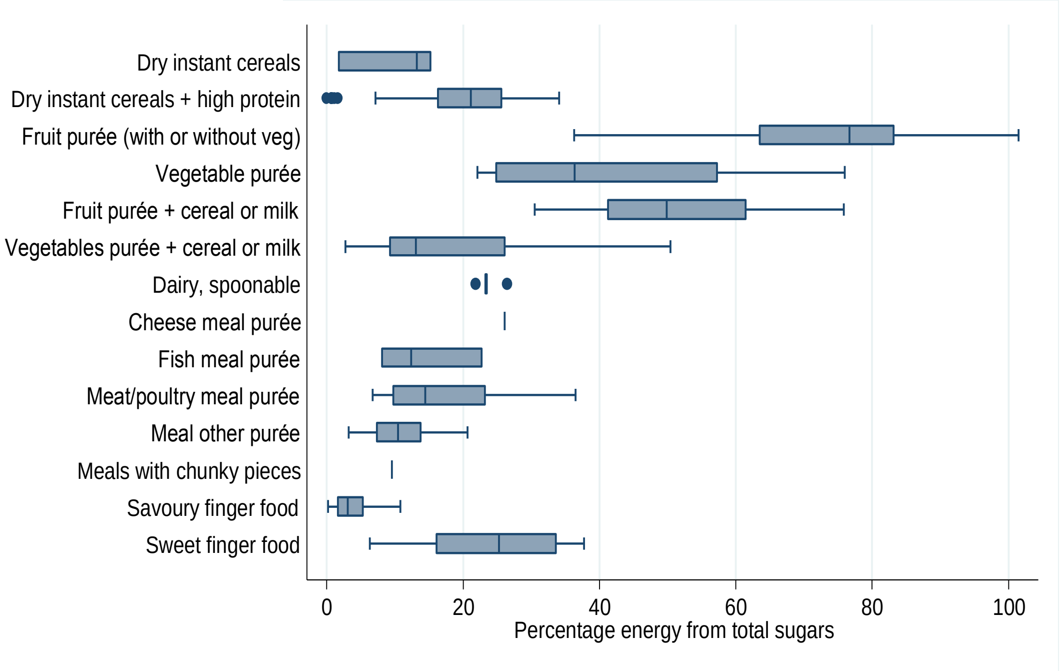

Fig. 9. Box plots of percentage energy from total sugars in CACFs targeted for 4–36-month-olds on the Spanish market in 2017

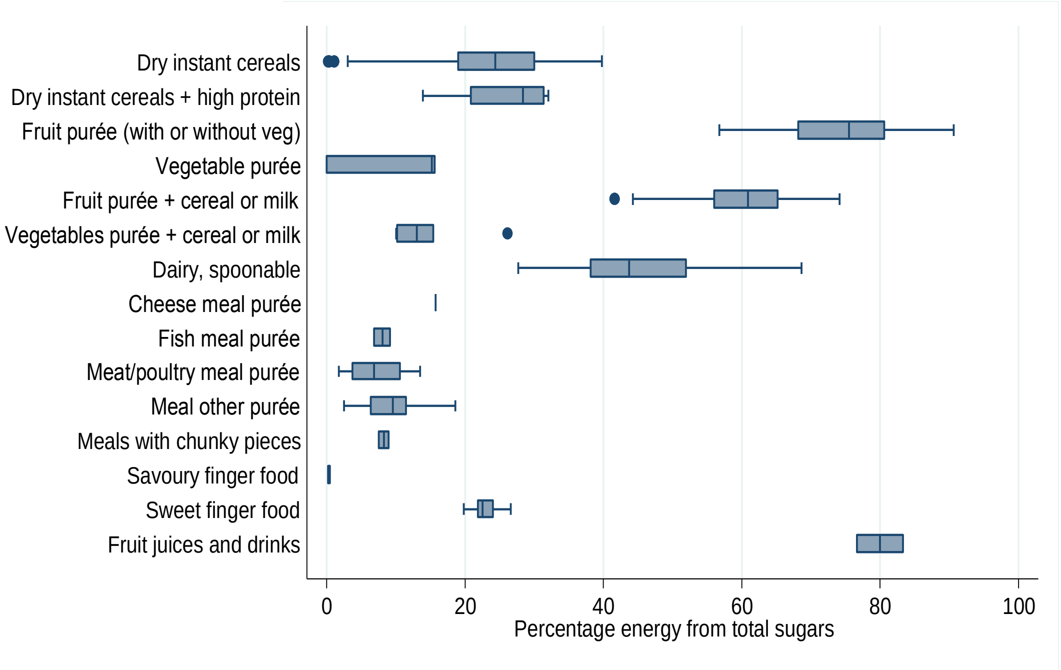

United Kingdom, Denmark and Spain was 29%, 42% and 10% respectively. Meal-type CACFs and vegetable purées with cereals and/or milk were lower in sugar than simple purées or dairy-based foods, but still derived a significant proportion of calories from total sugar. In the United Kingdom, for example, the average total-sugar content of spoonable meals with meat (named first) was 19% (minimum 7%, maximum 36%). Meal-type purées in Denmark contained over 17% of energy from total sugar (minimum 7%, maximum 36%), but levels in Spanish meal-type products were slightly lower, with a mean of 7% (minimum 2%, maximum 14%).

Results from the United Kingdom show that dry-cereal products containing fruit would be more likely to need a high-sugar indicator, as proposed in the NPM, than those that do not contain fruit (39% versus 27%). Dairy-based foods would require a front-of-pack indicator when more than 40% energy was derived from total sugar (much being from lactose, which is not considered free sugar); this indicator would apply to 60% of United Kingdom products with fruit, but only 13% without fruit. None of the five Danish dairy-based products exceeded the 40% sugar threshold, but 53% of the Spanish dairy-based products would require a high-sugar label. Any snacks with over 15% of energy from sugar would be classed as unsuitable for sale; this would include rusks, biscuits and most oat bars containing fruit currently on the market.

Added sugar

The percentage of products in each food category containing ingredients that were classed as added sugars in these analyses (sugar or sucrose, dextrose, fructose, glucose, maltose, galactose, trehalose, (any) syrup, honey, malt extract/malted barley, molasses, and also juice (other than lemon or lime juice, as they are not sweet-tasting and a small amount could be permitted in CACFs as a preservative)) are shown in Chapter 2.5, Table 24–26. The amount of added sugar was not provided on product labels. The type of product that contained added sugar and the type of sugars added varied across the three countries. About a quarter of the

products on the United Kingdom (28%) and Danish (21%) markets listed an added sugar in their ingredients, whereas about half of products (44%) on the Spanish market contained them. None of the products in the three countries listed galactose or trehalose in the ingredients.

Overall, 28% of products on the United Kingdom market in 2016/2017 contained added sugars, primarily juice, sugar or malt extract (Chapter 2.5, Table 24):

- 16% of all United Kingdom products were sweetened with a **juice** (other than lemon): 69% of sweet/confectionary finger foods, 24% of savoury finger foods, 22% of fish-meal purées, 11% of chunky meals, a large portion of the fruit purées with or without cereal/milk, and a small proportion of other product types;
- 9% of the United Kingdom products had **"sugar"** or sucrose listed in the ingredients: almost all (95%) of rusks/teething biscuits, 58% of dairy products, 15% of sweet/confectionary finger foods and 13% of dry cereals (without added protein); and
- **malt extract/malted barley** was also in the ingredients of 23% of dry cereals with added high protein, 42% of rusks/teething biscuits, 18% of sweet/confectionary finger foods and 9% of savoury finger foods.

Added lactose, which has not been classed as an added sugar, was listed in only 3% of the savoury finger foods and no other United Kingdom product. As observed in Chapter 2.5, Table 27, other sweet ingredients used in 7% of United Kingdom products are maltodextrin (labelled in 77% of dry cereals with added high protein (dried milk or whey protein) and 16% of other dry cereal products) and inulin (labelled in 37% of rusks/teething biscuits and a few other products). Oligosaccharides were also listed in dry cereals with added high protein (8%). These are also found in infant formula breast-milk substitute.

Overall, 21% of products on the Danish market in 2016/2017 in the data set contained added sugars, the main form being juice (Chapter 2.5, Table 25):

- 18% of the Danish products were sweetened with a **juice** (other than lemon): 33% of sweet/confectionary finger foods, 7% of savoury finger foods, 7% of general "other" puréed meals and a large portion of the fruit purees with or without cereal/milk; and
- **malt extract/malted barley** was used in about half of sweet/confectionary finger foods.

Lactose was not listed as an ingredient in any of the Danish products. Only 3% listed maltodextrin (in 16% of dry cereals with added high protein and 13% of sweet/confectionary finger foods).

Overall, 44% of products on the Spanish market in 2017 in the data set contained added sugars, the main types being juice, sugar, malt extract or honey (Chapter 2.5, Table 26):

- 17% of the Spanish products were sweetened with a **juice** (other than lemon): these were fruit purées without or without cereal/milk (only one other product item used juice); and
- compared to the United Kingdom and Denmark, a much larger proportion of the Spanish products (24%) were labelled with **"sugar"** (or sucrose): most (86%) of sweet/confectionary finger foods, two thirds of dairy products, half of fruit purées with cereal/milk, and about a third of dry cereal products;

- **honey and malt extract** was also listed in a substantial proportion of dry cereal, dairy products and sweet/confectionary finger foods (and malt extract in fruit purées with cereal/milk); and
- some sweet/confectionary finger foods also contained **dextrose, maltose or syrup.**

A small number of Spanish products had lactose listed in the ingredients (dairy and puréed meals). Other sweet ingredients used in a much higher proportion (30%) (Chapter 2.5, Table 29) than in the United Kingdom or Denmark are oligosaccharides, dextrinomaltosa, maltodextrin and inulin, which are also found in breast-milk substitutes. These were mainly listed as ingredients in dry cereal products: all of the eight products with added high protein and 33% (of the 74) other dry cereal products contained one of these ingredients. Oligosaccharides was also an ingredient in a puréed meal and fruit purées with milk, and maltodextrin was an ingredient in vegetable and fruit purées and dairy products (Chapter 2.5, Table 29).

Sugar content in relation to existing and proposed thresholds

The European Commission directive does not have regulations relating to total sugar, but stipulates the total carbohydrate in certain foods. It also stipulates the amount of added sugar such as sucrose, fructose, syrups and honey allowed in processed cereal-based foods, but it was not possible to establish the amount of these or the total added (or free) sugar in products, as this is not listed on packet information.

As discussed above, the existing thresholds have been criticized as being too lenient. The NPM proposes that CACFs contain no added sugar or sweetening agent. In addition, to influence the total-sugar content (for sugars that fall outside the definition of added sugars or sweetening agents), it is recommended that foods above a certain threshold should be required to indicate the percentage total energy from sugar on the front of pack.

Applying new and stricter total-sugar thresholds from the NPM to different food categories revealed that many foods currently on the market would need to be reformulated or include a front-of-pack indicator of high sugar content and display the percentage of energy from total sugar. For example, 59% and 42% of spoonable meals with meat in the United Kingdom and Denmark would need an indicator that the savoury food had greater than 15% energy from total sugar. Under the proposals in the NPM, almost all fruit purées would require a high-sugar indicator.

Table 8. Summary of total sugars[a] in infant complementary foods commercially available in the United Kingdom (N = 768) in 2016/2017, showing the percentage of products exceeding different thresholds of percentage energy from total sugar

	Marketed in United Kingdom for infants 4–36 months										
	Percentage (%) of products exceeding different thresholds of percentage energy (E) from total sugars						Percentage (%) energy from total sugar				
	**n**	**> 10% E**	**> 15% E**	**> 20% E**	**> 30% E**	**> 40% E**	**Mean**	**SD**	**Min**	**Max**	**Median**
**Dry, powdered and instant**											
Cereal, dry/instant	33	67	60	60	27[b]	6[b]	**20**	15	0	46	26
Cereal with fruit, instant	46	78	67	63	39[b]	2[b]	**24**	11	0	42	30
**Soft–wet spoonable**											
Fruit purée	124	100	100	100	99[b]	99[b]	**76**	11	24	98	77
Vegetable purée	33	94	91	64	36[b]	18[b]	**29**	15	5	63	25
Fruit and vegetable blended purée	16	100	100	100	94[b]	75[b]	**54**	16	30	83	54
Fruit purée with cereal/milk	54	100	100	100	98[b]	96[b]	**52**	10	30	83	51
Vegetable purée with cereal/milk	43	91	63	33[b]	14[b]	2[b]	**19**	9	3	42	17
Dairy with fruit, spoonable	10	100	100	100	80	60[b]	**40**	9	25	54	42
Dairy no fruit, spoonable	16	100	94	81	75	13[b]	**34**	10	15	49	36
Cheese meal purée	18	67	28[b]	11[b]	6[b]	0	**14**	7	5	31	13
Fish meal purée	9	67	33b	22[b]	11[b]	0	**16**	10	7	40	12
Meat meal purée	37	92	59[b]	35[b]	8[b]	0	**19**	7	7	36	17
Meal other purée	81	57	30[b]	11[b]	2[b]	0	**13**	6	0	33	11
**Meals with chunky pieces**											
Meal meat or fish, vegetable tray/pot	89	47	22[b]	9[b]	0	0	**12**	6	2	28	10
**Dry finger foods and snacks**											
Savoury finger food	80	31	10[b]	0	0	0	**7**	6	0	20	5
Sweet finger food and confectionery, rusks[c]	57	100	98	79	35	28	**37**	23	15	101	26
Fruit pieces/dried	9	100	100	100	78	56	**61**	27	28	91	72
**Juices and drinks**											
Fruit juice[c]	13	100	100	92	92	92	**76**	22	18	99	82

[a] Current European Commission regulations relate to the amount of added sucrose, fructose and glucose for some product types which could not be determined from packet information.
[b] Red text indicates the percentage of products that exceed total sugar thresholds proposed as high percentage sugar front-of-packet indicators for that food category.
[c] NPM recommends all juice, all confectionery and sweet snacks > 15% energy from sugar are not marketed for infants and young children < 36 months of age.

Table 9. Summary of total sugars[a] in infant complementary foods commercially available in Denmark (N = 319) in 2016/2017, showing the percentage of products exceeding proposed thresholds of percentage energy from total sugar

	Marketed in Denmark for infants 4–36 months										
		Percentage (%) of products exceeding different thresholds of percentage energy (E) from total sugars					Percentage (%) energy from total sugar				
	**n**	**> 10% E**	**> 15% E**	**> 20% E**	**> 30% E**	**> 40% E**	**Mean**	**SD**	**Min**	**Max**	**Median**
**Dry, powdered and instant**											
Cereal, dry/instant	3	67	33	0	0	0	**10**	7	2	15	13
Cereal with high added protein	43	81	81	60	2[b]	0	**19**	9	0	34	21
**Soft–wet spoonable**											
Fruit purée (with/without vegetables)	97	100	100	100	100[b]	99[b]	**73**	13	36	101	77
Vegetable purée	10	100	100	100	70[b]	40[b]	**42**	18	22	76	36
Fruit purée with cereal/milk	47	100	100	100	98[b]	79[b]	**51**	12	30	76	50
Vegetable purée with cereal/milk	21	67	33	29[b]	19[b]	10[b]	**18**	14	3	50	13
Dairy with/without fruit	5	100	100	100	0	0	**24**	2	22	26	23
Cheese meal purée	1	100	100[b]	100[b]	0	0	**26**	–	26	26	26
Fish meal purée	3	67	33[b]	33[b]	0	0	**14**	8	8	23	12
Meat meal purée	12	67	42[b]	33[b]	8[b]	0	**17**	9	7	36	14
Meal other purée	44	50	14[b]	2[b]	0	0	**11**	4	3	21	10
**Meals with chunky pieces**											
Meal meat or fish, vegetable tray/pot	1	0	0	0	0	0	**10**	-	10	10	10
**Dry finger foods and snacks**											
Savoury finger food	15	7	0	0	0	0	**4**	3	0	11	3
Sweet finger food and confectionery[c]	15	87	87	67	33	0	**24**	10	6	38	25
Fruit pieces/dried	0	–	–	–	–	–	**–**	–	–	–	–
**Juices and drinks**											
Fruit juice[c]	0	–	–	–	–	–	**–**	–	–	–	–

[a] Current European Commission regulations relate to the amount of added sucrose, fructose and glucose for some product types which could not be determined from packet information.
[b] Red text indicates the percentage of products that exceed total sugar thresholds proposed as high percentage sugar front-of-packet indicators for that food category.
[c] NPM recommends all juice, all confectionery and sweet snacks > 15% energy from sugar are not marketed for infants and young children < 36 months of age.

Table 10. Summary of total sugars[a] in infant complementary foods commercially available in Spain (N = 241) in 2017, showing the percentage of products exceeding proposed thresholds of percentage energy from total sugar

	Marketed in Spain for infants 4–36 months										
		Percentage (%) of products exceeding different thresholds of percentage energy (E) from total sugars					Percentage (%) energy from total sugar				
	**n**	**> 10% E**	**> 15% E**	**> 20% E**	**> 30% E**	**> 40% E**	**Mean**	**SD**	**Min**	**Max**	**Median**
**Dry, powdered and instant**											
Cereal, dry/instant	74	85	79	73	23[b]	0	**23**	10	0	40	24
Cereal with high added protein	8	100	87	75	38[b]	0	**26**	7	14	32	28
**Soft–wet spoonable**											
Fruit purée (with/without vegetables)	41	100	100	100	100[b]	100[b]	**74**	8	57	91	75
Vegetable purée	3	67	33	0	0	0	**10**	9	0	16	15
Fruit purée with cereal/milk	25	100	100	100	100[b]	100[b]	**60**	9	42	74	61
Vegetable purée with cereal/milk	5	60	40	20[b]	0	0	**15**	7	10	26	13
Dairy with/without fruit	15	100	100	100	87	53[b]	**45**	12	28	69	44
Cheese meal purée	1	100	100[b]	0	0	0	**16**	-	16	16	16
Fish meal purée	2	0	0	0	0	0	**8**	2	7	9	8
Meat meal purée	11	27	0	0	0	0	**7**	4	2	14	7
Meal other purée	43	45	5[b]	0	0	0	**9**	4	3	19	10
**Meals with chunky pieces**											
Meal meat or fish, vegetable tray/pot	2	0	0	0	0	0	**8**	1	8	9	8
**Dry finger foods and snacks**											
Savoury finger food	2	0	0	0	0	0	**0**	0	0	1	0
Sweet finger food and confectionery[c]	7	100	100	85	0	0	**23**	2	20	27	23
Fruit pieces/dried	0	–	–	–	–	–	**–**	–	–	–	–
**Juices and drinks**											
Fruit juice[c]	2	100	100	100	100	100	**80**	5	77	83	80

[a] Current European Commission regulations relate to the amount of added sucrose, fructose and glucose for some product types which could not be determined from packet information.
[b] Red text indicates the percentage of products that exceed total sugar thresholds proposed as high percentage sugar front-of-packet indicators for that food category.
[c] NPM recommends all juice, all confectionery and sweet snacks > 15% energy from sugar are not marketed for infants and young children < 36 months of age.

Total fat

There is a high degree of variation in terms of total fat content of foods in different categories (Fig. 10–12, Table 11–13). Total fat content of fruit purées was minimal. Simple vegetable purées in the United Kingdom and Denmark had on average only 14% and 15% energy from total fat respectively, but the mean fat content of the three Spanish vegetable purées in the sample was 25%, and these had olive oil content promoted on the packs. In dairy and meal-type products, the mean total fat content was between 23% and 40% of total energy, but some United Kingdom meals had below 10% energy from fat.

Fat content in relation to existing and proposed thresholds

The WHO Codex CAC/GL 8-1991 states the incorporation of fats and/or oils in formulated complementary foods serves to increase the energy density and the amount of essential fatty acids, and reduce total volume of food consumed. At least 20% of energy derived from fat is desirable. WHO 2003 guiding principles for complementary feeding mention 30–45% energy from fat (Annex 3). Most CACFs were within the current provisions of the European Commission directive for fat (less than 4.5 g/100 kcal total fat, which is roughly equal to less than 40% energy from total fat for most products types).

Fig. 10. Box plots of percentage energy from total fat in CACFs targeted for 4–36-month-olds on the United Kingdom market in 2016/2017

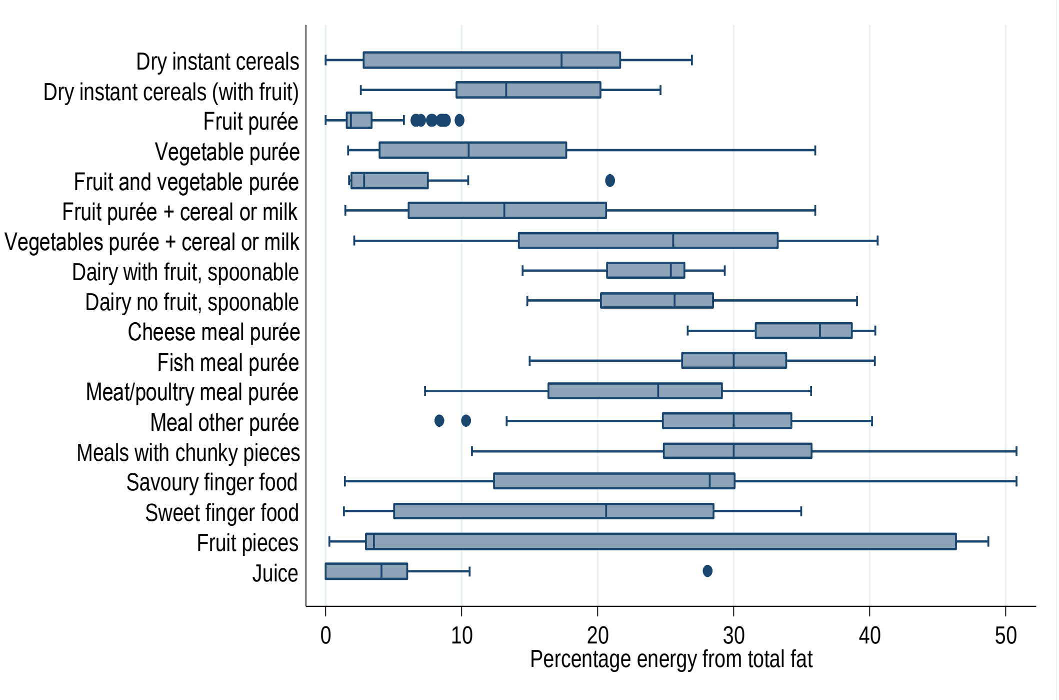

Fig. 11. Box plots of percentage energy from total fat in CACFs targeted for 4–36-month-olds on the Danish market in 2016/2017

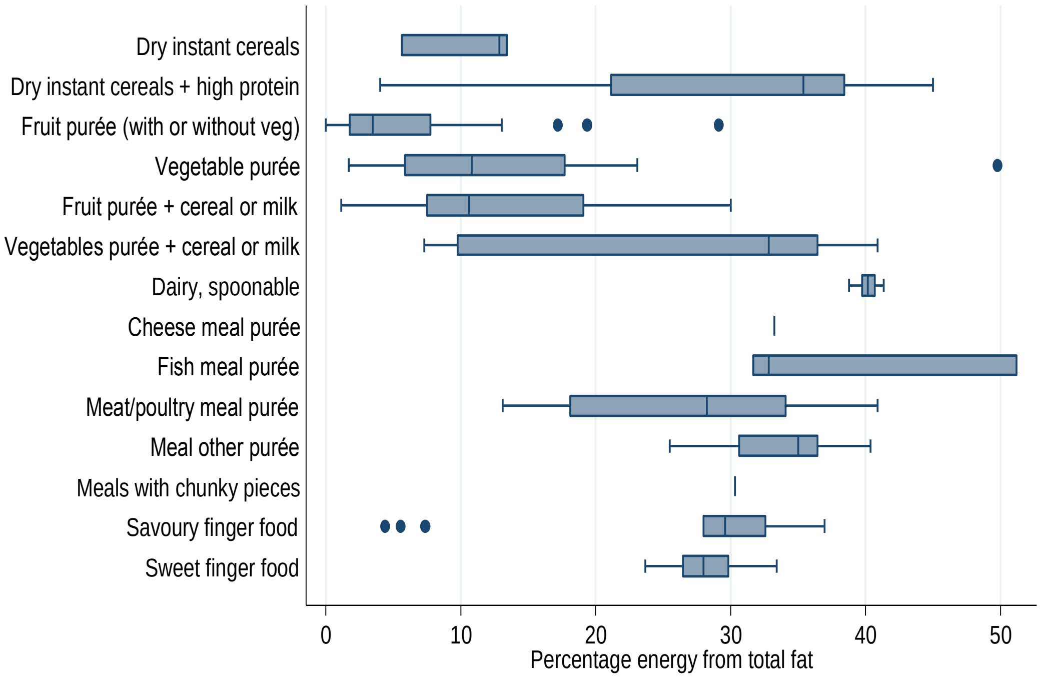

Fig. 12. Box plots of percentage energy from total fat in CACFs targeted for 4–36-month-olds on the Spanish market in 2017

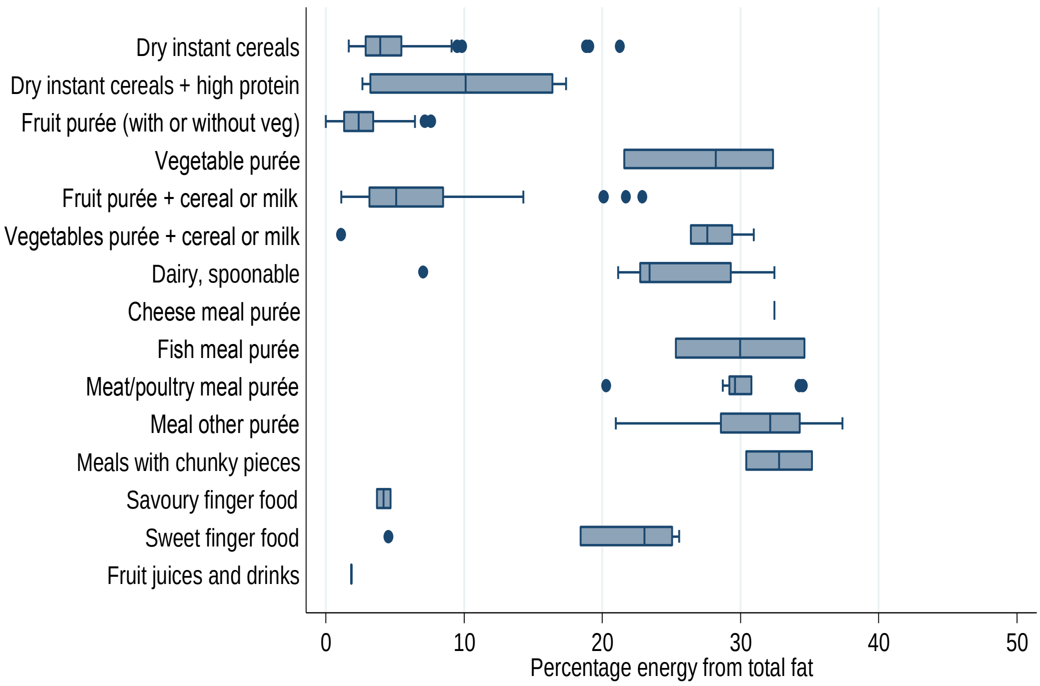

Table 11. Summary of total fats in infant complementary foods commercially available in the United Kingdom (N = 768) in 2016/2017, showing the percentage of products exceeding different thresholds of percentage energy from total fat

	Marketed in United Kingdom for infants 4–36 months									
	Percentage (%) of products exceeding different thresholds of percentage energy (E) from total fat					Percentage (%) energy from total fat				
	n	> 10% E	> 20% E	> 30% E	> 40% E	Mean	SD	Min	Max	Median
**Dry, powdered and instant**										
Cereal, dry/instant	33	54	48	0	0	**14**	10	0	27	17
Cereal with fruit, instant	46	74	28	0	0	**14**	7	3	25	13
**Soft–wet spoonable**										
Fruit purée	124	0	0	0	0	**3**	2	0	10	2
Vegetable purée	33	52	21	15	0	**14**	11	2	36	11
Fruit and vegetable blended purée	16	12	6	0	0	**5**	5	2	21	3
Fruit purée with cereal/milk	54	59	26	7	0	**14**	10	1	36	13
Vegetable purée with cereal/milk	43	79	65	37	2[a]	**24**	11	2	41	26
Dairy with fruit, spoonable	10	100	80	0	0	**24**	4	14	29	25
Dairy no fruit, spoonable	16	100	87	19	0	**25**	6	15	39	26
Cheese meal purée[b]	18	100	100	83	0	**35**	4	27	40	36
Fish meal purée	9	100	88	44	0	**29**	9	15	40	30
Meat meal purée[b]	37	97	62	22	0	**23**	7	7	36	24
Meal other purée	81	97	84	48	0	**29**	7	8	40	30
**Meals with chunky pieces**										
Meal meat or fish, vegetable tray/pot[b]	89	100	88	42	15[a]	**30**	9	11	51	30
**Dry finger foods and snacks**										
Savoury finger food	80	75	69	21	2[a]	**23**	12	1	51	28
Sweet finger food and confectionery, rusks[c]	57	74	53	21	0	**19**	11	1	35	21
Fruit pieces/dried	9	44	44	44	44[a]	**22**	24	0	49	4
**Juices and drinks**										
Fruit juice[c]	13	15	7	0	0	5	8	0	28	4

[a] Red text indicates the percentage of products in each category that exceed current European Commission thresholds for fat content (> 40%) of food (equivalent to 4.5 g/100 kcal).
[b] Meals with meat or cheese mentioned first in the name of a product have a higher maximum threshold regulation of about 50% energy from total fat (6 g/100 kcal), though these are not separated from the latter category in the analyses.
[c] NPM recommends all juice, all confectionery and sweet snacks > 15% energy from sugar are not marketed for infants and young children up to 36 months.

Table 12. Summary of total fats in infant complementary foods commercially available in Denmark (N = 319) in 2016/2017, showing the percentage of products exceeding different thresholds of percentage energy from total fat

	Marketed in Denmark for infants 4–36 months									
		Percentage (%) of products exceeding different thresholds of percentage energy (E) from total fat				Percentage (%) energy from total fat				
	**n**	**> 10% E**	**> 20% E**	**> 30% E**	**> 40% E**	**Mean**	**SD**	**Min**	**Max**	**Median**
**Dry, powdered and instant**										
Cereal, dry/instant	3	67	0	0	0	**11**	4	6	13	13
Cereal with high added protein	45	87	76	60	5[a]	**29**	12	4	45	35
**Soft–wet spoonable**										
Fruit purée (with/ without vegetables)	97	7	1	0	0	**5**	5	0	29	3
Vegetable purée	10	50	20	10	10[a]	**15**	14	2	50	11
Fruit purée with cereal/milk	47	51	19	0	0%	**13**	7	1	30	11
Vegetable purée with cereal/milk	21	71	52	52	10[a]	**25**	13	7	41	33
Dairy with/without fruit	5	100	100	100	40[a]	**40**	1	39	41	40
Cheese meal purée[b]	1	100	100	100	0	**33**	-	33	33	33
Fish meal purée	3	100	100	100	33[a]	**39**	11	32	51	33
Meat meal purée[b]	12	100	67	42	8[a]	**27**	9	13	41	28
Meal other purée	44	100	100	75	0	**34**	4	26	40	35
**Meals with chunky pieces**										
Meal meat or fish, vegetable tray/pot[b]	1	100	100	0	0	**30**	-	30	30	30
**Dry finger foods and snacks**										
Savoury finger food	15	80	80	40	0	**26**	11	4	37	30
Sweet finger food and confectionery[c]	15	100	100	20	0	**28**	3	24	33	28
Fruit pieces/dried	0	–	–	–	–	**–**	–	–	–	–
**Juices and drinks**										
Fruit juice[c]	0	–	–	–	–	**–**	–	–	–	–

[a] Red text indicates the percentage of products in each category that exceed current European Commission thresholds for fat content (> 40%) of food (equivalent to 4.5 g/100 kcal).
[b] Meals with meat or cheese mentioned first in the name of a product have a higher maximum threshold regulation of about 50% energy from total fat (6 g/100 kcal), though these are not separated from the latter category in the analyses.
[c] NPM recommends all juice, all confectionery and sweet snacks > 15% energy from sugar are not marketed for infants and young children up to 36 months.

Table 13. Summary of total fats in infant complementary foods commercially available in Spain (N = 241) in 2017, showing the percentage of products exceeding different thresholds of percentage energy from total fat[a]

	Marketed in Spain for infants 4–36 months									
		Percentage (%) of products exceeding different thresholds of percentage energy (E) from total fat				Percentage (%) energy from total fat				
	**n**	**> 10% E**	**> 20% E**	**> 30% E**	**> 40% E**	**Mean**	**SD**	**Min**	**Max**	**Median**
**Dry, powdered and instant**										
Cereal, dry/instant	74	4	1	0	0	5	4	2	21	4
Cereal with high added protein	8	50	0	0	0	10	7	3	17	10
**Soft–wet spoonable**										
Fruit purée (with/without vegetables)	41	0	0	0	0	3	2	0	8	2
Vegetable purée	3	100	100	33	0	27	5	22	32	28
Fruit purée with cereal/milk	25	24	8	0	0	7	6	1	23	5
Vegetable purée with cereal/milk	5	80	80	20	0	23	12	1	31	28
Dairy with/without fruit	15	87	87	7	0	24	8	7	32	23
Cheese meal purée[b]	1	100	100	100	0	32	-	32	32	32
Fish meal purée	2	100	100	50	0	30	7	25	35	30
Meat meal purée[b]	11	100	90	27	0	30	4	20	35	30
Meal other purée	43	100	100	63	0	31	4	21	37	32
**Meals with chunky pieces**										
Meal meat or fish, vegetable tray/pot[b]	2	100	100	50	0	33	3	30	35	33
**Dry finger foods and snacks**										
Savoury finger food	2	0	0	0	0	4	1	4	5	4
Sweet finger food and confectionery[c]	7	86	71	0	0	20	7	5	26	23
Fruit pieces/dried	0	–	–	–	–	–	–	–	–	–
**Juices and drinks**										
Fruit juice[c]	2	0	0	0	0	2	0	2	2	2

[a] No products exceed current European Commission thresholds (> 40%) for fat content of food (equivalent to 4.5 g/100 kcal).
[b] Meals with meat or cheese mentioned first in the name of a product have a higher maximum threshold regulation of about 50% energy from total fat (6 g/100 kcal), though these are not separated from the latter category in the analyses.
[c] NPM recommends all juice, all confectionery and sweet snacks > 15% energy from sugar are not marketed for infants and young children up to 36 months.

Meals with meat or cheese mentioned first in the name of a product have a higher maximum threshold regulation of about 50% energy from total fat (that is, 6 g/100 kcal), though these are not separated from the latter category in the analyses.

A small number of dried fruit snacks (nine) was identified in the United Kingdom, and four exceeded the 40% total-fat threshold. Two per cent of savoury snack foods, 15% of tray-type meals and 2% of vegetable-based purées with cereals or milk exceeded the existing threshold.

In Denmark, 4% of dry cereals with an added protein source (such as dried milk or whey), 10% of vegetable-only purées, 10% of vegetable purées with cereal/oil, two of the five dairy-based foods, one of the three meals with fish and 8% of puréed meals with meat exceeded the 40% fat threshold. None of the Spanish products in this sample exceeded the threshold.

There are no suggested changes to total-fat thresholds in the NPM; reducing the threshold to below 40% was considered unsuitable because infants and young children require more fat for growth than that advised for adult consumption.

Saturated fat

The mean saturated fat content for most of the product categories in the three countries was less than or equal to 10% energy from saturated fat (Fig. 13–15, Table 14–16). The saturated fat content of dairy-based foods in the United Kingdom, Denmark and Spain was 15%, 10% and 14% respectively and puréed meals with cheese in the name had higher levels: 19%, 16% and 19% respectively.

Some other meal-type purée products contained relatively high levels of energy from saturated fat, but the maximum recorded values were in the categories for fruit/vegetable purées with cereal/milk (31%, 34% and 29% in the United Kingdom, Denmark and Spain respectively).

Saturated fat content in relation to existing and proposed thresholds

There are no provisions in the European Commission directive or Codex standards for saturated fat, and there is apparently no evidence that the levels of saturated fat in infant foods has a significant negative effect on children's health.

No saturated fat thresholds for the NPM have been proposed, but if a maximum level of 10% energy from saturated fat was introduced, this would affect almost all of the cheese meals and most in the dairy category (especially in the United Kingdom), and many in the other meal categories. Many sweet snack foods had over 10% of energy from saturated fat, but most of these would not be suitable for sale because of the proposed total sugar threshold.

Fig. 13. Box plots of percentage energy from saturated fat in CACFs targeted for 4–36-month-olds on the United Kingdom market in 2016/2017

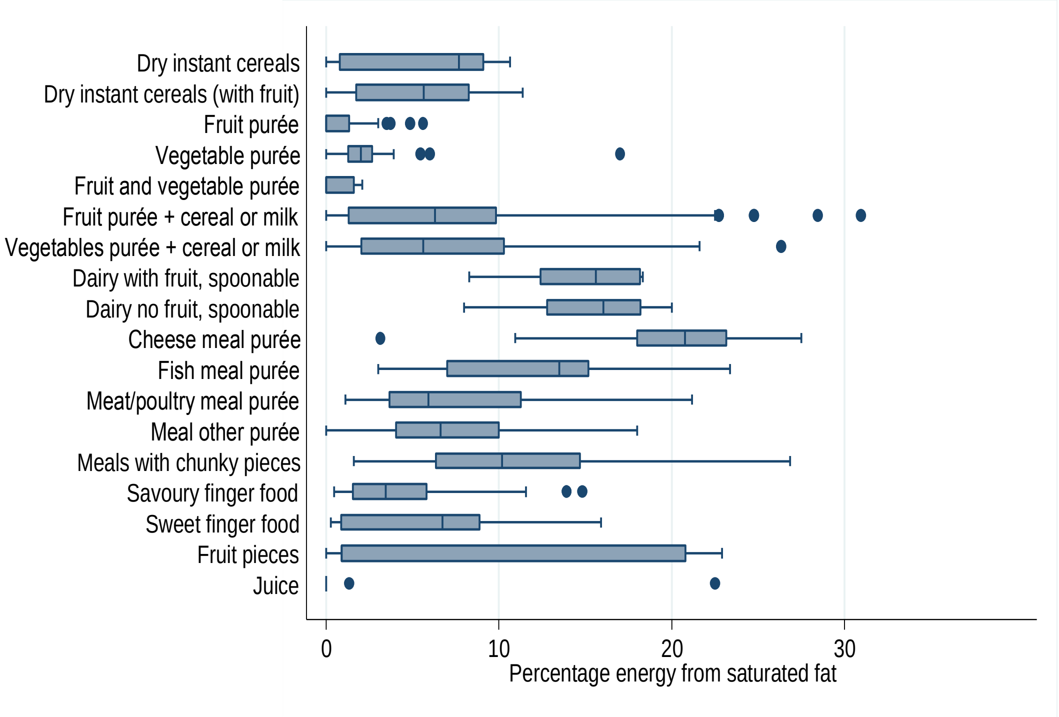

Fig. 14. Box plots of percentage energy from saturated fat in CACFs targeted for 4–36-month-olds on the Danish market in 2016/2017

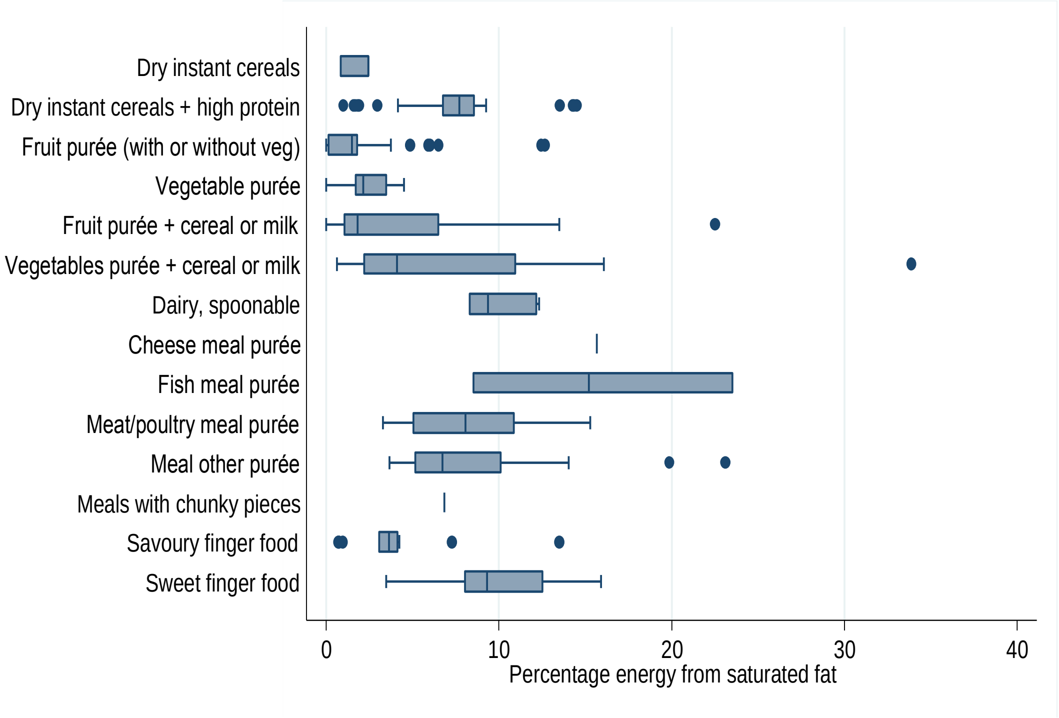

Fig. 15. Box plots of percentage energy from saturated fat in CACFs targeted for 4–36-month-olds on the Spanish market in 2017

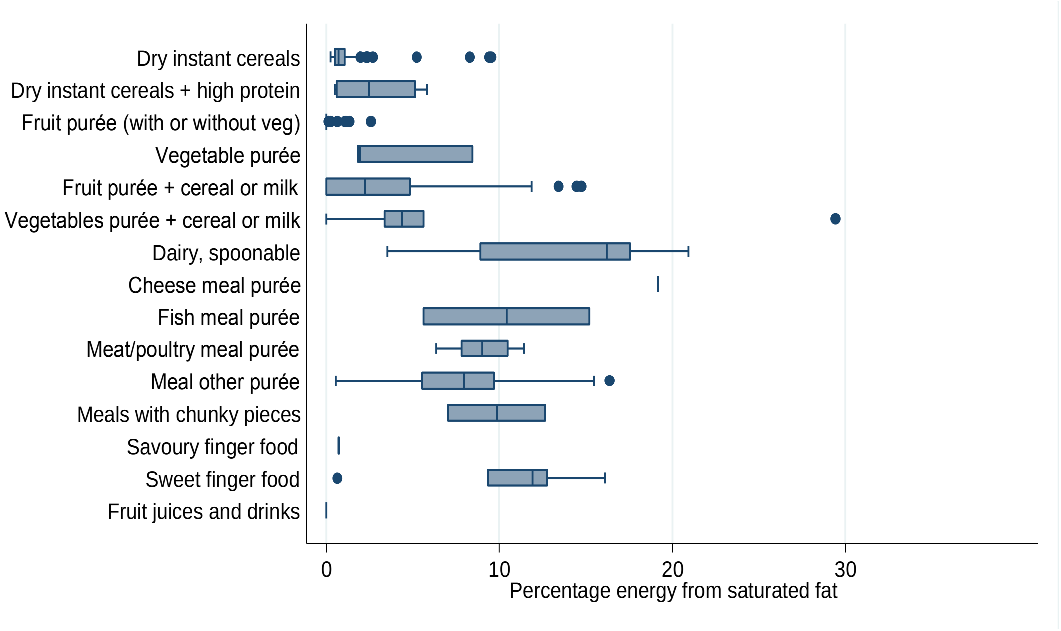

Protein

Protein content in relation to existing and proposed thresholds

Current European Commission regulations stipulate that 4 g/100 kcal of total protein must be from the protein listed in the product name (if the protein source is the first-named food) or otherwise 3 g/100 kcal of total protein in a product where the protein source is not listed first in the product name. Fig. 16–18 show that most, though not all, of the United Kingdom, Danish and Spanish meals contained over 4 g/100 kcal total protein. Regulations also stipulate 10% of the total weight of the product should be from the protein mentioned first in the product name, or 8% in products containing a traditional source of protein not mentioned first. As observed in Chapter 2.5, Table 30–32, a substantial number of meals do not meet these current regulations, especially when the regulations are interpreted as the first named food/ingredient rather than the first word in the name.

Meat is an important source of protein for infants and young children, but it is also an important source of micronutrients, including iron. CACFs typically have a lower protein content than homemade equivalents. The low proportion of meat and low protein density in certain products means it might be beneficial to increase threshold targets for meat, poultry, fish, offal or other traditional sources of protein in terms of grams per 100 kcal of product, and percentage protein by total weight of product. Higher protein thresholds were proposed in the earlier version of the NPM, and the low percentage of products meeting these is shown overall for each country in Table 22, Chapter 2.4 and by food category in Table 33, 36 and 39. However, given current uncertainties in the evidence on whether higher meat protein (needed to increase micronutrient intake) might cause rapid/excess weight gain, the current European Commission thresholds were retained in the draft NPM presented in Chapter 1.2.

Table 14. Summary of saturated fats in infant complementary foods commercially available in the United Kingdom (N = 768) in 2016/17, showing the percentage of products exceeding different thresholds of percentage energy from saturated fat[a]

	Marketed in United Kingdom for infants 4–36 months									
		Percentage (%) of products exceeding different thresholds of percentage energy (E) from saturated fat				Percentage energy from saturated fat				
	**n**	**> 5% E**	**> 10% E**	**> 15% E**	**> 20% E**	**Mean**	**SD**	**Min**	**Max**	**Median**
**Dry, powdered and instant**										
Cereal, dry/instant	33	55	3	0	0	5	4	0	11	8
Cereal with fruit, instant	46	52	2	0	0	5	3	0	11	6
**Soft–wet spoonable**										
Fruit purée	124	1	0	0	0	1	1	0	6	0
Vegetable purée	33	6	3	3	0	2	3	0	17	2
Fruit and vegetable blended purée	16	0	0	0	0	1	1	0	2	0
Fruit purée with cereal/milk	54	54	20	17	13	8	8	0	31	6
Vegetable purée with cereal/milk	43	51	23	16	5	7	7	0	26	6
Dairy with fruit, spoonable	10	100	90	50	0	15	3	8	18	16
Dairy no fruit, spoonable	16	100	81	56	0	15	4	8	20	16
Cheese meal purée	18	94	94	78	56	19	6	3	28	21
Fish meal purée	9	89	56	22	22	13	7	3	23	14
Meat meal purée	37	54	27	11	3	8	5	1	21	6
Meal other purée	81	57	22	1	0	7	4	0	18	7
**Meals with chunky pieces**										
Meal meat or fish, vegetable tray/pot	89	80	49	21	8	11	6	2	27	10
**Dry finger foods and snacks**										
Savoury finger food	80	29	6	0	0	4	3	0	15	3
Sweet finger food and confectionery, rusks[b]	57	54	16	2	0	6	5	0	16	7
Fruit pieces/dried	9	44	44	44	44	10	11	0	23	1
**Juices and drinks**										
Fruit juice[b]	13	8	8	8	8	2	6	0	23	0

[a] No current European Commission regulations on saturated fat.
[b] NPM recommends all juice, all confectionery and sweet snacks > 15% energy from sugar are not marketed for infants and young children up to 36 months.

Table 15. Summary of saturated fats in infant complementary foods commercially available in Denmark (N = 319) in 2016/2017, showing the percentage of products exceeding different thresholds of percentage energy from saturated fat[a]

	Marketed in Denmark for infants 4–36 months									
		Percentage (%) of products exceeding different thresholds of percentage energy (E) from saturated fat				Percentage energy from saturated fat				
	n	> 5% E	> 10% E	> 15% E	> 20% E	Mean	SD	Min	Max	Median
**Dry, powdered and instant**										
Cereal, dry/instant	3	0	0	0	0	2	1	1	2	2
Cereal with high added protein	43	81	9	0	0	7	3	1	14	8
**Soft–wet spoonable**										
Fruit purée (with/ without vegetables)	97	5	2	0	0	2	2	0	13	2
Vegetable purée	10	0	0	0	0	2	1	0	5	2
Fruit purée with cereal/milk	47	36	15	2	2	5	5	0	23	2
Vegetable purée with cereal/milk	21	43	33	10	5	7	8	1	34	4
Dairy with/without fruit	5	100	40	0	0	10	2	8	12	9
Cheese meal purée	1	100	100	100	0	16	-	16	16	16
Fish meal purée	3	100	67	33	33	16	8	9	24	15
Meat meal purée	12	75	33	0	0	8	4	3	15	8
Meal other purée	43	72	23	4	2	8	4	4	23	7
**Meals with chunky pieces**										
Meal meat or fish, vegetable tray/pot	1	100	0	0	0	7	-	7	7	7
**Dry finger foods and snacks**										
Savoury finger food	15	13	7	0	0	4	3	1	13	4
Sweet finger food and confectionery[b]	15	87	27	7	0	10	3	3	16	9
Fruit pieces/dried	0	–	–	–	–	–	–	–	–	–
**Juices and drinks**										
Fruit juice[b]	0	–	–	–	–	–	–	–	–	–

[a] No current European Commission regulations on saturated fat.
[b] NPM recommends all juice, all confectionery and sweet snacks > 15% energy from sugar are not marketed for infants and young children up to 36 months.

Table 16. Summary of saturated fats in infant complementary foods commercially available in Spain (N = 241) in 2017, showing the percentage of products exceeding different thresholds of percentage energy from saturated fat[a]

	Marketed in Spain for infants 4–36 months									
		Percentage (%) of products exceeding different thresholds of percentage energy (E) from saturated fat				Percentage energy from saturated fat				
	**n**	**> 5% E**	**> 10% E**	**> 15% E**	**> 20% E**	**Mean**	**SD**	**Min**	**Max**	**Median**
**Dry, powdered and instant**										
Cereal, dry/instant	74	5	0	0	0	1	2	0	10	1
Cereal with high added protein	8	25	0	0	0	3	2	0	6	2
**Soft-wet spoonable**										
Fruit purée (with/ without vegetables)	41	0	0	0	0	0	1	0	3	0
Vegetable purée	3	33	0	0	0	4	4	2	8	2
Fruit purée with cereal/milk	25	24	16	0	0	4	5	0	15	2
Vegetable purée with cereal/milk	5	40	20	20	20	9	12	0	29	4
Dairy with/without fruit	15	87	73	67	7	14	5	4	21	16
Cheese meal purée	1	100	100	100	0	19	-	19	19	19
Fish meal purée	2	100	50	0	0	10	7	6	15	10
Meat meal purée	11	100	27	0	0	9	2	6	11	9
Meal other purée	43	77	19	2	0	8	3	1	16	8
**Meals with chunky pieces**										
Meal meat or fish, vegetable tray/pot	2	100	50	0	0	10	4	7	13	10
**Dry finger foods and snacks**										
Savoury finger food	2	0	0	0	0	1	0	1	1	1
Sweet finger food and confectionery[b]	7	86	71	14	0	11	5	1	16	12
Fruit pieces/dried	0	–	–	–	–	–	–	–	–	–
**Juices and drinks**										
Fruit juice[b]	2	0	0	0	0	0	0	0	0	0

[a] No current European Commission regulations on saturated fat.
[b] NPM recommends all juice, all confectionery and sweet snacks > 15% energy from sugar are not marketed for infants and young children up to 36 months.

Fig. 16. Box plots of protein grams (g) per 100 kcal product in CACFs targeted for 4–36-month-olds on the United Kingdom market in 2016/2017

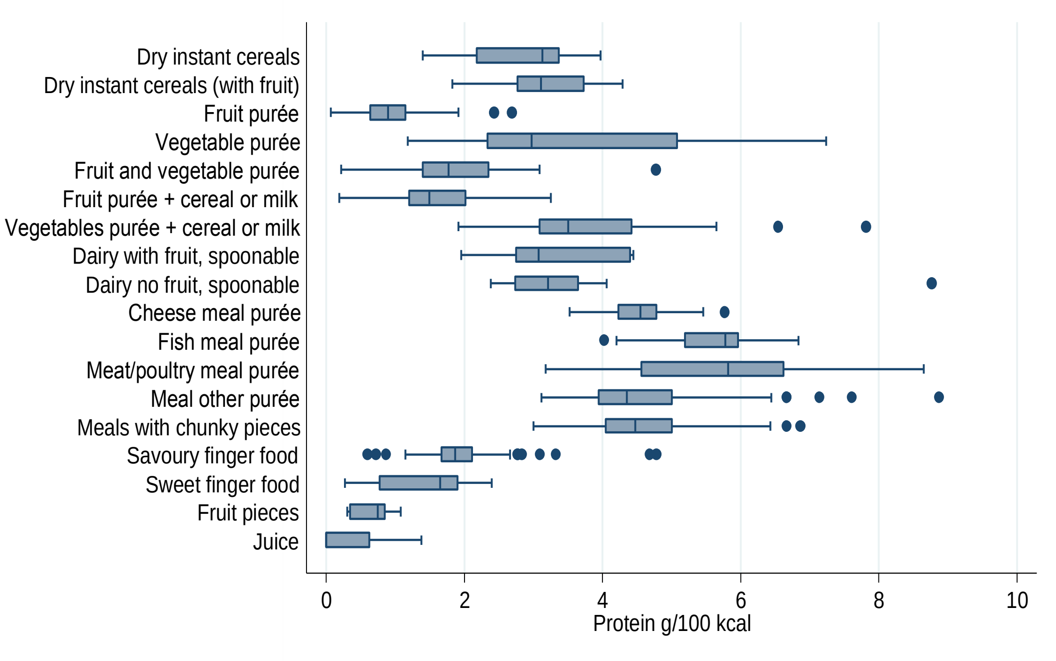

Fig. 17. Box plots of protein grams (g) per 100 kcal product in CACFs targeted for 4–36-month-olds on the Danish market in 2016/2017

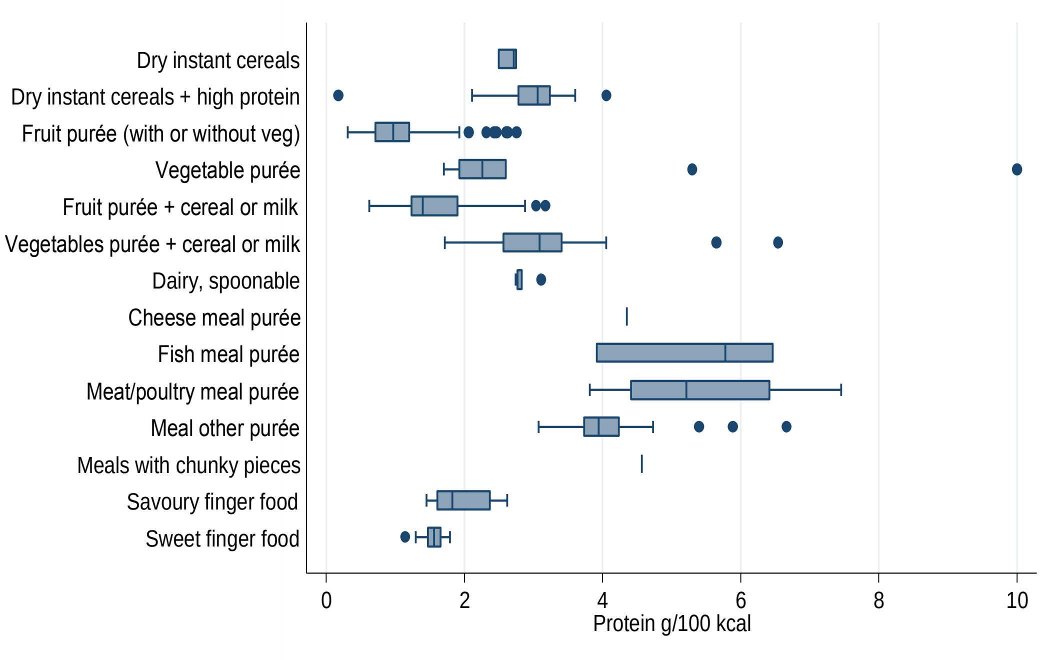

Fig. 18. Box plots of protein grams (g) per 100 kcal product in CACFs targeted for 4–36-month-olds on the Spanish market in 2017

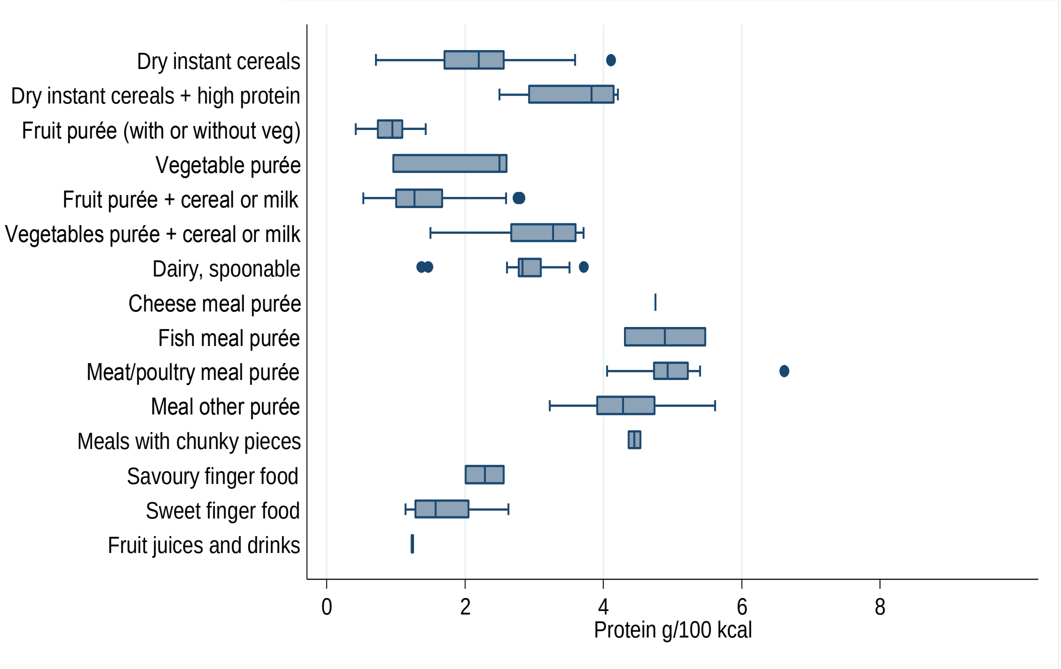

Sodium

Sodium content in relation to existing thresholds

A very small number of United Kingdom, Danish and Spanish products exceeded the existing provisions of the European Commission directive relating to sodium content of more than 200 mg sodium/100 g (Fig. 19–21) or 200 mg/100 kcal (Fig. 22–24, Table 17–19), or 100 mg/100 kcal sodium for the cereal-based products. These were dry cereals, savoury finger foods (including two United Kingdom outliers over 600 mg sodium/100 g not shown in Fig. 19), sweet finger foods in Spain (per 100 g, Fig. 21) and vegetable purées in the United Kingdom (including one outlier over 500 mg sodium/100 kcal not shown in Fig. 22).

Applying suggested sodium thresholds of 50 mg/100 kcal (except for meals with cheese, which may contain 100 mg/100 kcal) identified many products currently on the market that would require reformulation to reduce sodium levels. For example, 60% of meals with chunky/chopped pieces in the United Kingdom and the one Danish and two Spanish meals with chunky/chopped pieces in the data sets exceeded 50 mg/100 kcal. Of the vegetable purées sold in the United Kingdom, Denmark and Spain, 24%, 70% and 33% respectively contained more than 50 mg/100 g sodium. Savoury snack foods also tended to contain more sodium, and 11%, 7% and 43% of savoury snacks sold in the United Kingdom, Denmark and Spain respectively exceed the new proposed lower threshold of 50 mg/100 kcal. The European Commission directive also states that "Sodium salts may not be added to products based on fruit, nor to desserts [or] puddings except for technological purposes", but it was not possible to determine what salt had been added and whether it would have been for technological purposes.

Fig. 19. Box plots of sodium (mg) per 100 g product in CACFs targeted for 4–36-month-olds on the United Kingdom market in 2016/2017

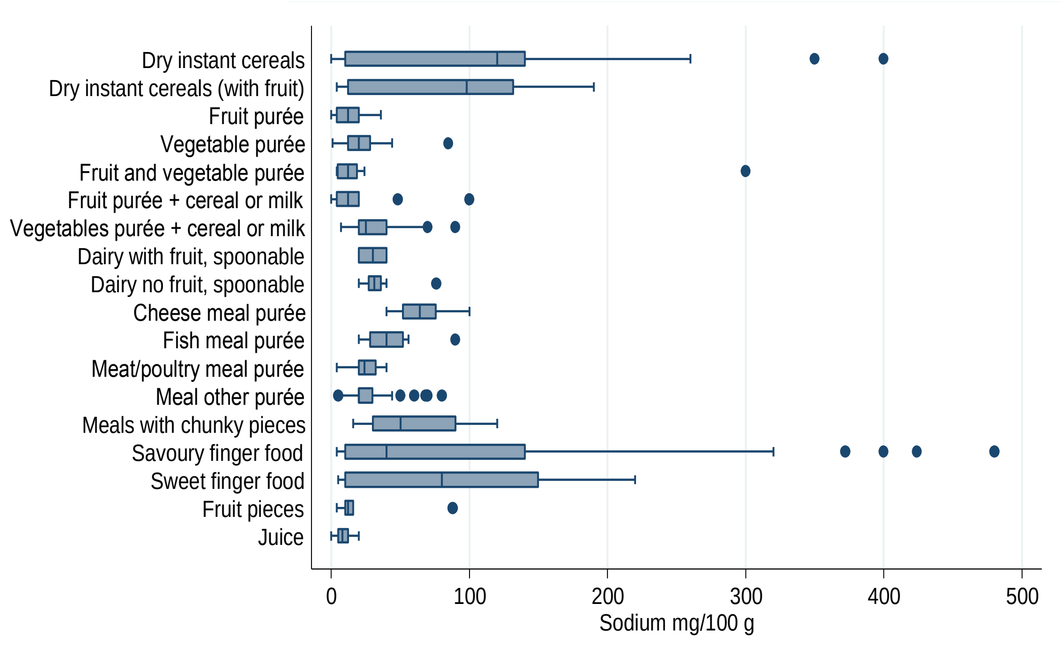

Fig. 20. Box plots of sodium (mg) per 100 g product in CACFs targeted for 4–36-month-olds on the Danish market in 2016/2017

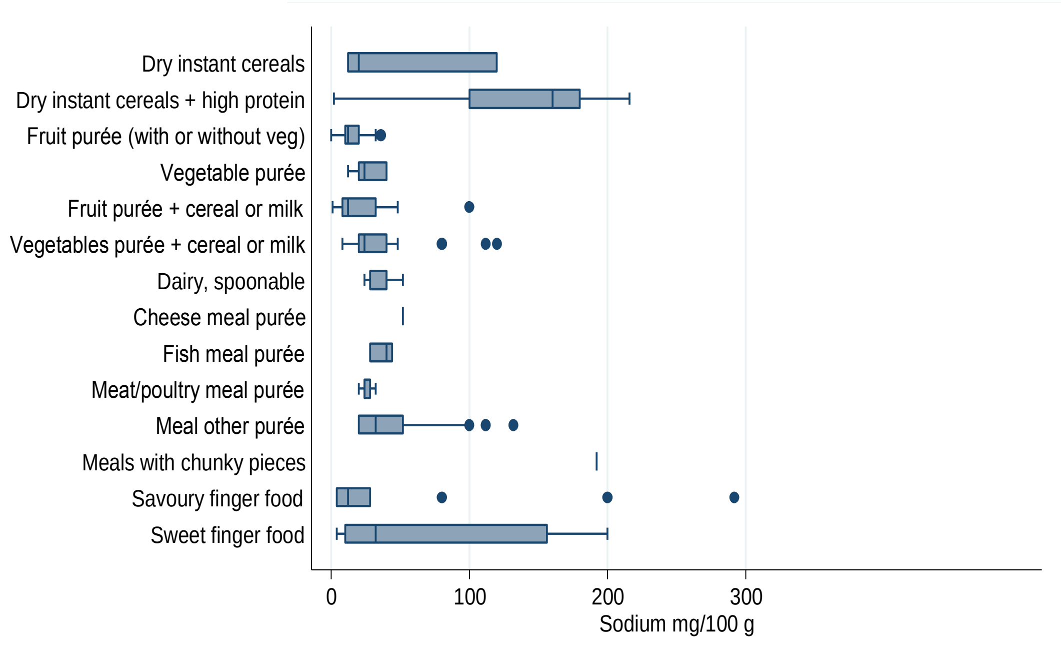

Fig. 21. Box plots of sodium (mg) per 100 g in CACFs targeted for 4–36-month-olds on the Spanish market in 2017

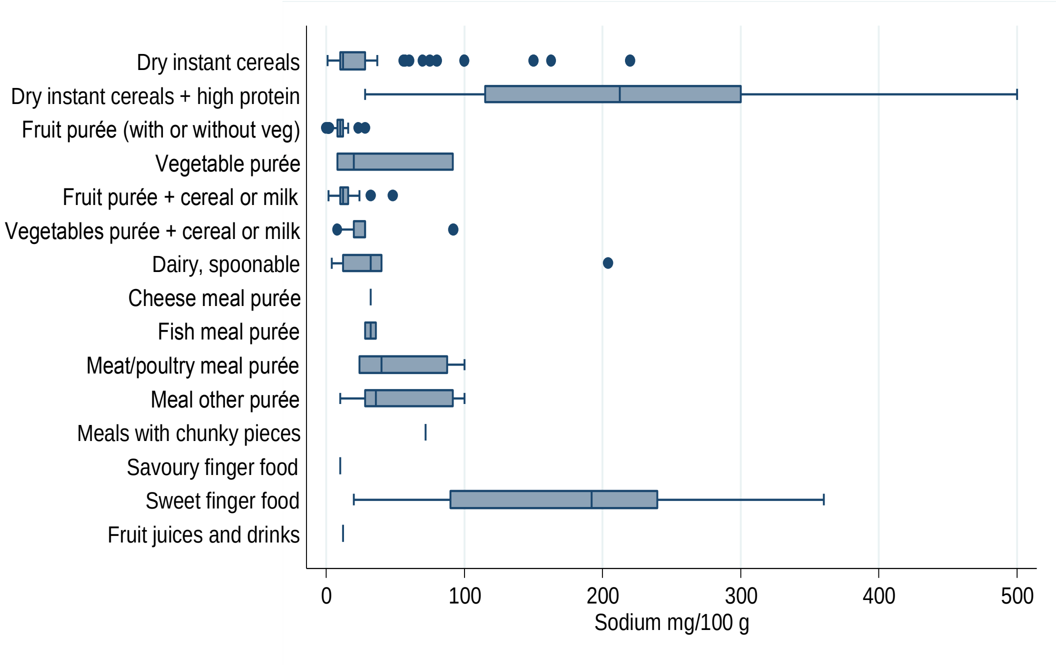

Fig. 22. Box plots of sodium (mg) per 100 kcal in CACFs targeted for 4–36-month-olds on the United Kingdom market in 2016/2017

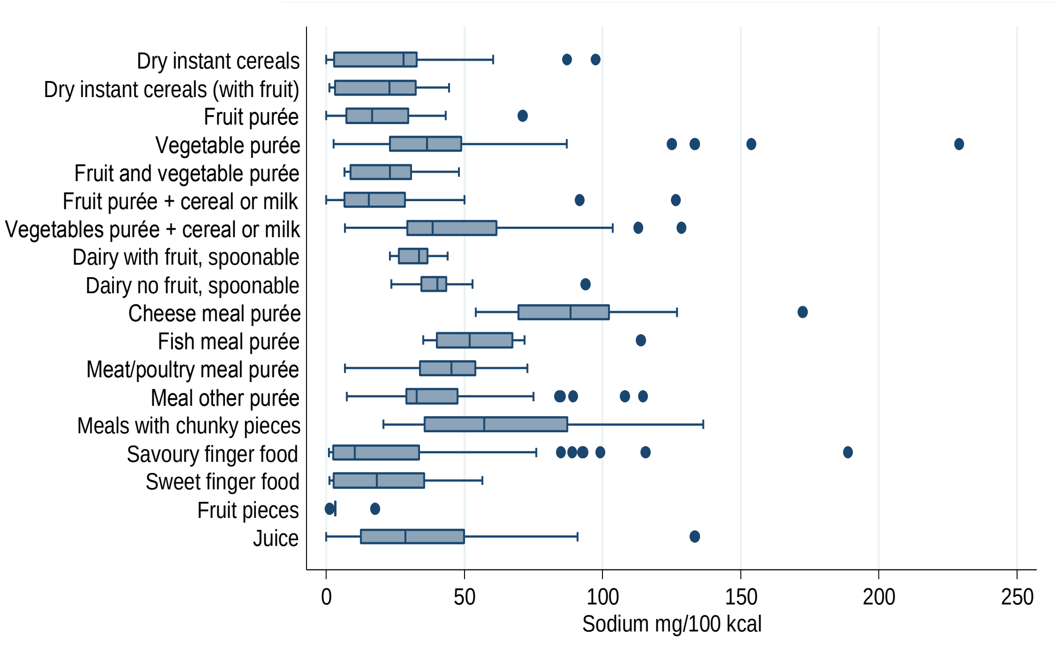

Fig. 23. Box plots of sodium (mg) per 100 kcal in CACFs targeted for 4–36-month-olds on the Danish market in 2016/2017

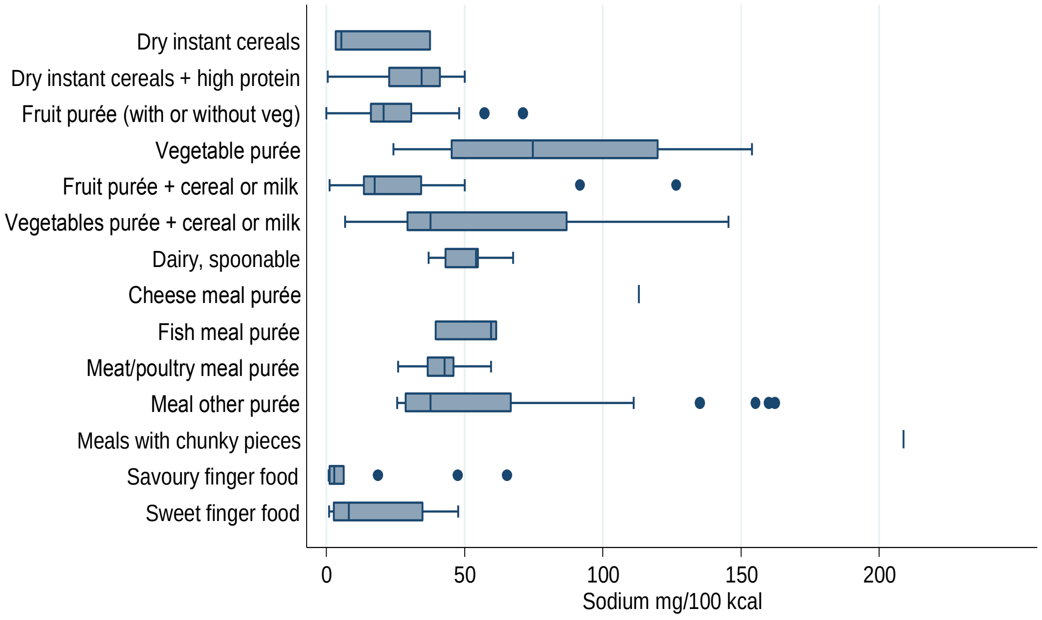

Fig. 24. Box plots of sodium (mg) per 100 kcal product in CACFs targeted for 4–36-month-olds on the Spanish market in 2017

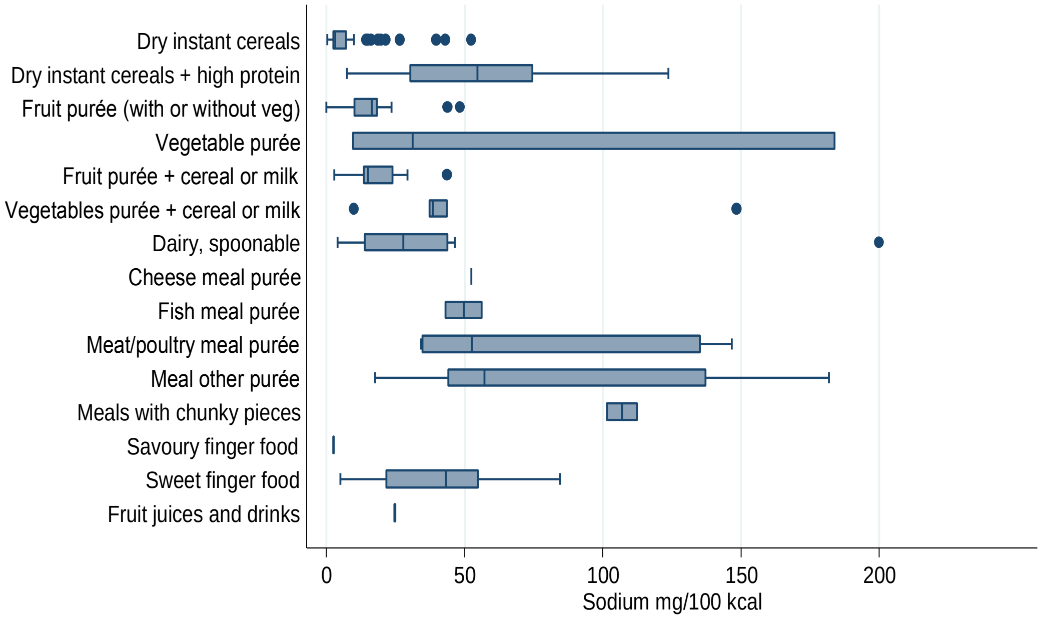

Table 17. Summary of estimated sodium content in infant complementary foods commercially available in the United Kingdom (N = 768) in 2016/2017, showing the percentage of products exceeding different thresholds of sodium mg per 100 kcal product

	Marketed in United Kingdom for infants 4–36 months										
		Percentage (%) of products exceeding different thresholds of sodium mg/100 kcal product					Sodium mg/100 kcal product				
	n	> 10 mg	> 20 mg	> 50 mg	> 100 mg	> 200 mg	Mean	SD	Min	Max	Median
**Dry, powdered and instant**											
Cereal, dry/instant	33	64	64	9[a]	0	0	24	23	0	98	28
Cereal with high added protein	46	57	52	0	0	0	19	15	1	44	23
**Soft–wet spoonable**											
Fruit purée	124	60	43	1[a]	0	0	19	14	0	71	19
Vegetable purée	33	88	79	24[a]	15[a]	3[b]	50	50	3	229	36
Fruit and vegetable blended purée	16	75	62	6[a]	6[a]	6[b]	55	131	7	545	24
Fruit purée with cereal/milk	54	65	39	4[a]	2[a]	0	21	21	0	127	15
Vegetable purée with cereal/milk	43	98	86	28[a]	7[a]	0	48	29	7	129	38
Dairy with fruit, spoonable	10	100	100	0	0	0	32	7	23	44	34
Dairy no fruit, spoonable	16	100	100	19[a]	0	0	42	16	24	94	40
Cheese meal purée	18	100	100	100	28[a]	0	91	29	54	172	88
Fish meal purée	9	100	100	56[a]	11[a]	0	58	24	35	114	52
Meat meal purée	37	97	92	32[a]	0	0	44	15	7	73	45
Meal other purée	81	99	91	23[a]	2[a]	0	41	21	7	115	33
**Meals with chunky pieces**											
Meal meat or fish, vegetable tray/pot	89	100	100	60[a]	18[a]	0	66	32	21	136	57
**Dry finger foods and snacks**											
Savoury finger food	80	50	37	11[a]	2[a]	0	24	32	1	189	10
Sweet finger food and confectionery, rusks[c]	57	56	44	2[a]	0	0	21	18	1	56	18
Fruit pieces/dried	9	22	0	0	0	0	6	7	1	18	3
**Juices and drinks**											
Fruit juice[c]	13	77	62	23[a]	15[a]	0	44	47	0	133	29

[a] Red text indicates the percentage of products in each category that exceed proposed new thresholds for sodium content of food per 100 g product. The current restrictions are also given in mg sodium per 100 mg product; these amounts for each category are not shown. Added salt versus intrinsic sodium could not be determined; this is regulated for some product types.
[b] Blue text indicates the percentage of products in each category that exceed current European Commission thresholds for sodium content of food per 100 g product.
[c] NPM recommends all juice, all confectionery and sweet snacks > 15% energy from sugar are not marketed for infants and young children up to 36 months.

Table 18. Summary of estimated sodium content in infant complementary foods commercially available in Denmark (N = 319) in 2016/2017, showing the percentage of products exceeding different thresholds of sodium mg per 100 kcal product

	Marketed in Denmark for infants 4–36 months										
		Percentage (%) of products exceeding different thresholds of sodium mg/100 kcal product					Sodium mg/100 kcal product				
	n	> 10 mg	> 20 mg	> 50 mg	> 100 mg	> 200 mg	Mean	SD	Min	Max	Median
**Dry, powdered and instant**											
Cereal, dry/instant	3	33	33	0	0	0	15	19	3	38	5
Cereal with high added protein	43	88	83	0	0	0	31	14	0	50	34
**Soft–wet spoonable**											
Fruit purée (with/without vegetables)	97	84	51	2[a]	0	0	23	13	0	71	21
Vegetable purée	10	100	100	70[a]	40[a]	0	81	46	24	154	75
Fruit purée with cereal/milk	47	81	45	4[a]	2[a]	0	26	23	1	127	17
Vegetable purée with cereal/milk	21	90	90	33[a]	24[a]	0	57	44	7	145	38
Dairy with/without fruit	5	100	100	60[a]	0	0	51	12	37	68	54
Cheese meal purée	1	100	100	100	100[a]	0	113	-	113	113	113
Fish meal purée	3	100	100	67[a]	0	0	53	12	39	62	59
Meat meal purée	12	100	100	8[a]	0	0	42	9	26	60	43
Meal other purée	44	100	100	35[a]	14[a]	0	55	39	26	162	38
**Meals with chunky pieces**											
Meal meat or fish, vegetable tray/pot	1	100	100	100[a]	100[a]	100[b]	209	-	209	209	209
**Dry finger foods and snacks**											
Savoury finger food	15	20	20	7[a]	0	0%	11	19	1	65	3
Sweet finger food and confectionery[c]	15	33	27	0	0	0%	15	17	1	48	8
Fruit pieces/dried	0	–	–	–	–	–	–	–	–	–	–
**Juices and drinks**											
Fruit juice[c]	0	–	–	–	–	–	–	–	–	–	–

[a] Red text indicates the percentage of products in each category that exceed proposed new thresholds for sodium content of food per 100 g product. The current restrictions are also given in mg sodium per 100 mg product; these amounts for each category are not shown. Added salt versus intrinsic sodium could not be determined; this is regulated for some product types.
[b] Blue text indicates the percentage of products in each category that exceed current European Commission thresholds for sodium content of food per 100 g product.
[c] NPM recommends all juice, all confectionery and sweet snacks > 15% energy from sugar are not marketed for infants and young children up to 36 months.

Table 19. Summary of estimated sodium content in infant complementary foods commercially available in Spain (N = 241) in 2017, showing the percentage of products exceeding different thresholds of sodium mg per 100 kcal product

	Marketed in Spain for infants 4–36 months										
		Percentage (%) of products exceeding different thresholds of sodium mg/100 kcal product					Sodium mg/100 kcal product				
	**n**	**> 10 mg**	**> 20 mg**	**> 50 mg**	**> 100 mg**	**> 200 mg**	**Mean**	**SD**	**Min**	**Max**	**Median**
**Dry, powdered and instant**											
Cereal, dry/instant	74	15	8	1[a]	0	0	7	10	0	52	3
Cereal with high added protein	8	87	87	50[a]	13[b]	0	56	36	7	124	55
**Soft-wet spoonable**											
Fruit purée (with/without vegetables)	41	71	15	0	0	0	15	10	0	48	16
Vegetable purée	3	67	67	33[a]	33[a]	0	75	95	10	184	31
Fruit purée with cereal/milk	25	88	38	0	0	0	18	8	3	44	15
Vegetable purée with cereal/milk	5	80	80	20[a]	20[a]	0	56	54	10	148	38
Dairy with/without fruit	15	80	53	7[a]	7[a]	0	36	48	4	200	28
Cheese meal purée	1	100	100	100	0	0	52	–	52	52	52
Fish meal purée	2	100	100	50[a]	0	0	50	9	43	56	50
Meat meal purée	11	100	100	55[a]	36[a]	0	79	49	34	147	53
Meal other purée	43	100	88	58[a]	47[a]	0	87	50	18	182	57
**Meals with chunky pieces**											
Meal meat or fish, vegetable tray/pot	2	100	100	100[a]	100[a]	0	107	8	101	113	107
**Dry finger foods and snacks**											
Savoury finger food	2	0	0	0	0	0	3	0	3	3	3
Sweet finger food and confectionery[c]	7	85	71	43[a]	0	0	43	25	5	85	43
Fruit pieces/dried	0	–	–	–	–	–	–	–	–	–	–
**Juices and drinks**											
Fruit juice[c]	2	100	100	0	0	0	25	0	24	25	25

[a] Red text indicates the percentage of products in each category that exceed proposed new thresholds for sodium content of food per 100 g product. The current restrictions are also given in mg sodium per 100 mg product; these amounts for each category are not shown. Added salt versus intrinsic sodium could not be determined; this is regulated for some product types.
[b] Blue text indicates the percentage of products in each category that exceed current European Commission thresholds for sodium content of food per 100 g product.
[c] NPM recommends all juice, all confectionery and sweet snacks > 15% energy from sugar are not marketed for infants and young children up to 36 months.

Applying the suggested threshold of 50 mg/100 kcal sodium also demonstrated that within each product category (apart from cheese-meal purée), it was feasible to achieve this threshold (a percentage of products already comply with the proposed cut-off in at least one of the countries studied). Table 33, 36 and 39 in Chapter 2.5 show the percentage of products in the United Kingdom, Denmark and Spain by food category meeting both of the thresholds for sodium content included in the NPM (50 mg sodium per 100 kcal product and 50 mg sodium per 100 g product). Several national dietary recommendations in the European Region advise limiting salt for infants and young children to prevent accustoming them to a high-salt diet.

Trans fatty acids

It was not possible to establish the trans fatty acid content of the foods from the three countries, as this information is not included on the back of packet. Access was gained to trans fatty acid values estimated from ingredients in CACF products on the United Kingdom market in 2012, but these estimates were determined using trans fatty acid content in products for adult consumption.

Just over 10% of the estimates of these 414 products exceeded the WHO recommendation of 1% energy from trans fatty acid, almost all of which were meal-type products. Most contained lamb, beef or cheese, which are known to contain the highest natural trans fatty acid values, so it might reasonably be assumed that the products do not contain artificial trans fatty acids. Nevertheless, according to WHO guidance, complementary foods should contain no industrially produced trans fatty acids *(83)*. This has been introduced into the NPM. Some national dietary recommendations in the European Region for this age group advise that trans fatty acid intake be as low as possible.

Meeting European Commission regulations overall

As far as could be determined from the available pack-label composition data, taking into account salt, protein, fat and carbohydrate content, only a small percentage of CACFs marketed for infants in the United Kingdom, Denmark and Spain (8%, 8% and 4% respectively) did not meet the 2006/125 EC regulations overall (see Chapter 2.5, Table 30–32). In particular, meal products in the United Kingdom and Denmark were unlikely to contain the required protein stipulated in the regulations. Fourteen per cent of United Kingdom meals with chunky pieces, four dried fruit products and eight products on the Danish market also exceed the fat regulations.

Assessing the overall strictness of the compositional criteria of the NPM

This section describes the steps taken to determine what percentage of CACF products recently on the market in United Kingdom, Denmark and Spain in 2016/2017 would meet all proposed nutrient requirements of the NPM (given the available pack-label information) and whether a 5% or 10% reformulation in their contents would shift a substantial proportion of products to meet the proposed requirements.

The overall percentage of products meeting all nutrient requirements is reported by NPM food category and in total for each country. The results can be found in Chapter 2.5, Table 33–41. Three separate analyses (United Kingdom N = 768, Denmark N = 319 and Spain N = 241) have been undertaken to determine the percentage of products in each country meeting:

a) proposed nutrient requirements in the NPM for infants and young children up to 36 months (results of the analyses are shown in Table 33, 36 and 39);

b) proposed nutrient requirements in the NPM for infants and young children after modelling a 5% reformulation in contents towards the proposed NPM (Table 34, 37 and 40); and
c) proposed nutrient requirements in the NPM for infants and young children after modelling a 10% reformulation in contents towards the proposed NPM (Table 35, 38 and 41).

The overall percentage of current products and products after reformulation that would meet all nutrient requirements of the NPM are summarized in Table 20 below.

These results were determined using packet-label information in relation to the following six proposed compositional requirements:

1. no added sugar as per ingredients;
2. finger foods less than 15% energy from total sugar;
3. sodium content less than 50 mg/100 kcal and 50 mg/100 g product (or 100 mg if the name includes cheese);
4. energy density of soft–wet spoonable foods more than or equal to 60 kcal/100 g (except vegetable purées);
5. protein sufficient in meals, as explained below; and
6. fat below existing regulations in the European Commission directive 2006/125/EC.

The following named ingredients were classed as added sugars for this exercise: sugar, (any) syrup, juice or juice concentrate (other than lemon or lime juice), molasses, malt extract, barley malt, malted barley extract, maltose, dextrose, fructose, glucose, sucrose and honey.

For dry cereals, the sodium content should be less than 50 mg/100 kcal (and does not need to also be less than 50 mg/100 g of product).

Protein content was determined as sufficient in:

- puréed meals with cheese in the name if total protein was more than or equal to 3 g/kcal;
- puréed meals with a fish or meat first-named food if the weight of protein from the named sources was more than or equal to 15% of the total weight of product and total protein was more than or equal to 6 g/kcal; and
- other puréed meals if protein from all named sources was more than or equal to 10% of the total weight of the product and total protein was more than or equal to 3.75 g/kcal.

For ease, it was assumed that meals with chunky pieces do not have a fish or meat as the first ingredient in the name, as these had not been subcategorized based on the name, as was done for purées.

The total fat proposed requirements for the NPM remain the same as the regulations in the European Commission Directive 2006/125/ EC, which is less than or equal to 4.5 g/100 kcal for all products except dry cereals (less than or equal to 3.3 g/100 kcal no added high protein) and cheese, fish or meat meals (less than or equal to 6 g/100 kcal).

Meeting proposed NPM content requirements before reformulation

Only about a third of products analysed that were on the market in the United Kingdom (31%), Denmark (34%) and Spain (29%) in 2016/2017 met all six proposed requirements in relation to nutrient content, despite large percentages of products meeting the individual requirements (see Chapter 2.5, Table 33, 36 and 39).

Almost all products (97–100%) in the three countries met the current European Commission regulations for fat, and these requirements remain in the proposed NPM. About three quarters or more (73–82%) met the proposed salt requirements, but only around half of the fish meals and, for Spain, many other meal types did not meet the salt requirement.

Most relevant products met the energy density proposals (81% in the United Kingdom, 68% in Denmark and 84% in Spain), although only about 50% or less of fruit purées and meals with meat (as the first-named food) marketed in the United Kingdom and Denmark met them. The percentage of meals that met the increased protein content proposals was particularly low, especially for those that included fish or meat as the first-named food. Almost all of the savoury finger foods contained less than 15% energy from total sugar requirements (90% in the United Kingdom, and 100% in Denmark and Spain); this may partially be due to the total-sugar content being taken into account during categorization of finger foods. However, only 5% of rusks on the United Kingdom market contained less than 15% energy from total sugar. About half (56%) of the Spanish products did not contain added sugar, against about three quarters of United Kingdom (72%) and Danish (79%) products. The type of product that contained added sugars and the type of sugars added varied across the three countries (see the subsection "Added sugar" above and Chapter 2.5, Table 24–26).

A summary of the total number and percentage of products meeting each of the compositional thresholds/criteria is provided for the United Kingdom, Denmark and Spain alongside the results of the seven pilot study countries in Table 22 (Chapter 2.4)

Modelling theoretical 5% and 10% content reformulations

The 5% and 10% changes towards the proposed nutrient requirements were first modelled in five areas relating to reducing total sugar, total sodium and total fat content, and increasing the energy density and protein content in certain products (Chapter 2.5, Table 34, 35 37, 38, 40 and 41).

In relation to sodium, modelling 5% and 10% reformulations only slightly increased the percentage of products overall meeting the salt requirements. Although many of the categories were affected, these were generally less than 10 percentage point increases, although larger increases can be seen for meat- and fish-named meals in the United Kingdom. Fruit purées and some meal products had substantial increases in products meeting energy density requirements (for example, from 40% to 58% for fruit purées in the United Kingdom, from 50% to 67% for Denmark, and 76% to 85% for Spain). Changes in meeting the protein requirements in meal products were minimal. Substantial increases in meeting the total-sugar threshold in sweet finger foods (from 13% to 20%, then to 33%) were seen in products in Denmark.

Modelling the 5% and 10% reformulations towards the proposed requirements did not substantially alter the percentage of products that met the overall proposed requirements for the six compositional criteria listed above when no restrictions to added sugars were modelled. This ranged from only a further 1% of products in Spain, to 6% in the United Kingdom, to 9% in Denmark meeting the overall requirements in the 10% change model.

When restriction of added sugars for all products was included in the models (a binary shift from "presence of added sugar" to "no added sugars"), this substantially altered the percentage of products that met the overall proposed requirements, with a further 39% of products in Spain, 12% in the United Kingdom and 15% in Denmark meeting the overall requirements in the 10%

change + no added sugar model. Overall in the 5% and 10% change + no added sugar models, 45% and 49% of products on the United Kingdom market (Table 34 and 35), 53% and 58% on the Danish market and (Table 37 and 38) and 67% and 69% on the Spanish market (Table 40 and 41) met all six requirements respectively.

Despite these improvements towards meeting the NPM requirements, only a small percentage of meal-type products in the models met the requirements: this was due mainly to not achieving the increase in protein requirements. This may be expected, as the required protein content increase from those in the European Commission regulations to those in the earlier version of the NPM was substantial, at 25–50%; for the NPM presented in Chapter 1.2, however, the requirements were reduced to those stated in the current European Commission regulations.

Modelling only a "no added sugar" reformulation with other contents remaining the same would produce 42% of products the United Kingdom, 47% in Denmark and 66% in Spain that meet all six requirements (tables by food category not shown).

A summary of the percentage of products meeting NPM requirements in all six areas before and after modelling reformulations is shown in Table 20.

Table 20. Summary of modelling reformulations: percentage of CACF products meeting NPM requirements for all six compositional criteria[a] before and after reformulation

	United Kingdom (%)	Denmark (%)	Spain (%)
No reformulations	31	34	29
Modelling 5% reformulation (added sugar not reformulated)	33	38	30
Modelling 10% reformulation (added sugar not reformulated)	37	43	30
Modelling "no added sugar" only	42	47	66
Modelling 5% reformulation plus reformulations for "no added sugar"	45	53	67
Modelling 10% reformulation plus reformulations for "no added sugar")	49	58	69

[a] The six criteria for reformulation are: restricting added sugar, reducing total sugar, sodium and total fat content, and increasing the energy density and protein content.

These results indicate that initial reformulation efforts should focus on "no added sugars" to provide the largest effect in meeting the proposed NPM requirements. As mentioned in other sections, however, further consideration of what should be classed as added sugars may be needed.

It should be noted that these analyses do not consider whether the products met the proposed labelling requirements and promotional restrictions in the NPM. For instance, it is proposed that "no added sugars" or other claims should not be allowed on the pack labels and other promotion material.

2.4. Pilot testing the NPM with a further seven European countries

A pilot test was conducted to assess the practical applicability of the earlier version of the NPM and determine where improvements were needed. Seven countries – Estonia, Hungary, Italy, Malta, Norway, Portugal and Slovenia – provided nutrition and market data on approximately 100–400 products from their domestic markets. These data were cleaned and analysed, and the proportion of products passing each criterion of the proposed NPM, and the NPM overall, was calculated.

The percentage of products passing the NPM in its entirety ranges from 15% (Hungary) to 42% (Estonia) (Table 21) for the six criteria that were assessed (as detailed in Table 22 and by food category in Chapter 2.5, Table 42–48). The criteria with the lowest pass rate concerned the protein content of meals, which was low across all countries, particularly Portugal (11%) and Hungary (12%). The percentage energy from total sugar in finger foods criteria also had a relatively low pass rate, particularly in Hungary and Italy (14%). The energy density pass rates for purées ranged from 61–84%, indicating that this could be increased in some purées for infants below 12 months. With regard to criteria affecting all products, the pass rate for "no added sugars/sweeteners" was lower than that for salt, Hungary having the lowest pass rate (42%) for added sugars/sweeteners (Table 22).

Table 21. Number of CACF products in the pilot test and the percentage meeting the overall composition criteria of the NPM

Country	Number of products submitted	Number of products meeting NPM	Percentage (%) of products meeting NPM	Percentage (%) of products meeting NPM if all products contained no added sugar
**Estonia**	134	56	**42**	59
**Hungary**	123	19	**15**	54
**Italy**	430	140	**33**	58
**Malta**	243	88	**36**	48
**Norway**	107	38	**36**	51
**Portugal**	125	41	**33**	66
**Slovenia**	152	35	**23**	58

The pilot study findings are broadly comparable with the validation undertaken for Danish, Spanish and United Kingdom products, where approximately a third of products passed the NPM overall (Table 20 (Chapter 2.3 and Table 22). The pilot data showed that between 23% and 58% of all products contained added sugar/sweeteners; if these products were only reformulated to remove the added sugars and sweeteners, between 51% and 66% of products in the seven countries would pass the proposed NPM requirements. This reflects the findings for the Danish, Spanish and United Kingdom products, indicating that removing added sugars from CACFs could be a priority area of focus.

Detailed feedback was received from the participating countries, the main themes from which included the strictness of certain categories and thresholds. Estonia, for example, felt that the less than 5% added water threshold for category 2b, vegetable purées, was too strict, Hungary considered the sugar, salt and energy density thresholds too strict due to their low pass rate, and

Table 22. Summary of CACF products from 10 countries meeting proposed NPM requirements by compositional criteria

	Total number of products	**Meets all six proposed NPM nutrient requirements (%)**	Percentage of products meeting NPM requirements: No added sugars/ sweeteners[a] (%)	Finger food < 15% total energy from total sugar (%)	Sodium < 50 mg/100 kcal and < 50 mg/100 g[b] (%)	Energy density > 60 kcal/100 g[c] (%)	Protein sufficient in meals[d] (%)	Fat not above current regulations[e] (%)
**Seven pilot countries (data collected 2018)**								
Estonia	134	**42**	77	60	92	68	30	98
Hungary	123	**15**	42	14	85	61	12	100
Malta	243	**36**	76	48	83	64	26	95
Norway	107	**36**	68	50	72	81	18	99
Portugal	125	**33**	58	50	79	81	11	98
Slovenia	152	**23**	51	45	73	84	21	98
Italy	430	**33**	62	14	77	80	42	96
**Original validation countries (data collected 2016/2017)**								
United Kingdom	768	**31**	72	56	73	70	38	97
Demark	319	**34**	79	57	82	68	15	97
Spain	241	**29**	56	22	80	84	39	100

[a] The following listed ingredients have been classed as added sugars and sweeteners for this analysis: sugar, (any) syrup, juice (but lemon or lime juice are not), molasses, malt extract, barley malt, malted barley extract, maltose, dextrose, fructose, glucose, sucrose and honey. Added lactose was not classed as an added sugar as it is a component of milk. Additionally, galacto-oligosaccharides, fructo-oligosaccharides, inulin, maltodextrose, maltodextrin and glycerol, which are often found in breast-milk substitutes, were not classed as sweeteners.
[b] Or < 100 mg sodium/100 kcal and 100 mg sodium/100 g if cheese meal; or only < 50 mg/100 kcal if dry cereal.
[c] Energy density requirements have not been set for the dry foods. Upper limits for energy density or portion size may be required for finger foods.
[d] Protein is sufficient in puréed meals with cheese in name if total protein is ≥ 3 g/kcal. Protein is sufficient in puréed meals with a fish or meat first-named food if these are ≥ 15% of total weight of product and total protein is > 6 g/kcal. Protein is sufficient in other puréed meals if named protein is ≥ 10% of total weight of product and total protein is ≥ 3.75 g/kcal. In the analysis of meals with chunky pieces, it was assumed a fish or meat is not first-named food for ease of analysis – this may not be the case, meaning the percentage shown meeting the criteria may be higher.
[e] Total fat requirement is < 4.5 g/100 kcal for all products except dry cereals (< 3.3 g/100 kcal, no added high protein) and cheese, fish or meat meals (6 g/100 kcal).

Norway had similar views on the protein thresholds for meals with fish or meat. Norway also suggested having a saturated fat threshold instead of a total fat threshold. Regarding the categorization of products, Estonia found the distinction between category 4b, sweet snacks, and category 4c, rusks and teething biscuits, to be confusing, and questioned whether fruit should be restricted as a sweetener. Slovenia also suggested that an extra category be added for milk drinks with sweet flavourings, such as chocolate.

Following on from the results of the NPM pilot test and the feedback received from the countries involved, the proposed NPM has been updated to simplify categories and improve its practical application. Categories 4b (sweet snacks) and 4c (rusks and teething biscuits) have been merged, as most rusks and teething biscuits contained added sugar, and United Kingdom National Health Service advice is to avoid giving infants and young children such products due to the added sugar content. Category 2b (vegetable purées) criteria have been changed to more than or equal to 95% single or mixed vegetables or legumes *and water combined*, with a “less than 25% added water” threshold; however, further research on this may be needed. Categories 2a (fruit purée) and 2c (fruit purée with cereal or milk) have been merged, as the proportions of cereal are rarely provided, making categorization difficult.

The decision was taken to retain the existing European Commission directive thresholds for protein. A very low proportion of products passed the higher thresholds. Concern remained, however, that some CACFs containing meat, fish or dairy products have lower protein density than homemade equivalents and may not contribute sufficiently to iron intake. Further discussion is needed regarding protein thresholds with a view to ensuring that sufficient protein is provided, while being mindful of evidence that overconsumption of protein at a young age may be linked to overweight and obesity in later life.

The pilot testing exercise also collected information on earliest age of suitability. A high proportion of products identified in these countries were promoted as suitable for infants under 6 months (Table 23).

Table 23. Percentage of CACF products promoted as suitable for infants under 6 months

**Country**	**Percentage (number/out of)**
Estonia	45 (60/134)
Hungary	50 (62/123)
Italy	47 (200/430)
Malta	34 (82/243)
Norway	10 (11/107)
Portugal	20 (25/125)
Slovenia	27 (41/152)

2.5. Supplementary tables

This section contains supplementary tables that have been referred to in chapters 2.3 and 2.4.

- Table 24–26 display the percentage of products in the United Kingdom, Denmark and Spain containing "added sugars" by food category.
- Table 27–29 display the percentage of products in the United Kingdom, Denmark and Spain in each food category containing lactose and other sweet ingredients not classed as "added sugars" in the NPM.
- Table 30–32 display the percentage of products in the United Kingdom, Denmark and Spain not meeting all regulations in the European Commission directive 2006/125/EC, determined, where possible, relating to salt, protein, fat and carbohydrate content.
- Table 33, 36 and 39 display the percentage of products in the United Kingdom, Denmark and Spain meeting proposed nutrient requirements in the NPM for infants and young children up to 36 months.
- Table 34, 37 and 40 display the percentage of products in the United Kingdom, Denmark and Spain meeting proposed nutrient requirements in the NPM after modelling a 5% reformulation in contents towards the proposed NPM.
- Table 35, 38 and 41 display the percentage of products in the United Kingdom, Denmark and Spain meeting proposed nutrient requirements in the NPM after modelling a 10% reformulation in contents towards the proposed NPM.
- Table 42–48 display the percentage of products in the pilot countries (Estonia, Hungary, Italy, Malta, Norway, Portugal and Slovenia) meeting proposed nutrient requirements in the NPM for infants and young children up to 36 months.

Table 24. Added sugars in infant complementary foods commercially available in United Kingdom in 2016/2017

	Percentage of products containing added sugars marketed in the United Kingdom in 2016/2017 for infants 4–36 months											
	N	Percentage of products containing added sugars	Juice (other than lemon)	Sugar/ sucrose	Dextrose	Fructose	Glucose	Maltose	Syrup	Honey	Malt extract/ malted barley extract	Molasses
**1 Dry, powdered and instant**												
1a Cereal, dry/instant	31	13	0	13	0	0	0	0	0	0	3	0
1b Cereal with high added protein	48	35	4	4	0	0	0	0	0	4	23	0
**2 Soft–wet spoonable**												
2a Fruit purée (with/without vegetables)	140	18	17	1	0	0	0	0	0	0	0	0
2b Vegetable purée	33	0	0	0	0	0	0	0	0	0	0	0
2c Fruit purée with cereal/milk	54	44	39	7	0	0	0	0	0	0	0	0
2d Vegetable purée with cereal/milk	43	2	2	0	0	0	0	0	0	0	0	0
2l Dairy with/without fruit	26	62	4	58	0	0	0	0	0	0	0	0
2e Cheese meal purée	18	6	6	0	0	0	0	0	0	0	0	0
2f Fish meal purée	9	22	22	0	0	0	0	0	0	0	0	0
2g Meat meal purée	37	5	5	0	0	0	0	0	0	0	0	0
2h Meal other, spoonable	81	6	6	0	0	0	0	0	0	0	0	0
**3 Meals with chunky pieces**												
3a Meal meat or fish, vegetable tray/pot	89	18	11	12	0	0	0	0	3	1	0	6
**4 Dry finger foods and snacks**												
4d Savoury finger food	79	43	24	8	3	0	0	0	0	5	9	3
4a/b Sweet finger food and confectionary	39	97	69	15	5	3	10	0	10	0	18	0
4c Rusks and teething biscuits	19	95	0	95	16	6	0	0	5	5	42	0
4e Fruit pieces/dried	9	22	0	0	0	0	0	22	0	0	0	0
**5 Juices and drinks**												
5a Fruit drink	13	100	100	0	0	0	0	0	0	0	0	0
Overall for all products	768	28	16	9	1	0	1	0	1	1	4	1

Note: galactose and trehalose were not listed as ingredients in any of the products.

Table 25. Added sugars in infant complementary foods commercially available in Denmark in 2016/2017

	Percentage of products containing added sugars marketed in Denmark in 2016/2017 for infants 4–36 months											
	N	Percentage (%) of products containing added sugars	Juice (other than lemon) (%)	Sugar/ sucrose (%)	Dextrose (%)	Fructose (%)	Glucose (%)	Maltose (%)	Syrup (%)	Honey (%)	Malt extract/ malted barley extract (%)	Molasses (%)
**1 Dry, powdered and instant**												
1a Cereal, dry/instant	3	0	0	0	0	0	0	0	0	0	0	0
1b Cereal with high added protein	45	33	31	0	0	0	2	0	2	0	0	0
**2 Soft–wet spoonable**												
2a Fruit purée (with/without vegetables)	97	12	12	0	0	0	0	0	0	0	0	0
2b Vegetable purée	10	0	0	0	0	0	0	0	0	0	0	0
2c Fruit purée with cereal/milk	47	47	47	0	0	0	0	0	0	0	0	0
2d Vegetable purée with cereal/milk	21	5	5	0	0	0	0	0	0	0	0	0
2l Dairy with/without fruit	5	0	0	0	0	0	0	0	0	0	0	0
2e Cheese meal purée	1	0	0	0	0	0	0	0	0	0	0	0
2f Fish meal purée	3	0	0	0	0	0	0	0	0	0	0	0
2g Meat meal purée	12	0	0	0	0	0	0	0	0	0	0	0
2h Meal other, spoonable	44	9	7	0	0	0	0	0	2	0	0	0
**3 Meals with chunky pieces**												
3a Meal meat or fish, vegetable tray/pot	1	0	0	0	0	0	0	0	0	0	0	0
**4 Dry finger foods and snacks**												
4d Savoury finger food	15	7	7	0	0	0	0	0	0	0	0	0
4a/b Sweet finger food and confectionary	15	80	33	0	0	0	0	0	0	0	47	0
4c Rusks and teething biscuits												
4e Fruit pieces/dried												
**5 Juices and drinks**												
5a Fruit drink												
Overall for all products	319	21	18	0	0	0	0	0	1	0	2	0

Note: galactose and trehalose were not listed as ingredients in any of the products.

Table 26. Added sugars in infant complementary foods commercially available in Spain in 2017

	Percentage of products containing added sugars marketed in Spain in 2017 for infants 4–36 months											
	N	Percentage (%) of products containing added sugars	Juice (other than lemon) (%)	Sugar/ sucrose (%)	Dextrose (%)	Fructose (%)	Glucose (%)	Maltose (%)	Syrup (%)	Honey (%)	Malt extract/ malted barley extract (%)	Molasses (%)
**1 Dry, powdered and instant**												
1a Cereal, dry/instant	74	50	1	30	3	0	3	1	3	18	18	0
1b Cereal with high added protein	8	63	0	38	0	0	0	0	0	13	13	0
**2 Soft–wet spoonable**												
2a Fruit purée (with/without vegetables)	41	61	61	5	0	0	0	61	0	0	5	0
2b Vegetable purée	3	33	0	0	0	0	0	0	0	0	0	0
2c Fruit purée with cereal/milk	25	72	56	48	0	0	0	56	0	0	36	0
2d Vegetable purée with cereal/milk	5	0	0	0	0	0	0	0	0	0	0	0
2l Dairy with/without fruit	15	73	0	67	0	0	0	0	7	13	7	0
2e Cheese meal purée	1	0	0	0	0	0	0	0	0	0	0	0
2f Fish meal purée	2	0	0	0	0	0	0	0	0	0	0	0
2g Meat meal purée	11	0	0	0	0	0	0	0	0	0	0	0
2h Meal other, spoonable	43	0	0	0	0	0	0	0	0	0	0	0
**3 Meals with chunky pieces**												
3a Meal meat or fish, vegetable tray/pot	2	50	0	50	0	0	0	0	0	0	0	0
**4 Dry finger foods and snacks**												
4d Savoury finger food	2	0	0	0	0	0	0	0	0	0	0	0
4a/b Sweet finger food and confectionary	7	100	0	86	14	0	0	0	29	29	43	0
4c Rusks and teething biscuits												
4e Fruit pieces/dried												
**5 Juices and drinks**												
5a Fruit drink	2	100	100	0	0	0	0	100	0	0	0	0
Overall for all products	241	44	17	24	1	0	1	17	2	8	12	0

Note: galactose and trehalose were not listed as ingredients in any of the products.

Table 27. Other added sweet-tasting ingredients in infant complementary foods commercially available in the United Kingdom in 2016/2017

	Percentage of products containing other added sweet ingredients marketed in the United Kingdom in 2016/2017 for infants 4–36 months					
	N	Lactose (%)	Oligo – sacchrides[a] (%)	Inulin (%)	Maltodextrin (%)	Glycerol (%)
**1 Dry, powdered and instant**						
1a Cereal, dry/instant	31	0	0	3	16	0
1b Cereal with high added protein	48	0	8	4	77	0
**2 Soft–wet spoonable**						
2a Fruit purée (with/without vegetables)	140	0	0	0	0	0
2b Vegetable purée	33	0	0	0	0	0
2c Fruit purée with cereal/milk	54	0	0	0	0	0
2d Vegetable purée with cereal/milk	43	0	0	0	0	0
2l Dairy with/without fruit	26	0	0	0	0	0
2e Cheese meal purée	18	0	0	0	0	0
2f Fish meal purée	9	0	0	0	0	0
2g Meat meal purée	37	0	0	0	0	0
2h Meal other, spoonable	81	0	0	0	0	0
**3 Meals with chunky pieces**						
3a Meal meat or fish, vegetable tray/pot	89	0	0	0	0	0
**4 Dry finger foods and snacks**						
4d Savoury finger food	79	3	0	0	1	0
4a/b Sweet finger food and confectionary	39	0	0	5	0	5
4c Rusks and teething biscuits	19	0	0	37	0	0
4e Fruit pieces/dried	9	0	0	0	0	0
**5 Juices and drinks**						
5a Fruit drink	13	0	0	0	0	0
Overall for all products	768	0	1	2	6	0

[a] Such as fructo-oligosaccharides or galactosaccaharides.

Table 28. Other added sweet-tasting ingredients in infant complementary foods commercially available in Denmark in 2016/2017

	Percentage of products containing other added sweet ingredients marketed in Denmark in 2016/2017 for infants 4–36 months					
	N	Lactose (%)	Oligo – sacchrides[a] (%)	Inulin (%)	Maltodextrin (%)	Glycerol (%)
**1 Dry, powdered and instant**						
1a Cereal, dry/instant	3	0	0	0	0	0
1b Cereal with high added protein	45	9	0	0	16	0
**2 Soft–wet spoonable**						
2a Fruit purée (with/without vegetables)	97	0	0	0	0	0
2b Vegetable purée	10	0	0	0	0	0
2c Fruit purée with cereal/milk	47	0	0	0	0	0
2d Vegetable purée with cereal/milk	21	0	0	0	0	0
2l Dairy with/without fruit	5	0	0	0	0	0
2e Cheese meal purée	1	0	0	0	0	0
2f Fish meal purée	3	0	0	0	0	0
2g Meat meal purée	12	0	0	0	0	0
2h Meal other, spoonable	44	0	0	0	0	0
**3 Meals with chunky pieces**						
3a Meal meat or fish, vegetable tray/pot	1	0	0	0	0	0
**4 Dry finger foods and snacks**						
4d Savoury finger food	15	0	0	0	0	0
4a/b Sweet finger food and confectionary	15	0	0	0	13	0
4c Rusks and teething biscuits						
4e Fruit pieces/dried						
**5 Juices and drinks**						
5a Fruit drink						
Overall for all products	319	1	0	0	3	0

[a] Such as fructo-oligosaccharides or galactosaccaharides.

Table 29. Other sweet-tasting ingredients in infant complementary foods commercially available in Spain in 2017

	Percentage of products containing other sweet ingredients marketed in Spain in 2017 for infants 4–36 months						
	N	Lactose (&)	Oligo – sacchrides[a] (%)	Inulin (%)	Dextrinomaltosa (%)	Maltodextrin (&)	Glycerol (%)
**1 Dry, powdered and instant**							
1a Cereal, dry/instant	74	0	46	8	31	30	0
1b Cereal with high added protein	8	0	88	0	63	13	0
**2 Soft–wet spoonable**							
2a Fruit purée (with/without vegetables)	41	0	0	0	0	0	0
2b Vegetable purée	3	0	0	0	0	33	0
2c Fruit purée with cereal/milk	25	0	4	0	0	4	0
2d Vegetable purée with cereal/milk	5	0	0	0	0	40	0
2l Dairy with/without fruit	15	13	20	0	0	13	0
2e Cheese meal purée	1	0	0	0	0	0	0
2f Fish meal purée	2	0	0	0	0	0	0
2g Meat meal purée	11	0	0	0	0	0	0
2h Meal other, spoonable	43	2	0	0	0	0	0
**3 Meals with chunky pieces**							
3a Meal meat or fish, vegetable tray/pot	2	0	0	0	0	0	0
**4 Dry finger foods and snacks**							
4d Savoury finger food	2	0	0	0	0	0	0
4a/b Sweet finger food and confectionary	7	0	0	0	0	14	0
4c Rusks and teething biscuits							
4e Fruit pieces/dried							
**5 Juices and drinks**							
5a Fruit drink	2	0	0	0	0	0	0
Overall for all products	241	1	19	3	12	13	0

[a]Such as fructo-oligosaccharides or galactosaccaharides.

Table 30. Percentage of products marketed in the United Kingdom not meeting European Commission regulations

	Marketed in the United Kingdom in 2016/2017 for infants 4–36 months					
	N	Not meeting all four nutrient regulations (%)	Sodium content high[a] (%)	Protein insufficent[b] (%)	Fat content high[c] (%)	Carbohydrate content high[d] (%)
**1 Dry, powdered and instant**						
1a Cereal, dry/instant	31	0	0	NA	0	NA
1b Cereal with high added protein	48	0	0	0	0	NA
**2 Soft–wet spoonable**						
2a Fruit purée (with/without vegetables)	140	2	1	NA	0	1
2b Vegetable purée	33	3	3	NA	0	NA
2c Fruit purée with cereal/milk	54	0	0	NA	0	NA
2d Vegetable purée with cereal/milk	43	2	0	NA	2	NA
2l Dairy with/without fruit	26	0	0	NA	0	0
2e Cheese meal purée	18	0	0	0[b]	0	NA
2f Fish meal purée	9	44	0	44[b]	0	NA
2g Meat meal purée	37	14	0	14[b]	0	NA
2h Meal other, spoonable	81	9	0	9[b]	0	NA
**3 Meals with chunky pieces**						
3a Meal meat or fish, vegetable tray/pot	89	27	0	12[b]	14	NA
**4 Dry finger foods and snacks**						
4d Savoury finger food	79	14	14	NA	3	NA
4a/b Sweet finger food and confectionary	39	3	3	NA	0	NA
4c Rusks and teething biscuits	19	5	0	NA	5	NA
4e Fruit pieces/dried	9	44	0	NA	44	NA
**5 Juices and drinks**						
5a Fruit drink	13	0	0	NA	0	0
Overall for all products	768	8	2		3	

NA = not applicable. Unable to determine amount of calcium in products.
Additional stipulations of European Commission regulations:

[a] ≥ 200 mg/100 kcal and ≥ 200 mg/100 g; or ≥ 300 mg/100 kcal if cheese meal; or only ≥ 100 mg/100 kcal if dry cereal.

Unable to determine whether sodium salts added to fruit, desserts or puddings except for technological purposes.

[b] Protein is sufficient in puréed meals with cheese in name if total protein is > 3 g/kcal. Protein is sufficient in puréed meals with a fish or meat first-named food if these are ≥ 10% of total weight of product and total protein is > 4 g/kcal. Protein is sufficient in other puréed meals if named protein is ≥ 8% of total weight of product and total protein is > 3 g/kcal. The calculation for meals with chunky pieces assumed a fish or meat is not first-named food – this may not be the case, meaning the percentage shown not meeting the criteria may be lower than actual.

[c] Total fat requirement is < 4.5 g/100 kcal for all products except dry cereals (< 3.3 g/100 kcal no added high protein) and cheese, fish or meat meals (6 g/100 kcal). Unable to determine lauric acid, myristc acid and linoleic acid content for which there are requirements when total fat content is over 3.3 g/100 kcal in cereals with high protein content.

[d] Total carbohydrates shall not exceed 20 g/100 fruit-only dishes, 25 g/100 g deserts and puddings, 10/100 g vegetable juices, 15 g/100 ml fruit juices, and 5 g/100 g other non-milk-based drinks. Unable to determine the amount of carbohydrate from fructose, glucose, glucose syrups or honey in dry cereal products.

Table 31. Percentage of products marketed in Denmark not meeting European Commission regulations

	Marketed in Denmark in 2016/2017 for infants 4–36 months					
	N	Not meeting all four nutrient regulations (%)	Sodium content high[a] (%)	Protein insufficent[b] (%)	Fat content high[c] (%)	Carbohydrate content high[d] (%)
**1 Dry, powdered and instant**						
1a Cereal, dry/instant	3	0	0	NA	0	NA
1b Cereal with high added protein	45	13	7	2	4	NA
**2 Soft–wet spoonable**						
2a Fruit purée (with/without vegetables)	97	2	0	NA	0	2
2b Vegetable purée	1	10	0	NA	10	NA
2c Fruit purée with cereal/milk	47	0	0	NA	0	NA
2d Vegetable purée with cereal/milk	21	10	0	NA	10	NA
2l Dairy with/without fruit	5	40	0	NA	40	0
2e Cheese meal purée	1	0	0	0[b]	0	NA
2f Fish meal purée	3	67	0	33[b]	33	NA
2g Meat meal purée	12	25	0	25[b]	0	NA
2h Meal other, spoonable	44	11	3	9[b]	0	NA
**3 Meals with chunky pieces**						
3a Meal meat or fish, vegetable tray/pot	1	100	100	0[b]	0	NA
**4 Dry finger foods and snacks**						
4d Savoury finger food	15	7	7	NA	0	NA
4a/b Sweet finger food and confectionary	15	0	0	NA	0	NA
4c Rusks and teething biscuits						
4e Fruit pieces/dried						
**5 Juices and drinks**						
5a Fruit drink						
Overall for all products	319	8	2		3	

NA = not applicable. Unable to determine amount of calcium in products.
Additional stipulations of European Commission regulations:

[a] ≥ 200 mg/100 kcal and ≥ 200 mg/100 g; or ≥ 300 mg/100 kcal if cheese meal; or only ≥ 100 mg/100 kcal if dry cereal.

Unable to determine whether sodium salts added to fruit, desserts or puddings except for technological purposes.

[b] Protein is sufficient in puréed meals with cheese in name if total protein is > 3 g/kcal. Protein is sufficient in puréed meals with a fish or meat first-named food if these are ≥ 10% of total weight of product and total protein is > 4 g/kcal. Protein is sufficient in other puréed meals if named protein is ≥ 8% of total weight of product and total protein is > 3 g/kcal. The calculation for meals with chunky pieces assumed a fish or meat is not first-named food – this may not be the case, meaning the percentage shown not meeting the criteria may be lower than actual.

[c] Total fat requirement is < 4.5 g/100 kcal for all products except dry cereals (< 3.3 g/100 kcal no added high protein) and cheese, fish or meat meals (6 g/100 kcal). Unable to determine lauric acid, myristc acid and linoleic acid content for which there are requirements when total fat content is over 3.3 g/100 kcal in cereals with high protein content.

[d] Total carbohydrates shall not exceed 20 g/100 fruit-only dishes, 25 g/100 g deserts and puddings, 10/100 g vegetable juices, 15 g/100 ml fruit juices, and 5 g/100 g other non-milk-based drinks. Unable to determine the amount of carbohydrate from fructose, glucose, glucose syrups or honey in dry cereal products.

Table 32. Percentage of products marketed in Spain not meeting European Commission regulations

	Marketed in Spain in 2016/2017 for infants 4–36 months					
	N	Not meeting all four nutrient regulations (%)	Sodium content high[a] (%)	Protein insufficent[b] (%)	Fat content high[c] (%)	Carbohydrate content high[d] (%)
**1 Dry, powdered and instant**	N					
1a Cereal, dry/instant	74	0	0	NA	0	NA
1b Cereal with high added protein	8	13	13	0	0	NA
**2 Soft–wet spoonable**						
2a Fruit purée (with/without vegetables)	41	0	0	NA	0	0
2b Vegetable purée	3	0	0	NA	0	NA
2c Fruit purée with cereal/milk	25	0	0	NA	0	NA
2d Vegetable purée with cereal/milk	5	0	0	NA	0	NA
2l Dairy with/without fruit	15	7	7	NA	0	0
2e Cheese meal purée	1	0	0	0[b]	0	NA
2f Fish meal purée	2	0	0	0[b]	0	NA
2g Meat meal purée	11	9	0	8[b]	0	NA
2h Meal other, spoonable	43	9	3	9[b]	0	NA
**3 Meals with chunky pieces**						
3a Meal meat or fish, vegetable tray/pot	2	0	0	0[b]	0	NA
**4 Dry finger foods and snacks**						
4d Savoury finger food	2	0	0	NA	0	NA
4a/b Sweet finger food and confectionary	7	43	43	NA	0	NA
4c Rusks and teething biscuits						
4e Fruit pieces/dried						
**5 Juices and drinks**						
5a Fruit drink	2	0	NA	0	0	0
Overall for all products	241	4	2		0	

NA = not applicable. Unable to determine amount of calcium in products.
Additional stipulations of European Commission regulations:

[a] ≥ 200 mg/100 kcal and ≥ 200 mg/100 g; or ≥ 300 mg/100 kcal if cheese meal; or only ≥ 100 mg/100 kcal if dry cereal.

Unable to determine whether sodium salts added to fruit, desserts or puddings except for technological purposes.

[b] Protein is sufficient in puréed meals with cheese in name if total protein is > 3 g/kcal. Protein is sufficient in puréed meals with a fish or meat first-named food if these are ≥ 10% of total weight of product and total protein is > 4 g/kcal. Protein is sufficient in other puréed meals if named protein is ≥ 8% of total weight of product and total protein is > 3 g/kcal. The calculation for meals with chunky pieces assumed a fish or meat is not first-named food – this may not be the case, meaning the percentage shown not meeting the criteria may be lower than actual.

[c] Total fat requirement is < 4.5 g/100 kcal for all products except dry cereals (< 3.3 g/100 kcal no added high protein) and cheese, fish or meat meals (6 g/100 kcal). Unable to determine lauric acid, myristc acid and linoleic acid content for which there are requirements when total fat content is over 3.3 g/100 kcal in cereals with high protein content.

[d] Total carbohydrates shall not exceed 20 g/100 fruit-only dishes, 25 g/100 g deserts and puddings, 10/100 g vegetable juices, 15 g/100 ml fruit juices, and 5 g/100 g other non-milk-based drinks. Unable to determine the amount of carbohydrate from fructose, glucose, glucose syrups or honey in dry cereal products.

Notes to Table 33–48

NA = not applicable; NPM = nutrient profile model; CACF = commercially available complementary foods for infants aged up to 36 months.

[a] The following listed ingredients have been classed as added sugars and sweeteners for this analysis: sugar, (any) syrup, juice (but lemon or lime juice are not), molasses, malt extract, barley malt, malted barley extract, maltose, dextrose, fructose, glucose, sucrose, honey.
Added lactose was not classed as an added sugar as it is a component of milk. Additionally, galacto-oligosaccharides, fructo-oligosaccharides, inulin, maltodextrose, maltodextrin and glycerol which are often found in breast-milk substitutes were not classed as sweeteners.
[b] Or < 100 mg sodium/100 kcal and 100 mg sodium/100 g if cheese meal; or only < 50 mg/100 kcal if dry cereal.
[c] Energy density requirements have not been set for the dry foods. Upper limits for energy density or portion size may be required for the finger foods.
[d] Protein is sufficient in puréed meals with cheese in name if total protein is ≥ 3 g/kcal. Protein is sufficient in puréed meals with a fish or meat first-named food if these are ≥ 15% of total weight of product and total protein is > 6 g/kcal. Protein is sufficient in other puréed meals if named protein is ≥ 10% of total weight of product and total protein is ≥ 3.75g/kcal. The calculation for meals with chunky pieces assumed a fish or meat is not first-named food – this may not be the case, meaning the percentage shown meeting the criteria may be lower than actual.
[e] Total fat requirement is < 4.5g/100 kcal for all products except dry cereals (< 3.3 g/100 kcal no added high protein) and cheese, fish or meat meals (6 g/100 kcal).
[f] Fruit drinks and vegetable drinks under the NPM proposals should not be marketed for infants and young children up to 36 months.

Colour coding
Percentages in purple indicate that modelling a 5% change in reformulation would increase the percentage of products that would meet the proposed NPM requirements.
Percentages in green indicate that modelling a 10% change in reformulation would increase the percentage of products that would meet the proposed NPM requirements.

Table 33. Percentage of commercially available complementary food (CACF) products available in the United Kingdom in 2016/2017 meeting proposed nutrient profile model (NPM) requirements

	Marketed in the United Kingdom in 2016/2017 for infants 4–36 months							
	N	Meets all six proposed NPM nutrient requirements	No added sugars/ sweeteners[a] (%)	Finger food < 15% total energy from total sugar (%)	Sodium < 50 mg/100 kcal and < 50 mg/100 g[b] (%)	Energy density > 60 kcal/100 g[c] (%)	Protein sufficient in meals[d] (%)	Fat not above current Commission regulations[e] (%)
**1 Dry, powdered and instant**								
1a Cereal, dry/instant	31	87	87	NA	100	NA	NA	100
1b Cereal with high added protein	48	58	65	NA	94	NA	NA	100
**2 Soft–wet spoonable**								
2a Fruit purée (with/without vegetables)	140	31	82	NA	99	40	NA	100
2b Vegetable purée	33	76	100	NA	76	NA	NA	100
2c Fruit purée with cereal/milk	54	41	56	NA	94	87	NA	100
2d Vegetable purée with cereal/milk	43	44	98	NA	72	60	NA	98
2l Dairy with/without fruit	26	35	38	NA	88	100	NA	100
2e Cheese meal purée	18	67	94	NA	72	94	100[d]	100
2f Fish meal purée	9	0	78	NA	44	89	0[d]	100
2g Meat meal purée	37	3	95	NA	62	51	3[d]	100
2h Meal other, spoonable	81	9	94	NA	77	81	25[d]	100
**3 Meals with chunky pieces**								
3a Meal meat or fish, vegetable tray/pot	89	14	82	NA	36	93	56[d]	86
**4 Dry finger foods and snacks**								
4d Savoury finger food	79	33	57	90	49	NA	NA	97
4a/b Sweet finger food and confectionary	39	0	3	0	51	NA	NA	100
4c Rusks and teething biscuits	19	5	5	5	47	NA	NA	95
4e Fruit pieces/dried	9	56	78	NA	78	NA	NA	56
**5 Juices and drinks**								
5a Fruit drink[f]	13	0	0	NA	62	NA	NA	100
Overall for all products	768	31	72		73			97

Table 34. Modelling 5% change towards proposed NPM requirements for CACF products available in the United Kingdom in 2016/2017

		Five of six requirements changed	Not changed: % reduction NA	Sugar content reduced by 5%	Sodium content reduced by 5%	Energy increased by 5%	Protein increased by 5%	Fat content reduced by 5%	Six changed, including no added sugars
	N	Meets all six proposed NPM nutrient requirements	No added sugars/ sweeteners[a] (%)	Finger food < 15% total energy from total sugar (%)	Sodium < 50 mg/100 kcal and < 50 mg/100 g[b] (%)	Energy density > 60 kcal/100 g[c] (%)	Protein sufficient in meals[d] (%)	Fat not above current Commission regulations[e] (%)	Meets all six proposed NPM nutrient requirements
**1 Dry, powdered and instant**									
1a Cereal, dry/instant	31	87	87	NA	100	NA	NA	100	100
1b Cereal with high added protein	48	58	65	NA	94	NA	NA	100	94
**2 Soft–wet spoonable**									
2a Fruit purée (with/without vegetables)	140	36	82	NA	99	47	NA	100	47
2b Vegetable purée	33	76	100	NA	76	NA	NA	100	76
2c Fruit purée with cereal/milk	54	41	56	NA	96	89	NA	100	85
2d Vegetable purée with cereal/milk	43	44	98	NA	72	60	NA	100	47
2l Dairy with/without fruit	26	35	38	NA	92	100	NA	100	92
2e Cheese meal purée	18	78	94	NA	83	100	100[d]	100	83
2f Fish meal purée	9	0	78	NA	56	89	0[d]	100	0
2g Meat meal purée	37	3	95	NA	68	62	3[d]	100	3
2h Meal other, spoonable	81	9	94	NA	80	86	25[d]	100	11
**3 Meals with chunky pieces**									
3a Meal meat or fish, vegetable tray/pot	89	20	82	NA	39	97	61[d]	91	24
**4 Dry finger foods and snacks**									
4d Savoury finger food	79	35	57	92	53	NA	NA	97	49
4a/b Sweet finger food and confectionary	39	0	3	3	51	NA	NA	100	0
4c Rusks and teething biscuits	19	5	5	11	47	NA	NA	100	
4e Fruit pieces/dried	9	56	78	NA	78	NA	NA	56	56
**5 Juices and drinks**									
5a Fruit drink[f]	13	0	0	NA	77	NA	NA	100	0
Overall for all products	768	33	72		75			98	45

Table 35. Modelling 10% change towards proposed NPM requirements for CACF products available in the United Kingdom in 2016/2017

		Five of six requirements changed	Not changed: % reduction NA	Sugar content reduced by 10%	Sodium content reduced by 10%	Energy increased by 10%	Protein increased by 10%	Fat content reduced by 10%	Six changed, including no added sugars
	N	Meets all six proposed NPM nutrient requirements	No added sugars/ sweeteners[a] (%)	Finger food < 15% total energy from total sugar (%)	Sodium < 50 mg/100 kcal and < 50 mg/100 g[b] (%)	Energy density > 60 kcal/100 g[c] (%)	Protein sufficient in meals[d] (%)	Fat not above current Commission regulations[e] (%)	Meets all six proposed NPM nutrient requirements
**1 Dry, powdered and instant**									
1a Cereal, dry/instant	31	87	87	NA	100	NA	NA	100	100
1b Cereal with high added protein	48	58	65	NA	94	NA	NA	100	94
**2 Soft–wet spoonable**									
2a Fruit purée (with/without vegetables)	140	45	82	NA	99	58	NA	100	57
2b Vegetable purée	33	76	100	NA	76	NA	NA	100	76
2c Fruit purée with cereal/milk	54	46	56	NA	96	91	NA	100	87
2d Vegetable purée with cereal/milk	43	47	98	NA	72	67	NA	100	49
2l Dairy with/without fruit	26	35	38	NA	96	100	NA	100	96
2e Cheese meal purée	18	78	94	NA	83	100	100[d]	100	83
2f Fish meal purée	9	0	78	NA	56	100	0[d]	100	0
2g Meat meal purée	37	3	95	NA	79	73	3[d]	100	3
2h Meal other, spoonable	81	16	94	NA	80	91	32[d]	100	19
**3 Meals with chunky pieces**									
3a Meal meat or fish, vegetable tray/pot	89	25	82	NA	43	99	63[d4]	96	28
**4 Dry finger foods and snacks**									
4d Savoury finger food	79	35	57	92	53	NA	NA	97	51
4a/b Sweet finger food and confectionary	39	0	3	3	51	NA	NA	100	0
4c Rusks and teething biscuits	19	5	5	11	47	NA	NA	100	5
4e Fruit pieces/dried	9	56	78	NA	78	NA	NA	56	56
**5 Juices and drinks**									
5a Fruit drink[f]	13	0	0	NA	77	NA	NA	100	0
Overall for all products	768	37	72		76			99	49

Table 36. Percentage of CACF products available in Denmark in 2016/2017 meeting proposed NPM requirements

	Marketed in Denmark in 2016/2017 for infants 4–36 months							
	N	Meets all six proposed NPM nutrient requirements	No added sugars/ sweeteners[a] (%)	Finger food < 15% total energy from total sugar (%)	Sodium < 50 mg/100 kcal and < 50 mg/100 g[b] (%)	Energy density > 60 kcal/100 g[c] (%)	Protein sufficient in meals[d] (%)	Fat not above current Commission regulations[e] (%)
**1 Dry, powdered and instant**								
1a Cereal, dry/instant	3	100	100	NA	100	NA	NA	100
1b Cereal with high added protein	45	56	67	NA	91	NA	NA	96
**2 Soft–wet spoonable**								
2a Fruit purée (with/without vegetables)	97	32	88	NA	98	42	NA	100
2b Vegetable purée	10	30	100	NA	30	NA	NA	90
2c Fruit purée with cereal/milk	47	43	53	NA	87	89	NA	100
2d Vegetable purée with cereal/milk	21	38	95	NA	67	67	NA	90
2l Dairy with/without fruit	5	40	100	NA	40	100	NA	60
2e Cheese meal purée	1	0	100	NA	0	0	100[d]	100
2f Fish meal purée	3	0	100	NA	33	100	33[d]	67
2g Meat meal purée	12	0	100	NA	83	50	0[d]	100
2h Meal other, spoonable	44	2	91	NA	64	100	14[d]	100
**3 Meals with chunky pieces**								
3a Meal meat or fish, vegetable tray/pot	1	0	100	NA	0	100	100[d]	100
**4 Dry finger foods and snacks**								
4d Savoury finger food	15	80	93	100	80	NA	NA	100
4a/b Sweet finger food and confectionary	15	13	20	13	67	NA	NA	100
4c Rusks and teething biscuits								
4e Fruit pieces/dried								
**5 Juices and drinks**								
5a Fruit drink[f]								
Overall for all products	319	34	79		82			97

Table 37. Modelling 5% change towards proposed NPM requirements for CACF products available in Denmark in 2016/2017

		Five of six requirements changed	Not changed: % reduction NA	Sugar content reduced by 5%	Sodium content reduced by 5%	Energy increased by 5%	Protein increased by 5%	Fat content reduced by 5%	Six changed, including no added sugars
	N	Meets all six proposed NPM nutrient requirements	No added sugars/ sweeteners[a] (%)	Finger food < 15% total energy from total sugar (%)	Sodium < 50 mg/100 kcal and < 50 mg/100 g[b] (%)	Energy density > 60 kcal/100 g[c] (%)	Protein sufficient in meals[d] (%)	Fat not above current Commission regulations[e] (%)	Meets all six proposed NPM nutrient requirements
**1 Dry, powdered and instant**									
1a Cereal, dry/instant	3	100	100	NA	100	NA	NA	100	100
1b Cereal with high added protein	45	58	67	NA	93	NA	NA	98	91
**2 Soft–wet spoonable**									
2a Fruit purée (with/without vegetables)	97	42	88	NA	98	54	NA	100	54
2b Vegetable purée	10	30	100	NA	40	NA	NA	90	30
2c Fruit purée with cereal/milk	47	45	53	NA	96	89	NA	100	85
2d Vegetable purée with cereal/milk	21	43	95	NA	67	67	NA	100	48
2l Dairy with/without fruit	5	40	100	NA	40	100	NA	100	40
2e Cheese meal purée	1	0	100	NA	0	0	100[d]	100	0
2f Fish meal purée	3	33	100	NA	33	100	67[d]	67	33
2g Meat meal purée	12	0	100	NA	92	50	0[d]	100	0
2h Meal other, spoonable	44	5	91	NA	66	100	14[d]	100	5
**3 Meals with chunky pieces**									
3a Meal meat or fish, vegetable tray/pot	1	0	100	NA	0	100	100[d]	100	0
**4 Dry finger foods and snacks**									
4d Savoury finger food	15	80	93	100	80	NA	NA	100	80
4a/b Sweet finger food and confectionary	15	13	20	20	67	NA	NA	100	13
4c Rusks and teething biscuits									
4e Fruit pieces/dried									
**5 Juices and drinks**									
5a Fruit drink[f]									
Overall for all products	319	38	79		84			99	53

Table 38. Modelling 10% change towards proposed NPM requirements for CACF products in Denmark in 2016/2017

	N	Five of six requirements changed	Not changed: % reduction NA	Sugar content reduced by 10%	Sodium content reduced by 10%	Energy increased by 10%	Protein increased by 10%	Fat content reduced by 10%	Six changed, including no added sugars
		Meets all six proposed NPM nutrient requirements	No added sugars/ sweeteners[a] (%)	Finger food < 15% total energy from total sugar (%)	Sodium < 50 mg/100 kcal and < 50 mg/100 g[b] (%)	Energy density > 60 kcal/100 g[c] (%)	Protein sufficient in meals[d] (%)	Fat not above current Commission regulations[e] (%)	Meets all six proposed NPM nutrient requirements
**1 Dry, powdered and instant**									
1a Cereal, dry/instant	3	100	100	NA	100	NA	NA	100	100
1b Cereal with high added protein	45	60	67	NA	93	NA	NA	100	93
**2 Soft–wet spoonable**									
2a Fruit purée (with/without vegetables)	97	51	88	NA	98	63	NA	100	62
2b Vegetable purée	10	30	100	NA	40	NA	NA	90	30
2c Fruit purée with cereal/milk	47	47	53	NA	96	98	NA	100	94
2d Vegetable purée with cereal/milk	21	48	95	NA	67	76	NA	100	52
2l Dairy with/without fruit	5	80	100	NA	80	100	NA	100	80
2e Cheese meal purée	1	0	100	NA	0	0	100[d]	100	0
2f Fish meal purée	3	33	100	NA	33	100	67[d]	67	33
2g Meat meal purée	12	0	100	NA	92	67	0[d]	100	0
2h Meal other, spoonable	44	7	91	NA	68	100	16[d]	100	7
**3 Meals with chunky pieces**									
3a Meal meat or fish, vegetable tray/pot	1	0	100	NA	0	100	100[d]	100	0
**4 Dry finger foods and snacks**									
4d Savoury finger food	15	80	93	100	80	NA	NA	100	80
4a/b Sweet finger food and confectionary	15	13	20	33	67	NA	NA	100	13
4c Rusks and teething biscuits									
4e Fruit pieces/dried									
**5 Juices and drinks**									
5a Fruit drink[f]									
Overall for all products	319	43	79		85			99	58

Table 39. Percentage of CACF products available in Spain in 2017 meeting proposed NPM requirements

	Marketed in Spain in 2017 for infants 4–36 months							
	N	Meets all six proposed NPM nutrient requirements	No added sugars/ sweeteners[a] (%)	Finger food < 15% total energy from total sugar (%)	Sodium < 50 mg/100 kcal and < 50 mg/100 g[b] (%)	Energy density > 60 kcal/100 g[c] (%)	Protein sufficient in meals[d] (%)	Fat not above current Commission regulations[e] (%)
**1 Dry, powdered and instant**								
1a Cereal, dry/instant	74	50	50	NA	99	NA	NA	100
1b Cereal with high added protein	8	0	38	NA	50	NA	NA	100
**2 Soft–wet spoonable**								
2a Fruit purée (with/without vegetables)	41	22	39	NA	100	76	NA	100
2b Vegetable purée	3	67	67	NA	67	NA	NA	100
2c Fruit purée with cereal/milk	25	28	28	NA	100	92	NA	100
2d Vegetable purée with cereal/milk	5	60	100	NA	80	80	NA	100
2l Dairy with/without fruit	15	27	27	NA	93	100	NA	100
2e Cheese meal purée	1	100	100	NA	100	100	100[d]	100
2f Fish meal purée	2	0	100	NA	50	100	0[d]	100
2g Meat meal purée	11	0	100	NA	46	100	0[d]	100
2h Meal other, spoonable	43	14	100	NA	42	77	47[d]	100
**3 Meals with chunky pieces**								
3a Meal meat or fish, vegetable tray/pot	2	0	50	NA	0	100	100[d]	100
**4 Dry finger foods and snacks**								
4d Savoury finger food	2	100	100	100	100	NA	NA	100
4a/b Sweet finger food and confectionary	7	0	0	0	14	NA	NA	100
4c Rusks and teething biscuits								
4e Fruit pieces/dried	0			NA				
**5 Juices and drinks**								
5a Fruit drink[f]	2	0	0	NA	100	NA	NA	100
Overall for all products	241	29	56		80			100

Table 40. Modelling 5% change towards proposed NPM requirements for CACF products available in Spain in 2017

		Five of six requirements changed	Not changed: % reduction NA	Sugar content reduced by 5%	Sodium content reduced by 5%	Energy increased by 5%	Protein increased by 5%	Fat content reduced by 5%	Six changed, including no added sugars
	N	Meets all six proposed NPM nutrient requirements	No added sugars/ sweeteners[a] (%)	Finger food < 15% total energy from total sugar (%)	Sodium < 50 mg/100 kcal and < 50 mg/100 g[b] (%)	Energy density > 60 kcal/100 g[c] (%)	Protein sufficient in meals[d] (%)	Fat not above current Commission regulations[e] (%)	Meets all six proposed NPM nutrient requirements
**1 Dry, powdered and instant**									
1a Cereal, dry/instant	74	50	50	NA	100	NA	NA	100	100
1b Cereal with high added protein	8	0	38	NA	50	NA	NA	100	50
**2 Soft–wet spoonable**									
2a Fruit purée (with/without vegetables)	41	27	39	NA	100	81	NA	100	81
2b Vegetable purée	3	67	67	NA	67	NA	NA	100	67
2c Fruit purée with cereal/milk	25	28	28	NA	100	92	NA	100	92
2d Vegetable purée with cereal/milk	5	60	100	NA	80	80	NA	100	60
2l Dairy with/without fruit	15	27	27	NA	93	100	NA	100	93
2e Cheese meal purée	1	100	100	NA	100	100	100[d]	100	100
2f Fish meal purée	2	0	100	NA	50	100	0[d]	100	0
2g Meat meal purée	11	0	100	NA	46	100	9[d]	100	0
2h Meal other, spoonable	43	14	100	NA	44	86	47[d]	100	14
**3 Meals with chunky pieces**									
3a Meal meat or fish, vegetable tray/pot	2	0	50	NA	0	100	100[d]	100	0
**4 Dry finger foods and snacks**									
4d Savoury finger food	2	100	100	100	100	NA	NA	100	100
4a/b Sweet finger food and confectionary	7	0	0	0	14	NA	NA	100	0
4c Rusks and teething biscuits									
4e Fruit pieces/dried	0			NA					
**5 Juices and drinks**									
5a Fruit drink[f]	2	0	0	NA	100	NA	NA	100	100
Overall for all products	241	30	56		81			100	67

Table 41. Modelling 10% change towards proposed NPM requirements for CACF products available in Spain in 2016/2017

		Five of six requirements changed	Not changed: % reduction NA	Sugar content reduced by 10%	Sodium content reduced by 10%	Energy increased by 10%	Protein increased by 10%	Fat content reduced by 10%	Six changed, including no added sugars
	N	Meets all six proposed NPM nutrient requirements	No added sugars/ sweeteners[a] (%)	Finger food < 15% total energy from total sugar (%)	Sodium < 50 mg/100 kcal and < 50 mg/100 g[b] (%)	Energy density > 60 kcal/100 g[c] (%)	Protein sufficient in meals[d] (%)	Fat not above current Commission regulations[e] (%)	Meets all six proposed NPM nutrient requirements
**1 Dry, powdered and instant**									
1a Cereal, dry/instant	74	50	50	NA	100	NA	NA	100	100
1b Cereal with high added protein	8	0	38	NA	50	NA	NA	100	50
**2 Soft–wet spoonable**									
2a Fruit purée (with/without vegetables)	41	27	39	NA	100	85	NA	100	85
2b Vegetable purée	3	67	67	NA	67	NA	NA	100	67
2c Fruit purée with cereal/milk	25	28	28	NA	100	100	NA	100	100
2d Vegetable purée with cereal/milk	5	60	100	NA	80	80	NA	100	60
2l Dairy with/without fruit	15	27	27	NA	93	100	NA	100	93
2e Cheese meal purée	1	100	100	NA	100	100	100[d]	100	100
2f Fish meal purée	2	0	100	NA	50	100	0[d]	100	0
2g Meat meal purée	11	0	100	NA	55	100	9[d]	100	0
2h Meal other, spoonable	43	14	100	NA	44	93	47[d]	100	14
**3 Meals with chunky pieces**									
3a Meal meat or fish, vegetable tray/pot	2	0	50	NA	0	100	100[d]	100	0
**4 Dry finger foods and snacks**									
4d Savoury finger food	2	100	100	100	100	NA	NA	100	100
4a/b Sweet finger food and confectionary	7	0	0	0	14	NA	NA	100	0
4c Rusks and teething biscuits									
4e Fruit pieces/dried	0			NA					
**5 Juices and drinks**									
5a Fruit drink[f]	2	0	0	NA	100	NA	NA	100	100
Overall for all products	241	30	56		81			100	69

Table 42. Percentage of CACF products available in Estonia in 2018 meeting proposed NPM requirements

	Percentage of products meeting NPM requirements							
Food category	Total number of products	Meets all six proposed NPM nutrient requirements (%)	No added sugars/ sweeteners[a] (%)	Finger food < 15% total energy from total sugar (%)	Sodium < 50 mg/100 kcal and < 50 mg/100 g[b] (%)	Energy density > 60 kcal/100 g[c] (%)	Protein sufficient in meals[d] (%)	Fat not above current regulations[e] (%)
1a Dry instant cereals	10	80	90	NA	100	NA	NA	90
1b Dry cereals (with high protein food)	4	50	50	NA	50	NA	NA	100
2a Fruit purée (with or without veg)	38	39	89	NA	100	47	NA	100
2b Vegetable purées	11	82	100	NA	82	NA	NA	100
2c Fruit purée with cereal or milk	25	48	52	NA	96	100	NA	100
2d Vegetables with cereal, soft–wet spoonable	2	0	100	NA	100	0	NA	100
2e Cheese meal, soft–wet spoonable	0	–	–	NA	–	–	–	–
2f Fish meal, soft–wet spoonable	2	0	100	NA	50	50	50	100
2g Meat/poultry meal, soft–wet spoonable	1	0	100	NA	100	0	100	100
2h Meal other, soft–wet spoonable	15	0	100	NA	87	80	0	87
2i Dairy, soft-wet spoonable	1	100	100	NA	100	100	NA	100
2j Meat only purée	3	100	100	NA	100	NA	100	100
2k Fish only purée	0	–	–	NA	–	NA	–	–
3a Tray/pot chunky meat or fish meal	6	33	100	NA	83	NA	50	100
3b Tray /pot chunky vegetable meal	0	–	–	NA	–	NA	–	–
4d Savoury snacks	1	100	100	100	100	NA	NA	100
4a Confectionery and bars	0	–	–	–	–	NA	NA	–
4b Sweet snacks	6	17	17	33	83	NA	NA	100
4c Rusks and teething biscuits	3	67	67	100	67	NA	NA	100
4e Fruit snacks	0	–	–	–	–	NA	NA	–
5a Fruit juices and drinks[f]	6	0	0	NA	100	NA	NA	100
5b Vegetable juices	0	–	–	NA	–	NA	NA	–
5c Other drinks non-milk/non-formula	0	–	–	NA	–	NA	NA	–
Total percentage of applicable products passing	134	42	77	60	92	68	30	98

Table 43. Percentage of CACF products available in Hungry in 2018 meeting proposed NPM requirements

Food category	Total number of products	Percentage of products meeting NPM requirements						
		Meets all six proposed NPM nutrient requirements (%)	No added sugars/ sweeteners[a] (%)	Finger food < 15% total energy from total sugar (%)	Sodium < 50 mg/100 kcal and < 50 mg/100 g[b] (%)	Energy density > 60 kcal/100 g[c] (%)	Protein sufficient in meals[d] (%)	Fat not above current regulations[e] (%)
1a Dry instant cereals	23	61	61	NA	96	NA	NA	100
1b Dry cereals (with high protein food)	0	–	–	NA	–	NA	NA	–
2a Fruit purée (with or without veg)	13	0	31	NA	100	31	NA	100
2b Vegetable purées	0	–	–	NA	–	NA	NA	–
2c Fruit purée with cereal or milk	31	3	32	NA	100	61	NA	100
2d Vegetables with cereal, soft–wet spoonable	1	0	100	NA	0	100	NA	100
2i Dairy, soft-wet spoonable	9	33	33	NA	100	100	NA	100
2e Cheese meal, soft–wet spoonable	0	–	–	NA	–	–	–	–
2f Fish meal, soft–wet spoonable	0	–	–	NA	–	–	–	–
2g Meat/poultry meal, soft–wet spoonable	0	–	–	NA	–	–	–	–
2h Meal other, soft–wet spoonable	16	0	100	NA	75	63	6	100
2j Meat only purée	1	100	100	NA	100	NA	100	100
2k Fish only purée	0	–	–	NA	–	NA	–	–
3a Tray/pot chunky meat or fish meal	0	–	–	NA	–	NA	–	–
3b Tray /pot chunky vegetable meal	0	–	–	NA	–	NA	–	–
4d Savoury snacks	0	–	–	–	–	NA	NA	–
4a Confectionery and bars	0	–	–	–	–	NA	NA	–
4b Sweet snacks	14	0	21	14	50	NA	NA	100
4c Rusks and teething biscuits	0	–	–	–	–	NA	NA	–
4e Fruit snacks	0	–	–	–	–	NA	NA	–
5a Fruit juices and drinks[f]	15	0	0	NA	67	NA	NA	100
5b Vegetable juices	0	–	–	NA	–	NA	NA	–
5c Other drinks non-milk/non-formula	0	–	–	NA	–	NA	NA	–
Total percentage of applicable products passing	123	15	42	14	85	61	12	100

Table 44. Percentage of CACF products available in Malta in 2018 meeting proposed NPM requirements

		Percentage of products meeting NPM requirements						
Food category	Total number of products	Meets all six proposed NPM nutrient requirements (%)	No added sugars/ sweeteners[a] (%)	Finger food < 15% total energy from total sugar (%)	Sodium < 50 mg/100 kcal and < 50 mg/100 g[b] (%)	Energy density > 60 kcal/100 g[c] (%)	Protein sufficient in meals[d] (%)	Fat not above current regulations[e] (%)
1a Dry instant cereals	18	100	100	NA	100	NA	NA	100
1b Dry cereals (with high protein food)	20	45	55	NA	90	NA	NA	100
2a Fruit purée (with or without veg)	53	42	89	NA	100	51	NA	100
2b Vegetable purées	17	47	100	NA	47	NA	NA	94
2c Fruit purée with cereal or milk	12	67	83	NA	92	92	NA	92
2d Vegetables with cereal, soft–wet spoonable	8	13	88	NA	63	25	NA	100
2i Dairy, soft-wet spoonable	17	35	41	NA	88	100	NA	100
2e Cheese meal, soft–wet spoonable	3	67	100	NA	67	100	100	100
2f Fish meal, soft–wet spoonable	5	0	100	NA	60	100	0	100
2g Meat/poultry meal, soft–wet spoonable	8	25	100	NA	100	38	25	100
2h Meal other, soft–wet spoonable	28	4	96	NA	79	64	21	86
2j Meat only purée	8	25	100	NA	100	NA	25	100
2k Fish only purée	0	–	–	NA	–	NA	–	–
3a Tray/pot chunky meat or fish meal	1	0	0	NA	0	NA	100	100
3b Tray /pot chunky vegetable meal	1	100	100	NA	100	NA	NA	100
4a Confectionery and bars	1	0	0	0	0	NA	NA	100
4d Savoury snacks	10	30	80	100	40	NA	NA	80
4b Sweet snacks	31	16	23	35	77	NA	NA	90
4c Rusks and teething biscuits	1	0	0	0	100	NA	NA	100
4e Fruit snacks	1	0	100	0	0	NA	NA	100
5a Fruit juices and drinks[f]	0	–	–	NA	–	NA	NA	–
5b Vegetable juices	0	–	–	NA	–	NA	NA	–
5c Other drinks non-milk/non-formula	0	–	–	NA	–	NA	NA	–
Total percentage of applicable products passing	243	36	76	48	83	64	26	95

Table 45. Percentage of CACF products available in Norway in 2018 meeting proposed NPM requirements

Food category	Total number of products	Percentage of products meeting NPM requirements						
		Meets all six proposed NPM nutrient requirements (%)	No added sugars/ sweeteners[a] (%)	Finger food < 15% total energy from total sugar (%)	Sodium < 50 mg/100 kcal and < 50 mg/100 g[b] (%)	Energy density > 60 kcal/100 g[c] (%)	Protein sufficient in meals[d] (%)	Fat not above current regulations[e] (%)
1a Dry instant cereals	0	–	–	NA	–	NA	NA	–
1b Dry cereals (with high protein food)	17	71	71	NA	100	NA	NA	100
2a Fruit purée (with or without veg)	22	50	82	NA	86	59	NA	100
2b Vegetable purées	3	33	100	NA	33	NA	NA	100
2c Fruit purée with cereal or milk	11	36	55	NA	82	91	NA	100
2d Vegetables with cereal, soft–wet spoonable	5	40	100	NA	60	80	NA	100
2i Dairy, soft-wet spoonable	7	14	14	NA	43	100	NA	100
2e Cheese meal, soft–wet spoonable	2	50	100	NA	50	100	100	100
2f Fish meal, soft–wet spoonable	1	0	100	NA	0	100	0	100
2g Meat/poultry meal, soft–wet spoonable	7	0	100	NA	14	100	0	100
2h Meal other, soft–wet spoonable	12	17	100	NA	50	83	17	100
2j Meat only purée	0	–	–	NA	–	NA	–	–
2k Fish only purée	0	–	–	NA	–	NA	–	–
3a Tray/pot chunky meat or fish meal	0	–	–	NA	–	NA	–	–
3b Tray /pot chunky vegetable meal	0	–	–	NA	–	NA	–	–
4d Savoury snacks	5	60	60	100	80	NA	NA	80
4a Confectionery and bars	5	0	20	0	100	NA	NA	100
4b Sweet snacks	6	17	33	50	83	NA	NA	100
4c Rusks and teething biscuits	0	–	–	–	–	NA	NA	–
4e Fruit snacks	0	–	–	–	–	NA	NA	–
5a Fruit juices and drinks[f]	4	0	0	NA	75	NA	NA	100
5b Vegetable juices	0	–	–	NA	–	NA	NA	–
5c Other drinks non-milk/non-formula	0	–	–	NA	–	NA	NA	–
Total percentage of applicable products passing	107	36	68	50	72	81	18	99

Table 46. Percentage of CACF products available in Portugal in 2018 meeting proposed NPM requirements

Food category	Total number of products	Percentage of products meeting NPM requirements						
		Meets all six proposed NPM nutrient requirements (%)	No added sugars/ sweeteners[a] (%)	Finger food < 15% total energy from total sugar (%)	Sodium < 50 mg/100 kcal and < 50 mg/100 g[b] (%)	Energy density > 60 kcal/100 g[c] (%)	Protein sufficient in meals[d] (%)	Fat not above current regulations[e] (%)
1a Dry instant cereals	25	64	64	NA	100	NA	NA	100
1b Dry cereals (with high protein food)	16	56	56	NA	100	NA	NA	100
2a Fruit purée (with or without veg)	25	36	64	NA	84	68	NA	100
2b Vegetable purées	1	0	100	NA	0	NA	NA	100
2c Fruit purée with cereal or milk	12	17	25	NA	92	92	NA	100
2d Vegetables with cereal, soft–wet spoonable	4	25	100	NA	25	50	NA	50
2i Dairy, soft-wet spoonable	12	17	33	NA	83	100	NA	100
2e Cheese meal, soft–wet spoonable	2	0	100	NA	100	100	0	100
2f Fish meal, soft–wet spoonable	0	–	–	NA	–	–	–	–
2g Meat/poultry meal, soft–wet spoonable	8	0	100	NA	63	100	0	100
2h Meal other, soft–wet spoonable	7	14	100	NA	43	71	29	86
2j Meat only purée	0	–	–	NA	–	NA	–	–
2k Fish only purée	0	–	–	NA	–	NA	–	–
3a Tray/pot chunky meat or fish meal	2	0	50	NA	0	NA	0	100
3b Tray /pot chunky vegetable meal	0	–	–	NA	–	NA	–	–
4d Savoury snacks	0	–	–	–	–	NA	NA	–
4a Confectionery and bars	0	–	–	–	–	NA	NA	–
4b Sweet snacks	3	0	0	33	67	NA	NA	100
4c Rusks and teething biscuits	7	14	14	57	29	NA	NA	100
4e Fruit snacks	0	–	–	–	–	NA	NA	–
5a Fruit juices and drinks[f]	1	0	0	NA	100	NA	NA	100
5b Vegetable juices	0	–	–	NA	–	NA	NA	–
5c Other drinks non-milk/non-formula	0	–	–	NA	–	NA	NA	–
Total percentage of applicable products passing	125	33	58	50	79	81	11	98

Table 47. Percentage of CACF products available in Slovenia in 2018 meeting proposed NPM requirements

		Percentage of products meeting NPM requirements						
Food category	Total number of products	Meets all six proposed NPM nutrient requirements (%)	No added sugars/ sweeteners[a] (%)	Finger food < 15% total energy from total sugar (%)	Sodium < 50 mg/100 kcal and < 50 mg/100 g[b] (%)	Energy density > 60 kcal/100 g[c] (%)	Protein sufficient in meals[d] (%)	Fat not above current regulations[e] (%)
1a Dry instant cereals	8	63	63	NA	100	NA	NA	100
1b Dry cereals (with high protein food)	19	5	11	NA	95	NA	NA	95
2a Fruit purée (with or without veg)	14	29	64	NA	100	50	NA	100
2b Vegetable purées	6	83	100	NA	83	NA	NA	100
2c Fruit purée with cereal or milk	23	30	39	NA	91	87	NA	100
2d Vegetables with cereal, soft–wet spoonable	8	13	100	NA	38	75	NA	100
2i Dairy, soft-wet spoonable	17	6	12	NA	82	100	NA	100
2e Cheese meal, soft–wet spoonable	2	50	100	NA	50	100	100	100
2f Fish meal, soft–wet spoonable	0	–	–	NA	–	–	–	–
2g Meat/poultry meal, soft–wet spoonable	1	100	100	NA	100	100	100	100
2h Meal other, soft–wet spoonable	17	6	100	NA	53	94	12	100
2j Meat only purée	1	100	100	NA	100	NA	100	100
2k Fish only purée	0	–	–	NA	–	NA	–	–
3a Tray/pot chunky meat or fish meal	5	0	100	NA	0	NA	0	100
3b Tray /pot chunky vegetable meal	2	0	100	NA	0	NA	0	100
4d Savoury snacks	9	67	67	100	89	NA	NA	100
4a Confectionery and bars	0	–	–	–	–	NA	NA	–
4b Sweet snacks	6	0	0	0	33	NA	NA	100
4c Rusks and teething biscuits	6	0	17	17	33	NA	NA	67
4e Fruit snacks	1	100	100	0	100	NA	NA	100
5a Fruit juices and drinks[f]	6	0	0	NA	50	NA	NA	100
5b Vegetable juices	1	0	100	NA	0	NA	NA	100
5c Other drinks non-milk/non-formula	0	–	–	NA	–	NA	NA	–
Total percentage of applicable products passing	152	23	51	45	73	84	21	98

Table 48. Percentage of CACF products available in Italy in 2018 meeting proposed NPM requirements

Food category	Total number of products	Percentage of products meeting NPM requirements						
		Meets all six proposed NPM nutrient requirements (%)	No added sugars/ sweeteners[a] (%)	Finger food < 15% total energy from total sugar (%)	Sodium < 50 mg/100 kcal and < 50 mg/100 g[b] (%)	Energy density > 60 kcal/100 g[c] (%)	Protein sufficient in meals[d] (%)	Fat not above current regulations[e] (%)
1a Dry instant cereals	75	96	96	NA	100	NA	NA	100
1b Dry cereals (with high protein food)	13	38	38	NA	100	NA	NA	100
2a Fruit purée (with or without veg)	60	30	65	NA	100	53	NA	100
2b Vegetable purées	23	52	96	NA	52	NA	NA	100
2c Fruit purée with cereal or milk	41	20	24	NA	100	78	NA	100
2d Vegetables with cereal, soft–wet spoonable	0	–	–	NA	–	–	NA	–
2i Dairy, soft–wet spoonable	54	0	0	NA	93	100	NA	100
2e Cheese meal, soft–wet spoonable	18	39	100	NA	39	94	100	100
2f Fish meal, soft–wet spoonable	20	15	100	NA	65	75	35	100
2g Meat/poultry meal, soft–wet spoonable	25	28	100	NA	84	96	40	100
2h Meal other, soft–wet spoonable	9	0	100	NA	44	89	11	100
2j Meat only purée	25	8	100	NA	80	NA	8	100
2k Fish only purée	0	–	–	NA	–	NA	–	–
3a Tray/pot chunky meat or fish meal	4	100	100	NA	100	NA	100	100
3b Tray /pot chunky vegetable meal	0	–	–	NA	–	NA	–	–
4d Savoury snacks	4	50	50	100	50	NA	NA	100
4a Confectionery and bars	0	–	–	–	–	NA	NA	–
4b Sweet snacks	6	0	0	0	0	NA	NA	83
4c Rusks and teething biscuits	27	0	0	4	7	NA	NA	100
4e Fruit snacks	0	–	–	–	–	NA	NA	–
5a Fruit juices and drinks[f]	10	0	0	NA	80	NA	NA	100
5c Other drinks non-milk/non-formula	0	–	–	NA	–	NA	NA	–
6a Dry instant meat/fish	12	0	100	NA	8	NA	NA	0
6b Dry instant vegetable	4	0	75	NA	0	NA	NA	0
Total percentage of applicable products passing	430	33	62	14	77	80	42	96

References[4]

1. Resolution WHA69.9. Ending inappropriate promotion of foods for infants and young children. Sixty-ninth World Health Assembly agenda item 12.1. Geneva: World Health Organization; 2016 (http://apps.who.int/gb/ebwha/pdf_files/WHA69/A69_R9-en.pdf).
2. Pan American Health Organization, World Health Organization. Guiding principles for complementary feeding of the breastfed child. Washington: Pan American Health Organization; 2003 (https://www.ennonline.net/compfeedingprinciples).
3. The optimal duration of exclusive breastfeeding. Report of an expert consultation. Geneva: World Health Organization; 2002 (http://apps.who.int/iris/handle/10665/67219).
4. Feeding in the first year of life: draft SACN report [website]. London: Public Health England; 2017 (https://www.gov.uk/government/consultations/feeding-in-the-first-year-of-life-draft-sacn-report).
5. Your baby's first solid foods. In: NHS Choices [website]. London: NHS Choices; 2018 (https://www.nhs.uk/Conditions/pregnancy-and-baby/Pages/solid-foods-weaning.aspx).
6. EFSA Panel on Dietetic Products, Nutrition and Allergies (NDA). Scientific opinion on the appropriate age for introduction of complementary feeding of infants. EFSA Journal 2009;7(12):1423.
7. Fewtrell M, Bronsky J, Campoy C, Domellöf M, Embleton N, Fidler Mis N et al. Complementary feeding: a position paper by the European Society for Paediatric Gastroenterology, Hepatology, and Nutrition (ESPGHAN) Committee on Nutrition. J Pediatr Gastroenterol Nutr. 2017;64(1):119–32.
8. Complementary feeding of young children in developing countries: a review of current scientific knowledge. Geneva: World Health Organization; 1998 (http://www.who.int/nutrition/publications/infantfeeding/WHO_NUT_98.1/en/).
9. Smith HA, Becker GE. Early additional food and fluids for healthy breastfed full-term infants. Cochrane Database Syst Rev. 2016(8).
10. Kramer MS, Kakuma R. Optimal duration of exclusive breastfeeding. Cochrane Database Syst Rev. 2012;(8): CD006462. doi:10.1002/14651858.CD006462.pub4.
11. Lennox A, Sommerville J, Ong K, Henderson H, Allen R, editors. Diet and nutrition survey of infants and young children, 2011. London: Department of Health and Social Care; 2013 (https://www.gov.uk/government/publications/diet-and-nutrition-survey-of-infants-and-young-children-2011).
12. Garcia AL, Raza S, Parrett A, Wright CM. Nutritional content of infant commercial weaning foods in the UK. Arch Dis Child. 2013;98(10):793–7.
13. Boyle RJ, Garcia-Larsen V, Ierodiakonou D, Leonard-Bee J, Reeves T, Trivella M et al. Review of scientific published literature on infant feeding and development of atopic and autoimmune disease. Review B: timing of introduction of allergenic foods to the infant diet. London: Imperial Consultants; 2016 (https://www.food.gov.uk/sites/default/files/fs305005breport.pdf).
14. Daniels L, Mallan KM, Fildes A, Wilson J. The timing of solid introduction in an "obesogenic" environment: a narrative review of the evidence and methodological issues. Aust N Z J Public Health 2015;39(4):366–73.
15. Hetherington MM, Cecil JE, Jackson DM, Schwartz C. Feeding infants and young children. From guidelines to practice. Appetite 2011;57(3):791–5.
16. Ventura AK, Worobey J. Early influences on the development of food preferences. Curr Biol. 2013;23(9): R401-8. doi:10.1016/j.cub.2013.02.037.
17. Mura Paroche M, Caton SJ, Vereijken C, Weenen H, Houston-Price C. How infants and young children learn about food: a systematic review. Front Psychol. 2017;8:1046.
18. Anzman-Frasca S, Ventura AK, Ehrenberg S, Myers KP. Promoting healthy food preferences from the start: a narrative review of food preference learning from the prenatal period through early childhood. Obes Rev. 2018;19(4):576–604.
19. Harris G, Coulthard H. Early eating behaviours and food acceptance revisited: breastfeeding and introduction of complementary foods as predictive of food acceptance. Curr Obes Rep. 2016;5(1):113–20.
20. Nehring I, Kostka T, von Kries R, Rehfuess EA. Impacts of in utero and early infant taste experiences on later taste acceptance: a systematic review. J Nutr. 2015;145(6):1271–9.

[4] All weblinks accessed 12 March 2019.

21. Nicklaus S. Children's acceptance of new foods at weaning. Role of practices of weaning and of food sensory properties. Appetite 2011;57(3):812–5.
22. Coulthard H, Harris G, Emmett P. Long-term consequences of early fruit and vegetable feeding practices in the United Kingdom. Public Health Nutr. 2010;13(12):2044–51.
23. Elliott CD. Sweet and salty: nutritional content and analysis of baby and toddler foods. J Public Health (Oxf.) 2011;33(1):63–70.
24. Thow AM, Hawkes C. Global sugar guidelines: an opportunity to strengthen nutrition policy. Public Health Nutr. 2014;17(10):2151–5.
25. Crawley H, Westland S. Baby foods in the UK. A review of commercially produced jars and pouches of baby foods marketed in the UK. London: First Steps Nutrition Trust; 2017 (http://www.firststepsnutrition.org/pdfs/Baby_Food_in_the_UK%20_2017.pdf).
26. Dunford E, Louie JC, Byrne R, Walker KZ, Flood VM. The nutritional profile of baby and toddler food products sold in Australian supermarkets. Matern Child Health J 2015;19(12):2598–604.
27. Garcia AL, McLean K, Wright CM. Types of fruits and vegetables used in commercial baby foods and their contribution to sugar content. Matern Child Nutr. 2016;12(4):838–47.
28. Foterek K, Buyken AE, Bolzenius K, Hilbig A, Nöthlings U, Alexy U. Commercial complementary food consumption is prospectively associated with added sugar intake in childhood. Br J Nutr. 2016;115(11):2067–74.
29. Carstairs SA, Craig LC, Marais D, Bora QE, Kizebrink K. A comparison of preprepared commercial infant feeding meals with home-cooked recipes. Arch Dis Child. 2016;101(11):1037–42.
30. Mesch CM, Stimming M, Foterek K, Hilbig A, Alexy U, Kersting M et al. Food variety in commercial and homemade complementary meals for infants in Germany. Market survey and dietary practice. Appetite 2014;76:113–9.
31. Sheiham A, James WP. Diet and dental caries: the pivotal role of free sugars reemphasized. J Dent Res. 2015;94(10):1341–7.
32. Rapley G. Baby-led weaning: transitioning to solid foods at the baby's own pace. Community Pract. 2011;84(6):20–3.
33. Taylor RW, Williams SM, Fangupo LJ, Wheeler BJ, Taylor BJ, Daniels L et al. Effect of a baby-led approach to complementary feeding on infant growth and overweight: a randomized clinical trial. JAMA Pediatr. 2017;171(9):838–46.
34. Cichero JAY. Introducing solid foods using baby-led weaning vs. spoon-feeding: a focus on oral development, nutrient intake and quality of research to bring balance to the debate. Nutr Bull. 2016;41(1):72–7.
35. Le Reverend BJ, Edelson LR, Loret C. Anatomical, functional, physiological and behavioural aspects of the development of mastication in early childhood. Br J Nutr. 2014;111(3):403–14.
36. Coulthard H, Harris G, Emmett P. Delayed introduction of lumpy foods to children during the complementary feeding period affects child's food acceptance and feeding at 7 years of age. Matern Child Nutr. 2009;5(1):75–85.
37. Fangupo LJ, Heath A-LM, Williams SM, Erickson Williams LW, Morison BJ, Fleming EA et al. A baby-led approach to eating solids and risk of choking. Pediatrics 2016;138(4): pii: e20160772.
38. Guiding principles for feeding non-breastfed children 6–24 months of age. Geneva: World Health Organization; 2005 (https://www.who.int/maternal_child_adolescent/documents/9241593431/en/).
39. Koletzko B, Hirsch NL, Jewell JM, Caroli M, Breda JR, Weber M. Pureed fruit pouches for babies: child health under squeeze. J Pediatr Gastroenterol Nutr. 2018;67(5):561–3. doi:10.1097/MPG.0000000000002061.
40. Tzioumis E, Kay M, Wright M, Adair L. Health effects of commercially available complementary foods: a systematic review. Chapel Hill: Gillings School of Global Public Health, University of North Carolina; 2015 (http://www.who.int/nutrition/topics/CF_health_effects_commercially_systematicreview.pdf).
41. Cogswell ME, Gunn JP, Yuan K, Park S, Merritt R. Sodium and sugar in complementary infant and toddler foods sold in the United States. Pediatrics 2015;135(31):416–23.
42. Haigh C, Schneider J. Junk food for babies? An investigation into foods marketed for babies and young children. London: Sustain; 2009 (https://www.sustainweb.org/pdf/CFC_Baby_food_report.pdf).

43. Fitzhugh K, Lobstein TL. Children's food examined: an analysis of 358 products targeted at children. London: The Food Commission (UK) Ltd.; 2000 (http://www.foodcomm.org.uk/pdfs/Childrens_Food_Examined.pdf).
44. Zand N, Chowdhry BZ, Pollard LV, Pullen FS, Snowden MJ, Zotor FB. Commercial 'ready-to-feed' infant foods in the UK: macro-nutrient content and composition. Matern Child Nutr. 2015;11(2):202–14.
45. The Commission of the European Communities. Commission Directive 2006/125/EC on processed cereal-based foods and baby foods for infants and young children. O. J. E. U. 2006, L 339:16–35 (http://eur-lex.europa.eu/legal-content/EN/TXT/PDF/?uri=CELEX:32006L0125&from=EN).
46. Zand N, Chowdhry BZ, Zotor FB, Wray DS, Amuna P, Pullen FS. Essential and trace elements content of commercial infant foods in the UK. Food Chem. 2011;128(1):123–8.
47. Zand N, Chowdhry BZ, Wray DS, Pullen FS, Snowden MJ. Elemental content of commercial "ready to-feed" poultry and fish based infant foods in the UK. Food Chem. 2012;135(4):2796–801.
48. Loughrill E, Govinden P, Zand N. Vitamins A and E content of commercial infant foods in the UK: a cause for concern? Food Chem. 2016;210:56–62.
49. Mok E, Vanstone CA, Gallo S, Li P, Constantin E, Weiler HA. Diet diversity, growth and adiposity in healthy breastfed infants fed homemade complementary foods. Int J Obes (Lond.) 2017;41:776.
50. Verger EO, Eussen S, Holmes BA. Evaluation of a nutrient-based diet quality index in UK young children and investigation into the diet quality of consumers of formula and infant foods. Public Health Nutr. 2016;19(10):1785–94.
51. Hilbig A, Foterek K, Kersting M, Alexy U. Home-made and commercial complementary meals in German infants: results of the DONALD study. J Hum Nutr Diet. 2015;28(6):613–22.
52. Lockyer S. Commercial vs. home-made baby foods – how do they match up nutritionally? Nutr Bull. 2016;41(4):339–43.
53. Maslin K, Venter C. Nutritional aspects of commercially prepared infant foods in developed countries: a narrative review. Nutr Res Rev. 2017;30(1):138–48.
54. Smith JP, Sargent GM, Mehta K, James J, Berry N, Koh C et al. A rapid evidence assessment. Does marketing of commercially available complementary foods affect infant and young child feeding? Canberra: Australian National University; 2015 (http://www.who.int/nutrition/topics/CF_anu_effects_marketingcommercial.pdf).
55. Langley-Evans SC. Nutrition in early life and the programming of adult disease: a review. J Hum Nutr Diet 2015;28(Suppl. 1):1–14.
56. Horta BL, Victoria CG. Long-term effects of breastfeeding. A systematic review. Geneva: World Health Organization; 2013 (http://apps.who.int/iris/bitstream/handle/10665/79198/9789241505307_eng.pdf?sequence=1).
57. Lefebvre CM, John RM. The effect of breastfeeding on childhood overweight and obesity: a systematic review of the literature. J Am Assoc Nurse Pract. 2014;26(7):386–401.
58. Mameli C, Mazzantini S, Zuccotti GV. Nutrition in the first 1000 days: the origin of childhood obesity. Int J Environ Res Public Health 2016;13(9):23.
59. Laursen MF, Bahl MI, Michaelsen KF, Licht TR. First foods and gut microbes. Front Microbiol. 2017;8:356: doi:10.3389/fmicb.2017.00356.
60. Huh SY, Rifas-Shiman SL, Taveras EM, Oken E, Gillman ME. Timing of solid food introduction and risk of obesity in preschool-aged children. Pediatrics 2011;127(3):e544–51.
61. Moss BG, Yeaton WH. Early childhood healthy and obese weight status: potentially protective benefits of breastfeeding and delaying solid foods. Matern Child Health J. 2014;18(5):1224–32.
62. Michaelsen KF, Greer FR. Protein needs early in life and long-term health. Am J Clin Nutr. 2014;99(3):718S–22S.
63. Pimpin L, Jebb S, Johnson L, Wardle J, Ambrosini GL. Dietary protein intake is associated with body mass index and weight up to 5 y of age in a prospective cohort of twins. Am J Clin Nutr. 2016;103(2):389–97.
64. Socha P, Grote V, Gruszfeld D, Janas R, Demmelmair H, Closa-Monasterolo R et al. Milk protein intake, the metabolic-endocrine response, and growth in infancy: data from a randomized clinical trial. Am J Clin Nutr. 2011;94(6 Suppl.):1776s–84s.
65. Tang M, Krebs NF. High protein intake from meat as complementary food increases growth but not adiposity in breastfed infants: a randomized trial. Am J Clin Nutr. 2014;100(5):1322–8.

66. Colak H, Dulgergil CT, Dalli M, Hamidi MM. Early childhood caries update: a review of causes, diagnoses, and treatments. J Nat Sci Biol Med. 2013;4(1):29–38.
67. Welsh JA, Figueroa J. Intake of added sugars during the early toddler period. Nutr Today 2017;52(Suppl.):S60–8.
68. Newens KJ, Walton J. A review of sugar consumption from nationally representative dietary surveys across the world. J Hum Nutr Diet. 2016;29(2):225–40.
69. Karjalainen S, Soderling E, Sewon L, Lapinleimu H, Simell O. A prospective study on sucrose consumption, visible plaque and caries in children from 3 to 6 years of age. Community Dent Oral Epidemiol. 2001;29(2):136–42.
70. Karjalainen S, Tolvanen M, Pienihakkinen K, Söderling E, Lagström H, Simell O et al. High sucrose intake at 3 years of age is associated with increased salivary counts of mutans streptococci and lactobacilli, and with increased caries rate from 3 to 16 years of age. Caries Res. 2015;49(2):125–32.
71. Peres MA, Sheiham A, Liu P, Demarco FF, Silva AE, Assunção MC et al. Sugar consumption and changes in dental caries from childhood to adolescence. J Dent Res. 2016;95(4):388–94.
72. Guideline: sugars intake for adults and children. Geneva: World Health Organization; 2015 (http://www.who.int/nutrition/publications/guidelines/sugars_intake/en/).
73. Moynihan PJ, Kelly SA. Effect on caries of restricting sugars intake: systematic review to inform WHO guidelines. J Dent Res. 2014;93(1):8–18.
74. Moore DA, Goodwin TL, Brocklehurst PR, Armitage CJ, Glenny AM. When are caregivers more likely to offer sugary drinks and snacks to infants? A qualitative thematic synthesis. Qual Health Res 2017;27(1):74–88.
75. Walker RW, Goran MI. Laboratory Determined sugar content and composition of commercial infant formulas, baby foods and common grocery items targeted to children. Nutrients 2015;7(7):5850–67.
76. Codex standard for processed cereal-based foods for infants and young children. CODEX STAN 74–1981. Rome: Food and Agriculture Organization of the United Nations & World Health Organization; adopted 1981, revised 2006, amended 2017 (http://www.fao.org/fao-who-codexalimentarius/sh-proxy/en/?lnk=1&url=https%253A%252F%252Fworkspace.fao.org%252Fsites%252Fcodex%252FStandards%252FCODEX%2BSTAN%2B74-1981%252FCXS_074e.pdf).
77. Department of Health, the Food Standards Agency, and devolved administrations in Scotland, Northern Ireland and Wales in collaboration with the British Retail Consortium. Guide to creating a front of pack (FoP) nutrition label for pre-packed products sold through retail outlets. London: Department of Health; 2016 (https://assets.publishing.service.gov.uk/government/uploads/system/uploads/attachment_data/file/566251/FoP_Nutrition_labelling_UK_guidance.pdf).
78. Australian Competition and Consumer Commission v H.J. Heinz Company Australia Limited [2018] FCA 360. Federal Court of Australia, 2018 (http://www.judgments.fedcourt.gov.au/judgments/Judgments/fca/single/2018/2018fca0360).
79. Pearce J, Langley-Evans SC. The types of food introduced during complementary feeding and risk of childhood obesity: a systematic review. Int J Obes. 2013;37:477–85.
80. Hopkins D, Emmett P, Steer C, Rogers I, Noble S, Emond A. Infant feeding in the second 6 months of life related to iron status: an observational study. Arch Dis Child. 2007;92(10):850–4.
81. Gunther AL, Buyken AE, Kroke A. Protein intake during the period of complementary feeding and early childhood and the association with body mass index and percentage body fat at 7 y of age. Am J Clin Nutr. 2007;85(6):1626–33.
82. Scientific Advisory Committee on Nutrition 48th meeting, 30th June 2016, Skipton House, London. Final minutes. London: SACN; 2016 (https://app.box.com/s/qv74594fo7mwxgsqbrvc5fdbrycchma4/1/8429053429/77229943237/1).
83. Guidance on ending the inappropriate promotion of foods for infants and young children. Implementation manual. Geneva: World Health Organization; 2017 (http://apps.who.int/iris/bitstream/handle/10665/260137/9789241513470-eng.pdf;jsessionid=E2794EB5967BC17BAA1F78A8A66C2B04?sequence=1).

PART 3

Annexes

Annex 1

EUROPEAN COMMISSION DIRECTIVE 2006/125/EC ANNEX I[5]

Key points relating to Annex I
Article 1
1. This Directive is a 'specific Directive' within the meaning of Article 4(1) of Directive 89/398/EEC.

2. This Directive covers foodstuffs for particular nutritional use fulfilling the particular requirements of infants and young children in good health in the Community and are intended for use by infants while they are being weaned, and by young children as a supplement to their diet and/or for their progressive adaptation to ordinary food. They comprise:

(a) 'processed cereal-based foods' which are divided into the following four categories:
(i) simple cereals which are or have to be reconstituted with milk or other appropriate nutritious liquids;
(ii) cereals with an added high protein food which are or have to be reconstituted with water or other protein free liquid;
(iii) pastas which are to be used after cooking in boiling water or other appropriate liquids;
(iv) rusks and biscuits which are to be used either directly or, after pulverisation, with the addition of water, milk or other suitable liquids;
(v) b) 'baby foods' other than processed cereal-based foods.

ANNEX I
ESSENTIAL COMPOSITION OF PROCESSED CEREAL-BASED FOODS FOR INFANTS AND YOUNG CHILDREN
The requirements concerning nutrients refer to the products ready for use marketed as such or reconstituted as instructed by the manufacturer.

1. CEREAL CONTENT
Processed cereal-based foods are prepared primarily from one or more milled cereals and/or starchy root products. The amount of cereal and/or starchy root shall not be less than 25 % of the final mixture on a dry weight for weight basis.

2. PROTEIN
2.1. For products mentioned in Article 1(2)(a)(ii) and (iv), the protein content shall not exceed 1.3 g/100 kJ (5.5 g/100 kcal).
2.2. For products mentioned in Article 1(2)(a)(ii), the added protein shall not be less than 0.48 g/100 kJ (2 g/100 kcal).
2.3. For biscuits mentioned in Article 1(2)(a)(iv), made with the addition of a high protein food, and presented as such, the added protein shall not be less than 0.36 g/100 kJ (1.5 g/100 kcal).
2.4. The chemical index of the added protein shall be equal to at least 80 % of that of the reference protein (casein as defined in Annex III), or the protein efficiency ratio (PER) of the protein in the mixture shall be equal to at least 70 % of that of the reference protein. In all cases,

[5] *Source*: The Commission of the European Communities. Commission Directive 2006/125/EC on processed cereal-based foods and baby foods for infants and young children. O. J. E. U. 2006, L 339:16–35 (http://eur-lex.europa.eu/legal-content/EN/TXT/PDF/?uri=CELEX:32006L0125&from=EN).

the addition of amino acids shall be permitted solely for the purpose of improving the nutritional value of the protein mixture, and only in the proportions necessary for that purpose.

3. CARBOHYDRATES

3.1. If sucrose, fructose, glucose, glucose syrups or honey are added to products mentioned in Article 1(2)(a)(i) and (iv):

- the amount of added carbohydrates from these sources shall not exceed 1.8 g/100 kJ (7.5 g/100 kcal),
- the amount of added fructose shall not exceed 0.9 g/100 kJ (3.75 g/100 kcal).

3.2. If sucrose, fructose, glucose syrups or honey are added to products mentioned in Article 1(2)(a)(ii):

- the amount of added carbohydrates from these sources shall not exceed 1.2 g/100 kJ (5 g/100 kcal),
- the amount of added fructose shall not exceed 0.6 g/100 kJ (2.5 g/100 kcal).

4. LIPIDS

4.1. For products mentioned in Article 1(2)(a)(i) and (iv), the lipid content shall not exceed 0.8 g/100 kJ (3.3 g/100 kcal).

4.2. For products mentioned in Article 1(2)(a)(ii), the lipid content shall not exceed 1.1 g/100 kJ (4.5 g/100 kcal). If the lipid content exceeds 0.8 g/100 kJ (3.3 g/100 kcal):

- the amount of lauric acid shall not exceed 15% of the total lipid content,
- the amount of myristic acid shall not exceed 15% of the total lipid content,
- the amount of linoleic acid (in the form of glycerides = linoleates) shall not be less than 70 mg/100 kJ (300 mg/100 kcal) and shall not exceed 285 mg/100 kJ (1 200 mg/100 kcal).

5. MINERALS

5.1. Sodium

- sodium salts may only be added to processed cereal-based foods for technological purposes,
- the sodium content of processed cereal-based foods shall not exceed 25 mg/100 kJ (100 mg/100 kcal).

5.2. Calcium

5.2.1. For products mentioned in Article 1(2)(a)(ii), the amount of calcium shall not be less than 20 mg/100 kJ (80 mg/100 kcal).

5.2.2. For products mentioned in Article 1(2)(a)(iv), manufactured with the addition of milk (milk biscuits) and presented as such, the amount of calcium shall not be less than 12 mg/100 kJ (50 mg/100 kcal).

Annex 2

EUROPEAN COMMISSION DIRECTIVE 2006/125/EC ANNEX II [6]

Key points from Annex II
Compositional regulations
The requirements concerning nutrients refer to the products ready for use, marketed as such or reconstituted as instructed by the manufacturer.

1. Protein
1.1. If meat, poultry, fish, offal or other traditional source of protein are the only ingredients mentioned in the name of the product, then:

- the named meat, poultry, fish, offal or other traditional protein source, in total, shall constitute not less than 40% by weight of the total product,
- each named meat, poultry, fish, offal or other traditional source of protein shall constitute not less than 25%, by weight, of total named protein sources,
- the total protein from the named sources shall not be less than 1.7 g/100 kJ (7 g/100 kcal).

1.2. If meat, poultry, fish, offal or other traditional source of protein, singularly or in combination, are mentioned first in the name of the product, whether or not the product is presented as a meal, then:

- the named poultry, fish, offal or other traditional protein source, in total, shall constitute not less than 10% by weight of the total product,
- each named meat, poultry, fish, offal or other traditional source of protein shall constitute not less than 25% by weight, of total named protein sources,
- the protein from the named sources shall not be less than 1 g/100 kJ (4 g/100 kcal).

1.3. If meat, poultry, fish, offal or other traditional source of protein, singularly or in combination are mentioned, but not first, in the name of the product, whether or not the product is presented as a meal, then:

- the named meat, poultry, fish, offal or other traditional protein source, in total, shall constitute not less than 8% by weight of the total product,
- each named meat, poultry, fish, offal or other traditional source of protein shall constitute not less than 25%, by weight, of total named protein sources,
- the protein from the named sources shall not be less than 0.5 g/100 kJ (2.2 g/100 kcal),
- the total protein in the product from all sources shall not be less than 0.7 g/100 kJ (3 g/100 kcal).

1.4. If cheese is mentioned together with other ingredients in the name of a savoury product, whether or not the product is presented as a meal, then:

- the protein from the dairy sources shall not be less than 0.5 g/100 kJ (2.2 g/100 kcal),

[6] *Source*: The Commission of the European Communities. Commission Directive 2006/125/EC on processed cereal-based foods and baby foods for infants and young children. O. J. E. U. 2006, L 339:16–35 (http://eur-lex.europa.eu/legal-content/EN/TXT/PDF/?uri=CELEX:32006L0125&from=EN). as detailed in appendix of: Crawley H,

- the total protein in the product from all sources shall not be less than 0.7 g/100 kJ (3 g/100 kcal).

1.5. If the product is designated on the label as a meal, but does not mention meat, poultry, fish, offal or other traditional source of protein in the name of the product, the total protein in the product from all sources shall not be less than 0.7 g/100 kJ (3 g/100 kcal).

1.6. Sauces presented as an accompaniment to a meal shall be exempt from the requirements of points 1.1 to 1.5 inclusive.

1.7. Sweet dishes that mention dairy products as the first or only ingredient in the name shall contain not less than 2.2 g dairy protein/100 kcal. All other sweet dishes shall be exempt from the requirements in 1.1 to 1.5.

2. Carbohydrates
The quantities of total carbohydrates present in fruit and vegetable juices and nectars, fruit-only dishes, and desserts or puddings shall not exceed:

- 10 g/100 ml for vegetable juices and drinks based on them,
- 15 g/100 ml for fruit juices and nectars and drinks based on them,
- 20 g/100 g for fruit-only dishes,
- 25 g/100 g for desserts and puddings,
- 5 g/100 g for other non-milk-based drinks.

3. Fat
3.1. For products referred to in point 1.1:
If meat or cheese are the only ingredients or are mentioned first in the name of a product, the total fat in the product from all sources shall not exceed 1.4 g/100 kJ (6 g/100 kcal).

3.2. For all other products, the total fat in the product from all sources shall not exceed 1.1 g/100 kJ (4.5 g/100 kcal).

4. Sodium
4.1. The final sodium content in the product shall be either not more than 48 mg/100 kJ (200 mg/100 kcal) or not more than 200 mg per 100 g. However if cheese is the only ingredient mentioned in the name of the product, the final sodium content in the product shall not be more than 70 mg/100 kJ (300 mg/100 kcal).

4.2. Sodium salts may not be added to products based on fruit, nor to desserts or puddings except for technological purposes.

5. Vitamins
Vitamin A shall not be added to baby foods other than vegetable juices.

Vitamin D shall not be added to baby foods.

Annex 3

GUIDELINES FOR THE ENERGY REQUIREMENTS AND FAT FROM OF COMPLEMENTARY FOOD NEEDED FOR INFANTS[7]

A. The energy needs from complementary foods for infants with "average" breastmilk intake in developing countries are approximately 200 kcal per day at 6–8 months of age, 300 kcal per day at 9–11 months of age, and 550 kcal per day at 12–23 months of age. In industrialized countries these estimates differ somewhat (130, 310 and 580 kcal/d at 6-8, 9–11 and 12-23 months, respectively) because of differences in average breastmilk intake.

B. Scientific rationale: the total energy requirements of healthy, breastfed infants are approximately 615 kcal/d at 6–8 months, 686 kcal/d at 9–11 months, and 894 kcal/d at 12–23 months of age … . Energy needs from complementary foods are estimated by subtracting average breastmilk energy intake from total energy requirements at each age. Among breastfed children in developing countries, average breastmilk energy intake is 413, 379 and 346 kcal/d at 6–8, 9–11 and 12–23 months, respectively … . The equivalent values for industrialized countries (for breastfed children only) are 486, 375 and 313 kcal/d, respectively.

For the average healthy breastfed infant, meals of complementary foods should be provided 2–3 times per day at 6–8 months of age and 3–4 times per day at 9–11 and 12–24 months of age. Additional nutritious snacks (such as a piece of fruit or bread or chapatti with nut paste) may be offered 1–2 times per day, as desired. Snacks are defined as foods eaten between meals – usually self-fed, convenient and easy to prepare. If energy density or amount of food per meal is low, or the child is no longer breastfed, more frequent meals may be required. Nutrient-dense foods are recommended, and assuming a gastric capacity of 30 g/kg body weight/meal and a minimum energy density of complementary foods of 0.8 kcal/g.

Nutrient intake that needs to be supplied by complementary foods is 97% for iron, 86% for zinc, 81% for phosphorus, 76% for magnesium, 73% for sodium and 72% for calcium. Given the relatively small amounts of complementary foods that are consumed at 6–24 months the nutrient density (amount of each nutrient per 100 kcal of food) of complementary foods needs to be very high.

Unfortified complementary foods that are predominantly plant-based generally provide insufficient amounts of certain key nutrients (particularly iron, zinc and calcium) to meet the recommended nutrient intakes during the age range of 6-24 months. In industrialized countries, iron-fortified complementary foods have been widely consumed for decades, and some manufacturers have added zinc as a fortificant in recent years.

For fat the range of 30–45% of total energy has been suggested as a reasonable compromise between the risks of too little intake (such as inadequate essential fatty acids and low energy density) and excessive intake (thought to potentially increase the likelihood of childhood obesity and future cardiovascular disease, although the evidence on this point is limited …).

---

[7] *Source*: Pan American Health Organization, World Health Organization. Guiding principles for complementary feeding of the breastfed child. Washington (DC): Pan American Health Organization; 2003 (https://www.ennonline.net/compfeedingprinciples).

Annex 4

INFORMATION IN CODEX/STAN 074-1981 REVISED 2017 ON CEREAL BASED FOODS

2.1 Product definitions
Four categories are distinguished:
2.1.1 Products consisting of cereals which are or have to be prepared for consumption with milk or other appropriate nutritious liquids;
2.1.2 Cereals with an added high protein food which are or have to be prepared for consumption with water or other appropriate protein-free liquid;
2.1.3 Pasta which are to be used after cooking in boiling water or other appropriate liquids;
2.1.4 Rusks and biscuits which are to be used either directly or, after pulverization, with the addition of water, milk or other suitable liquids.
2.2 Other definitions
2.2.1 The term infant means a person not more than 12 months of age.
2.2.2 The term young children means persons from the age of more than 12 months up to the age of three years (36 months).

Energy density
3.2 The energy density of cereal-based foods should not be less than 3.3 kJ/g (0.8 kcal/g).
3.3.2 For products mentioned in points 2.1.2 and 2.1.4, the protein content shall not exceed 1.3 g/100 kJ (5.5 g/100 kcal).
3.3.3 For products mentioned in point 2.1.2 the added protein content shall not be less than 0.48 g/100 kJ (2 g/100 kcal).
For biscuits mentioned in point 2.1.4 made with the addition of a high protein food, and presented as such, the added protein shall not be less than 0.36 g/100 kJ (1.5 g/ 100 kcal).

Carbohydrates
3.4.1 if sucrose, fructose, glucose, glucose syrup or honey are added to products mentioned in points 2.1.1 and 2.1.4:

- the amount of added carbohydrates from these sources shall not exceed 1.8 g/100 kJ(7.5 g/100 kcal);
- the amount of added fructose shall not exceed 0.9 g/100 kJ (3.75 g/100 kcal).

3.4.2 If sucrose, fructose, glucose, glucose syrup or honey are added to products mentioned in point 2.1.2:

- the amount of added carbohydrates from these sources shall not exceed 1.2 g/100 kJ (5 g/100 kcal);
- the amount of added fructose shall not exceed 0.6 g/100 kJ (2.5 g/100 kcal).

Lipids
3.5.1 For products mentioned in point 2.1.2 the lipid content shall not exceed 1.1g/100 kJ (4.5 g/100 kcal). If the lipid content exceeds 0.8g/100kJ (3.3g/100kcal):

- the amount of linoleic acid (in the form of triglycerides=linoleates) shall not be less than 70 mg/100kJ (300 mg/100 kcal) and shall not exceed 285 mg/100 kJ (1200 mg/100 kcal);
- the amount of lauric acid shall not exceed 15% of the total lipid content;
- the amount of myristic acid shall not exceed 15% of the total lipid content.

3.5.2 Product categories 2.1.1 and 2.1.4 shall not exceed a maximum lipid content of 0.8 g /100 kJ (3.3 g/100kcal).

Minerals
3.6.1 The sodium content of the products described in Sections 2.1.1 to 2.1.4 of this Standard shall not exceed 24mg/100 kJ (100 mg/100 kcal) of the ready-to-eat product.
3.6.2 The calcium content shall not be less than 20 mg/100 kJ (80 mg/100 kcal) for products mentioned in points 2.1.2.
3.6.3 The calcium content shall not be less than 12 mg/100 kJ (50 mg/100 kcal) for products mentioned in point 2.1.4 manufactured with the addition of milk and presented as such.

Codex standard for canned baby foods CODEX STAN 73-1981 amended 2017

3.2 Consistency and particle size
3.2.1 Ready-to-eat baby foods are homogeneous or comminuted in the following forms:
(a) strained: food of a fairly uniform, small particle size which does not require and does not encourage chewing before being swallowed;
(b) junior: food that ordinarily contains particles of a size to encourage chewing by infants and children.

3.2.2 Dry baby foods, after reconstitution with water or other suitable liquid, approximate to the consistency and particle size of strained or junior foods under 3.2.1.

Annex 5

INFORMATION RELATING TO THE PROPOSED REGULATION (EU) NO. 609/2013[8]

Compositional requirements referred to in Article 2(2)

1. CEREAL CONTENT
Processed cereal-based food shall be prepared primarily from one or more milled cereals and/or starchy root products.

The amount of cereal and/or starchy root shall not be less than 25 % of the final mixture on a dry weight for weight basis.

2. PROTEIN
2.1. For cereals with an added high protein food which are or have to be reconstituted with water or other protein-free liquid and rusks and biscuits which are to be used either directly or, after pulverisation, with the addition of water, milk or other suitable liquids, the protein content shall not exceed 1,3 g/100 kJ (5,5 g/100 kcal).

2.2. For cereals with an added high protein food which are or have to be reconstituted with water or other protein-free liquid, the added protein shall not be less than 0,48 g/100 kJ (2 g/100 kcal).

2.3. For biscuits which are to be used either directly or, after pulverisation, with the addition of water, milk or other suitable liquids, made with the addition of a high protein food, and presented as such, the added protein shall not be less than 0,36 g/100 kJ (1,5 g/100 kcal).

2.4. The chemical index of the added protein shall be equal to at least 80 % of that of the reference protein (casein as set out in Table 1), or the protein efficiency ratio (PER) of the protein in the mixture shall be equal to at least 70 % of that of the reference protein. In all cases, the addition of amino acids shall be permitted solely for the purpose of improving the nutritional value of the protein mixture, and only in the proportions necessary for that purpose.

---

[8] *Sources*: Regulation (EU) No. 609/2013 of the European Parliament and of The Council of 12 June 2013 on food intended for infants and young children, food for special medical purposes, and total diet replacement for weight control and repealing Council Directive 92/52/EEC, Commission Directives 96/8/EC, 1999/21/EC, 2006/125/EC and 2006/141/EC, Directive 2009/39/EC of the European Parliament and of the Council and Commission Regulations (EC) No 41/2009 and (EC) No 953/2009. Strasbourg: European Parliament; 2013 (http://extwprlegs1.fao.org/docs/pdf/eur125448.pdf).

Annexes to the Commission delegated regulation (EU) …/... supplementing Regulation (EU) No 609/2013 of the European Parliament and of the Council as regards the specific compositional and information requirements for processed cereal-based food and baby food. Brussels: European Commission; 2015 (http://www.europarl.europa.eu/RegData/docs_autres_institutions/commission_europeenne/actes_delegues/2015/06507/COM_ADL(2015)06507(ANN)_EN.pdf).

Table 1. Amino acid composition of casein (g per 100 g of protein)

Arginine	3.7
Cystine	0.3
Histidine	2.9
Isoleucine	5.4
Leucine	9.5
Lysine	8.1
Methionine	2.8
Phenylalanine	5.2
Threonine	4.7
Tryptophan	1.6
Tyrosine	5.8
Valine	6.7

3. CARBOHYDRATES

3.1. If sucrose, fructose, glucose, glucose syrups or honey are added to simple cereals which are or have to be reconstituted with milk or other appropriate nutritious liquids and rusks and biscuits which are to be used either directly or, after pulverisation, with the addition of water, milk or other suitable liquids:

- the amount of added carbohydrates from these sources shall not exceed 1,8 g/100 kJ (7,5 g/100 kcal),
- the amount of added fructose shall not exceed 0,9 g/100 kJ (3,75 g/100 kcal).

3.2. If sucrose, fructose, glucose syrups or honey are added to cereals with an added high protein food which are or have to be reconstituted with water or other protein-free liquid:

- the amount of added carbohydrates from these sources shall not exceed 1,2 g/100 kJ (5 g/100 kcal),
- the amount of added fructose shall not exceed 0,6 g/100 kJ (2,5 g/100 kcal).

4. LIPIDS

4.1. For simple cereals which are or have to be reconstituted with milk or other appropriate nutritious liquids and rusks and biscuits which are to be used either directly or, after pulverisation, with the addition of water, milk or other suitable liquids, the lipid content shall not exceed 0,8 g/100 kJ (3,3 g/100 kcal).

4.2. For cereals with an added high protein food which are or have to be reconstituted with water or other protein-free liquid, the lipid content shall not exceed 1,1 g/100 kJ (4,5 g/100 kcal). If the lipid content exceeds 0,8 g/100 kJ (3,3 g/100 kcal):

- the amount of lauric acid shall not exceed 15 % of the total lipid content,
- the amount of myristic acid shall not exceed 15 % of the total lipid content,
- the amount of linoleic acid (in the form of glycerides = linoleates) shall not be less than 70 mg/100 kJ (300 mg/100 kcal) and shall not exceed 285 mg/100 kJ (1 200 mg/100 kcal).

5. MINERALS

5.1. Sodium

5.1.1. Sodium salts may only be added to processed cereal-based food for technological purposes.

5.1.2. The sodium content of processed cereal-based food shall not exceed 25 mg/100 kJ (100 mg/100 kcal).

5.2. Calcium

5.2.1. For cereals with an added high protein food which are or have to be reconstituted with water or other protein-free liquid, the amount of calcium shall not be less than 20 mg/100 kJ (80 mg/100 kcal).

5.2.2. For rusks and biscuits which are to be used either directly or, after pulverisation, with the addition of water, milk or other suitable liquids, manufactured with the addition of milk (milk biscuits) and presented as such, the amount of calcium shall not be less than 12 mg/100 kJ (50 mg/100 kcal).

6. VITAMINS

6.1. For processed cereal-based food, the amount of thiamin shall not be less than 25 µg/100 kJ (100 µg/100 kcal).

6.2. For cereals with an added high protein food which are or have to be reconstituted with water or other protein-free liquid, the limits set out in Table 2 shall apply. Those limits shall also apply where vitamins A and D are added to other processed cereal-based foods.

Table 2. Vitamins

	Per 100 kJ		Per 100 kcal	
**Vitamin**	**Minimum**	**Maximum**	**Minimum**	**Maximum**
Vitamin A (µg-RE)	16.7	27.2	70	114
Vitamin D (µg)	0.48	0.72	2	3
Thiamine (µg)	9.6	72	40	300
Riboflavin (µg)	14.3	95.6	60	400
Pantothenic acid (mg)	0.1	0.48	0.4	2
Vitamin B_6 (µg)	4.8	41.8	20	175
Biotin (µg)	0.24	1.8	1	7.5
Folate (µg-DFE)	3.6	11.4	15	47.6
Vitamin B_{12} (µg)	0.02	0.12	0.1	0.5
Vitamin C (mg)	0.96	7.2	4	30
Vitamin K (µg)	0.24	6	1	25
Vitamin E (mg α-tocopherol)	0.14	1.2	0.6	5

7. MAXIMUM LIMITS FOR VITAMINS, MINERALS AND TRACE ELEMENTS, IF ADDED

The maximum limits set out in Table 3 shall apply. By way of derogation from Article 2(4), the limits for potassium and calcium refer to the product as sold.

Table 3. Nutrient maximum per 100 kcal

Vitamin A (µg RE)	180
Vitamin E (mg α-TE)[a]	3
Vitamin D (µg)	3
Vitamin C (mg)	12.5/25[b]

Thiamin (mg)	0.5
Riboflavin (mg)	0.4
Niacin (mg NE)[c]	4.5
Vitamin B6 (mg)	0.35
Folic acid (µg)	50
Vitamin B12 (µg)	0.35
Pantothenic acid (mg)	1.5
Biotin (µg)	10
Potassium (mg)	160
Calcium (mg)	80/180[d] /100[e]
Magnesium (mg)	40
Iron (mg)	3
Zinc (mg)	2
Copper (µg)	40
Iodine (µg)	35
Manganese (mg)	0.6

[a] α-TE = d-α-tocopherol equivalent.
[b] Limit applicable to products fortified with iron.
[c] NE = Niacin equivalent = mg nicotinic acid + mg tryptophan/60.
[d] Limit applicable to simple cereals which are or have to be reconstituted with milk or other appropriate nutritious liquids, and cereals with an added high protein food which are or have to be reconstituted with water or other protein-free liquid.
[e] Limit applicable to rusks and biscuits which are to be used either directly or, after pulverisation, with the addition of water, milk or other suitable liquids.

Compositional requirements referred to in Article 2(3)

1. PROTEIN

1.1. If meat, poultry, fish, offal or other traditional source of protein are the only ingredients mentioned in the name of the product:

- the named meat, poultry, fish, offal or other traditional protein source, in total, shall constitute not less than 40 % by weight of the total product,
- each named meat, poultry, fish, offal or other traditional source of protein shall constitute not less than 25 %, by weight, of total named protein sources,
- the total protein from the named sources shall not be less than 1,7 g/100 kJ (7 g/100 kcal).

1.2. If meat, poultry, fish, offal or other traditional source of protein, singularly or in combination, are mentioned first in the name of the product, whether or not the product is presented as a meal:

- the named meat, poultry, fish, offal or other traditional protein source, in total, shall constitute not less than 10 % by weight of the total product,
- each named meat, poultry, fish, offal or other traditional source of protein shall constitute not less than 25 % by weight, of total named protein sources,
- the protein from the named sources shall not be less than 1 g/100 kJ (4 g/100 kcal).

1.3. If meat, poultry, fish, offal or other traditional source of protein, singularly or in combination are mentioned, but not first, in the name of the product, whether or not the product is presented as a meal:

- the named meat, poultry, fish, offal or other traditional protein source, in total, shall constitute not less than 8 % by weight of the total product,
- each named meat, poultry, fish, offal or other traditional source of protein shall constitute not less than 25 %, by weight, of total named protein sources,
- the protein from the named sources shall not be less than 0,5 g/100 kJ (2,2 g/100 kcal),
- the total protein in the product from all sources shall not be less than 0,7 g/100 kJ (3 g/100 kcal).

1.4. If cheese is mentioned together with other ingredients in the name of a savoury product, whether or not the product is presented as a meal:

- the protein from the dairy sources shall not be less than 0,5 g/100 kJ (2,2 g/100 kcal),
- the total protein in the product from all sources shall not be less than 0,7 g/100 kJ (3 g/100 kcal).

1.5. If the product is designated on the label as a meal, but does not mention meat, poultry, fish, offal or other traditional source of protein in the name of the product, the total protein in the product from all sources shall not be less than 0,7 g/100 kJ (3 g/100 kcal).

1.6. Sauces presented as an accompaniment to a meal shall be exempt from the requirements of points 1.1 to 1.5.

1.7. Sweet dishes that mention dairy products as the first or only ingredient in the name shall contain not less than 2,2 g dairy protein/100 kcal. All other sweet dishes shall be exempt from the requirements in 1.1 to 1.5.

1.8. The addition of amino acids shall be permitted solely for the purpose of improving the nutritional value of the protein present, and only in the proportions necessary for that purpose.

2. CARBOHYDRATES

The quantities of total carbohydrates present in fruit and vegetable juices and nectars, fruit-only dishes, and desserts or puddings shall not exceed:

- 10 g/100 ml for vegetable juices and drinks based on them,
- 15 g/100 ml for fruit juices and nectars and drinks based on them,
- 20 g/100 g for fruit-only dishes,
- 25 g/100 g for desserts and puddings,
- 5 g/100 g for other non-milk-based drinks.

3. FAT

3.1. For products referred to in point 1.1:
If meat or cheese are the only ingredients or are mentioned first in the name of a product, the total fat in the product from all sources shall not exceed 1,4 g/100 kJ (6 g/100 kcal).

3.2. For all other products, the total fat in the product from all sources shall not exceed 1,1 g/100 kJ (4,5 g/100 kcal).

4. SODIUM

4.1. The final sodium content in the product shall be either not more than 48 mg/100 kJ (200 mg/100 kcal) or not more than 200 mg per 100 g. However if cheese is the only ingredient

mentioned in the name of the product, the final sodium content in the product shall not be more than 70 mg/100 kJ (300 mg/100 kcal).
4.2. Sodium salts may not be added to products based on fruit, nor to desserts, puddings except for technological purposes.

5. VITAMINS
Vitamin C
In a fruit juice, nectar, or vegetable juice the final content of vitamin C in the product shall be either not less than 6 mg/100 kJ (25 mg/100 kcal) or not less than 25 mg per 100 g.

Vitamin A
In vegetable juices, the final content of vitamin A in the product shall be not less than 25 µg RE/100 kJ (100 µg RE/100 kcal). Vitamin A shall not be added to other baby food.

Annex 6

OBJECTION FROM EUROPEAN PARLIAMENT[9]

A. whereas the Commission has failed to present to Parliament and the Council the report on young-child formula required by Regulation (EU) No 609/2013 (Article 12), which is a necessary condition for national strategies to reduce childhood obesity;

B. whereas part 3 of Annex I to the delegated regulation allows 30 % of the energy in baby foods to be provided by sugar (7,5 g sugar/100 kcal is equivalent to 30 kcal from sugar in 100 kcal energy);

C. whereas the provisions contained in part 3 of Annex I are contrary to all health advice from the World Health Organisation (WHO)2 – which recommends limiting the intake of free sugars to less than 10 % of total energy intake, with a further reduction to less than 5 % of total energy intake for additional health benefits – and from scientific committees in Member States, which have recommended significant reductions in total sugar intake; whereas the introduction of such foods – especially at such an early stage – is likely to contribute to rising levels of childhood obesity and may affect the developing taste preferences of children; whereas, in the case of infants and young children in particular, added sugar levels should be kept to a minimum;

[9] *Source*: P8 TA(2016)0015. Objection to a delegated act: specific compositional and information requirements for processed cereal-based food and baby food. Strasbourg: European Parliament; 2016 (http://www.europarl.europa.eu/sides/getDoc.do?pubRef=-//EP//NONSGML+TA+P8-TA-2016-0015+0+DOC+PDF+V0//EN).

Annex 7

MATERNAL, INFANT AND YOUNG CHILD NUTRITION. GUIDANCE ON ENDING THE INAPPROPRIATE PROMOTION OF FOODS FOR INFANTS AND YOUNG CHILDREN[10]

PURPOSE

1. The purpose of this document is to provide guidance on ending the inappropriate promotion of foods for infants and young children, with the aim to promote, protect and support breastfeeding, prevent obesity and noncommunicable diseases, promote healthy diets, and ensure that caregivers receive clear and accurate information on feeding.

SCOPE

2. The term "foods" is used in this guidance to refer to both foods and beverages (including complementary foods). Guidance on the inappropriate promotion of breast-milk substitutes is contained in the Code of Marketing of Breast-milk Substitutes and subsequent relevant Health Assembly resolutions. The current document does not replace any provisions in the Code but clarifies the inclusion of certain products that should be covered by the Code and subsequent resolutions.

3. This guidance applies to all commercially produced foods that are marketed as being suitable for infants and young children from the age of 6 months to 36 months. Products are considered to be marketed as being suitable for this age group if they (a) are labelled with the words "baby", "infant," "toddler" or "young child"; (b) are recommended for introduction at an age of less than 3 years; (c) have a label with an image of a child who appears to be younger than 3 years of age or feeding with a bottle; or (d) are in any other way presented as being suitable for children under the age of 3 years. This approach is in line with the relevant Codex guidelines and standards on foods for infants and young children that refer to young children up to the age of 3 years *(1)*.

4. This guidance is not applicable to vitamin and mineral food supplements and home-fortification products such as micronutrient powders and small-quantity lipid-based nutrient supplements. Although such supplements and products are often classified as foods for regulatory purposes, they are not foods per se, but fortification products. Many of the principles contained in this guidance, including those concerning adherence to national and global standards for nutrient levels, safety and quality and to prohibitions on any messages indicating their use for infants under 6 months of age, should nevertheless be applied to such products.

5. The promotion of foods for infants and young children occurs through government programmes, non-profit organizations and private enterprises. This guidance is applicable in all these settings, as the principles it contains are important regardless of who is responsible for the promotion.

---

[10] *Source*: Maternal, infant and young child nutrition. Guidance on ending the inappropriate promotion of foods for infants and young children. In: Sixty-ninth World Health Assembly: documentation [website]. Geneva: World Health Organization; 2016 (Document A69/7 Add.1; http://apps.who.int/gb/e/e_wha69.html).

DEFINITIONS

6. Foods for infants and young children are defined as commercially produced food or beverage products that are specifically marketed as suitable for feeding children up to 36 months of age.

7. Marketing means product promotion, distribution, selling, advertising, product public relations and information services.

8. Promotion is broadly interpreted to include the communication of messages that are designed to persuade or encourage the purchase or consumption of a product or raise awareness of a brand. Promotional messages may be communicated through traditional mass communication channels, the Internet and other marketing media using a variety of promotional methods. In addition to promotional techniques aimed directly at consumers, measures to promote products to health workers or to consumers through other intermediaries are included. There does not have to be a reference to a brand name of a product for the activity to be considered as advertising or promotion.

9. Cross-promotion (also called brand crossover promotion or brand stretching) is a form of marketing promotion where customers of one product or service are targeted with promotion of a related product. This can include packaging, branding and labelling of a product to closely resemble that of another (brand extension). In this context, it can also refer to use of particular promotional activities for one product and/or promotion of that product in particular settings to promote another product.

RECOMMENDATIONS

10. **Recommendation 1**. Optimal infant and young child feeding should be promoted based on the guiding principles for complementary feeding of the breastfed child *(2)* and the guiding principles for feeding non-breastfed children 6–24 months of age *(3)*. Emphasis should be placed on the use of suitable, nutrient-rich, home-prepared, and locally available foods that are prepared and fed safely *(4)*.

11. **Recommendation 2**. Products that function as breast-milk substitutes should not be promoted. A breast-milk substitute should be understood to include any milks (or products that could be used to replace milk, such as fortified soy milk), in either liquid or powdered form, that are specifically marketed for feeding infants and young children up to the age of 3 years (including follow-up formula and growing-up milks). It should be clear that the implementation of the International Code of Marketing of Breast-milk Substitutes and subsequent relevant Health Assembly resolutions covers all these products.

12. **Recommendation 3**. Foods for infants and young children that are not products that function as breast-milk substitutes should be promoted only if they meet all the relevant national, regional and global standards for composition, safety, quality and nutrient levels and are in line with national dietary guidelines. Nutrient profile models should be developed and utilized to guide decisions on which foods are inappropriate for promotion. Relevant Codex standards and guidelines *(5)* should be updated and additional guidelines developed in line with WHO’s guidance to ensure that products are appropriate for infants and young children, with a particular focus on avoiding the addition of free sugars and salt.

13. **Recommendation 4**. The messages used to promote foods for infants and young children should support optimal feeding and inappropriate messages should not be included. Messages about commercial products are conveyed in multiple forms, through advertisements, promotion

and sponsorship, including brochures, online information and package labels. Irrespective of the form, messages should always:

- include a statement on the importance of continued breastfeeding for up to two years or beyond and the importance of not introducing complementary feeding before 6 months of age;
- include the appropriate age of introduction of the food (this must not be less than 6 months);
- be easily understood by parents and other caregivers, with all required label information being visible and legible.

14. Messages should not:

- include any image, text or other representation that might suggest use for infants under the age of 6 months (including references to milestones and stages);
- include any image, text or other representation that is likely to undermine or discourage breastfeeding, that makes a comparison to breast-milk, or that suggests that the product is nearly equivalent or superior to breast-milk;
- recommend or promote bottle feeding;
- convey an endorsement or anything that may be construed as an endorsement by a professional or other body, unless this has been specifically approved by relevant national, regional or international regulatory authorities.

15. **Recommendation 5**. There should be no cross-promotion to promote breast-milk substitutes indirectly via the promotion of foods for infants and young children.

- The packaging design, labelling and materials used for the promotion of complementary foods must be different from those used for breast-milk substitutes so that they cannot be used in a way that also promotes breast-milk substitutes (for example, different colour schemes, designs, names, slogans and mascots other than company name and logo should be used).
- Companies that market breast-milk substitutes should refrain from engaging in the direct or indirect promotion of their other food products for infants and young children by establishing relationships with parents and other caregivers (for example through baby clubs, social media groups, childcare classes and contests).

16. **Recommendation 6**. Companies that market foods for infants and young children should not create conflicts of interest in health facilities or throughout health systems. Health workers, health systems, health professional associations and nongovernmental organizations should likewise avoid such conflicts of interest. Such companies, or their representatives, should not:

- provide free products, samples or reduced-price foods for infants or young children to families through health workers or health facilities, except:
    - as supplies distributed through officially sanctioned health programmes. Products distributed in such programmes should not display company brands;
- donate or distribute equipment or services to health facilities;
- give gifts or incentives to health care staff;
- use health facilities to host events, contests or campaigns;
- give any gifts or coupons to parents, caregivers and families;
- directly or indirectly provide education to parents and other caregivers on infant and young child feeding in health facilities;
- provide any information for health workers other than that which is scientific and factual;
- sponsor meetings of health professionals and scientific meetings.

17. Likewise, health workers, health systems, health professional associations and nongovernmental organizations should not:

- accept free products, samples or reduced-price foods for infants or young children from companies, except:
    - as supplies distributed through officially sanctioned health programmes. Products distributed in such programmes should not display company brands;
- accept equipment or services from companies that market foods for infants and young children;
- accept gifts or incentives from such companies;
- allow health facilities to be used for commercial events, contests or campaigns;
- allow companies that market foods for infants and young children to distribute any gifts or coupons to parents, caregivers and families through health facilities;
- allow such companies to directly or indirectly provide education in health facilities to parents and other caregivers;
- allow such companies to sponsor meetings of health professionals and scientific meetings.

18. **Recommendation 7**. The WHO set of recommendations on the marketing of foods and non-alcoholic beverages to children *(6)* should be fully implemented, with particular attention being given to ensuring that settings where infants and young children gather are free from all forms of marketing of foods high in saturated fats,[11] trans-fats, free sugars or salt. While foods marketed to children may not be specifically intended for infants and young children, they may, nevertheless, be consumed by them. A range of strategies should be implemented to limit the consumption by infants and young children of foods that are unsuitable for them.

References

1. Codex guidelines on formulated complementary foods for older infants and young children (CAC/GL-8-1991, revised in 2013); Codex standard for processed cereal-based foods for infants and young children (Codex/STAN 074-1981, revised in 2006); Codex standard for canned baby foods (CODEX STAN 73-1981); and Codex standard for follow-up formula (CODEX STAN 156-1987).
2. PAHO and WHO. Guiding principles for complementary feeding of the breastfed child. 2003. http://www.who.int/maternal_child_adolescent/documents/a85622/en/.
3. WHO. Guiding principles for feeding non-breastfed children 6–24 months of age. 2005 http://www.who.int/maternal_child_adolescent/documents/9241593431/en/.
4. See WHO/UNICEF. Global strategy for infant and young child feeding, Geneva. 2003. http://apps.who.int/iris/bitstream/10665/42590/1/9241562218.pdf?ua=1&ua=1.
5. Codex Guidelines on formulated complementary foods for older infants and young children (CAC/GL-8-1991, revised in 2013); Codex standard for processed cereal-based foods for infants and young children (Codex/STAN 074-1981, revised in 2006); Codex standard for canned baby foods (Codex/STAN 73-1981, revised in 1989); Codex advisory list of vitamin components for use in foods for infants and children (CAC/GL 10-1979, revised in 2009).
6. WHO. Set of recommendations on the marketing of foods and non-alcoholic beverages to children. Geneva: World Health Organization; 2010.

[11] While diets for young children should have adequate fat content, a 2008 joint FAO/WHO expert consultation proposed that no more than 35% of total energy should come from fat.

Annex 8

WHO VITAMIN AND MINERAL REQUIREMENTS IN HUMAN NUTRITION 2004[12]

Annex 1

Recommended nutrient intakes[a] — minerals

Group	Calcium[b] (mg/day)	Selenium (µg/day)	Magnesium (mg/day)	Zinc[c] (mg/day) High bioavailability	Zinc[c] (mg/day) Moderate bioavailability	Zinc[c] (mg/day) Low bioavailability
**Infants**						
0–6 months	300[d]<br>400[g]	6	26[d]<br>36[h]	1.1[d]	2.8	6.6
7–12 months	400	10	54	0.8[d]<br>2.5[j]	4.1	8.4
**Children**						
1–3 years	500	17	60	2.4	4.1	8.3
4–6 years	600	22	76	2.9	4.8	9.6
7–9 years	700	21	100	3.3	5.6	11.2

[a] Recommended nutrient intake (RNI) is the daily intake which meets the nutrient requirements of almost all (97.5%) apparently healthy individuals in an age- and sex-specific population.
[b] See Chapter 4 for details.
[c] See Chapter 12 for details.
[d] Breastfed.
[e] Neonatal iron stores are sufficient to meet the iron requirement for the first 6 months in full-term infants. Premature infants and low birth weight infants require additional iron.
[f] Recommendation for the age group 0–4.9 years.
[g] Cow milk-fed.
[h] Formula-fed.

Group	Iron (mg/day) 15% Bioavailability	Iron (mg/day) 12% Bioavailability	Iron (mg/day) 10% Bioavailability	Iron (mg/day) 5% Bioavailability	Iodine (µg/day)
**Infants**					
0–6 months	[e]	[e]	[e]	[e]	90[f]
7–12 months	6.2[i]	7.7[i]	9.3[i]	18.6[i]	90[f]
**Children**					
1–3 years	3.9	4.8	5.8	11.6	90[f]
4–6 years	4.2	5.3	6.3	12.6	90[f]
7–9 years	5.9	7.4	8.9	17.8	120 (6–12 yrs)

[12] *Source*: World Health Organization, Food and Agricultural Organization of the United Nations. Vitamin and mineral requirements in human nutrition, 2nd edition. Geneva: World Health Organization; 2004 (http://www.who.int/nutrition/publications/micronutrients/9241546123/en/).

[i] Bioavailability of dietary iron during this period varies greatly.
[j] Not applicable to infants exclusively breastfed.
[k] Particularly during the growth spurt.
[l] Pre-menarche.
[m] Not specified.
[n] It is recommended that iron supplements in tablet form be given to all pregnant women because of the difficulties in correctly assessing iron status in pregnancy. In non-anaemic pregnant women, daily supplements of 100 mg of iron (e.g. as ferrous sulphate) given during the second half of pregnancy are adequate. In anaemic women higher doses are usually required.

Annex 2

Recommended nutrient intakes[a] — water- and fat-soluble vitamins

	Water-soluble vitamins					
Group	Vitamin C[b] (mg/day)	Thiamine (mg/day)	Riboflavin (mg/day)	Niacin[c] (mg NE/day)	Vitamin B_6 (mg/day)	Pantothenate (mg/day)
**Infants**						
0–6 months	25	0.2	0.3	2[i]	0.1	1.7
7–12 months	30	0.3	0.4	4	0.3	1.8
**Children**						
1–3 years	30	0.5	0.5	6	0.5	2.0
4–6 years	30	0.6	0.6	8	0.6	3.0
7–9 years	35	0.9	0.9	12	1.0	4.0

[a] Recommended nutrient intake (RNI) is the daily intake which meets the nutrient requirements of almost all (97.5%) apparently healthy individuals in an age- and sex-specific population.
[b] See Chapter 7 for details.
[c] NE = Niacin equivalents.
[d] DFE = Dietary folate equivalents; μg of DFE provided = [μg of food folate + (1.7 × μg of synthetic folic acid)].
[e] Vitamin A values are "recommended safe intakes" instead of RNIs. See Chapter 2 for further details.
[f] Recommended safe intakes as μg retinol equivalent (RE)/day; conversion factors are as follows:
1 μg retinol = 1 RE
1 μg β-carotene = 0.167 μg RE
1 μg other provitamin A carotenoids = 0.084 μg RE.

Water-soluble vitamins			Fat-soluble vitamins			
Biotin (μg/day)	Vitamin B_{12} (μg/day)	Folate[d] (μg DFE/day)	Vitamin A[e,f] (μg RE/day)	Vitamin D (μg/day)	Vitamin E[g] (mg α-TE/day)	Vitamin K[h] (μg/day)
5	0.4	80	375	5	2.7[j]	5[k]
6	0.7	80	400	5	2.7[j]	10
8	0.9	150	400	5	5.0[j]	15
12	1.2	200	450	5	5.0[j]	20
20	1.8	300	500	5	7.0[j]	25

[g] Data were not strong enough to formulate recommendations. The figures in the table therefore represent the best estimate of requirements.
[h] See Chapter 6 for details.
[i] Preformed niacin.
[j] See Chapter 5 for details.
[k] This intake cannot be met by infants who are exclusively breastfed. To prevent bleeding due to vitamin K deficiency, all breast-fed infants should receive vitamin K supplementation at birth according to nationally approved guidelines.
[l] Not specified.

Annex 9

NUTRIENT PROFILE MODEL FOR CHILDREN OVER 36 MONTHS[13]

	Food category	Included in category (examples)	Not included in category (examples)	Customs tariff code (position and/or subposition number)[a]	Marketing not permitted if product exceeds, per 100 g[b]						
					total fat (g)	sat. fat (g)	total sugars (g)	added sugars (g)	non-sugar sweeteners (g)	salt (g)	energy (kcal)
1	Chocolate and sugar confectionery, energy bars, and sweet toppings and desserts	Chocolate and other products containing cocoa; white chocolate; jelly, sweets and boiled sweets; chewing gum and bubble gum; caramels; liquorice sweets; spreadable chocolate and other sweet sandwich toppings; nut spreads, including peanut butter; cereal, granola and muesli bars; marzipan	Chocolate flavoured breakfast cereals; cakes and pastries; biscuits and other baked goods covered in chocolate	17.04; 18.06; some of 19.05; 20.06; some of 20.08; some of 21.06	Not permitted						
2	Cakes, sweet biscuits and pastries; other sweet bakery wares, and dry mixes for making such	Pastries; croissants; cookies/ biscuits; sponge cakes; wafers; fruit pies; sweet buns; chocolate-covered biscuits; cake mixes and batters	Bread and bread products	19.01.20; 19.05.20; 19.05.31; 19.05.32	Not permitted						
3	Savoury snacks	Popcorn and maize corn; seeds; nuts and mixed nuts; savoury biscuits and pretzels; other snacks made from rice, maize, dough or potato		08.01; 08.02; 10.05; 19.04.10, 19.04.20; some of 19.05; 20.05.20; 20.08.11; 20.08.19; 20.08.99				0		0.1[c]	
4	Beverages										
	a) Juices	100% fruit and vegetable juices; juices reconstituted from concentrate, and smoothies		20.09	Not permitted[d]						
	b) Milk drinks[e]	Milks and sweetened milks; almond, soya, rice and oat milks	Cream	Some of 04.01; some of 04.02; 22.02.90	2.5			0	0		
	c) Energy drinks[f]			Some of 22.02	Not permitted						
	d) Other beverages	Cola, lemonade, orangeade; other soft drinks, mineral and/or flavoured waters (including aerated) with added sugars or sweetener	100% fruit and vegetable juices; milk drinks	22.01; some of 22.02				0	0		
5	Edible ices	Ice cream, frozen yoghurt, iced lollies and sorbets		21.05	Not permitted						
6	Breakfast cereals[g]	Oatmeal; cornflakes; chocolate breakfast cereals; mueslis		19.04.10; 19.04.20	10		15			1.6	
7	Yoghurts, sour milk, cream and other similar foods	Yoghurt; kephir; buttermilk; flavoured sour, fermented milk and drinking yoghurt; fromage frais; cheese-based and other yoghurt substitutes; yoghurt products containing additional ingredients (such as fruit, muesli); cream	Milks and sweetened milks; almond, rice and oat milks	Some of 04.02; 04.03; 04.04; some of 04.06.10; 19.01.10; 19.01.90; some of 21.06	2.5	2.0	10			0.2[c]	
8	Cheese	Medium-hard and hard cheeses; soft cheeses; fresh cheese (such as ricotta, mozzarella); grated or powdered cheese; cottage cheese; processed cheese spreads		04.06	20					1.3	
9	Ready-made and convenience foods and composite dishes	Pizzas; lasagne and other pasta dishes with sauces; quiches; ready meals; ready-made sandwiches; filled pastas; soups and stews (packaged or tinned); mixes and dough		Some of 16; some of 19.01.20; 19.02.19; 19.02.20; some of 19.05; some of 20.05; 21.04	10	4	10			1	225
10	Butter and other fats and oils	Butter; vegetable oils, margarines and spreads		04.05; 15		20				1.3	
11	Bread, bread products and crisp breads[g]	Ordinary bread (containing cereal, leavens and salt); gluten-free bread; unleavened bread; crisp breads; rusks and toasted breads	Sweet biscuits; pastries; cakes	19.05.10; 19.05.40; 19.05.90	10		10			1.2	
12	Fresh or dried pasta, rice and grains		Filled pasta and pasta in sauce	10; some of 11; 19.02 excluding 19.02.20	10		10			1.2	
13	Fresh and frozen meat, poultry, fish and similar	Eggs		02 excluding 02.10; some of 03 excluding 03.05	Permitted						
14	Processed meat, poultry, fish and similar	Sausage, ham, bacon; chicken nuggets; smoked and pickled fish; tinned fish in brine or oils; fish fingers and breaded/battered fish	Pepperoni pizza	02.10; some of 03; some of 16	20					1.7	
15	Fresh and frozen fruit, vegetables and legumes	Fruit and vegetables; legumes; starchy vegetables, roots and tubers	Tinned fruits, vegetables and legumes; fruit in syrup; dried fruit; frozen fruit with added sugar	07 excluding 07.10, 07.11, 07.12, 07.13; some of 08 excluding 08.01; 08.02; 08.11; 08.12; 08.13; 08.14	Permitted						
16	Processed fruit, vegetables and legumes	Tinned fruit, vegetables and legumes; dried fruit,[h] dried vegetables and legumes; marmalade; jams; pickled vegetables and fruit; stewed fruits; fruit peel; frozen French fries; frozen fruit with added sugar	Fruit juice	07.10; 07.11; 07.12; 07.13; some of 08.03; some of 08.05; some of 08.06; 08.11, 08.12, 08.13 and 08.14; 20.01; 20.02; 20.03; 20.04; 20.05; 20.06; 20.07; 20.08.20, 20.08.30, 20.08.40, 20.08.50, 20.08.60, 20.08.70, 20.08.80; 20.08.93; 20.08.97; 20.08.99	5		10	0		1	
17	Sauces, dips and dressings	Salad dressings; tomato ketchup; mayonnaise; ready-to-use dips; soya sauce; mustard and mustard flour		21.03	10			0		1	

Sat. fat = saturated fat.

[a] Where appropriate, a four-digit position number has been given. Where "some of" is indicated, this means that most (but not all) food products in this position number are covered. In some instances a six-digit subposition is provided so as to pinpoint specific products more easily.

[b] The food products should, where possible, be assessed as sold or as reconstituted (if necessary) according to the manufacturer's instructions.

[c] Salt equivalent.

[d] This is in line with the WHO Guidelines on Sugars Intake for Children and Adults (in press), as fruit juices are a significant source of free sugars for children. However, it is recognized that countries, according to national context and national food-based dietary guidelines, may take the decision to permit the marketing of 100% fruit juices in small portions.

[e] This nutrient profile model applies to products for children above 36 months. Follow-up formulas and growing-up milks are not covered by this model. It should be noted that World Health Assembly Resolution WHA39.28, adopted in 1986, states that the practice of providing infants with specially formulated milks (so called "follow-up milks") is not necessary. Further, any food or drink given before complementary feeding is nutritionally required may interfere with the initiation or maintenance of breastfeeding and should, therefore, be neither promoted nor encouraged for use by infants during this period.

[f] There is no agreement on a definition of energy drinks. However, such a category of drinks includes a variety of non-alcoholic beverages. While caffeine is considered the main ingredient, a number of other substances are often present. The most common of these include guarana, taurine, glucuronolactone and vitamins. A common feature is that these beverages are marketed for their actual or perceived effects as stimulants, energizers and performance enhancers.

[g] For this category, countries may choose to include a threshold for minimum dietary fibre content, for example >6g dietary fibre.

[h] This is in line with the WHO Guidelines on Sugars Intake for Children and Adults (in press), as dried fruits are a significant source of concentrated sugars for children. However, it is recognized that countries, according to national context and national food-based dietary guidelines, may take the decision to permit the marketing of dried fruits in small portions.

13 *Source*: WHO Regional Office for Europe nutrient profile model. Copenhagen: WHO Regional Office for Europe; 2015 (http://www.euro.who.int/en/health-topics/disease-prevention/nutrition/publications/2015/who-regional-office-for-europe-nutrient-profile-model-2015).

Annex 10

EXISTING EUROPEAN COMMISSION LABELLING REQUIREMENTS: ARTICLE 8 FROM THE COMMISSION DIRECTIVE 2006/125/EC[14]

1. The labelling of the products concerned shall bear in addition to the particulars provided for in Article 3 of Directive 2000/13/EC, the following mandatory particulars:

(a) a statement as to the appropriate age from which the product may be used, regard being had to its composition, texture or other particular properties. The stated age shall not be less than four months for any product. Products recommended for use from the age of four months may indicate that they are suitable from that age unless independent persons having qualifications in medicine, nutrition or pharmacy, or other professionals responsible for maternal and child care, advise otherwise;
(b) information as to the presence or absence of gluten if the indicated age from which the product may be used is below six months;
(c) the available energy value expressed in kJ and kcal, and the protein, carbohydrate and lipid content, expressed in numerical form, per 100 g or 100 ml of the product as sold and, where appropriate, per specified quantity of the product as proposed for consumption;
(d) the average quantity of each mineral substance and of each vitamin governed by a specific level as specified in Annex I and Annex II respectively, expressed in numerical form, per 100 g or 100 ml of the product as sold and, where appropriate, per specified quantity of the product as proposed for consumption;
(e) instructions for appropriate preparation, when necessary, and a statement as to the importance of following those instructions.

2. The labelling may bear:
(a) the average quantity of the nutrients set out in Annex IV when such declaration is not covered by the provisions of paragraph 1(d), expressed in numerical form, per 100 g or 100 ml of the product as sold and, where appropriate, per specified quantity of the product as proposed for consumption;
(b) in addition to numerical information, information on vitamins and minerals shown in Annex V, expressed as a percentage of the reference values given therein, per 100 g or 100 ml of the product as sold, and where appropriate, per specified quantity of the product as proposed for consumption, provided that the quantities present are at least equal to 15 % of the reference values.

[14] *Source*: The Commission of the European Communities. Commission Directive 2006/125/EC on processed cereal-based foods and baby foods for infants and young children. O. J. E. U. 2006, L 339:16–35 (http://eur-lex.europa.eu/legal-content/EN/TXT/PDF/?uri=CELEX:32006L0125&from=EN).

The WHO Regional Office for Europe

The World Health Organization (WHO) is a specialized agency of the United Nations created in 1948 with the primary responsibility for international health matters and public health. The WHO Regional Office for Europe is one of six regional offices throughout the world, each with its own programme geared to the particular health conditions of the countries it serves.

Member States

Albania
Andorra
Armenia
Austria
Azerbaijan
Belarus
Belgium
Bosnia and Herzegovina
Bulgaria
Croatia
Cyprus
Czechia
Denmark
Estonia
Finland
France
Georgia
Germany
Greece
Hungary
Iceland
Ireland
Israel
Italy
Kazakhstan
Kyrgyzstan
Latvia
Lithuania
Luxembourg
Malta
Monaco
Montenegro
Netherlands
North Macedonia
Norway
Poland
Portugal
Republic of Moldova
Romania
Russian Federation
San Marino
Serbia
Slovakia
Slovenia
Spain
Sweden
Switzerland
Tajikistan
Turkey
Turkmenistan
Ukraine
United Kingdom
Uzbekistan

World Health Organization Regional Office for Europe
UN City, Marmorvej 51, DK-2100 Copenhagen Ø, Denmark
Tel.: +45 45 33 70 00 Fax: +45 45 33 70 01
Email: eurocontact@who.int
Website: www.euro.who.int
